# Catalytic Asymmetric Diels–Alder Reaction of β,β-Disubstituted Enals with Cyclopentadiene: Syntheses of (−)-Camphene, (−)-β-Santalene, and (−)‑(*Z*)‑β-Santalol

**DOI:** 10.1021/jacs.6c14863

**Published:** 2026-10-27

**Authors:** Anna Iniutina, Santanu Ghosh, Nobuya Tsuji, Chandra Kanta De, Benjamin List

**Affiliations:** † Max-Planck-Institut für Kohlenforschung, D-45470 Mülheim an der Ruhr, Germany; ‡ Shiv Nadar University, Greater Noida 201314, India; § Institute for Chemical Reaction Design and Discovery (WPI-ICReDD), Hokkaido University, Sapporo 001-0021, Japan

## Abstract

Despite the long and rich history of the Diels–Alder reaction, certain aspects of its scope still remain remarkably underexplored. Most prominently, reactions with β,β-disubstituted enals as dienophiles are scarcely represented in the literature. The first general and highly enantioselective method, enabled by a strongly acidic and confined imidodiphosphorimidate organocatalyst, is reported here. The reaction tolerates a broad range of enals, including substrates with functional groups and complex alkenyl substituents, delivering the corresponding cycloadducts in good yields and excellent enantioselectivity. The synthetic utility of the method is further highlighted by concise asymmetric total syntheses of (−)-camphene, (−)-β-santalene, and (−)-(*Z*)-β-santalol from inexpensive starting materials.

## Graphical abstract

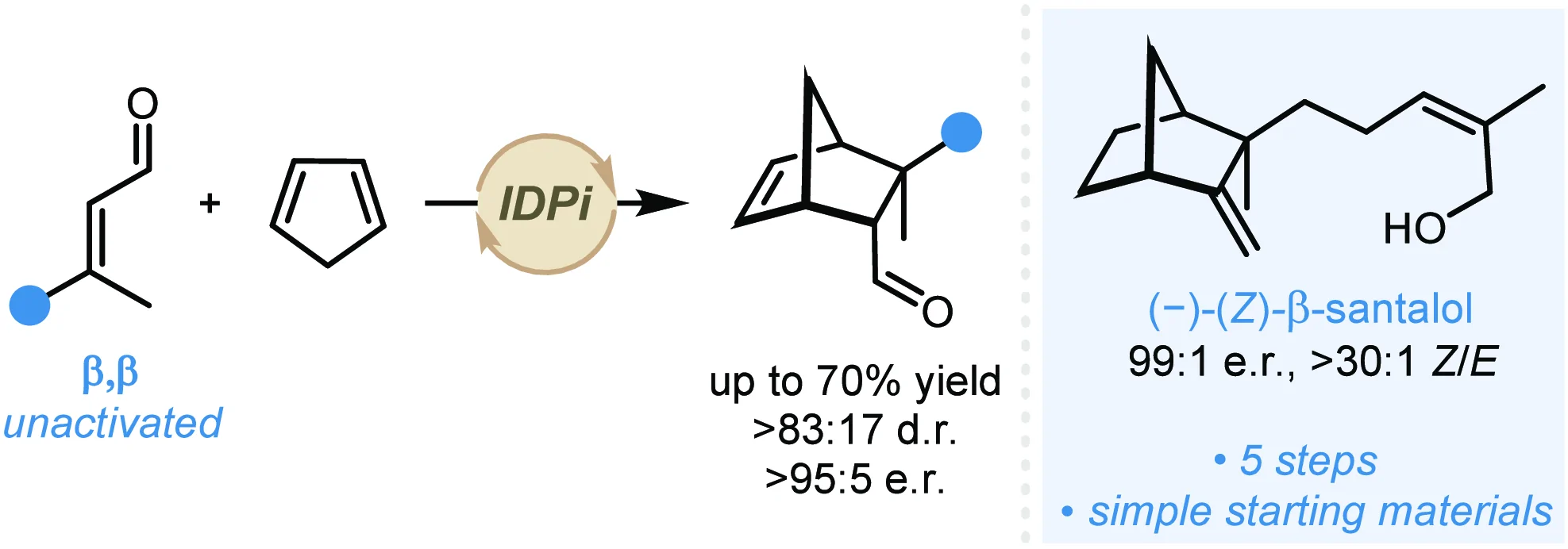

Discovered nearly a century ago, the Diels–Alder reaction has become a versatile and reliable tool for constructing complex molecules.^1^ Being essentially limited only by the HOMO_diene_–LUMO_dienophile_ energy gap (for normal electron-demand reactions) and geometric constraints, it accommodates a broad scope of compatible reaction partners which continues to expand.^2^ The reactivity of diene and dienophile as well as the (stereo)­selectivity of the cycloaddition can be enhanced through catalytic strategies.^3^ Despite this versatility, Diels–Alder reactions of β,β-disubstituted enals remain surprisingly underdeveloped. To date, only one stereoselective example involving the simplest β,β-disubstituted enal, 3-methyl-2-butenal (**1b**), has been reported (Figure 1A). Thirupathi et al. employed a “fluorine-enhanced” oxazaborolidine catalyst in combination with bis­(trifluoromethanesulfonyl)­imide and TiCl_4_ (**A**), affording the cycloadduct in 82% yield and 94:6 e.r.^4^ In the most closely related example, Hayashi et al. employed a prolinol catalyst (**B**) for the reaction of an OBz-substituted analog of **1b** with 1,3-cyclopentadiene (**2**), affording the product in moderate yield and with an e.r. of 97.5:2.5. Notably, the presence of an electron-withdrawing substituent in the dienophiles was essential for this reaction, as demonstrated by the reaction scope.^5^

**Figure 1:**
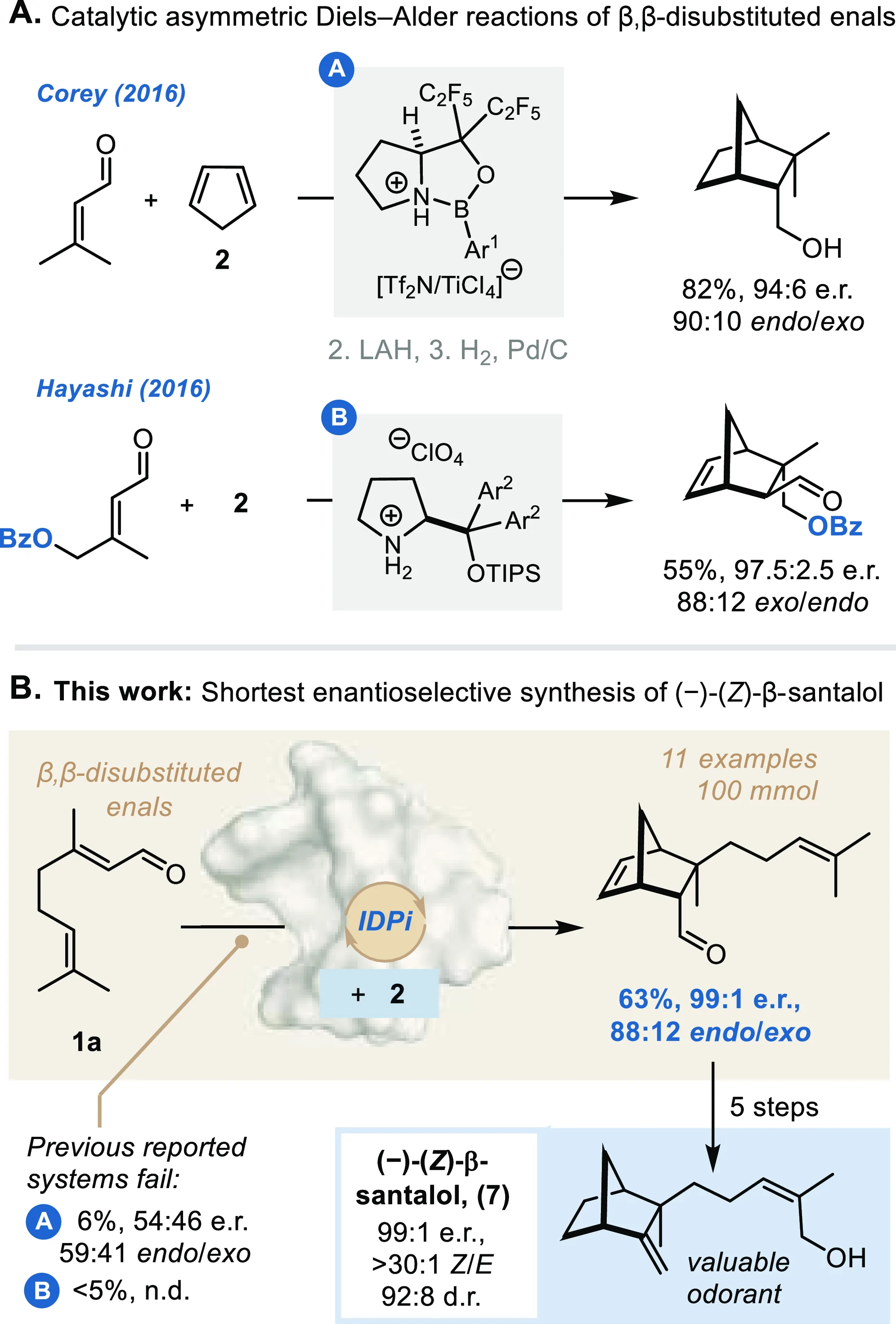
(A) Known examples of catalytic asymmetric Diels–Alder reactions with β,β-disubstituted enals. Ar^1^ = 2-tolyl, Ar^2^ = 3,5-(CF_3_)_2_C_6_H_3_. (B) Attempts to employ **1a** as a dienophile using reported systems and our approach.

The limited utility of β,β-disubstituted enals arises from both steric and electronic effects.^6^ Steric congestion around the double bond hinders the cyclic transition state, while hyperconjugation of the β-alkyl substituents raises the LUMO energy, increasing the HOMO–LUMO gap.^7,8^ Moreover, many β,β-disubstituted enals are prone to self-condensation and polymerization,^9^ particularly under strongly acidic conditions.

Despite these challenges, such a Diels–Alder reaction would provide rapid access to complex molecular architectures, establishing up to four stereocenters, including one quaternary stereocenter. This potential was recognized as early as the 1970s by Düttmann and Weyerstahl,^10^ inspired by Brieger,^11^ who proposed the reaction of **2** with citral as a key step in a concise route to β-santalene (**6**), the parent terpene of β-santalol. However, citral proved too labile under both thermal and Lewis acidic conditions, affording the desired cycloadduct in only ca. 1% yield as a racemate.

An asymmetric version of this approach would enable installation of all carbon atoms and stereocenters of (−)-(*Z*)-β-santalol (**7**)—main odor vector of sandalwood oil, in a single step from readily available chemicals. We therefore decided to revisit this reaction using modern catalytic methods. Applying systems **A** and **B** to geranial (**1a**, *E*-isomer of citral) and **2** failed to afford the desired cycloadduct (**4a**) in significant yield (Figure 1B). Moreover, system **A** is highly moisture- and air-sensitive, complicating its handling and scale-up. Prolinol catalysts (including **B**), in turn, preferentially afford *exo*-cycloadducts of β-substituted enals.

Inspired by previous success of highly acidic and confined imidodiphosphorimidate (IDPi) catalysts (**3**) in asymmetric Diels–Alder reaction of enals^12^ and disubstituted enones,^13^ we hypothesized that these catalysts may also enable the reaction between **1a** and **2**.

We initiated our catalyst screening using IDPi **3a**, which proved to be suitable for the Diels–Alder reaction of other enals, however this catalyst afforded **4a** in only 15% yield and 73:27 e.r. (Table 1, entry 1).

**Table 1:**
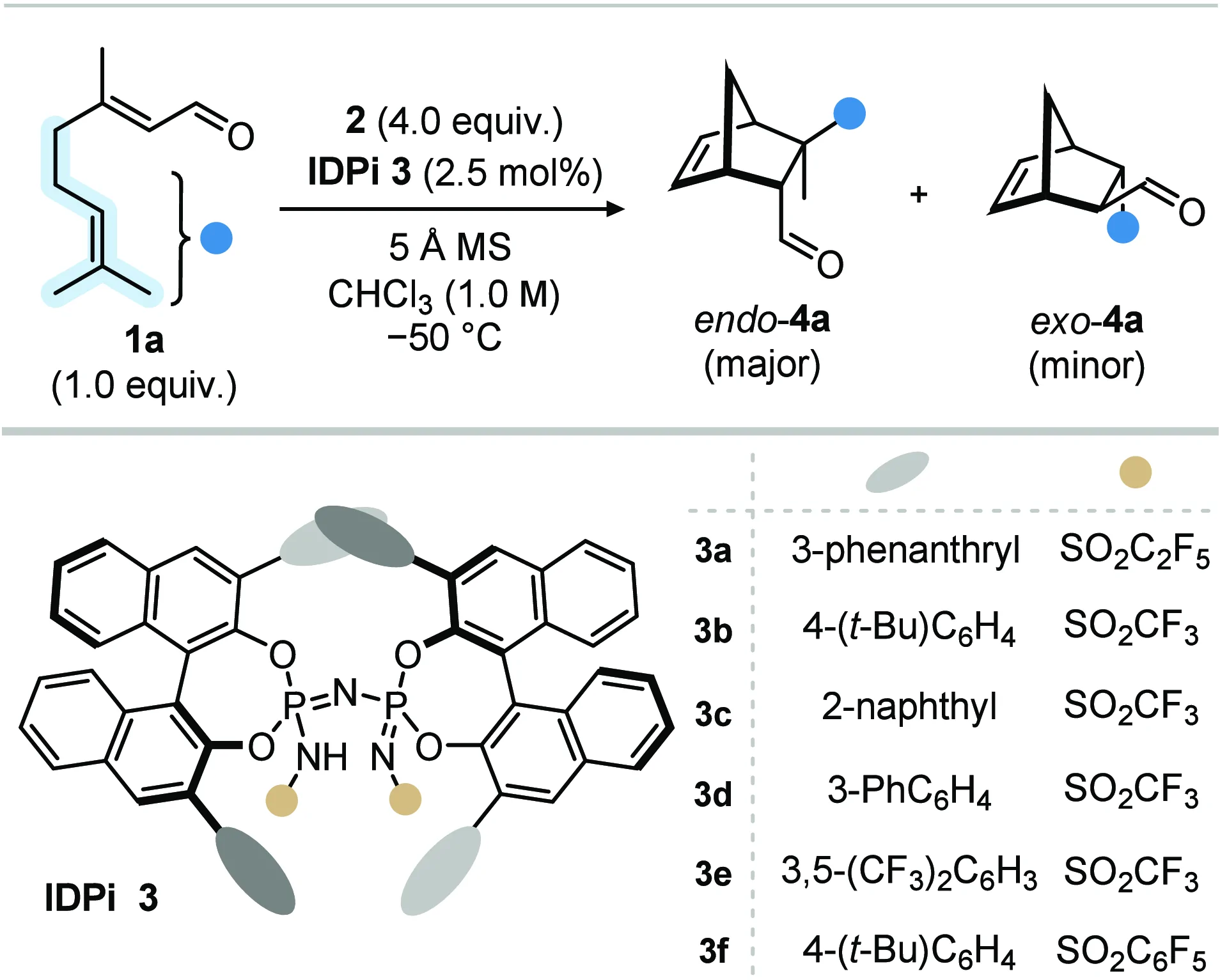
Reaction Development

entry^a^	catalyst	yield (%)^b^	*endo*/*exo*	e.r. (*endo*)^c^
1	**3a**	15	87:13	73:27
2	**3b**	21	90:10	98:2
3	**3c**	9	85:15	77:23
4	**3d**	12	77:23	73:27
5	**3e**	24	60:40	69:31
6	**3f**	27	89:11	95:5
7^d^	**3f**	41	88:12	99:1
7^e^	**3f**	50	89:11	99:1
7^f^	**3f**	59	88:12	99:1

a 0.05 mmol scale.

b Yields and *endo*/*exo* ratios were determined via ^1^H NMR using CH_2_Br_2_ as an internal standard.

c Determined by chiral GC.

d In MTBE (1.0 M).

e In MTBE (1.0 M), **2** added before **1a**.

f In MTBE (1.7 M), **2** added first, −40 °C. MS = molecular sieves. See SI, Chapter 2.2 for the details.

Screening other IDPi motifs identified **3b**, bearing bulky 4-(*t*-Bu)­C_6_H_4_ substituents, as a promising catalyst (entry 2). Product decomposition in the presence of **3b** was identified as a major obstacle. We therefore turned to the less acidic and more sterically confined C_6_F_5_-substituted analog of **3b** (**3f**, entry 6),^14^ which improved the yield while maintaining excellent enantioselectivity. Switching to methyl *tert*-butyl ether (MTBE), together with increased concentration and temperature, ultimately afforded **4a** in 59% yield, with an 88:12 *endo*/*exo* ratio and 99:1 e.r. (entry 7).

We next investigated potentially compatible β,β-disubstituted enals (Figure 2). Using our system, the enantioselective cycloaddition of **1b** afforded the corresponding product in 57% yield and e.r. of 96:4.

**Figure 2:**
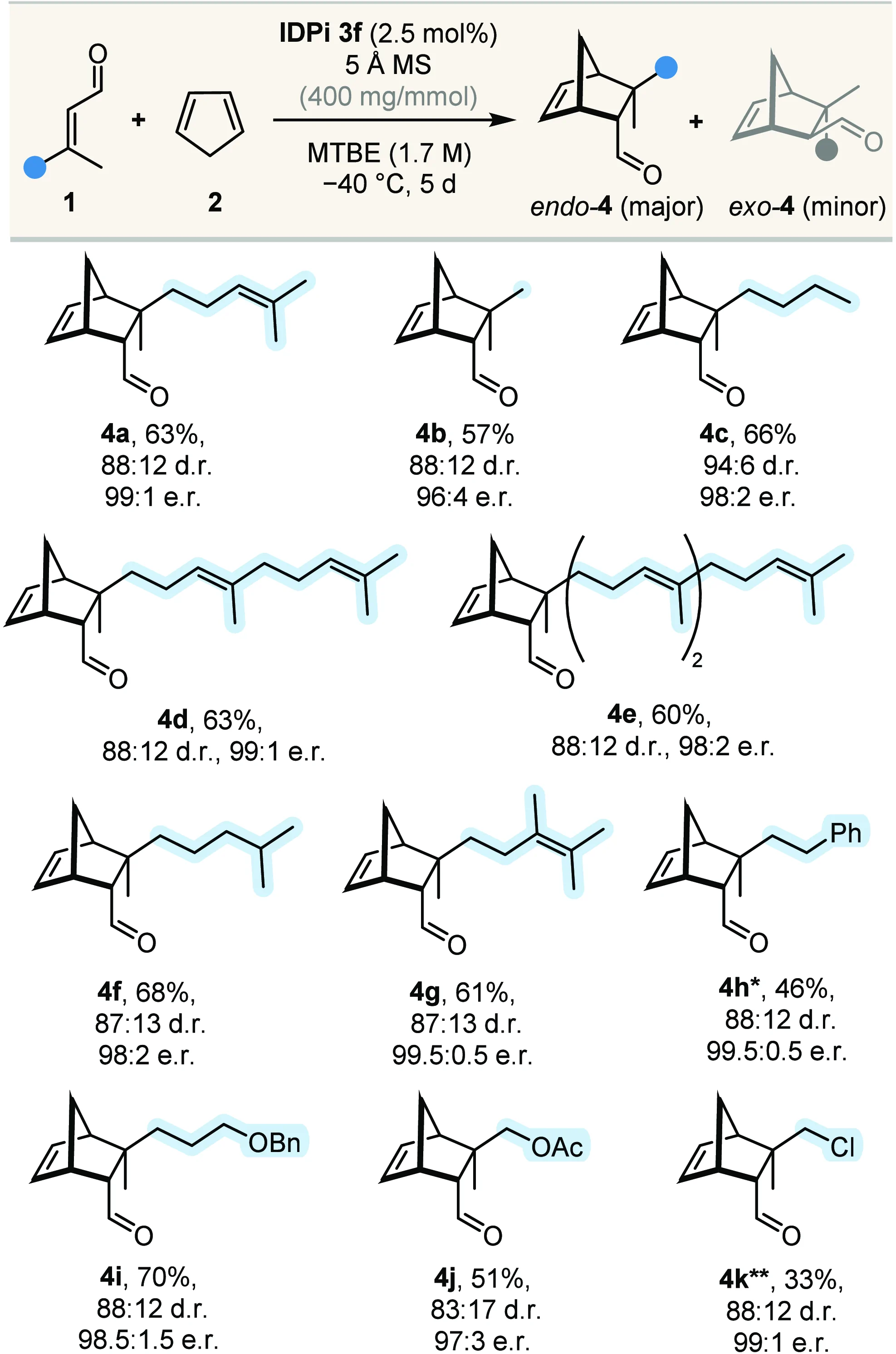
Substrate scope, 0.2 mmol scale. Isolated yields after chromatographic purification. Enantiomeric ratios for the major (*endo*) isomer are reported. *7 days, **at –60 °C.

Remarkably, one of the β-substituents could be extended to *n*-butyl (**1c**), farnesyl (**1d**), or even ‘geranylgeranyl’ (**1e**) without compromising yield or enantioselectivity. Hydrogenated (**1f**) and methyl-substituted geranial derivatives (**1g**) were likewise compatible. In the case of the less reactive phenyl-substituted enal (**1h**), the product was obtained in slightly reduced yield while maintaining excellent >99:1 e.r. Oxygen-containing substituents, including OBn (**1i**) and OAc (**1j**), were also well tolerated. Even the highly labile chloro-substituted enal (**1k**) underwent cycloaddition, providing **4k** in moderate yield with good diastereoselectivity and 99:1 e.r. Unfortunately, we found that simultaneous elongation of both β,β-substituents results in a complete loss of reactivity. Similarly, switching to less reactive dienes also did not lead to product formation. These remain current limitations of the approach (see SI, Chapter 4.2).

To further demonstrate the synthetic utility of the developed system, we applied it to the total syntheses of natural products **6**, **7**, and (−)-camphene (**9**) (Figure 3). Driven by the high value of sandalwood oil and the looming scarcity of its natural supply, the synthesis of **7** has attracted considerable attention for over five decades.^15^ To date, two catalytic enantioselective syntheses, reported by Fehr et al.^16^ and Chapuis et al.,^17^ represent the state of the art and demonstrate the industrial relevance, as evidenced by their patenting.^18,19^ Despite high stereoselectivity, both require specialized building blocks and/or protecting-group manipulations. We sought to develop a more efficient and sustainable alternative. To the best of our knowledge, no enantioselective synthesis of **9** in which the stereochemistry is established through an intermolecular reaction has been reported.

**Figure 3:**
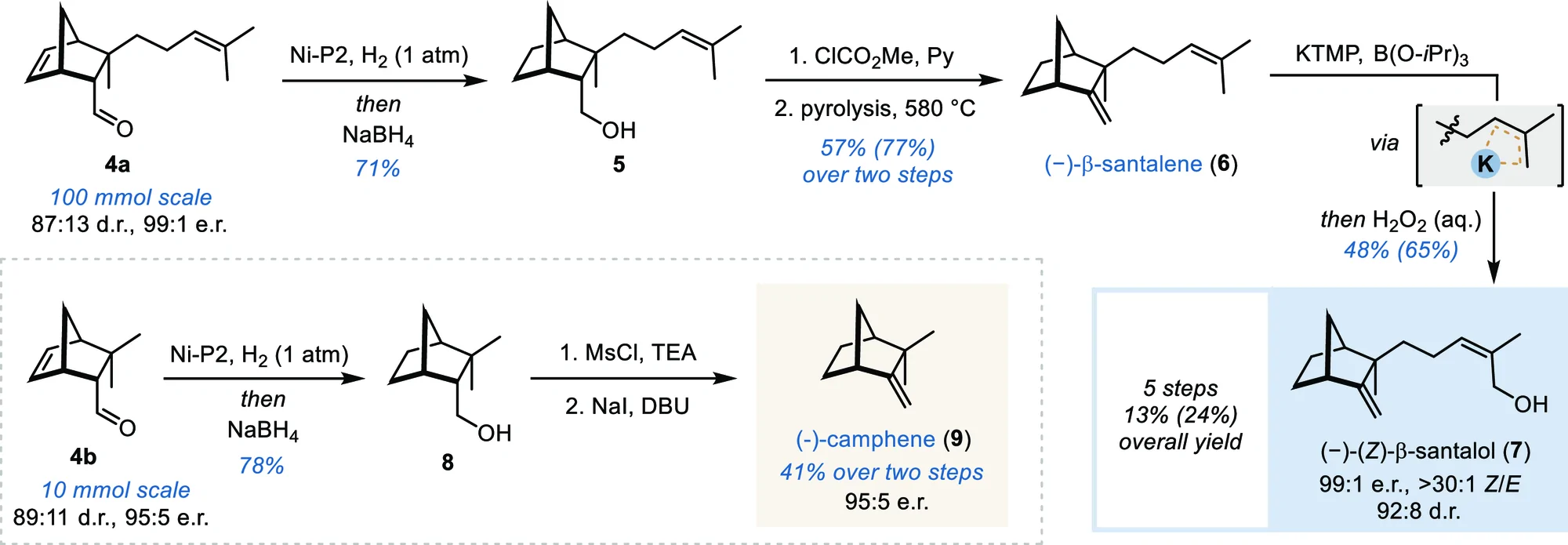
Organocatalytic enantioselective total syntheses of **7** and **9**. Yields, reported in parentheses, are based on recovered starting material. Ni–P2 is generated from Ni­(OAc)_2_·4H_2_O (0.2 equiv), NaBH_4_ (0.16 equiv), and NaOH (0.008 equiv).

The Diels–Alder reaction of **1a** proved readily scalable to 100 mmol. Extending the reaction time to 7 days allowed for a reduced loading of **3f** (1.5 mol %), while retaining good yield (67%, 14.7 g, 67.3 mmol) and excellent enantioselectivity for **4a**. Both **3f** and molecular sieves (MS) could be recovered. Moreover, a commercially available citral mixture was found to be compatible with the reaction conditions, furnishing **4a** with identical stereoselectivity (see SI, Chapters 7.1, 7.2).

Selective hydrogenation of the bicyclic double bond^20^ with nickel boride (“Ni–P2”), followed by NaBH_4_ reduction in a one-pot sequence, delivered the bicyclo[2.2.1]­heptane core of **7**. Literature precedents for elimination of the resulting alcohol in the bicyclo[2.2.1]­heptane system employ mesylate formation and require either hexamethylphosphoramide use or further transient iodide formation.^20,21^ We explored a more streamlined approach based on carbonate formation followed by pyrolysis, which enabled solvent-free elimination without additional reagents and produced only CO_2_ and methanol as byproducts. This sequence furnished sesquiterpene **6** in good yield over four steps from readily available starting materials. The same reduction–elimination sequence was applied to **4b** to access **9**. While reduction proceeded smoothly to give alcohol **8** in 78% yield, pyrolysis was inefficient, likely due to the low boiling point of **9**. We therefore turned to the mesylate route, which provided **9** in 41% yield over two steps.

Installation of the *Z*-allylic alcohol represented a significant challenge, as conventional allylic oxidation methods tend to favor the formation of the thermodynamically more stable (*E*)-isomers.^22^ Reported approaches for direct conversion of **6** to **7** rely either on engineered P450 monooxygenase enzymes^23^ or a three-step allylic chlorination/S_N_2’ sequence,^24^ but both afford mixtures of isomers.

Our attention was therefore drawn to Schlosser’s superbase-mediated deprotonation/oxidation strategy, which exploits unique preference of 2-butenylpotassium species to adopt an *endo-Z* conformation.^25,26^ To introduce an alcohol moiety the generated anion is further trapped with fluorodimethoxyborane, followed by oxidation with H_2_O_2_.^27^ We were pleased to identify an analogous system utilizing easily accessible potassium 2,2,6,6-tetramethylpiperidinide (KTMP) and triisopropyl borate reported by Clanton et al.^28^ Applying these conditions to **6** furnished **7** in 48% yield (65% brsm) and 97:3 *Z*/*E* selectivity. Overall, this approach provides **7** in just five steps and 13% (24% brsm) total yield from readily available reagents, constituting the shortest enantioselective synthesis of this compound reported to date.

To further optimize the economics of the route, we investigated whether MS could be replaced by less expensive drying agents. Surprisingly, common desiccants failed to afford the desired product in appreciable yield, prompting us to examine the interaction between IDPi catalyst and MS. A significant ^31^P NMR shift upon mixing **3f** with MS is consistent with H^+^-Na^+^ cation exchange, generating the sodium salt of IDPi (IDPi-Na) and Brønsted acid site within the zeolite framework of the MS (represented by OH-groups^29^) (Figure 4A, a.-b.).^30,31^ Neither independently IDPi-Na nor H-exchanged 5 Å MS catalyzed the reaction alone.

**Figure 4:**
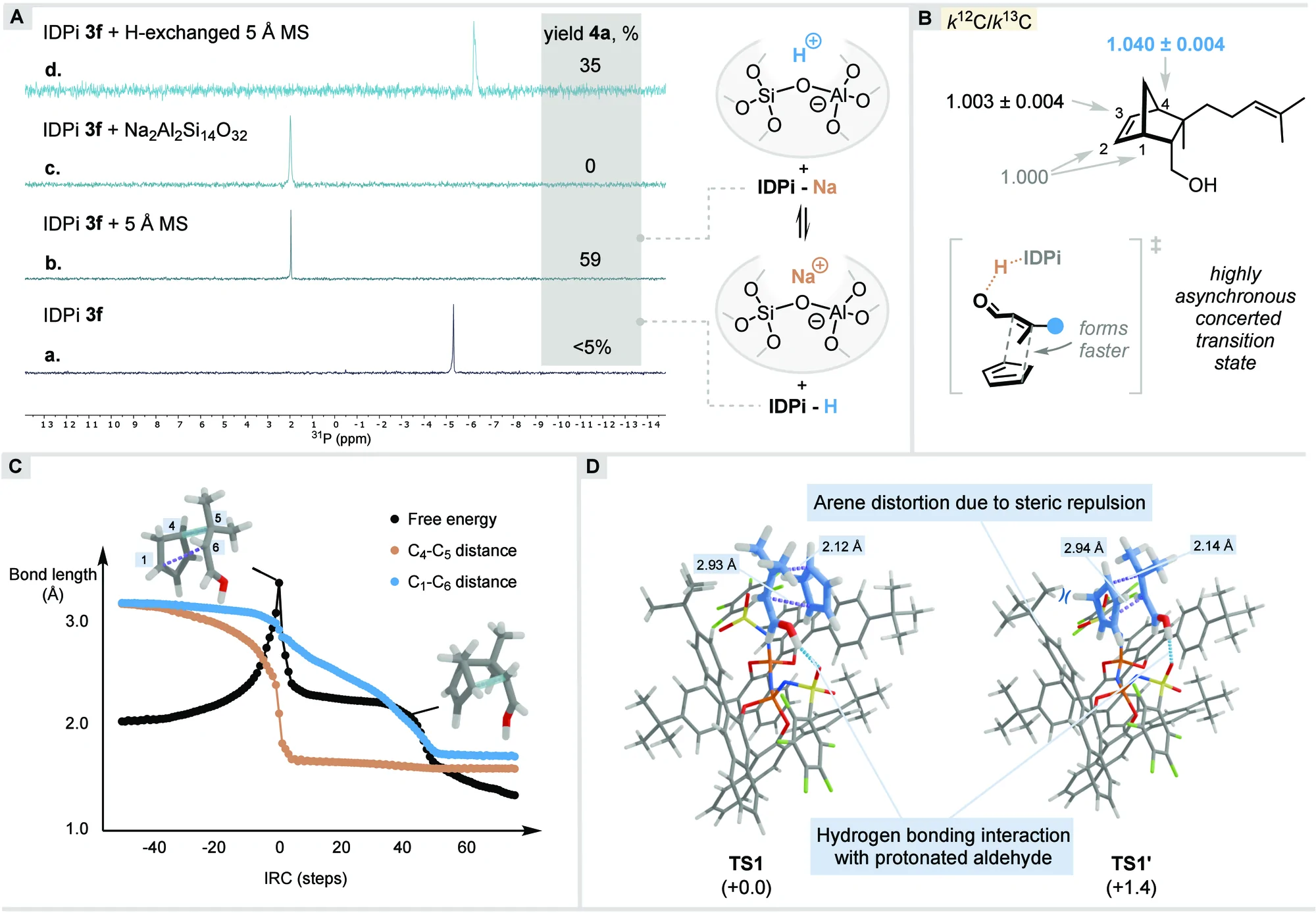
Mechanistic investigations. (A) Interactions of IDPi and molecular sieves studied by ^31^P NMR. (B) Intramolecular competition ^13^C NMR KIE studies at natural abundance. (C) IRC plot of reaction between **1b** and **2**. Geometry optimization and electronic energies were obtained at r^2^SCAN-3c level of theory. (D) Comparison of transition states leading to the major isomer (**TS1**), the minor isomer (**TS1′**). The free energies were obtained at SMD­(CHCl_3_)-ωB97M-V/def2-TZVPP//r^2^SCAN-3c (223.15 K) level of theory.

However, when H-exchanged 5 Å MS were used in combination with **3f** under the standard conditions, product formation was observed, despite no evidence for the formation of IDPi-Na (d.). Compared with the pure **3f** sample, a slight decrease in the ^31^P chemical shift was observed, presumably due to the removal of water/solvent molecules from the catalyst pocket.^32^ In contrast, sodium aluminosilicate, which has a similar elemental composition to 5 Å MS but lacks its zeolitic structure and drying properties, generated IDPi-Na (c.), but failed to promote the reaction. These observations suggest that protonated, Brønsted-acidic form of **3f** is the active catalytic species, while the corresponding sodium salt potentially acts as a dormant “reservoir form,” preventing deleterious acid-catalyzed reactions of enals. Furthermore, the strongly desiccating effect of MS is required for efficient catalysis. The precise nature of this interplay remains to be elucidated.

To gain deeper insight into origin of the observed reactivity, we performed intramolecular ^13^C NMR kinetic isotope effect (KIE) studies at natural abundance (Figure 4B). A substantial KIE^33^ was observed at C4, exceeding those reported for IDPi-catalyzed Diels–Alder reactions of substituted enones,^13^ oxazaborolidine-catalyzed reactions of benzoquinone,^35^ and Lewis acid-catalyzed reactions of acrolein.^36^ This result is consistent with a highly asynchronous concerted transition state. A high degree of asynchronicity has been identified as a key factor in lowering the activation barrier for Diels–Alder reactions involving similar dienophiles.^37^

Computational studies of the reaction between **1b** and **2** catalyzed by IDPi **3f** further supported this picture (Figure 4C). The reaction proceeds through transition state (**TS1**) toward *endo*-**4b**. Whereas the C4–C5 bond is formed at the transition state, the C1–C6 bond length is 2.93 Å. The asynchronicity of the reaction is visualized by plotting electronic energy and the forming bond lengths against the intrinsic reaction coordinate (IRC). There is an evident shoulder on the IRC profile, which corresponds to the spontaneous formation of the C1–C6 bond.

To elucidate the origin of enantioselectivity, we also calculated the transition state leading to the minor enantiomer (**TS1′**; Figure 4D). **TS1** is 1.4 kcal/mol lower in energy than **TS1′**, reproducing the experimental 95:5 e.r. (see SI, Chapter 2.2) closely (96:4 calculated). In both transition states, protonated aldehyde **1b** is anchored within the IDPi active site, with the diene approaching from either face. The energy difference primarily arises from steric interactions: **TS1′** requires distortion of the BINOL 3,3′-substituent to reduce steric repulsion between the *tert*-butyl group and diene **2**. Distortion–interaction analysis^38^ further identifies distortion of the IDPi anion as the dominant contributor to stereoselectivity (see SI, Chapter 8).

This work establishes a highly enantioselective organocatalytic Diels–Alder reaction of unactivated β,β-disubstituted enals, representing the first general application of these dienophiles. Its synthetic utility is demonstrated by successful scale-up to 100 mmol and application to concise asymmetric total syntheses of (−)-camphene and (−)-β-santalene from readily available bulk chemicals, culminating in the shortest enantioselective synthesis of the valuable odorant (−)-(*Z*)-β-santalol reported to date.

## Supplementary Material



## Notes

Additional experimental details, compound synthesis and characterization (PDF)

## Abbreviations

HOMO: highest occupied molecular orbital
LUMO: lowest unoccupied molecular orbital
EWG: electron-withdrawing group
Tf: triflyl
n.d.: not determined
IDPi: imidodiphosphorimidate
*i*-Pr: *iso*-propyl
MS: molecular sieves
equiv.: equivalents
e.r.: enantiomeric ratio
CpH: cyclopentadiene
*t*-Bu: *tert*-butyl
*n*-Bu: normal butyl
MTBE: methyl *tert*-butyl ether
GC: gas chromatography
Ph: phenyl
SI: Supporting Information
Bn: benzyl
Ac: acetyl
KTMP: potassium 2,2,6,6-tetramethylpiperidinide
brsm: based on recovered starting material
KIE: kinetic isotope effect
IRC: intrinsic reaction coordinate
