# Supplementary material for: Catalytic Asymmetric Diels–Alder Reaction of β,β-Disubstituted Enals with Cyclopentadiene: Syntheses of (−)-Camphene, (−)-β-Santalene, and (−)‑(Z)‑β-Santalol
Source: J Am Chem Soc. 2026 Oct 27;148(44):47666–71. doi: 10.1021/jacs.6c14863 (PMC13761267; doi:10.1021/jacs.6c14863)
Supplement: Supplementary file 1 [file ja6c14863_si_001.pdf]

Supporting Information for

Catalytic Asymmetric Diels−Alder Reaction of β,β-Disubstituted Enals with Cyclopentadiene: Syntheses of (−)-Camphene, (−)-β-Santalene, and (−)-(*Z*)-β-Santalol

Anna Iniutina, Santanu Ghosh, Nobuya Tsuji, Chandra Kanta De, Benjamin List*

*E-mail: list@kofo.mpg.de

Table of Contents

1. General Information 4
2. Method Development 7
   2.1. Evaluation of Existing Catalytic Systems 7
   2.2. Reaction Conditions Optimization 11
3. Substrates Synthesis 14
   3.1. Synthesis via Horner–Wadsworth–Emmons reaction (GP1) 14
   3.2. Synthesis via Oxidation of Alcohols (GP2) 17
4. Organocatalytic Diels−Alder Reaction of β,β-Disubstituted Enals (GP3) 19
   4.1. Substrate Scope 22
   4.2. Limitations of the Method 30
5. IDPi Catalyst Synthesis and Characterization 33
6. Mechanistic Experiments 34
   6.1. Stoichiometry Experiment 34
   6.2. Interactions of the IDPi catalyst and MS 34
   6.3. Characterization of Molecular Sieves after the Diels−Alder Reaction 37
   6.4. Intramolecular Competition ^{13}C Kinetic Isotope Effect at Natural Abundance 38
7. Total Syntheses of (−)-β-Santalene, (−)-(*Z*)-β-Santalol, and (−)-Camphene 40
   7.1. Upscaling of the Diels−Alder Reactions 40
   7.2. Citral as a Geranial Source 41
   7.3. Intermediate Alcohols 4a-R and 5 42
   7.4. Intermediate Alcohol 8 43
   7.5. Intermediate Carbonate 5-C 44
   7.6. (−)-β-Santalene 6 45
   7.7. (−)-Camphene 9 45
   7.8. (–)-(*Z*)-β-Santalol 7 46
   7.9. (–)-(*E*)-β-Santalol 7-(*E*) 47
8. Computational Studies 49

8.1. Methods........49
8.2. Benchmark Experiment for the Computational Study........49
8.3. Results and Discussion........49
8.4. Cartesian Coordinates of the Optimized Structures........52
9. NMR Spectra........100
9.1. NMR Spectra of Enal Substrates 1........100
9.2. NMR Spectra of Diels−Alder Products 4........105
9.3. NMR Spectra of the Total Syntheses Intermediates........118
9.4. NMR Spectra of Catalysts........127
10. GC and HPLC traces........130
11. References........146

1. General Information

Unless otherwise stated, all reactions were magnetically stirred and conducted in flame-dried glassware in anhydrous solvents under Ar, applying standard Schlenk techniques. Solid reagents were added through a weak Ar counter-flow. Cooling baths were prepared in Dewar vessels, filled with ice/water (0 °C) or dry ice/acetone (−78 °C). Heated silicon oil baths were used for reactions requiring elevated temperatures. Solvents were removed under reduced pressure at 40 °C using a rotary evaporator. All given yields are isolated yields of chromatographically and NMR spectroscopically pure materials, unless otherwise stated.

Chemicals

Chemicals were purchased from commercial suppliers and used without further purification unless otherwise stated. All starting aldehydes were purified by vacuum distillation and stored under Ar at -20 °C.

Geranial (*E/Z* 95:5) **1a-(*E*)**, neral (*Z/E* 95:5) **1a-(*Z*)** and geranylgeraniol were a kind gift of BASF. (*E*)-3,6,7-trimethylocta-2,6-dienal **1g** was obtained from KOFO MPI warehouse.

The following compounds were synthesized from commercially available starting materials according to literature procedures: ((perfluorophenyl)sulfonyl)phosphorimidoyl trichloride and 3,3'-disubstituted BINOL.[1]

IDPi catalysts were synthesized according to the reported procedures: **3a**[2], **3b**-**3f**[3,4]. Alternatively, some of the IDPi catalysts can now also be purchased from commercial suppliers (TCI).

Solvents

Solvents were dried by distillation from an appropriate drying agent in the technical department of the Max-Planck-Institut für Kohlenforschung and received in Schlenk flasks under argon and stored over 4 Å when applicable.

Thin Layer Chromatography (TLC)

Was performed using silica gel pre-coated glass plates (SIL G-25, with fluorescent indicator UV254; Macherey-Nagel) which were visualized by irradiation with UV light (λ = 254 or 366 nm), *p*-anisaldehyde, basic $KMnO_4$, and/or phosphomolybdic acid (PMA) stains. Preparative thin-layer chromatography was performed on silica gel pre-coated glass plates SIL G-100, with fluorescent indicator UV254 (Macherey-Nagel).

Column Chromatography

Column chromatography was carried out using Merck silica gel (60 Å, 230−400 mesh, particle size 0.040−0.063 mm) using technical grade solvents. Elution was accelerated using Ar stream. Automated column chromatography was conducted on a Biotage® Isolera ISO-4SW instrument, using Biotage® Sfär chromatography cartridges. All fractions containing a desired substance were combined and concentrated in vacuo, then redissolved in an appropriate solvent and filtered through cotton to remove silica residues.

Nuclear Magnetic Resonance Spectroscopy

^{1}H, ^{13}C, ^{19}F, ^{31}P nuclear magnetic resonance (NMR) spectra were recorded on a Bruker Avance III 500 or a Bruker Avance Neo 600 MHz (equipped with a BBO CryoProbe) spectrometer in a suitable deuterated solvent. Chemical shifts are reported in ppm relative to the residual protonated solvent peaks or ^{13}C peaks of the solvents employed as references. The resonance multiplicity is described as: s (singlet), d (doublet), t (triplet), q (quartet), p (pentet), hept (heptet), m (multiplet), and b (broad). All spectra were recorded at 298 K unless otherwise noted, processed with MestReNova 15.0.0, and coupling constants are reported as observed.

Mass Spectrometry

Electrospray ionization (ESI) mass spectrometry was conducted on a Bruker ESQ 3000 spectrometer. High resolution mass spectrometry (HRMS) was performed on a Finnigan MAT 95 (EI) or Bruker APEX III FTMS (7 T magnet, ESI). The ionization method and mode of detection employed is indicated for the respective experiment and all masses are reported in atomic units per elementary charge (m/z) with an intensity normalized to the most intense peak.

Specific rotations

Were measured with a Rudolph RA Autopol IV Automatic Polarimeter at the indicated temperature (T) with a sodium lamp (sodium D line, $\lambda = 589$ nm). Measurements were performed in an acid resistant 1 mL cell (50 mm length) with concentrations (g/(100 mL)) reported in the corresponding solvent.

High-Performance Liquid Chromatography (HPLC)

Was performed on a Shimadzu LC-20AD liquid chromatograph SIL-20AC auto sampler, CMB-20A using Daicel/Merck columns with a chiral stationary phase. All solvents used were HPLC-grade solvents purchased from Sigma-Aldrich. The column employed and the respective solvent mixture are indicated for each experiment.

Gas Chromatography (GC)

GC analyses on a chiral stationary phase were performed on HP 6890 and 5890 series instruments (split-mode capillary injection system, flame ionization detector (FID), hydrogen carrier gas). The conditions employed are described in detail for the individual experiments.

The grammar and language refinement in certain parts of this document were assisted by GPT-5.6 (OpenAI). The output was checked and approved by the authors.

Abbreviations: Ar = argon, DCM = dichloromethane, DIBAL = diisobutylaluminum hydride, EA = ethyl acetate, e.e. = enantiomeric excess, e.r. = enantiomeric ratio, equiv. = equivalents, FG = functional group, GC = gas chromatography, GP = general procedure, IDPi = imidodiphosphorimidate, KIE = kinetic isotope effect, MTBE = methyl *tert*-butyl ether, MS = molecular sieves, rt = room temperature, TEA = triethylamine, TLC = thin layer chromatography.

2. Method Development

2.1. Evaluation of Existing Catalytic Systems

- *Cationic Chiral Fluorinated Oxazaborolidines*

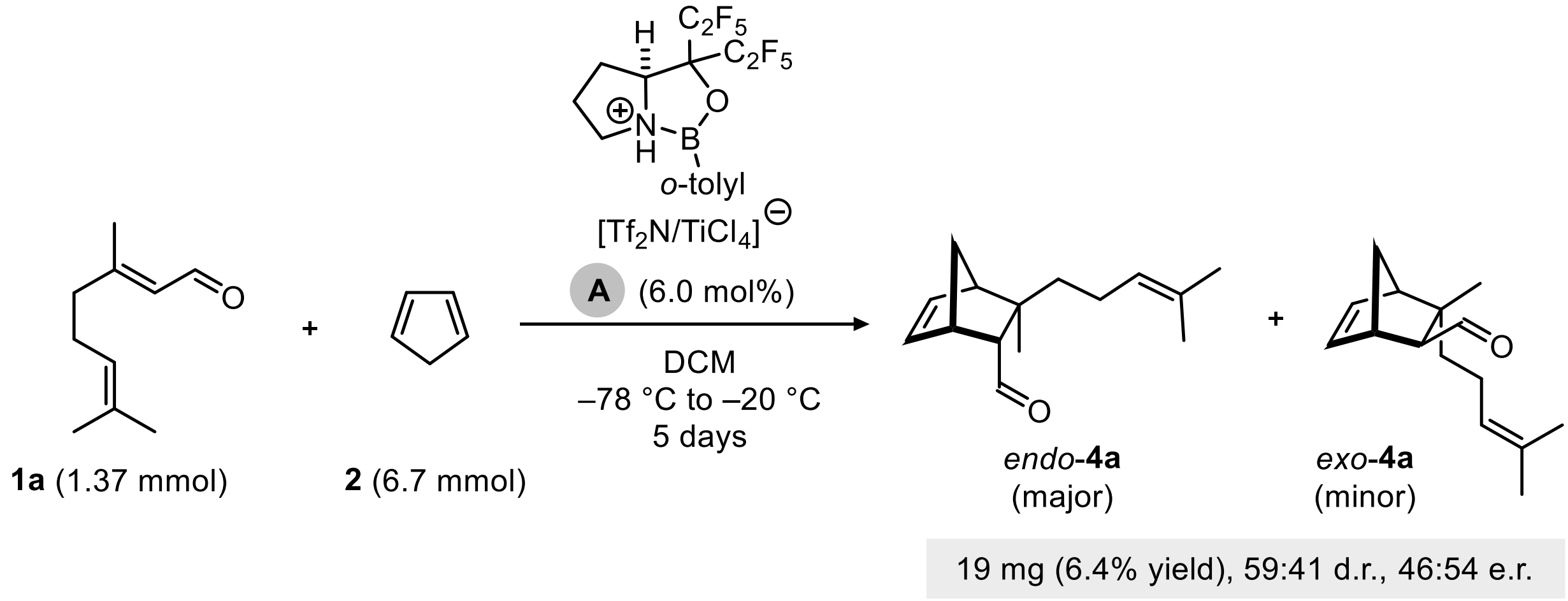

The catalyst was prepared according to the previously reported procedure; NMR spectroscopy data was in agreement with the literature.[5,6]

^{1}H NMR (501 MHz, CD_2Cl_2) δ 7.65 – 7.60 (m, 1H), 7.55 – 7.49 (m, 1H), 7.35 – 7.29 (m, 2H), 4.42 (t, J = 7.0 Hz, 1H), 3.42 – 3.34 (m, 1H), 3.31 – 3.21 (m, 1H), 2.43 (s, 3H), 2.12 – 1.99 (m, 4H).

The reaction conditions were adopted from a reported example employing 3-methyl crotanaldehyde dienophile.[6]

A ~0.2 M solution of the oxazaborolidine in benzene (0.45 mL) was placed in a 25 mL flame-dried weighted Schlenk flask and the solvent was removed under reduced pressure on a Schlenk line. The residue (39.5 mg, 0.09 mmol) was taken up in anhydrous DCM and the resulting clear solution was cooled to –78 °C. A freshly prepared, pre-mixed 0.3 M solution of Tf_2NH (19.0 mg, 0.06 mmol) and $TiCl_4$ (68.0 µL, 0.06 mmol, 1 M solution in DCM) was added and the resulting pale red coloured solution was stirred at –78 °C for 20 min. To the resulting solution at –78 °C, was added **1a** (234.0 µL, 1.37 mmol), followed by slow addition of **2** (555.0 µL, 6.7 mmol) over a period of one hour. The reaction mixture was stirred at –20 °C for 5 days and then quenched with Et_3N (50 µL), and brought to 23 °C. The solvent was removed by rotary evaporation and the crude product was purified by silica gel column chromatography (see GP3 for the details of purification) affording 19 mg (6.4% yield) of the product.

Despite the prolonged reaction time in our hands, this catalytic system delivered only 6.4% yield of the desired Diels–Alder product. Significant isomerization of the starting enal **1a** to its (*Z*)-

isomer was observed (Figure S1). This isomer was found to be unreactive in the target Diels–Alder reaction (see Chapter 7.2).

^{1}H NMR spectrum of ***1a***

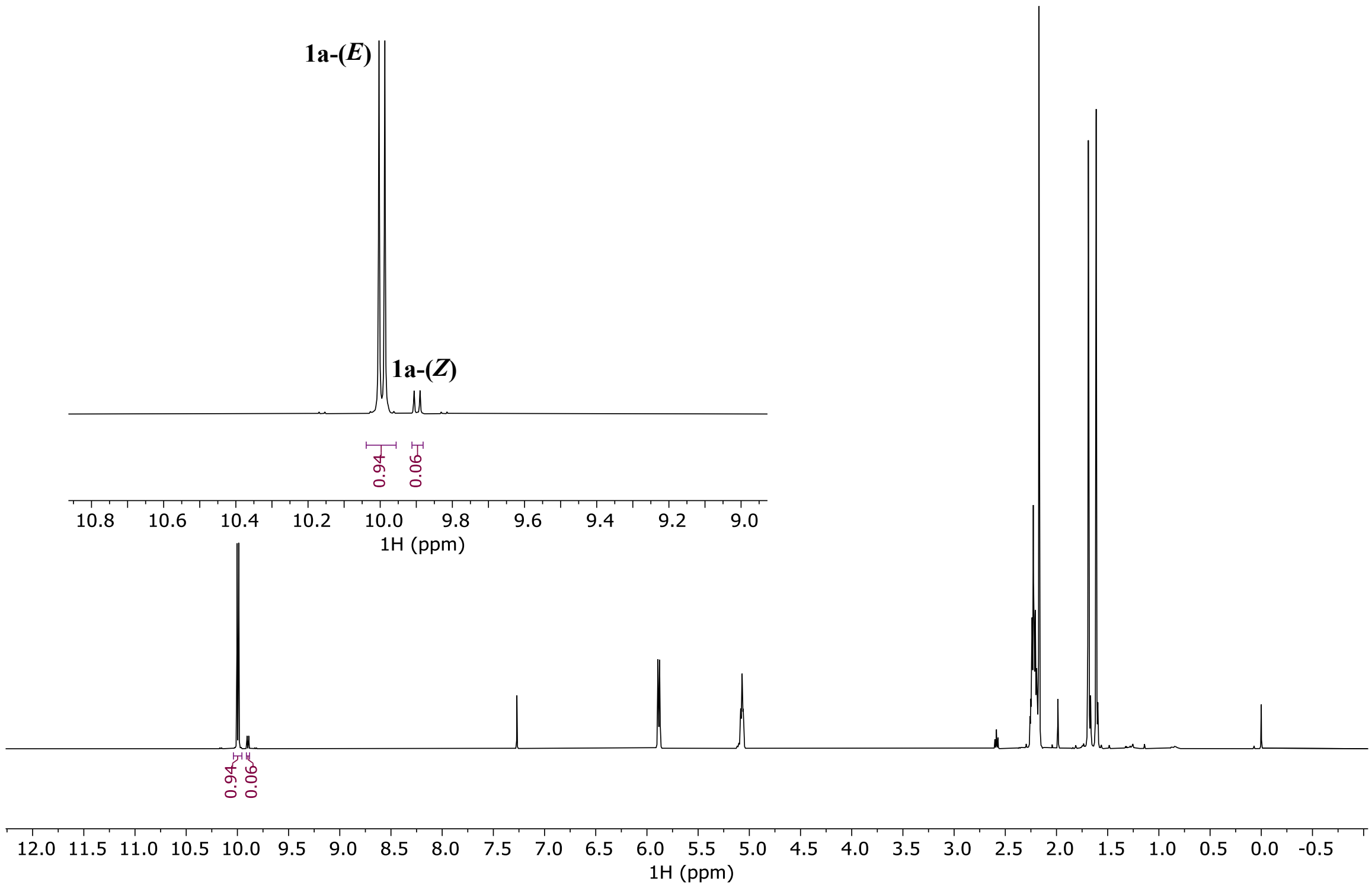

^{1}H NMR spectrum of the crude reaction mixture after 5 days

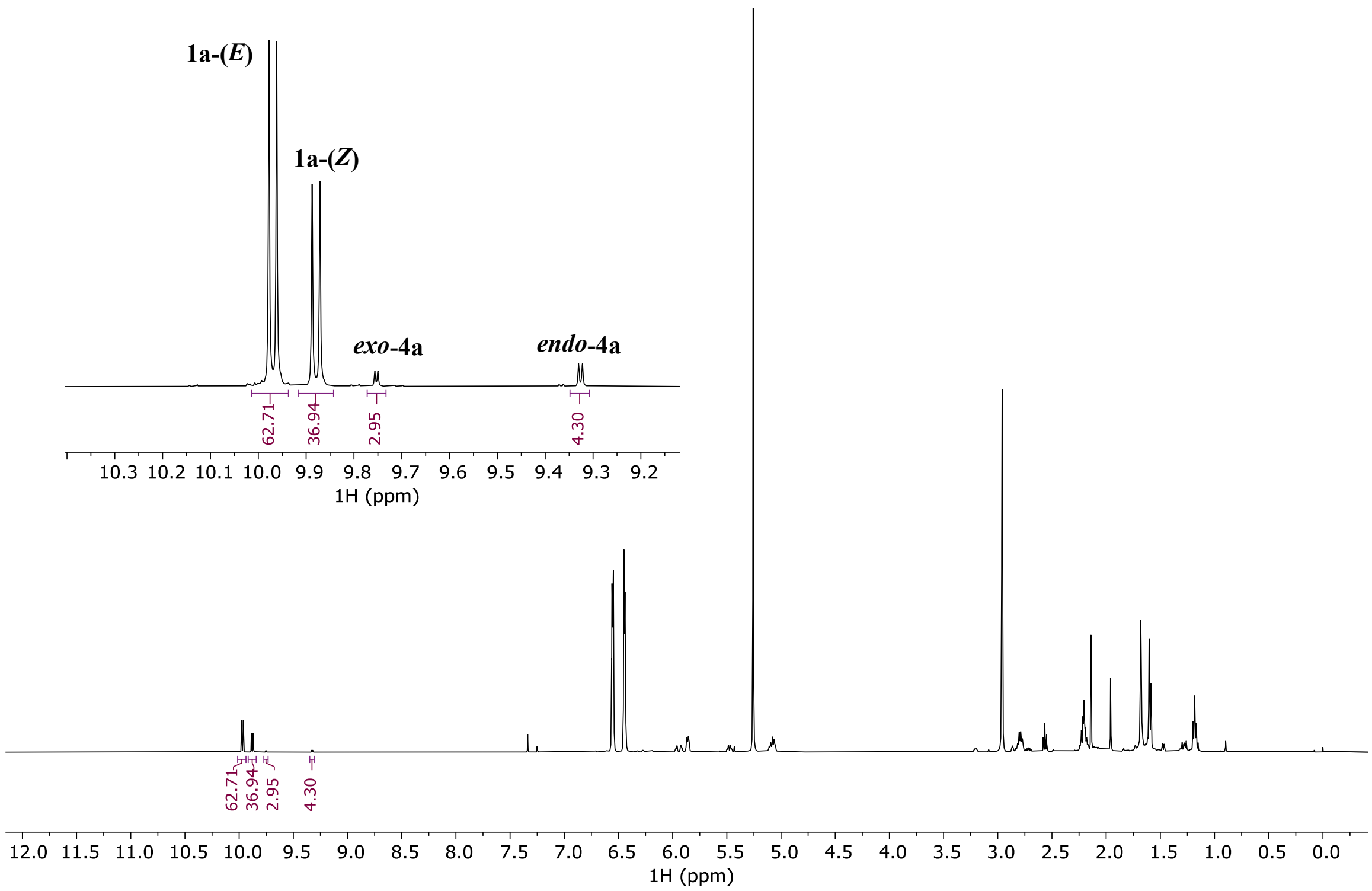

Figure S1. Evaluation of the chiral oxazaborolidine catalyst (**A**)

- *Diarylprolinol Silyl Ethers*

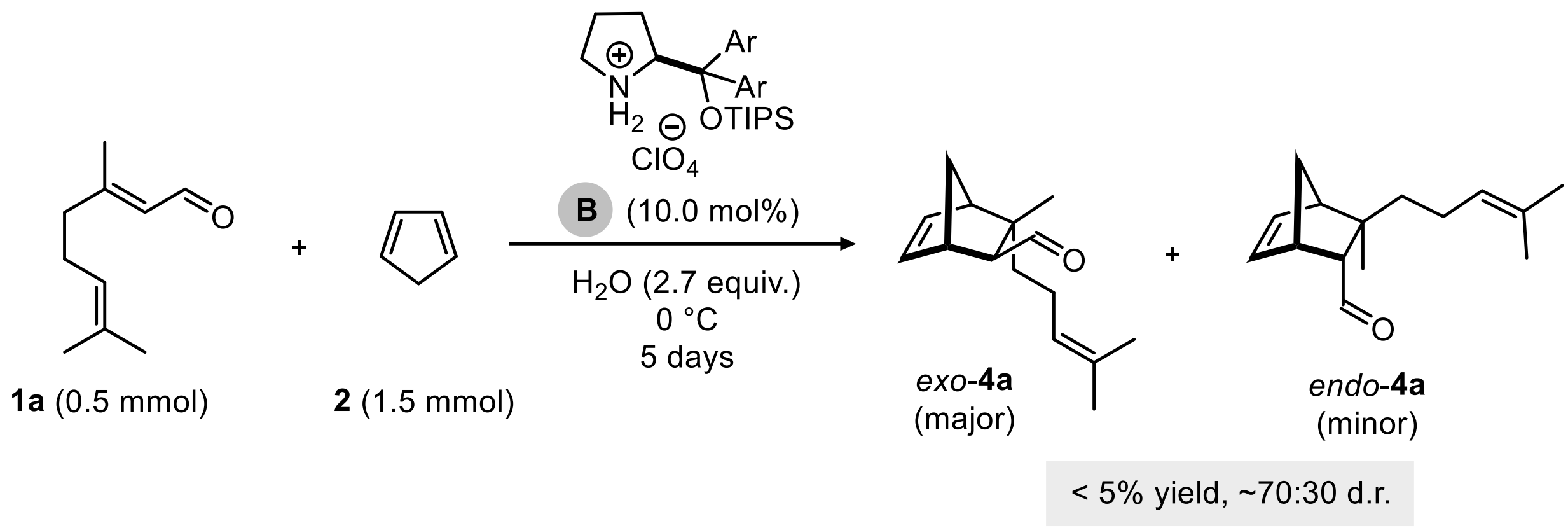

The catalyst was prepared according to the previously reported procedure; NMR spectroscopy data was in agreement with the literature.[7,8] <u>*Note*</u>: DCE was used instead of DCM for TIPS-protection described by Zhang and Wang[7]; ninhydrin stain was used to visualize the products and starting materials during the catalyst synthesis.

^{1}H NMR (501 MHz, CD_2Cl_2) δ 8.50 (bs, 1H), 8.07 (s, 1H), 8.05 (s, 1H), 7.87 (s, 2H), 7.84 (s, 2H), 6.17 (bs, 1H), 4.95 (p, J = 7.3 Hz, 1H), 3.66 – 3.58 (m, 1H), 2.87 (dt, J = 13.0, 6.9 Hz, 1H), 2.51 (dtd, J = 12.9, 8.0, 4.6 Hz, 1H), 2.20 (dt, J = 13.5, 8.1 Hz, 1H), 2.02 (dt, J = 13.7, 8.2 Hz, 1H), 1.79 – 1.66 (m, 1H), 0.98 – 0.92 (m, 18H), 0.88 – 0.74 (m, 3H).

The reaction conditions were adopted from a reported example employing (*E*)-2-methyl-4-oxobut-2-enyl benzoate dienophile.[8]

To a stirred heterogeneous mixture of the catalyst (39.0 mg, 0.05 mmol), **1a** (85.5 µL, 0.5 mmol) and water (25.0 µL) was added **2** (123.0 µL, 1.5 mmol) at 0 °C. After 5 days the reaction mixture was quenched with saturated $NaHCO_3$ solution, extracted with ethyl acetate and dried over Na_2SO_4. The organic phase was evaporated by rotary evaporator under reduced pressure to leave the crude reaction mixture.

Despite the prolonged reaction time in our hands this catalytic system failed to yield significant amount of the desired Diels−Alder product. As expected, the Diels–Alder reaction of β-substituted enals catalysed by prolinol catalysts preferentially afforded the *exo*-isomer of the product (Figure S2). In contrast to the example above, we observed only minor isomerization of **1a**; **1a** remained almost intact after 5 days. Relatively high reaction temperatures (0 °C) and prolonged reaction times presented an additional challenge: under these conditions, cyclopentadiene **2** undergoes dimerization and is prone to evaporation.

^{1}H NMR spectrum of the crude reaction mixture after 5 days

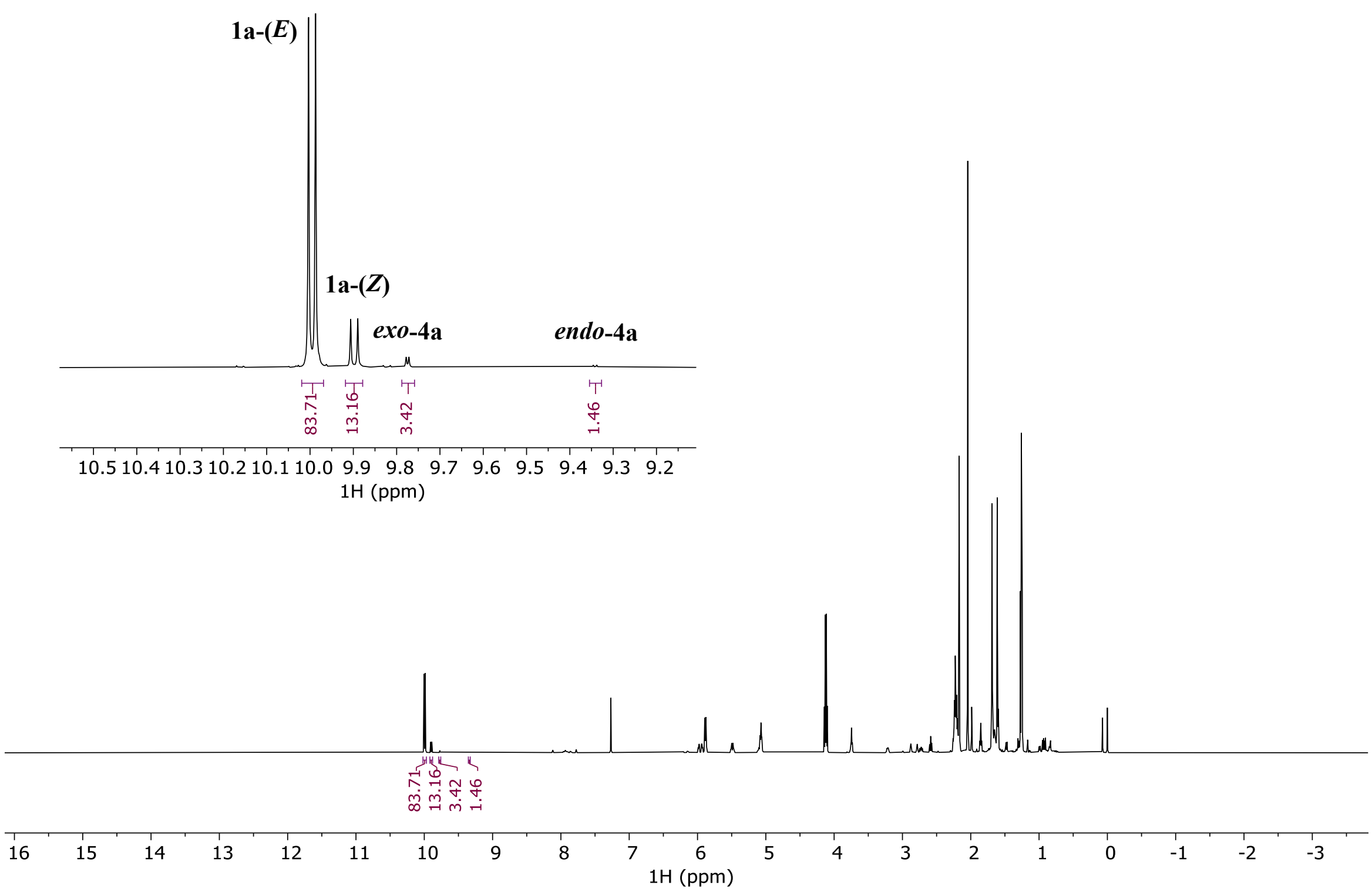

Figure S2. Evaluation of the chiral prolinol catalyst (**B**)

2.2. Reaction Conditions Optimization

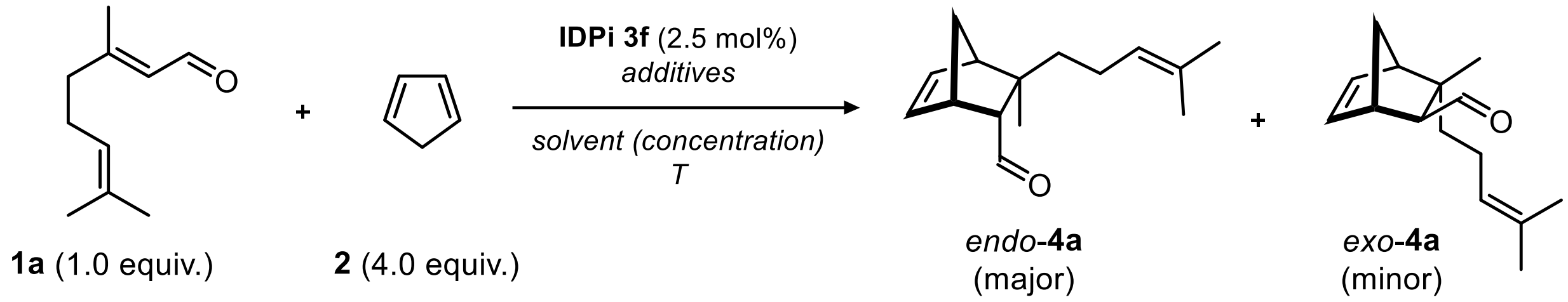

According to GP3. The screening was performed on 0.05 mmol scale. After quenching, the reaction mixture was processed as follows: filtered through a syringe filter and transferred to NMR tube diluting with 300 μL of DCM-*d2*. The NMR yield, conversion and *endo*/*exo* ratio were determined in relation to CH_2Br_2 as internal standard. Then the product was isolated using preparative TLC and analysed with chiral GC to determine enantiomeric ratios.

Table S1. *Reaction Conditions Optimization*

solvent	conver-sion, %	yield, %	*endo*/*exo*	e.r. (*endo*)
DCM	78	12	80:20	92:8
$CHCl_3$	89	27	89:11	95:5
toluene	33	0	-	-
Et_2O	75	33	85:15	94:6
MTBE	full	41	88:12	99:1
CpH (neat)	82	19	88:12	96:4

1.0 M, −50 °C, 5 d, 5 Å MS (20 mg)

order of addition of SM	conver-sion, %	yield, %	*endo*/*exo*	e.r. (*endo*)
MS-**3f**-**1a**-**2**	full	41	88:12	99:1
MS-**3f**-**2**-**1a**	98	50	89:11	99:1
MS-**1a**-**2**-**3f**	55	14	-	-
**3f**-**1a**-**2**-MS	71	21	-	-
standard + syringe pump 1a over 3 d*	76	20	-	-

MTBE (1.0 M), −50 °C, 5 d, 5 Å MS (20 mg), MS = 5 Å MS, * after the addition was complete (3 d) reaction was left for additional 3 d

concentration	conver-sion, %	yield, %	*endo*/*exo*	e.r. (*endo*)
0.5 M	88	14	91:9	-
1.0 M	98	50	89:11	99:1
1.5 M	full	53	88:12	-
2.4 M	56*	10	-	-
1.7 M	full	56	89:11	99:1

MS-**3f**-**2**-**1a**, MTBE, −50 °C, 5 d, 5 Å MS (20 mg)

*no proper stirring

***T*, °C**	conver-sion, %	yield, %	*endo*/*exo*	e.r. (*endo*)
−30	full	38	80:20	93:7
−40	full	59	88:12	99:1
−50	full	56	89:11	99:1
−60	95	45	91:9	99:1
−70	77	24	91:9	99:1

MS-**3f**-**2**-**1a**, MTBE (1.7 M), 5 d, 5 Å MS (20 mg)

additives	none	Na_2SO_4*	$MgSO_4$	$CaCl_2$	$CaSO_4$	3 Å MS**	4 Å MS	SiO_2 (various forms)
conversion, %	29	18	39	31	11	22	39	~27
yield, %	1	0	2	2	0	4	15	traces
endo/*exo*	-	-	-	-	-	-	85:15	-
e.r. (*endo*)	-	-	-	-	-	-	98:2	-

additives	5 Å MS (100 mg/mmol)	5 Å MS (250 mg/mmol)	5 Å MS (300 mg/mmol)	5 Å MS (400 mg/mmol)***	Zeolite Y (Na form)	Al_2O_3 (various forms)	Zeolite Y (H form)
conversion, %	66	87	99	full	32	~25	26
yield, %	17	51	56	59	0	traces	0
endo/*exo*	85:15	89:11	88:12	88:12	-	-	-
e.r. (*endo*)	98:2	99:1	99:1	99:1	-	-	-

in MTBE (1.7 M), 5 d, 20 mg of an additive, −40 °C; *the salts were grinded and dried at 100 °C for 2 d prior to use; **dried in high vacuum at 130 °C for 2 d; ***further increasing of MS loading led to problems with stirring

Decomposition of the Diels–Alder Product in the Presence of IDPi Catalysts

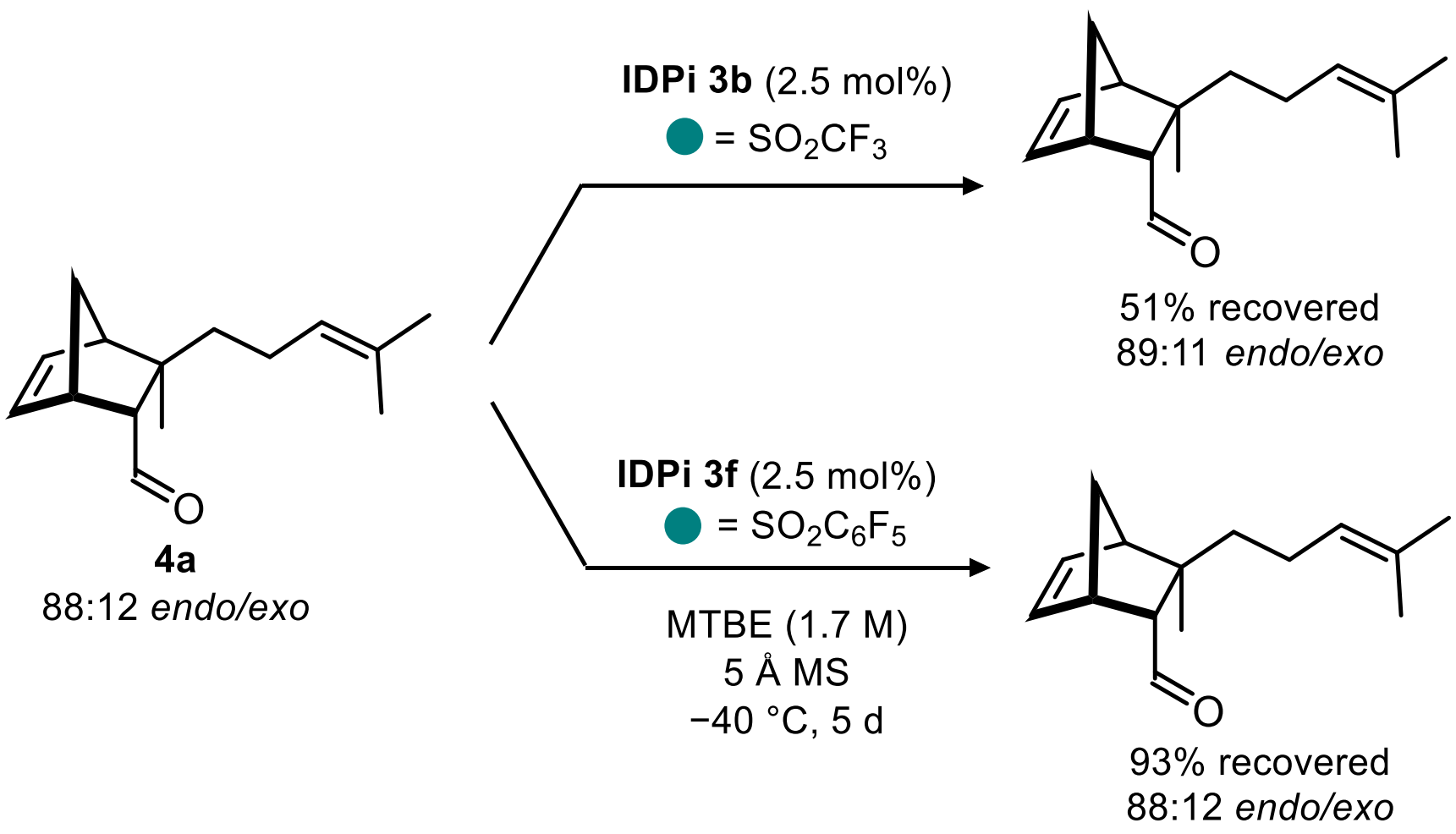

Figure S3. Stability of **4a** in the presence of IDPi catalysts

Test samples: Activated (130 °C, 2 d, high vacuum) molecular sieves 5 Å (17.0 mg) and corresponding IDPi catalyst (**3b** or **3f**, 0.025 equiv., 2.5 mol%) were added in a flame-dried vial under Ar. MTBE (1.7 M, 30 µL) was added and the obtained mixture was stirred for 15 min at rt. Then the reaction vessel was put in dry ice bath and after 5 min Diels–Alder product **4a** was added (1.0 equiv., 0.05 mmol, 11.0 mg) followed by addition of CH_2Br_2 (0.05 mmol, 3.5 µL) and obtained mixture was then kept stirring in −40 °C cryostat for 5 d.

Comparison sample: Activated (130 °C, 2 d, high vacuum) molecular sieves 5 Å (17.0 mg) were added in a flame-dried vial under Ar. MTBE (1.7 M, 30 µL) was added and the obtained mixture was stirred for 15 min at rt. Then Diels–Alder product **4a** was added (1.0 equiv., 0.05 mmol, 11.0 mg) followed by addition of CH_2Br_2 (0.05 mmol, 3.5 µL). After stirring the mixture for 15 min,

molecular sieves were removed by filtration through syringe filter and the reaction mixture was transferred to NMR tube diluting with 300 µL of DCM-*d2*. The yield after filtration was found to be 93%.

After completion of the reaction, it was quenched by the addition of TEA (0.03 equiv.) and allowed to warm up to rt. Molecular sieves were removed by filtration through syringe filter and the reaction mixture was transferred to NMR tube diluting with 300 µL of DCM- *d2*. Recovered yields were determined in relation to the internal standard and accounting the loss after filtration determined from the comparison sample.

*Side reactivity of geranial **1a***

Several side products were found to accompany the reaction, mostly presented by polymers of enals. In case of geranial **1a** we were able to identify the main side-product arises from the condensation between **1a-(*E*)** and **1a-(*Z*)**. We attempted to suppress this process by performing the reaction at higher dilutions and via slow addition of the enal using syringe pump, however, as can be seen from the optimization tables, neither of these approaches lead to improved yield of the target Diels–Alder product.

(*E*)-2-(2,6-dimethylhepta-1,5-dien-1-yl)-7-methyl-4-methylene-3,4,4a,5,6,8a-hexahydro-2H-chromene (4a-S)

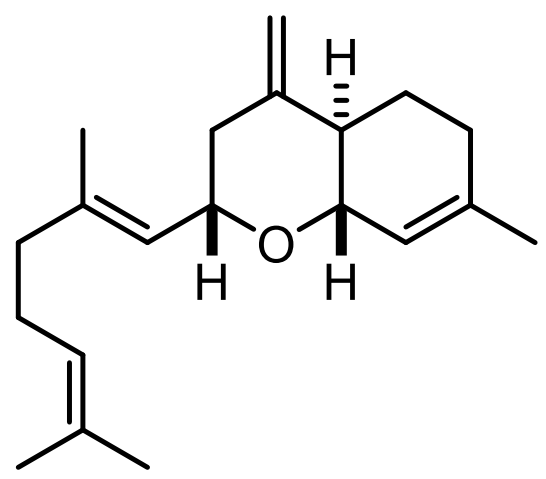

^{1}H NMR (600 MHz, $CDCl_3$) δ 5.44 (dq, *J* = 2.7, 1.4 Hz, 1H), 5.26 (dq, *J* = 8.1, 1.3 Hz, 1H), 5.10 (thept, *J* = 7.0, 1.4 Hz, 1H), 4.77 (q, *J* = 1.6 Hz, 1H), 4.64 (q, *J* = 1.6 Hz, 1H), 4.19 (ddd, *J* = 11.0, 8.1, 3.0 Hz, 1H), 3.61 (dhept, *J* = 9.4, 3.7 Hz, 1H), 2.24 (dd, *J* = 13.4, 3.0 Hz, 1H), 2.18 (ddt, *J* = 13.4, 11.0, 1.8 Hz, 1H), 2.15 – 2.06 (m, 3H), 2.04–1.99 (m, 3H), 1.99–1.91 (m, 2H), 1.69–1.67 (m, 9H), 1.60–1.59 (m, 3H), 1.52–1.43 (m, 1H).

^{13}C NMR (151 MHz, $CDCl_3$) δ 148.3, 138.9, 137.0, 131.8, 125.5, 124.2, 123.8, 105.1, 80.0, 76.4, 44.3, 42.7, 39.6, 30.6, 26.5, 25.8, 23.1, 22.6, 17.8, 17.1.

HRMS *m/z* (GC-EI): calcd. for $C_{20}H_{30}O$ ($[M]^+$): 286.229665; found 286.229673.

3. Substrates Synthesis

3.1. Synthesis via Horner–Wadsworth–Emmons reaction (GP1)

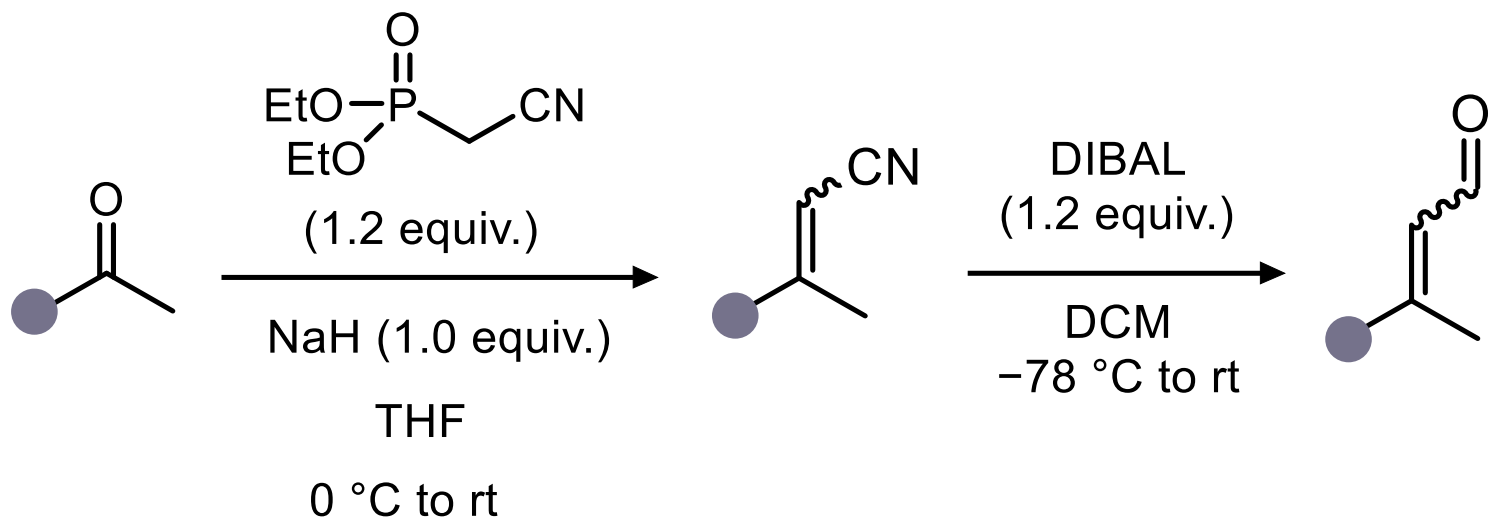

The procedure was adopted from Dong et al.[9]

In a two-neck round-bottom flask under Ar diethyl cyanomethylphosphonate (1.2 equiv.) was dissolved in dry tetrahydrofuran (0.6 M) and cooled down to 0 °C. Sodium hydride (60% in mineral oil, 1 equiv.) was slowly added, followed by addition of a solution of ketone (1 equiv.) in small amount of dry tetrahydrofuran. The resulting solution was kept stirring at rt for 6 h. After completion the reaction was quenched with water and extracted with DCM (3x). The combined organic phase was washed with water (3x), dried over anhydrous Na_2SO_4 and concentrated in vacuo.

The obtained residue was dissolved in DCM (1.0 M) and diisobutylaluminum hydride (DIBAL, 1.0 M in hexanes, 1.2 equiv.) was added dropwise at −78 °C under Ar atmosphere. The reaction mixture was kept at −20 °C for 2 h and monitored by TLC. After reaction was completed, EA (10 mL) was added to quench the remaining DIBAL. The reaction mixture was warmed up to rt followed by addition of another 10 mL of EA and 20 mL of saturated potassium sodium tartrate aqueous solution. The suspension was stirred vigorously for 4 h to give a biphasic mixture, which was separated and aqueous layer was extracted with EA (3x). The organic layers were combined, dried over Na_2SO_4 and then concentrated in vacuo. The residue was purified by automated silica gel column chromatography (MTBE:*n*-pentane from 0% to 25%) to give the corresponding alk-2-enals **1**.

3-Methylhept-2-enal (1c)

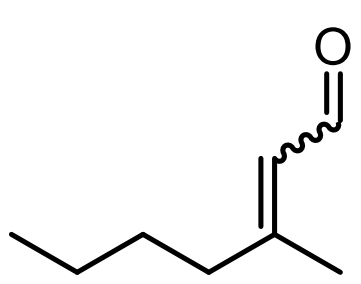

Obtained as a mixture of *E/Z* isomers (*E/Z* ≈ 75:25), **1c-(*E*)** was separated using automated silica gel column chromatography (MTBE:*n*-pentane from 0% to 13%). Light yellow oil, 62.4% yield. The NMR spectroscopic data are reported for **1c-(*E*)** and are in agreement with the literature.[10]

^{1}H NMR (501 MHz, CD_2Cl_2) δ 9.96 (d, *J* = 8.1 Hz, 1H), 5.81 (dq, *J* = 8.1, 1.3 Hz, 1H), 2.30–2.17 (m, 2H), 2.14 (d, *J* = 1.3 Hz, 3H), 1.53–1.43 (m, 2H), 1.39–1.29 (m, 2H), 0.91 (t, *J* = 7.3 Hz, 3H).

3,7-Dimethyloct-2-enal (1f)

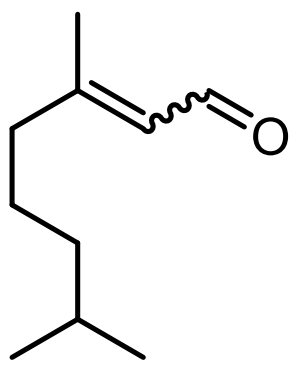

Obtained as a mixture of *E/Z* isomers (*E/Z* ≈ 95:5) as a light yellow oil, 43.8% yield. The NMR spectroscopic data are reported for **1f-(*E*)** in agreement with the literature.[11]

^{1}H NMR (501 MHz, $CDCl_3$) δ 10.02 (d, *J* = 8.1 Hz, 1H), 5.90 (dp, *J* = 8.2, 1.3 Hz, 1H), 2.22–2.17 (m, 2H), 2.16 (d, *J* = 1.4 Hz, 3H), 1.62–1.48 (m, 1H+2H), 1.28–1.15 (m, 2H), 0.88 (d, *J* = 6.6 Hz, 6H).

3-Methyl-5-phenylpent-2-enal (1h)

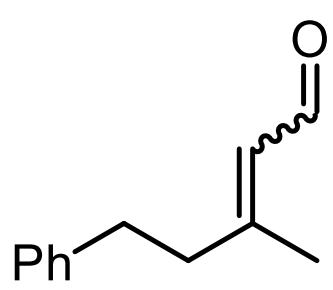

Obtained as a mixture of *E/Z* isomers (*E/Z* ≈ 65:35), **1h-(*E*)** was separated using automated silica gel column chromatography (MTBE:*n*-pentane from 0% to 12%). Light yellow oil, 55.0% yield. The NMR spectroscopic data reported for **1h-(*E*)** and are in agreement with the literature.[12]

^{1}H NMR (501 MHz, $CDCl_3$) δ 9.99 (d, *J* = 8.0 Hz, 1H), 7.24–7.15 (m, 5H), 5.90 (dq, *J* = 8.0, 1.3 Hz, 1H), 2.85–2.74 (m, 2H), 2.56–2.50 (m, 2H), 2.19 (d, *J* = 1.4 Hz, 3H).

6-(Benzyloxy)-3-methylhex-2-enal (1i)

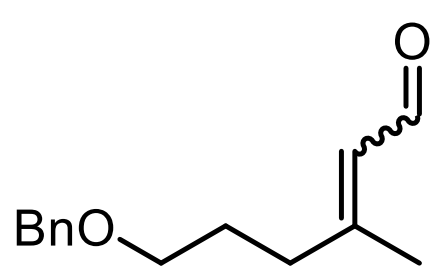

Obtained as a mixture of *E/Z* isomers (*E/Z* ≈ 91:9) as a light yellow oil (961 mg, 4.41 mmol, 44.1% yield). ^{13}C shifts of the major isomer are reported.

^{1}H NMR (501 MHz, CD_2Cl_2) δ 9.98 (d, *J* = 8.0 Hz, $1H_{maj}$), 9.95 (d, *J* = 8.2 Hz, $1H_{min}$), 7.45–7.23 (m, $5H_{maj+min}$), 5.85 (ddq, *J* = 8.0, 2.5, 1.3 Hz, $1H_{maj+min}$), 4.48 (s, $2H_{maj+min}$), 3.49 (t, *J* = 6.2 Hz, $2H_{maj+min}$), 2.72–2.64 (m, $2H_{min}$), 2.40–2.27 (m, $2H_{maj}$), 2.16 (d, *J* = 1.2 Hz, $3H_{maj}$), 1.98 (d, *J* = 1.3 Hz, $3H_{min}$), 1.87–1.78 (m, $2H_{maj+min}$).

^{13}C NMR (126 MHz, CD_2Cl_2) δ 191.4, 164.1, 139.1, 128.7, 128.0, 127.9, 127.6, 73.2, 69.8, 37.6, 27. 8, 17.7.

HRMS *m/z* (API-MS): calcd. for $C_{14}H_{18}O_2Na$ ($[M+Na]^+$): 241.11971; found 241.11990.

4-Chloro-3-methylbut-2-enal (1k)

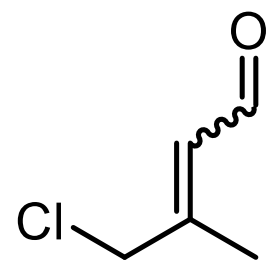

From *chloroacetone* and *triethyl phosphonoacetate* via Horner–Wadsworth–Emmons reaction and reduction with $LiAlH_4$ as described by Matsuda et al.[13] The obtained allylic alcohol was oxidized as described in GP2 yielding the product as mixture of *E/Z* isomers (*E/Z* ≈ 76:24), **1k-(*E*)** was separated using automated silica gel column chromatography (MTBE:*n*-pentane from 0% to 6%), obtaining 327 mg (2.76 mmol, 27.6%) of yellow oil. Light and temperature sensitive compound, was stored at −78 °C. The NMR spectroscopic data reported for **1k-(*E*)** and are in agreement with the literature.[14]

^{1}H NMR (501 MHz, CD_2Cl_2) δ 10.02 (d, *J* = 7.6 Hz, 1H), 6.07 (dq, *J* = 7.6, 1.2 Hz, 1H), 4.13 (d, *J* = 1.0 Hz, 2H), 2.24 (d, *J* = 1.3 Hz, 3H).

3.2. Synthesis via Oxidation of Alcohols (GP2)

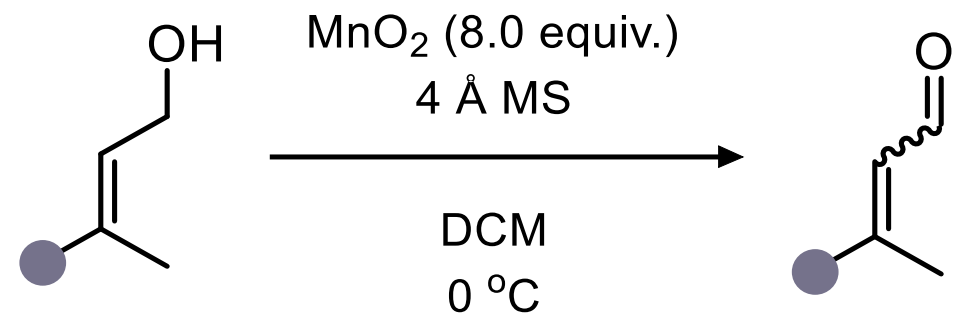

The procedure was adopted from Breinbauer et al.[14]

In a two-neck flame dried round-bottom flask under Ar 1.0 g of activated 4 Å MS was transferred, followed by addition of allylic alcohol substrate (1.0 equiv.) in DCM (0.1 M). Activated MnO_2 (8.0 equiv.) was added to the obtained mixture. The black reaction mixture was stirred for 8 h or until full consumption of starting alcohol was achieved (monitored by TLC). MnO_2 was removed by filtration through a pad of SiO_2 washing with DCM. The filtrate was collected and the solvent was evaporated under reduced pressure. The residue was purified by automated silica gel column chromatography (MTBE:*n*-pentane from 0% to 15%) to give the corresponding alk-2-enals **1**.

(6*E*)-3,7,11-Trimethyldodeca-2,6,10-trienal (farnesal) (1d)

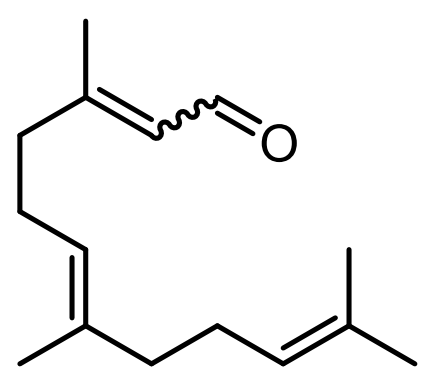

Obtained as a mixture of *E/Z* isomers (*E/Z* ≈ 75:25), **1d-(*E*)** was separated using automated silica gel column chromatography (MTBE:*n*-pentane from 0% to 17%) as a light yellow oil, 75.5% yield. The NMR spectroscopic data reported for **1d-(*E*)** and are in agreement with the literature.[15]

^{1}H NMR (501 MHz, CD_2Cl_2) δ 9.96 (d, J = 8.1 Hz, 1H), 5.82 (dq, J = 8.0, 1.2 Hz, 1H), 5.14–5.05 (m, 2H), 2.28–2.16 (m, 4H), 2.14 (d, J = 1.3 Hz, 3H), 2.10–2.02 (m, 2H), 2.01–1.95 (m, 2H), 1.66 (d, J = 1.5 Hz, 3H), 1.62–1.57 (m, 6H).

(6*E*,10*E*)-3,7,11,15-tetramethylhexadeca-2,6,10,14-tetraenal (1e)

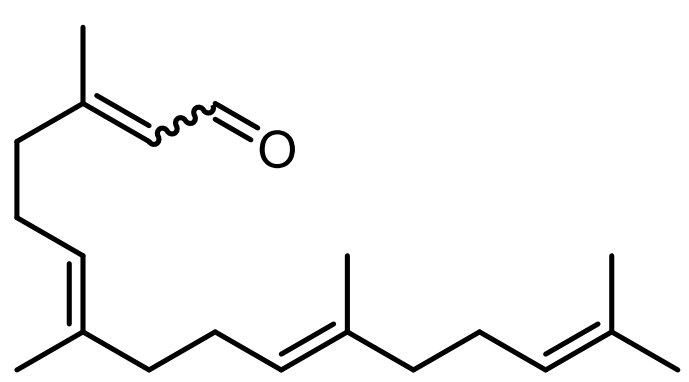

Obtained as a mixture of *E/Z* isomers (*E/Z* ≈ 96:4) as a light yellow oil, 92.0% yield. The NMR spectroscopic data are reported for **1e-(*E*)** and are in agreement with the literature.[15]

^{1}H NMR (501 MHz, $CDCl_3$) δ 9.99 (d, J = 8.0 Hz, 1H), 5.91–5.86 (m, 1H), 5.13–5.05 (m, 3H), 2.26–2.18 (m, 4H), 2.17 (d, J = 1.3 Hz, 3H), 2.11–2.01 (m, 4H), 2.02–1.93 (m, 4H), 1.68 (d, J = 1.6 Hz, 3H), 1.63–1.57 (m, 9H).

2-Methyl-4-oxobut-2-en-1-yl acetate (1j)

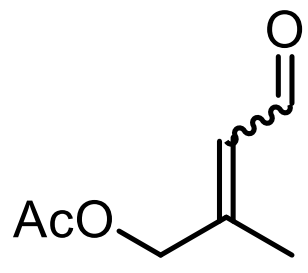

Corresponding allylic alcohol obtained from prenol (3-methyl-2-buten-1-ol) as described by Barrero et al.[16] The oxidation product is obtained according to GP2 as mixture of *E/Z* isomers (*E/Z* ≈ 95:5) as a light yellow oil, 39.2% yield. The NMR spectroscopic data are reported for **1j-(*E*)** and in agreement with the literature.[17]

^{1}H NMR (501 MHz, CD_2Cl_2) δ 10.07 (d, *J* = 7.9 Hz, 1H), 6.06–5.99 (m, 1H), 4.69–4.64 (m, 2H), 2.18 (dt, *J* = 1.5, 0.8 Hz, 3H), 2.15 (s, 3H).

4. Organocatalytic Diels−Alder Reaction of β,β-Disubstituted Enals (GP3)

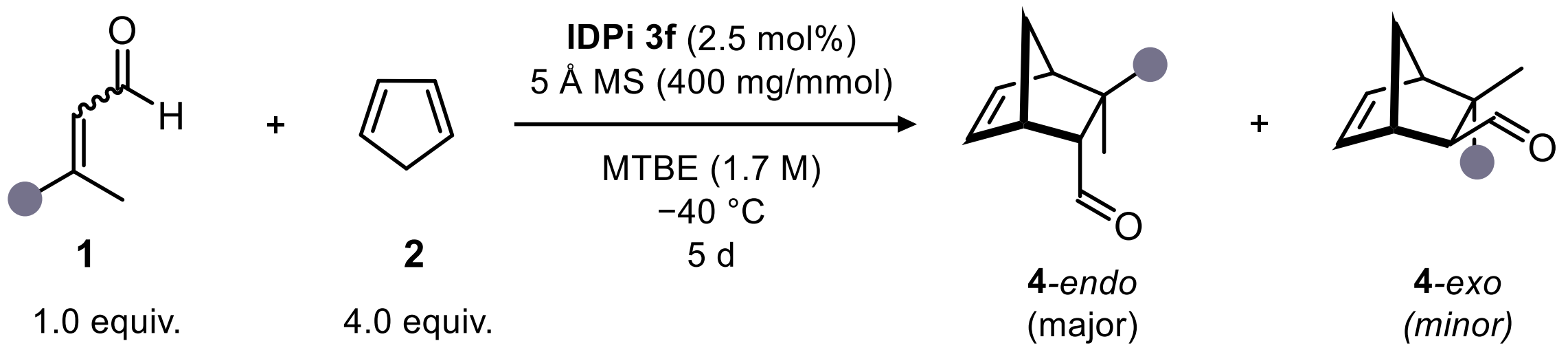

Activated (130 °C, 2 d, high vacuum) molecular sieves 5 Å (80.0 mg), and IDPi catalyst **3f** (0.025 equiv., 2.5 mol%, 0.005 mmol, 8.3 mg) were introduced in a flame-dried glass vial under Ar. MTBE (1.7 M, 120 μL) was added, and the resulting suspension was stirred at rt for 15 min. The reaction mixture was then cooled in a dry ice bath, followed by the addition of cyclopentadiene **2** (4.0 equiv., 0.8 mmol, 66.0 μL). After stirring for 10 min in the dry ice bath, the enal substrate **1** (1.0 equiv., 0.2 mmol) was added. The mixture was then transferred to a cryostat maintained at −40 °C and stirred for 5 d, unless otherwise specified.

Upon completion of the reaction, the mixture was quenched with triethylamine (0.03 equiv.) and allowed to warm to rt. The molecular sieves were removed by filtration through a short pad of Celite®, washed with MTBE, and the solvent was removed under reduced pressure. The crude product was purified by column chromatography using *n*-pentane/MTBE (9:1) as eluent, unless otherwise noted.

Racemate Synthesis

The racemic samples were synthesized as described in GP3 using 1:1 *S,S:R,R* enantiomers catalyst mixture on 0.05 mmol scale. The racemic products were isolated using thin-layer preparative chromatography.

Influence of Enal Double-Bond Geometry on the Reaction Outcome

To avoid the formation of complex isomeric mixtures of cycloadducts, we focused on the preparation of enals with a defined double-bond geometry, typically the (*E*)-isomer. During scale-up experiments using citral mixture (see Chapter 7.2), we found that the (*Z*)-isomer (neral) did not participate in the target Diels–Alder reaction, most likely due to its propensity for intramolecular cyclization. Other (*Z*)-configured substrates also proved difficult to engage in the target Diels–Alder reaction. Typically, the (*Z*)-isomers remained detectable until the end of the reaction despite the apparent consumption of their (*E*)-counterparts, as exemplified by the reaction between *E*/*Z*-mixture of **1g** and diene **2** (Figure S4, representing NMR spectra of aliquots taken at indicated time periods from the reaction which was set up according to GP3 in the presence of the internal standard (CH_2Br_2)). We speculate that the (*Z*)-configuration, which positions C–H-bond containing

substituents in close proximity to the carbonyl group, hinders coordination of the catalyst to the carbonyl group within the confined catalytic pocket.

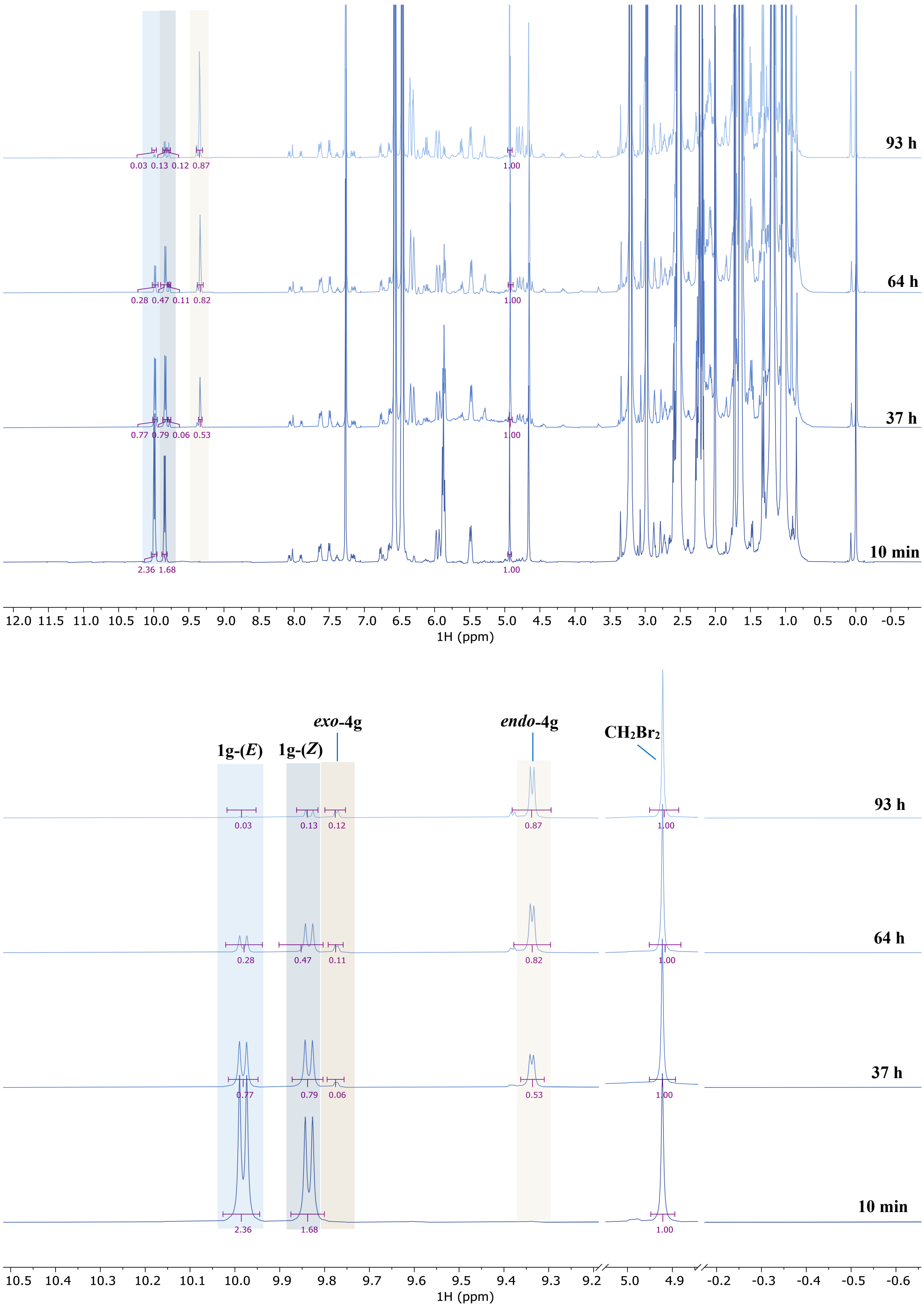

Figure S4. Diels–Alder reaction of *E*/*Z*-mixture of **1g**.

This observation is further supported by the fact that β,β-disubstituted enals bearing substituents larger than methyl on both sides do not participate in the target Diels–Alder reaction (see Figure S5).

4.1. Substrate Scope

(1*S*,2*R*,3*R*,4*R*)-3-Methyl-3-(4-methylpent-3-en-1-yl)bicyclo[2.2.1]hept-5-ene-2-carbaldehyde (4a)

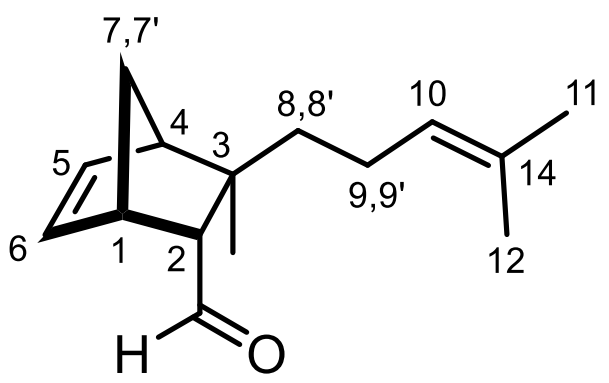

Following GP3 with geranial as enal. The product obtained as mixture of isomers *endo*/*exo* = 88:12. Yield 62.9%, 27.5 mg, 0.126 mmol, light yellow oil.

^{1}H NMR (501 MHz, $CDCl_3$) δ 9.77 (d, *J* = 3.2 Hz, 1H-CHO_{min}), 9.34 (d, *J* = 4.0 Hz, 1H-CHO_{maj}), 6.34 (dd, *J* = 5.7, 2.9 Hz, 1H-6_{maj}), 6.29 (dd, *J* = 5.7, 3.1 Hz, 1H-5_{maj}), 6.21 (dd, *J* = 5.7, 2.9 Hz, 1H-6_{min}), 6.14 (dd, *J* = 5.7, 3.1 Hz, 1H-5_{min}), 5.14–5.07 (m, 1H-10_{maj}), 5.09–5.02 (m, 1H-10_{min}), 3.07–2.91 (m, 1H-1_{maj}), 2.60–2.53 (m, 1H-4_{maj}), 2.52–2.49 (m, 1H-2_{maj}), 2.48–2.46 (m, 1H-2_{min}), 2.12–2.02 (m, 2H-$9,9‘_{maj}$), 2.02–1.94 (m, 2H-$9,9‘_{min}$), 1.73–1.66 (m, 5H, $11_{maj+min}$, $7_{maj+min}$, $8_{maj+min}$), 1.64–1.59 (m, 4H, $12_{maj+min}$, $8‘_{maj+min}$), 1.50–1.47 (m, 1H, $7‘_{maj+min}$), 0.91 (s, 3H-13_{maj}+13_{min}).

^{13}C NMR (126 MHz, $CDCl_3$) δ 206.8 (CHO_{maj}), 206.1(CHO_{min}), 138.3 (5_{maj}), 137.4 (5_{min}), 135.4 (6_{min}), 134.1 (6_{maj}), 131.7 (14_{maj}), 131.5 (14_{min}), 124.3 ($10_{maj+min}$), 61.7 (2_{maj}), 60.1 (2_{min}), 52.7 (4_{maj}), 52.6 (4_{min}), 48.8 ($3_{maj+min}$), 47.2 ($7,7‘_{maj}$), 46.0 ($7,7‘_{min}$), 45.9 (1_{maj}), 44.4 ($8,8‘_{maj}$), 44.2 (1_{min}), 42.6 ($8,8‘_{min}$), 27.0 (11_{min}), 25.7 (11_{maj}), 24.0 ($9,9‘_{min}$), 23.7 ($9,9‘_{maj}$), 22.4 (13_{min}), 20.5 (13_{maj}), 17.7 (12_{maj}), 17.6 (12_{min}).

HRMS *m/z* (GC-EI): calcd. for $C_{15}H_{22}O$ ($[M]^+$): 218.166515; found 218.166530.

GC (29.5 m BGB-178, injection temperature: 220 °C; 100 °C – iso 5 min, 1 °C/min, 230 °C iso 10 min, 0.5 bar H_2)

t_{R1} = 88.41 min (minor), t_{R2} = 91.15 min (minor), t_{R3} = 97.38 min (major), t_{R4} = 99.10 min (major); e.r. (minor) = 93:7 (86% e.e.), e.r. (major) = 99:1 (98% e.e.)

$[\boldsymbol{\alpha}]_{\boldsymbol{D}}^{\mathbf{25}} = -66.55$ (*c* = 0.565, $CHCl_3$)

(1*S*,2*R*,4*R*)-3,3-dimethylbicyclo[2.2.1]hept-5-ene-2-carbaldehyde (4b)

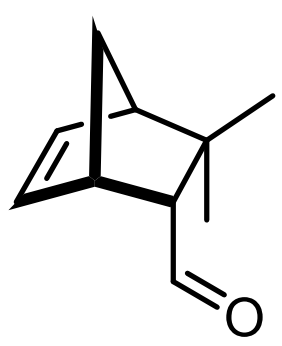

Following GP3 with prenal as enal. The product obtained as mixture of isomers *endo*/*exo* = 88:12. Yield 57.0%, 17.1 mg, 0.114 mmol, colourless oil.

^{1}H NMR (501 MHz, CD_2Cl_2) δ 9.77 (d, *J* = 3.4 Hz, $1H_{min}$), 9.33 (d, *J* = 4.2 Hz, $1H_{maj}$), 6.33–6.29 (m, $2H_{maj+min}$), 6.24 (dd, *J* = 5.7, 3.1 Hz, $1H_{min}$), 6.16 (dd, *J* = 5.7, 3.1 Hz, $1H_{min}$) 3.01–2.96 (m, $1H_{maj}$), 2.98–2.94 (m, $1H_{min}$), 2.48–2.43 (m, $1H_{maj}$), 2.43 (dd, *J* = 4.2, 3.3 Hz, $1H_{maj}$), 1.74–1.68 (m, $1H_{min+maj}$), 1.49–1.47 (m, $1H_{maj+min}$), 1.30 (s, $3H_{maj}$), 1.17 (s, $3H_{min}$), 1.03 (s, $3H_{min}$), 0.91 (s, $3H_{maj}$).

^{13}C NMR (126 MHz, CD_2Cl_2) δ 206.4_{maj}, 205.7_{min}, 138.3_{maj}, 137.8_{min}, 135.2_{min}, 133.6_{maj}, 62.8_{maj}, 60.6_{min}, 54.4_{maj}, 54.0_{min}, $47.6_{maj+min}$, 46.6_{min}, 46.3_{maj}, 45.3_{maj}, 45.1_{min}, 32.0_{maj}, 29.9_{min}, 26.1_{min}, 23.9_{maj}.

HRMS *m/z* (GC-EI): calcd. for $C_{10}H_{14}O$ ($[M]^+$): 150.103915; found 150.103890.

GC (29.5 m BGB-178, injection temperature: 220 °C; 70 °C – iso 5 min, 1 °C/min, 110 °C; 8 °C/min, 220 °C iso 10 min, 0.5 bar H_2)

t_{R1} = 20.58 min (minor), t_{R2} = 20.97 min (minor), t_{R3} = 22.43 min (major), t_{R4} = 22.91 min (major); e.r. (minor) = 59:41 (18% e.e.), e.r. (major) = 96:4 (92% e.e.)

$[\alpha]_D^{25} = -19.13$ (*c* = 0.486, $CHCl_3$)

(1*S*,2*R*,3*R*,4*R*)-3-butyl-3-methylbicyclo[2.2.1]hept-5-ene-2-carbaldehyde (4c)

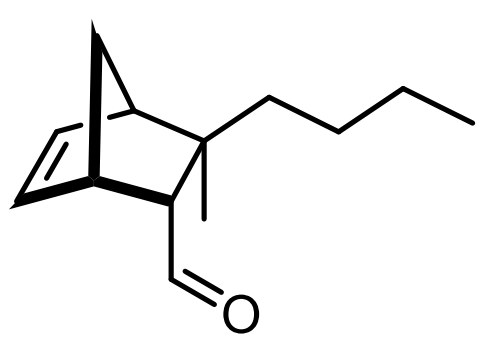

Following GP3 with 3-methylhept-2-enal as enal. The product obtained as mixture of isomers *endo*/*exo* = 94:6. Yield 65.6%, 24.9 mg, 0.130 mmol, colourless oil.

^{1}H NMR (501 MHz, CD_2Cl_2) δ 9.73 (d, *J* = 3.2 Hz, $1H_{min}$), 9.30 (d, *J* = 4.0 Hz, $1H_{maj}$), 6.31 (dd, *J* = 5.7, 2.9 Hz, $1H_{maj}$), 6.28 (dd, *J* = 5.7, 3.2 Hz, $1H_{maj}$), 6.19 (dd, *J* = 5.7, 3.0 Hz, $1H_{min}$), 6.12 (dd, *J* = 5.7, 3.1 Hz, $1H_{min}$), 2.98–2.93 (m, $1H_{maj}$), 2.59–2.57 (m, $1H_{min}$), 2.55–2.47 (m, $1H_{maj}$), 2.47–2.42 (m, $1H_{maj}$), 2.42–2.40 (m, $1H_{min}$), 1.69 (dt, *J* = 8.7, 1.7 Hz, $1H_{maj}$), 1.51–1.42 (m, $2H_{maj+min}$), 1.37–1.23 (m, $5H_{maj+min}$), 0.91 (t, *J* = 7.1 Hz, $3H_{maj+min}$), 0.85 (s, $3H_{maj+min}$).

^{13}C NMR (126 MHz, CD_2Cl_2, major isomer is reported) δ 206.5, 138.2, 134.1, 61.6, 48.7, 47.0, 45.9, 44.1, 29.7, 27.1, 23.5, 20.3, 13.8.

HRMS *m/z* (GC-EI): calcd. for $C_{13}H_{24}N_1O$ ($[M+NH_4]^+$): 210.185239; found 210.185170.

GC (29.5 m BGB-178, injection temperature: 220 °C; 100 °C–iso 5 min, 2 °C/min, 180 °C; 5 °C/min, 220 °C iso 10 min, 0.5 bar H_2)

t_{R1} = 17.35 min (minor), t_{R2} = 17.74 min (minor), t_{R3} = 18.35 min (major), t_{R4} = 18.83 min (major); e.r. (minor) = 51:49 (2% e.e.), e.r. (major) = 98:2 (96% e.e.)

$[\alpha]_D^{25} = -16.98$ (*c* = 0.502, $CHCl_3$)

(1*S*,2*R*,3*R*,4*R*)-3-((*E*)-4,8-dimethylnona-3,7-dien-1-yl)-3-methylbicyclo[2.2.1]hept-5-ene-2-carbaldehyde (4d)

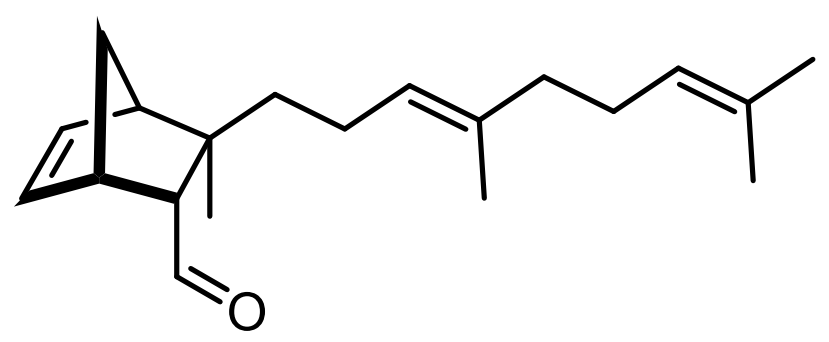

Following GP3 with (6*E*)-3,7,11-trimethyldodeca-2,6,10-trienal (farnesal) as enal. The product obtained as mixture of isomers *endo*/*exo* = 88:12. Yield 63.2%, 36.1 mg, 0.126 mmol, light yellow oil.

^{1}H NMR (501 MHz, CD_2Cl_2) δ 9.76 (d, *J* = 3.1 Hz, $1H_{min}$), 9.33 (d, *J* = 3.9 Hz, $1H_{maj}$), 6.34 (dd, *J* = 5.7, 2.9 Hz, $1H_{maj}$), 6.29 (dd, *J* = 5.7, 3.2 Hz, $1H_{maj}$), 6.22 (dd, *J* = 5.7, 3.0 Hz, $1H_{min}$), 6.15 (dd, *J* = 5.7, 3.0 Hz, $1H_{min}$), 5.14 (tq, *J* = 7.1, 1.4 Hz, $1H_{maj+min}$), 5.10 (tdt, *J* = 5.7, 2.9, 1.4 Hz, $1H_{maj+min}$), 3.00–2.95 (m, $1H_{maj+min}$), 2.58–2.55 (m, $1H_{maj+min}$), 2.51–2.47 (t, *J* = 3.63 Hz, $1H_{maj+min}$), 2.12–2.03 (m, $4H_{maj+min}$), 2.00–1.95 (m, $2H_{maj+min}$), 1.73–1.70 (m, $1H_{maj+min}$), 1.69–1.66 (m, $3H_{maj+min}$), 1.63–1.61 (m, $3H_{maj+min}$), 1.61–1.58 (m, $3H_{maj+min}$+$1H_{maj+min}$), 1.56–1.50 (m, $1H_{maj+min}$), 1.48–1.44 (m, $1H_{maj+min}$), 1.16 (s, $3H_{min}$), 0.90 (s, $3H_{maj}$).

^{13}C NMR (126 MHz, CD_2Cl_2) δ 206.7_{maj}, 206.2_{min}, 138.6_{maj}, 137.7_{min}, 135.8_{min}, 135.5_{maj}, 135.3_{min}, 134.6_{maj}, $131.7_{maj+min}$, 124.8_{maj}, 124.75_{min}, 124.71_{maj}, 124.68_{min}, 62.1_{maj}, 60.5_{min}, 53.2_{maj}, 53.0_{min}, $49.1_{maj+min}$, $47.5_{maj+min}$, 46.3_{maj}, 44.8_{maj}, 44.6_{min}, 43.0_{min}, $40.1_{maj+min}$, 27.12_{maj}, 27.08_{min}, $25.8_{maj+min}$, 24.3_{min}, 24.0_{maj}, 22.6_{min}, 20.7_{maj}, $17.8_{maj+min}$, $16.1_{maj+min}$.

HRMS *m/z* (GC-EI): calcd. for $C_{20}H_{30}O_1Na_1$ ($[M+Na]^+$): 309.21889; found 309.21885.

HPLC (Chiralpak IB-N3, 100% *n*-heptane, 298 K, 220 nm)

t_{R1} = 7.53 min (minor), t_{R2} = 8.04 min (major), t_{R3} = 12.40 min (minor), t_{R4} = 14.77 min (major); e.r. (minor) = 99:1 (98% e.e.), e.r. (major) = 99:1 (98% e.e.)

$[\alpha]_D^{25}$ = −67.59 (*c* = 0.505, $CHCl_3$)

(1*S*,2*R*,3*R*,4*R*)-3-methyl-3-((3*E*,7*E*)-4,8,12-trimethyltrideca-3,7,11-trien-1-yl)bicyclo[2.2.1]hept-5-ene-2-carbaldehyde (4e)

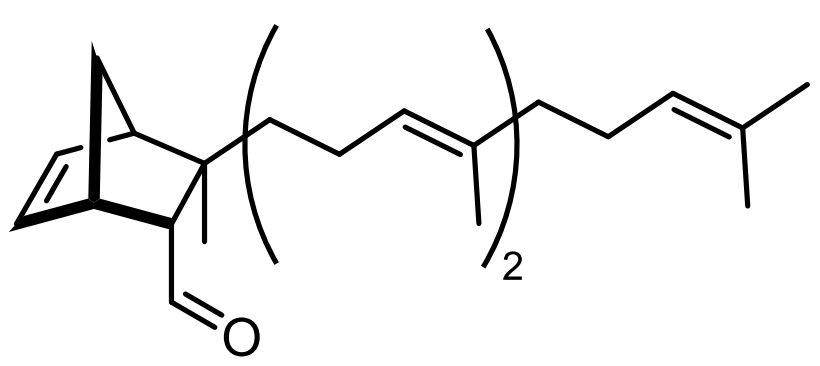

Following GP3 with (6*E*,10*E*)-3,7,11,15-tetramethylhexadeca-2,6,10,14-tetraenal as enal. The product obtained as mixture of isomers *endo*/*exo* = 88:12. Yield 60.0%, 42.5 mg, 0.120 mmol, light yellow oil.

^{1}H NMR (501 MHz, CD_2Cl_2) δ 9.75 (d, *J* = 3.2 Hz, $1H_{min}$), 9.32 (d, *J* = 3.9 Hz, $1H_{maj}$), 6.32 (dd, *J* = 5.7, 2.9 Hz, $1H_{maj}$), 6.28 (dd, *J* = 5.7, 3.1 Hz, $1H_{maj}$), 6.21 (dd, *J* = 5.7, 3.0 Hz, $1H_{min}$), 6.13 (dd, *J* = 5.7, 3.1 Hz, $1H_{min}$), 5.16–5.05 (m, $3H_{maj+min}$), 2.99–2.93 (m, $1H_{maj+min}$), 2.57–2.53 (m, $1H_{maj+min}$), 2.49–2.46 (m, $1H_{maj+min}$), 2.10–2.01 (m, $6H_{maj+min}$), 2.02–1.93 (m, $5H_{maj+min}$), 1.71–1.68 (m, $1H_{maj+min}$), 1.66 (d, *J* = 1.4 Hz, $3H_{maj+min}$), 1.62 (d, *J* = 1.3 Hz, $3H_{maj+min}$), 1.60–1.57 (m, $7H_{maj+min}$), 1.46–1.42 (m, $1H_{maj+min}$), 1.15 (s, $3H_{min}$), 0.89 (s, $3H_{maj}$).

^{13}C NMR (126 MHz, CD_2Cl_2) δ 206.7_{maj}, 206.1_{min}, 138.6_{maj}, 137.7_{min}, 135.8_{min}, 135.5_{maj}, 135.4_{maj}, 135.3_{min}, $134.6_{maj+min}$, $131.6_{maj+min}$, $124.8_{maj+min}$, $124.77_{maj+min}$, $124.6_{maj+min}$, 62.1_{maj}, 60.5_{min}, 53.2_{maj}, 53.0_{min}, $49.0_{maj+min}$, $47.5_{maj+min}$, $46.4_{maj+min}$, 44.8_{maj}, 44.6_{min}, $40.2_{maj+min}$, $40.1_{maj+min}$, $27.2_{maj+min}$, 27.0_{maj}, 26.9_{min}, 25.8_{maj}, 24.4_{min}, 24.0_{maj}, 22.6_{min}, $20.7_{maj+min}$, $17.8_{maj+min}$, $16.1_{maj+min}$, $16.09_{maj+min}$.

HRMS *m/z* (GC-EI): calcd. for $C_{25}H_{38}ONa$ ($[M+Na]^+$): 377.28149; found 377.28159.
HPLC (Chiralpak IB-N3, 100% *n*-heptane, 298 K, 220 nm)

t_{R1} = 7.52 min (minor), t_{R2} = 8.08 min (major), t_{R3} = 11.34 min (minor), t_{R4} = 13.40 min (major); e.r. (minor) = 99:1 (98% e.e.), e.r. (major) = 98:2 (96% e.e.)

$[\alpha]_D^{25} = -71.05$ (*c* = 0.485, $CHCl_3$)

(1*S*,2*R*,3*R*,4*R*)-3-methyl-3-(4-methylpentyl)bicyclo[2.2.1]hept-5-ene-2-carbaldehyde (4f)

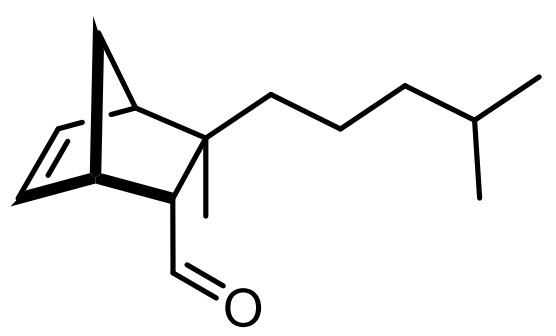

Following GP3 with 3,7-dimethyloct-2-enal as enal. The product obtained as mixture of isomers *endo*/*exo* = 87:13. Yield 68.0%, 30.0 mg, 0.136 mmol, light yellow oil.

^{1}H NMR (501 MHz, CD_2Cl_2) δ 9.74 (d, *J* = 3.2 Hz, $1H_{min}$), 9.31 (d, *J* = 4.0 Hz, $1H_{maj}$), 6.31 (dd, *J* = 5.7, 2.9 Hz, $1H_{maj}$), 6.27 (dd, *J* = 5.7, 3.1 Hz, $1H_{maj}$), 6.19 (dd, *J* = 5.7, 3.0 Hz, $1H_{min}$), 6.16–6.11 (m, $1H_{min}$), 2.97–2.93 (m, $1H_{maj+min}$), 2.55–2.50 (m, $1H_{maj+min}$), 2.47–2.43 (m, $1H_{maj+min}$), 1.71–1.69 (m, $1H_{maj+min}$), 1.68–1.65 (m, $1H_{maj+min}$), 1.57–1.53 (m, $3H_{maj+min}$), 1.47–1.43 (m, $2H_{maj+min}$), 1.40–1.34 (m, $2H_{maj+min}$), 1.19 (s, $3H_{min}$), 1.18 (s, $3H_{min}$), 1.16 (s, $3H_{min}$), 0.88 (s, $3H_{maj}$), 0.87 (s, $3H_{maj}$), 0.86 (s, $3H_{maj}$).

^{13}C NMR (126 MHz, CD_2Cl_2) δ 206.7_{maj}, 206.1_{min}, 138.4_{maj}, 136.0_{min}, 135.9_{min}, 134.2_{maj}, 62.3_{min}, 61.8_{maj}, 52.9_{maj}, 52.2_{min}, 47.9_{min}, 47.2_{maj}, 46.3_{min}, 46.0_{maj}, 44.7_{maj}, 44.5_{min}, 39.9_{maj}, 39.7_{min}, 28.1_{maj}, 27.2_{min}, $23.3_{maj+min}$, $22.8_{maj+min}$, $22.5_{maj+min}$, 22.3_{maj}, 22.2_{min}, 20.6_{min}, 20.5_{maj}.

HRMS *m/z* (GC-EI): calcd. for $C_{15}H_{24}O$ ($[M]^+$): 220.182165; found 220.182190.

GC (29.5 m BGB-178, injection temperature: 220 °C; 100 °C – iso 5 min, 2 °C/min, 180 °C; 5 °C/min, 220 °C iso 10 min, 0.5 bar H_2)

t_{R1} = 70.73 min (minor), t_{R2} = 71.61 min (minor), t_{R3} = 73.93 min (major), t_{R4} = 75.94 min (major); e.r. (minor) = 81:19 (62% e.e.), e.r. (major) = 98:2 (96% e.e.)

$[\boldsymbol{\alpha}]_{\boldsymbol{D}}^{\mathbf{25}} = -$20.67 (*c* = 0.328, $CHCl_3$)

(1*S*,2*R*,3*R*,4*R*)-3-(3,4-dimethylpent-3-en-1-yl)-3-methylbicyclo[2.2.1]hept-5-ene-2-carbaldehyde (4g)

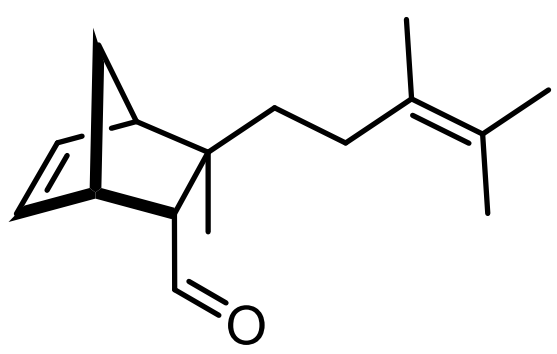

Following GP3 with 3,6,7-trimethylocta-2,6-dienal as enal. The product obtained as mixture of isomers *endo*/*exo* = 87:13. Yield 61.3%, 28.5 mg, 0.122 mmol, light yellow oil.

^{1}H NMR (501 MHz, CD_2Cl_2) δ 9.76 (d, *J* = 3.2 Hz, $1H_{min}$), 9.33 (d, *J* = 3.9 Hz, $1H_{maj}$), 6.34 (dd, *J* = 5.7, 2.9 Hz, $1H_{maj}$), 6.30 (dd, *J* = 5.7, 3.2 Hz, $1H_{maj}$), 6.25 (dd, *J* = 5.7, 3.0 Hz, $1H_{min}$), 6.16 (dd, *J* = 5.7, 3.1 Hz, $1H_{min}$), 3.00–2.95 (m, $1H_{maj+min}$), 2.61–2.54 (m, $1H_{maj+min}$), 2.50–2.47 (m, $1H_{maj+min}$), 2.16–2.02 (m, $2H_{maj+min}$), 1.72 (dt, *J* = 8.8, 1.7 Hz, $1H_{maj+min}$), 1.66–1.62 (m, $11H_{maj+min}$), 1.47 (dt, *J* = 8.8, 1.8 Hz, $1H_{maj+min}$), 1.17 (s, $3H_{min}$), 0.91 (s, $3H_{maj}$).

^{13}C NMR (126 MHz, CD_2Cl_2) δ 206.8_{maj}, 206.1_{min}, 138.6_{maj}, 137.7_{min}, 135.8_{min}, 134.5_{maj}, $128.0_{maj+min}$, 124.1_{maj}, 123.7_{min}, 62.2_{maj}, 60.7_{min}, 53.0_{maj}, 52.8_{min}, $49.1_{maj+min}$, $47.5_{maj+min}$, 46.4_{min}, 46.3_{maj}, 44.6_{min}, 43.0_{maj}, 30.7_{min}, 30.4_{maj}, 22.5_{min}, 20.7_{maj}, 20.62_{min}, 20.57_{maj}, $20.1_{maj+min}$, 18.7_{maj}, 18.6_{min}.

HRMS *m/z* (GC-EI): calcd. for $C_{16}H_{24}O$ ($[M]^+$): 232.182320; found 232.182165.

2D HPLC (1st dimension: PVA-SIL, 100% *n*-heptane, 308 K; 2nd dimension: Chiralcel OD-3, *n*-heptane/2-propanol = 99.5:0.5, 298 K; 210 nm)

t_{R1} = 5.375 min (minor), t_{R2} = 6.110 min (minor), t_{R3} = 5.293 min (minor), t_{R4} = 7.237 min (major); e.r. (minor) = >99.5:0.5 (99% e.e.), e.r. (major) = >99.5:0.5 (99% e.e.)

$[\boldsymbol{\alpha}]_{\boldsymbol{D}}^{\mathbf{25}} = -$44.93 (*c* = 0.552, $CHCl_3$)

(1*S*,2*R*,3*R*,4*R*)-3-methyl-3-phenethylbicyclo[2.2.1]hept-5-ene-2-carbaldehyde (4h)

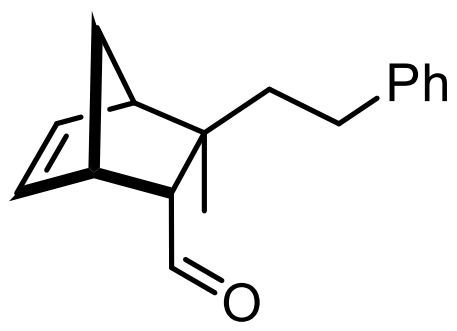

Following GP3 with 3-methyl-5-phenylpent-2-enal as enal. Reaction time – 7 d. The product obtained as mixture of isomers *endo*/*exo* = 88:12. Yield 46.0%, 22.1 mg, 0.092 mmol, light yellow oil.

^{1}H NMR (501 MHz, CD_2Cl_2) δ 9.79 (d, *J* = 3.2 Hz, 1H$_{min}$), 9.37 (d, *J* = 3.9 Hz, 1H$_{maj}$), 7.32–7.17 (m, 5H$_{maj+min}$), 6.37 (dd, *J* = 5.7, 2.9 Hz, 1H$_{maj}$), 6.33 (dd, *J* = 5.7, 3.1 Hz, 1H$_{maj}$), 6.25 (dd, *J* = 5.7, 3.0 Hz, 1H$_{min}$), 6.17 (dd, *J* = 5.7, 3.0 Hz, 1H$_{min}$), 3.04–2.99 (m, 1H$_{maj+min}$), 2.74–2.67 (m, 2H$_{maj+min}$), 2.64–2.60 (m, 1H$_{maj+min}$), 2.58–2.54 (m, 1H$_{maj+min}$), 1.96–1.88 (m, 1H$_{maj+min}$), 1.87–1.77 (m, 1H$_{maj+min}$), 1.74 (dd, *J* = 9.0, 1.7 Hz, 1H$_{maj+min}$), 1.49 (dd, *J* = 8.9, 1.8 Hz, 1H$_{maj+min}$), 1.25 (s, 3H$_{min}$), 0.99 (s, 3H$_{maj}$).

^{13}C NMR (126 MHz, CD_2Cl_2, major isomer is reported) δ 206.23, 142.83, 138.21, 134.22, 128.33, 128.28, 125.67, 61.60, 52.76, 48.84, 47.07, 46.63, 45.93, 31.46, 20.33.

HRMS *m/z* (GC-EI): calcd. for $C_{17}H_{20}Na_1O_1$ ($[M+Na]^+$): 263.14064; found 263.14065.

2D HPLC (1st dimension: PVA-SIL, 100% *n*-heptane, 308 K; 2nd dimension: Chiralcel OD-3, *n*-heptane/2-propanol = 99:1, 298 K; 210 nm)

t_{R1} = 9.967 min (minor), t_{R2} = 12.165 min (minor), t_{R3} = 11.539 min (major), t_{R4} = 15.889 min (major); e.r. (minor) = >99.5:0.5 (99% e.e.), e.r. (major) = >99.5:0.5 (99% e.e.)

$[\boldsymbol{\alpha}]_D^{25}$ = −22.45 (*c* = 0.545, $CHCl_3$)

(1*S*,2*R*,3*R*,4*R*)-3-(3-(benzyloxy)propyl)-3-methylbicyclo[2.2.1]hept-5-ene-2-carbaldehyde (4i)

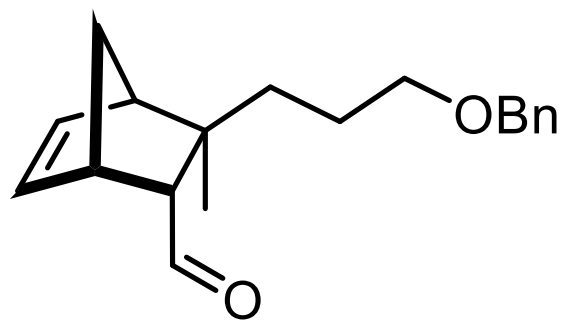

Following GP3 with 6-(benzyloxy)-3-methylhex-2-enal as enal. The product obtained as mixture of isomers *endo*/*exo* = 88:12. Yield 70.0%, 40.0 mg, 0.140 mmol, transparent oil.

^{1}H NMR (501 MHz, CD_2Cl_2) δ 9.74 (d, *J* = 3.2 Hz, 1H$_{min}$), 9.31 (d, *J* = 3.9 Hz, 1H$_{maj}$), 7.35–7.26 (m, 5H$_{maj+min}$), 6.33 (dd, *J* = 5.7, 2.9 Hz, 1H$_{maj}$), 6.29 (dd, *J* = 5.8, 3.1 Hz, 1H$_{maj}$), 6.21 (dd, *J* = 5.7, 3.0 Hz, 1H$_{min}$), 6.14 (dd, *J* = 5.7, 3.1 Hz, 1H$_{min}$), 4.48 (s, 2H$_{maj}$), 4.45 (s, 2H$_{min}$), 3.48 (t, *J* = 6.2 Hz, 2H$_{maj}$), 3.41 (t, *J* = 6.5 Hz, 2H$_{min}$), 2.98–2.95 (m, 1H$_{maj+min}$), 2.55–2.53 (m, 1H$_{maj+min}$), 2.47–2.43 (m, 1H$_{maj+min}$), 1.72–1.65 (m, 5H$_{maj}$), 1.59–1.55 (m, 5H$_{min}$), 1.45 (dt, *J* = 8.8, 1.8 Hz, 1H$_{maj+min}$), 1.13 (s, 3H$_{min}$), 0.87 (s, 3H$_{maj}$).

^{13}C NMR (75 MHz, CD_2Cl_2) δ 206.7$_{maj+min}$, 139.3$_{min}$, 138.5$_{maj}$, 136.1$_{min}$, 134.5$_{maj}$, 130.3$_{maj+min}$, 128.6$_{maj+min}$, 127.9$_{maj+min}$, 127.7$_{maj+min}$, 73.1$_{maj+min}$, 71.3$_{maj}$, 71.2$_{min}$, 61.9$_{maj}$, 60.2$_{min}$, 48.9$_{maj+min}$, 47.4$_{maj+min}$, 46.2$_{maj}$, 44.7$_{min}$, 41.1$_{maj+min}$, 30.0$_{maj+min}$, 26.1$_{min}$, 25.8$_{maj}$, 20.6$_{maj+min}$.

HRMS *m/z* (ESI): calcd. for $C_{19}H_{24}O_2Na_1$ ($[M+Na]^+$): 307.16651; found 307.16685.

2D HPLC (YMC PVA-SIL *n*-heptane/2-propanol = 99.8:0.2, 308 K, 220 nm; Chiralpak IB-N3, *n*-heptane/2-propanol = 99:1, 298 K, 220 nm)

t_{R1} = 4.833 min (minor), t_{R2} = 5.091 min (minor), t_{R3} = 5.151 min (major), t_{R4} = 5.864 min (major); e.r. (minor) = 96:4 (92% e.e.), e.r. (major) = 98.5:1.5 (97% e.e.)

$[\alpha]_D^{25} = -61.40$ (c = 0.589, $CHCl_3$)

((1*R*,2*R*,3*R*,4*S*)-3-formyl-2-methylbicyclo[2.2.1]hept-5-en-2-yl)methyl acetate (4j)

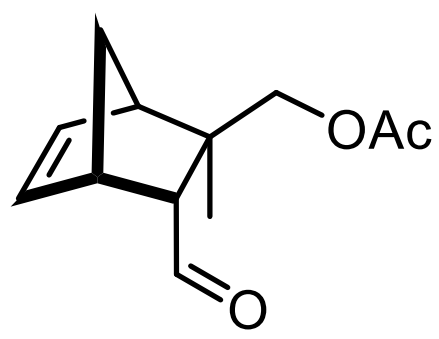

Following GP3 with 2-methyl-4-oxobut-2-en-1-yl acetate as enal. The product obtained as mixture of isomers *endo*/*exo* = 83:17. Yield 50.8%, 21.2 mg, 0.102 mmol, transparent oil.

^{1}H NMR (501 MHz, CD_2Cl_2) δ 9.78 (d, J = 2.6 Hz, $1H_{min}$), 9.40 (d, J = 3.2 Hz, $1H_{maj}$), 6.38 (dd, J = 5.7, 3.0 Hz, $1H_{maj}$), 6.26 (dd, J = 5.7, 3.2 Hz, $1H_{maj}$), 6.20–6.18 (m, $2H_{min}$), 4.14 (d, J = 10.8 Hz, $1H_{maj+min}$), 4.04 (d, J = 10.9 Hz, $1H_{maj+min}$), 3.07–3.02 (m, $1H_{maj+min}$), 2.69–2.65 (m, $1H_{maj+min}$), 2.57–2.52 (m, $1H_{maj+min}$), 2.07 (s, $3H_{maj}$), 2.05 (s, $3H_{min}$), 1.66 (dt, J = 9.1, 1.7 Hz, $1H_{maj+min}$), 1.48 (dt, J = 9.1, 1.8 Hz, $1H_{maj+min}$), 1.21 (s, $3H_{min}$), 0.97 (s, $3H_{maj}$).

^{13}C NMR (75 MHz, CD_2Cl_2) δ 205.0_{maj}, 204.8_{min}, $171.2_{maj+min}$, 137.3_{maj}, 137.0_{min}, 136.6_{min}, 135.7_{maj}, 73.1_{maj}, 72.5_{min}, 59.4_{maj}, 57.0_{min}, 51.5_{min}, 50.1_{maj}, 48.0_{min}, 47.0_{maj}, $46.3_{maj+min}$, 45.9_{maj}, 44.2_{min}, 21.3_{min}, 20.9_{maj}, $19.6_{maj+min}$.

HRMS *m/z* (GC-EI): calcd. for $C_{12}H_{16}O_3Na$ ($[M+Na]^+$): 231.099715; found 231.099721.
GC (24.5 m Ivadex-1, injection temperature: 220 °C; 100 °C–iso 10 min, 1 °C/min, 180 °C – iso 10 min; 5 °C/min, 220 °C – iso 10 min; 0.5 bar H_2)

t_{R1} = 34.30 min (minor), t_{R2} = 34.66 min (minor), t_{R3} = 35.39 min (major), t_{R4} = 36.24 min (major); e.r. (minor) = 66:34 (32% e.e.), e.r. (major) = 97:3 (94% e.e.)

$[\alpha]_D^{25} = -57.70$ (c = 0.310, $CHCl_3$)

(1*S*,2*R*,3*R*,4*R*)-3-(chloromethyl)-3-methylbicyclo[2.2.1]hept-5-ene-2-carbaldehyde (4k)

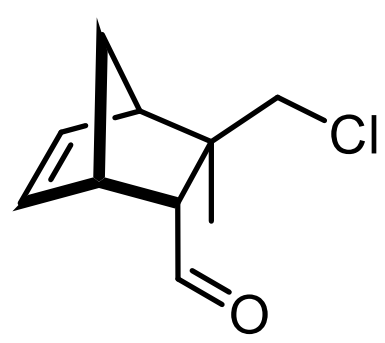

Following GP3 with 4-chloro-3-methylbut-2-enal as enal. Reaction performed at −60 °C. The product obtained as mixture of isomers *endo*/*exo* = 88:12. Yield 32.7%, 12.0 mg, 0.065 mmol, transparent oil.

^{1}H NMR (501 MHz, CD_2Cl_2) δ 9.77 (d, J = 2.6 Hz, $1H_{min}$), 9.41 (d, J = 2.7 Hz, $1H_{maj}$), 6.38 (dd, J = 5.7, 2.9 Hz, $1H_{maj+min}$), 6.25 (dd, J = 5.7, 3.2 Hz, $1H_{maj+min}$), 3.72 (d, J = 10.7 Hz, $1H_{maj+min}$), 3.64 (d, J = 10.8 Hz, $1H_{maj+min}$), 3.08–3.03 (m, $1H_{maj+min}$), 2.72–2.69 (m, $1H_{maj+min}$),

2.65 (t, J = 3.1 Hz, 1$H_{maj+min}$), 1.62 (dt, J = 9.1, 1.7 Hz, 1$H_{maj+min}$), 1.50 (dt, J = 9.1, 1.9 Hz, 1$H_{maj+min}$), 1.30 (s, 3H_{min}), 1.05 (s, 3H_{maj}).

^{13}C NMR (126 MHz, CD_2Cl_2) δ 204.64_{maj}, 204.55_{min}, 137.12_{maj}, 137.08_{min}, 136.9_{min}, 136.0_{maj},

60.6_{maj}, 58.5_{min}, 56.9_{maj}, 56.0_{min}, 52.32_{min}, 51.35_{maj}, $49.9_{maj+min}$, 46.9_{maj}, 46.7_{min}, 45.9_{maj}, 44.6_{min}, 21.5_{min}, 20.0_{maj}.

HRMS *m/z* (API-MS): calcd. for $C_{10}H_{13}Cl_1O_1Na_1$ ($[M+Na]^+$): 207.05471; found 207.05464.

GC (29.5 m BGB-176, injection temperature: 220 °C; 100 °C–iso 5 min, 1 °C/min, 180 °C; 5 °C/min, 220 °C iso 10 min, 0.5 bar H_2)

t_{R1} = 60.03 min (minor), t_{R2} = 60.66 min (minor), t_{R3} = 64.43 min (major), t_{R4} = 65.19 min (major); e.r. (minor) = 93:7 (86% e.e.), e.r. (major) = 99:1 (98% e.e.)

$[\alpha]_D^{25} = -31.27$ (c = 0.366, $CHCl_3$)

4.2. Limitations of the Method

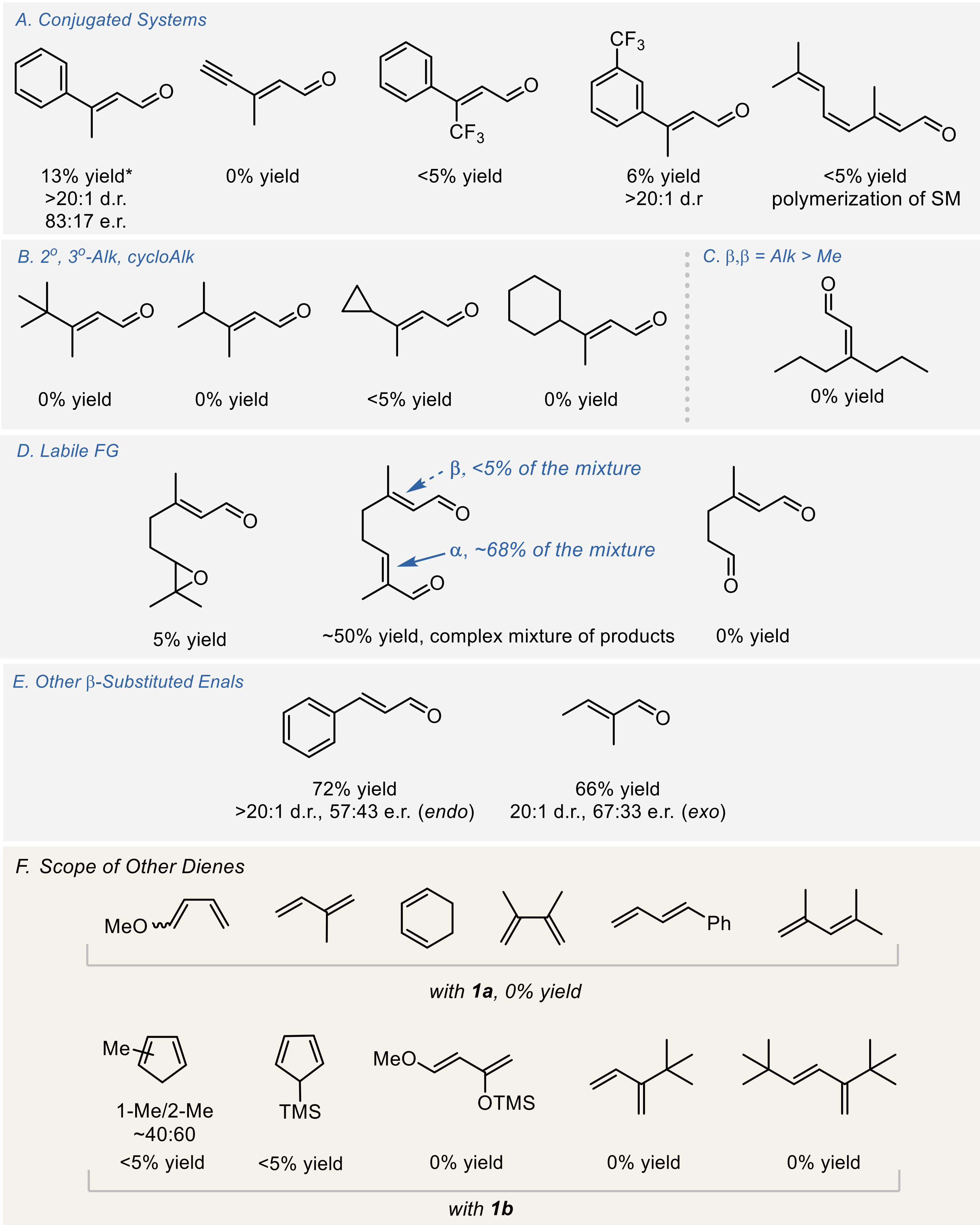

Figure S5. Unsuccessful substrates. *The reaction performed at −30 °C, at the standard temperature (−40 °C) only traces of the product were observed.

*Reactions were performed on 0.2 mmol scale using GP3 and IDPi **3f**, unless otherwise noted. For the dienophile scope cyclopentadiene (**2**) was used as diene.*

Conjugated enals (A.) generally afforded little to none of the desired Diels–Alder product, presumably due to their reduced dienophilic reactivity/electrophilicity resulting from the extended conjugation of the double bond with aryl substituents.[18]

Secondary and tertiary alkyl substituents in β-position, as well as replacing the β-methyl group with larger alkyl substituents (B., C.), likewise prevented formation of the desired product. This limitation is likely attributable to increased steric interference of the said alkyl groups during approaching to the transition state.

Introduction of labile functional group in dienophile like aldehyde or epoxide (D.) led to complex reaction mixtures involving reactions of the labile groups rather than Diels–Alder reactivity. In competition experiment with the substrate containing both α,β- and β,β-disubstituted enal moieties, a mixture of products was formed; the Diels–Alder product of α,β-substituted enal "end" was found to be the major constituent, consistent with its greater intrinsic dienophilic reactivity.[19]

We also examined other classes of β-substituted enals (E.), including mono-β-substituted and α,β-disubstituted enals. Although these substrates delivered good yields of the desired Diels–Alder adducts with excellent diastereoselectivity, only low to moderate enantioselectivity with IDPi **3f** was achieved.

The diene scope (F.) was found to be largely limited to cyclopentadiene. In reactions with **1a**, common acyclic dienes, such as isoprene and substituted 1,3-butadienes, which lack a locked *s-cis* conformation, were found to be almost unreactive and failing to provide the desired product. Moreover, steric effects further limit the use of substituted dienes in combination with the sterically demanding β,β-disubstituted enals. Increasing the reaction time (7 days) and temperature (−30 °C) did not lead to a significant improvement in yield.

We thus investigated a less sterically demanding enal, prenal **1b**. Several cyclopentadiene derivatives were also evaluated as diene substrates, motivated by their structural similarity to **2** and the successful results obtained with the latter. However, even methylcyclopentadiene, the simplest substituted cyclopentadiene, failed to provide a reasonable yield of the desired product. Instead, already after 48 h the diene was found to be completely consumed, potentially as a result of protonation[20] and subsequent side reactions/rearrangements of the resulting stabilized allylic cations. Lowering the reaction temperature to −60 °C did not improve the product yield, which remained below 5%, but slowed the oligomerization of prenal. Addition of an extra equivalent of

methylcyclopentadiene midway through the reaction (after 48 h) also failed to improve the product yield.

Only traces of the product were observed with TMS-cyclopentadiene; in contrast to methylcyclopentadiene, however, the diene remained largely intact. Increasing the reaction temperature to −30 °C and even −20 °C did not lead to product formation, while prenal oligomerization proceeded to completion. Finally, the highly reactive Danishefsky diene also failed to afford the desired product, most likely due to competing Mukaiyama aldol/hetero-Diels–Alder reaction pathways.[22,23]

5. IDPi Catalyst Synthesis and Characterization

The IDPi catalyst was synthesized according to previously reported procedures.[1] Spectroscopic data is in agreement with the previous reports.

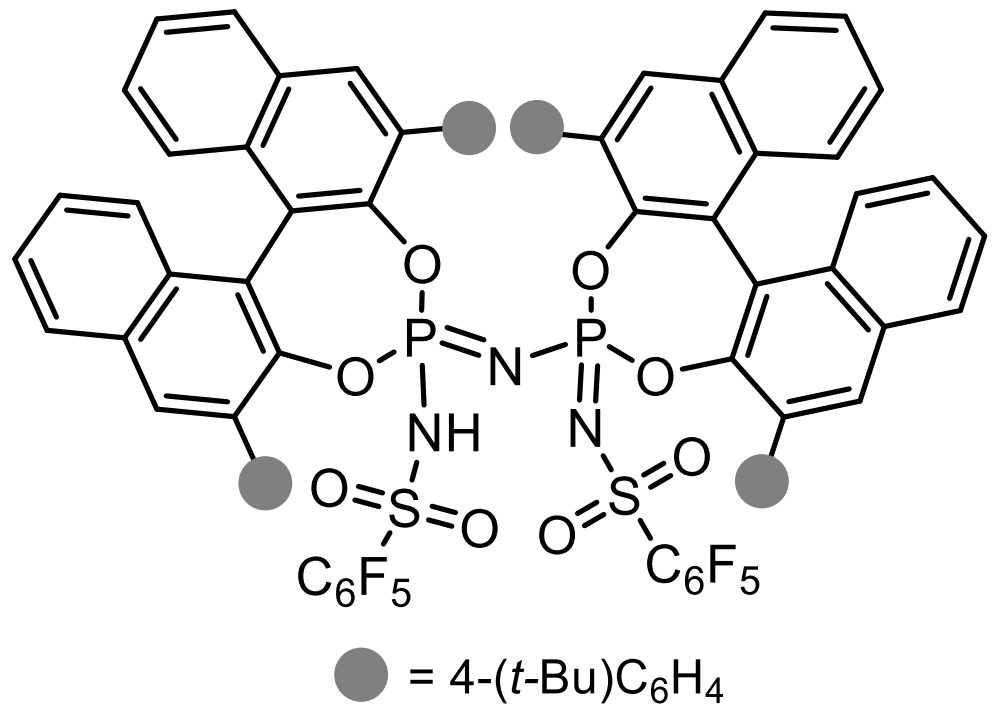

***N,N′*-(azanediylbis(2,6-bis(4-(*tert*-butyl)phenyl)-4λ^5-dinaphtho[2,1-*d*:1',2'-*f*][1,3,2]dioxaphosphepine-4-yl-4-ylidene))bis(2,3,4,5,6-pentafluorobenzenesulfonamide) (3f)**

^{1}H NMR (600 MHz, CD_2Cl_2) δ 8.21 (ddt, *J* = 8.2, 1.3, 0.6 Hz, 2H), 8.08 (s, 2H), 8.00 (d, *J* = 8.4 Hz, 2H), 7.91 (s, 2H), 7.74 (ddd, *J* = 8.2, 6.8, 1.1 Hz, 2H), 7.61 – 7.56 (m, 2H), 7.53 (ddd, *J* = 8.4, 6.8, 1.3 Hz, 2H), 7.42 (s, 8H), 7.39 (d, *J* = 8.4 Hz, 2H), 7.38 – 7.36 (m, 4H), 6.75 (bd, *J* = 8.6 Hz, 4H), 6.70 (bs, 1H, NH), 6.62 (bd, *J* = 8.6 Hz, 4H), 1.26 (s, 18H), 0.93 (s, 18H).

^{13}C NMR (151 MHz, CD_2Cl_2) δ 151.4, 150.6, 144.2 (dd, *J* = 259.9, 11.5 Hz), 143.9 (t, *J* = 5.1 Hz), 143.8 (t, *J* = 5.7 Hz), 143.5 (dt, *J* = 261.3, 13.3 Hz), 137.4 (bdt, *J* = 256.8, 14.8 Hz), 134.5 (t, *J* = 1.3 Hz), 133.4 (t, *J* = 1.8 Hz), 133.3, 132.8, 132.4, 132.1, 132.1, 131.7, 131.5, 130.8, 129.5, 129.3, 129.2, 128.7, 127.6, 127.19, 127.16, 127.1, 127.0, 126.2 125.0, 123.7 (t, *J* = 1.4 Hz), 123.0 (t, *J* = 1.3 Hz), 117.6 (t, *J* = 14.1 Hz), 34.8, 34.6, 31.3, 31.2.

^{19}F NMR (565 MHz, CD_2Cl_2) δ −135.61 (d, *J* = 20.7 Hz), −146.45 (bs), −160.42 (bt, *J* = 21.0 Hz).

^{31}P NMR (203 MHz, CD_2Cl_2) δ −7.91.

HRMS *m/z* (ESI(−)): calcd. for $C_{92}H_{72}F_{10}N_3O_8P_2S_2$ ([M-H]$^-$): 1662.4082; found 1662.4067.

*Synthesis of sodium salt of **3f** (IDPi-Na)*

In a glass vial 8.0 mg of **3f** were dissolved in 300 µL of DCM followed by addition of 300 µL of distilled water. Then Na_2CO_3 (1.5 equiv., 0.0073 mmol, 0.8 mg) was added to the vial. The obtained biphasic mixture was vigorously stirred for 3 hours and then centrifuged to separate the layers. The upper layer was removed using syringe, the remaining organic solution was evaporated to dryness and then dried in high vacuum at 40 °C for 2 d.

6. Mechanistic Experiments

6.1. Stoichiometry Experiment

For the stoichiometric experiment between **1a** and **3f** 40.0 mg (0.024 mmol) of the latter were dissolved in 350 µL of DCM-d_2 and 60.0 mg of 5 Å MS were added. The obtained mixture was cooled down to −40 °C and 4.0 µL (0.024 mmol) of geranial were added. After stirring for 30 min, the mixture was transferred to NMR tube, molecular sieves were allowed to settle down and NMR spectra were acquired at −40 °C.

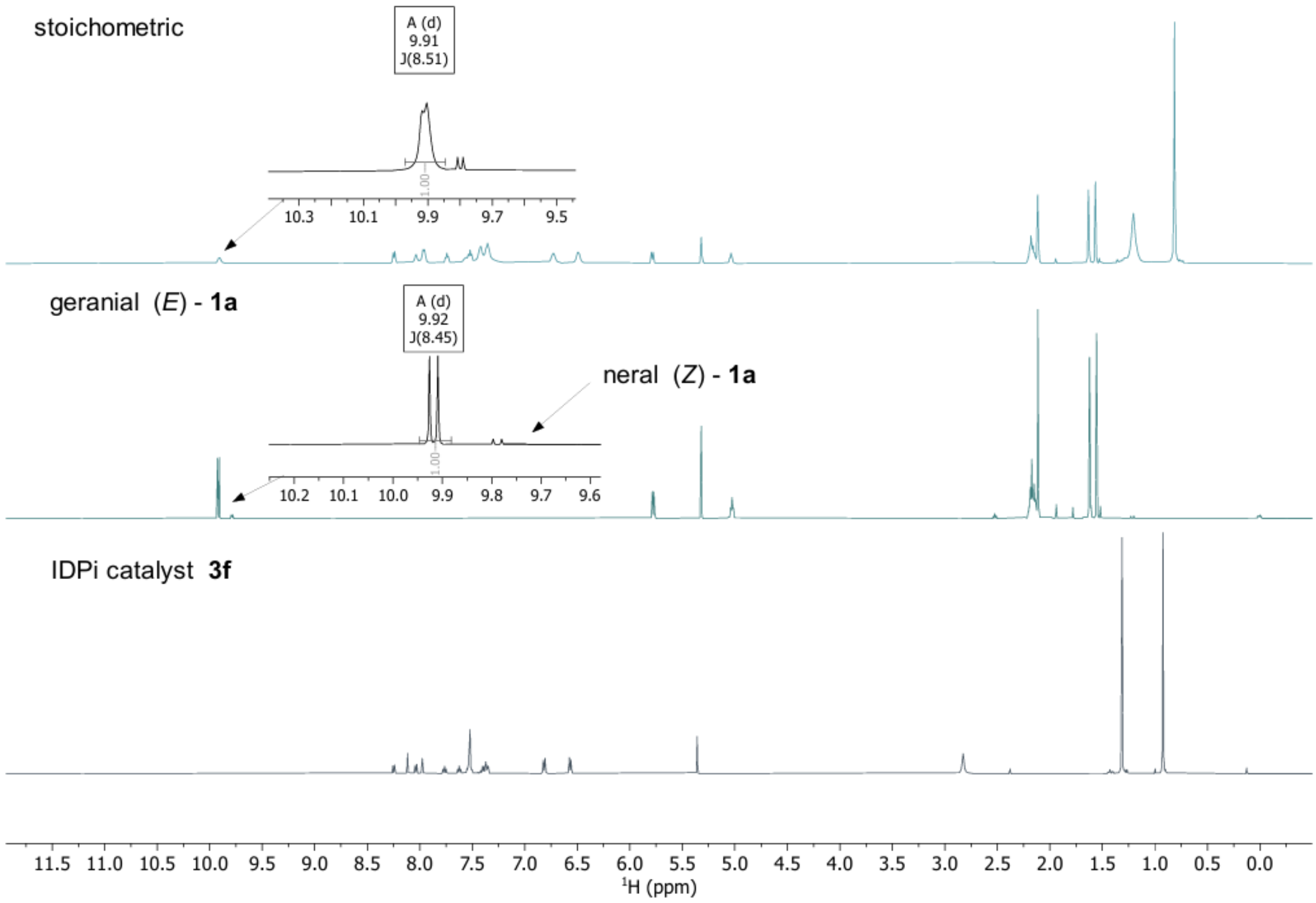

Figure S6. Substrate activation by IDPi catalyst **3f** observed by ^{1}H NMR

Only weak activation of the carbonyl group was observed in the experiment represented by broadening of the geranial signals (neral signals remain almost intact).

6.2. Interactions of the IDPi catalyst and MS

As *in situ* NMR monitoring was not feasible due to absence of reactivity without stirring, we sought to investigate the elementary steps of the reaction.

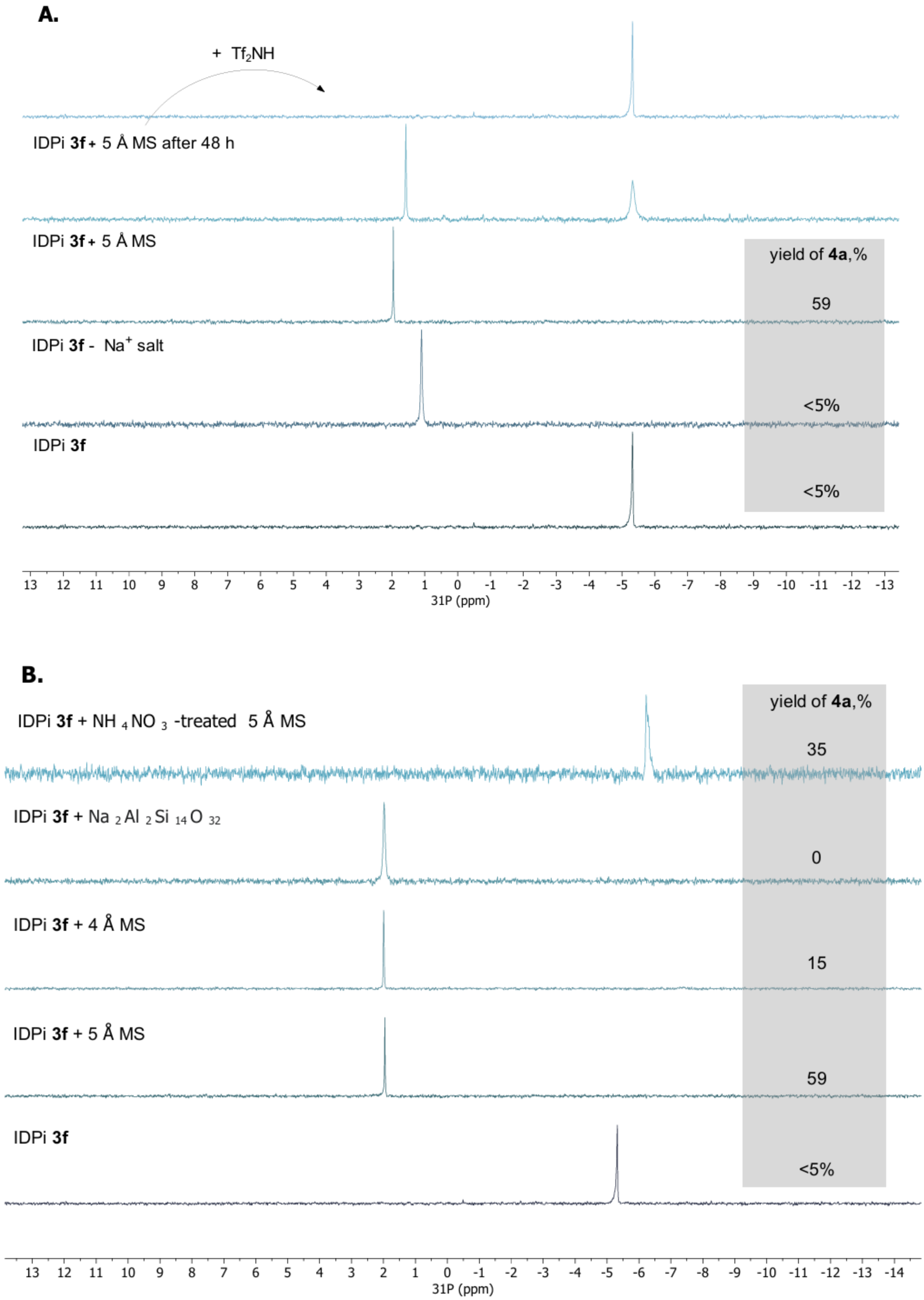

Figure S7. **A**. ^{31}P NMR comparison of IDPi **3f**, its mixture with 5 Å MS and sodium salt of **3f**. **B**. ^{31}P NMR analysis of IDPi **3f** mixtures with various additives.

Samples preparation: the samples were prepared by dissolving 5.0 mg of **3f** in 300 µL of MTBE followed by addition of 20.0 mg of an additive. The obtained suspension was stirred for 20

min and then transferred to NMR tube equipped with the inlet containing toluene-*d8*. In case of pure IDPi **3f** and IDPi-Na **3f** (see Chapter 5 for the details of the synthesis) the samples were prepared analogously by dissolving 5.0 mg of the compound in 300 µL of MTBE and then directly transferring to NMR tube equipped with the inlet containing toluene-*d8.*

Upon mixing **3f** with 5 Å MS significant shift in ^{31}P NMR spectrum is observed (−5.8 ppm vs 1.9 ppm). Given that molecular sieves are zeolites containing exchangeable cations (Na^+ in case of 5 Å and 4 Å MS, and K^+ in case of 3 Å MS)[24] and IDPis are strong organic Brønsted acids the observed shift can be explained by Na^+–H^+ exchange.[25] This event would generate sodium salt of IDPi and OH-acidic site on the molecular sieve surface. However, independently synthesized sodium salt of IDPi ("IDPi-Na", see Chapter 5 for the details of the synthesis) demonstrated slightly different ^{31}P shift and did not deliver Diels−Alder product when utilized in the reaction (both when utilized solely and together with 5 Å MS). At the same time, new observed species were found to convert back to acidic form of IDPi upon standing and during the reaction course (Figure S7, A).

Further experiments with different types of molecular sieves and additives revealed that formation of new species is not required to achieve the observed reactivity. Among molecular sieves of different pore size ones containing Na^+ demonstrated the best yields (see 4 Å MS, Figure S7, B). Additionally, when non-zeolite structured analog of such molecular sieves—sodium aluminosilicate —was utilized in the reaction no product formation was observed, despite ^{31}P NMR showing the presence of the same new species as in case with 5 Å MS.

To further investigate the role of these new species, hydrogen-exchanged ("H-substituted") analog of 5 Å MS was prepared by treatment with NH_4NO_3 and subsequent calcination as described by Zhang and coworkers.[26] This modified material retains the zeolite crystal structure and, therefore, can function as a molecular sieve but lacks Na^+ (as can be seen from elemental mapping (EDS) analysis, Figure S8) to undergo ion exchange with IDPi and generate the new species observed with 5 Å MS. The absence Na^+-salt formation can be clearly seen from ^{31}P NMR spectrum. *Notably, in this case, the product formation was still observed, albeit in slightly reduced yield, despite the absence of new species formation* (Figure S7, B).

In summary, these experiments indicate that the primary and most critical role of the molecular sieves is to serve as a drying agent, removing residual moisture from both the reaction mixture and catalytic pocket of the IDPi **3f**. The observed formation of the catalytically inactive Na^+-salt of IDPi

via cation exchange with 5 Å MS might serve for attenuation of IDPi reactivity, preventing it from undesired acid-catalyzed reactions with labile enals.

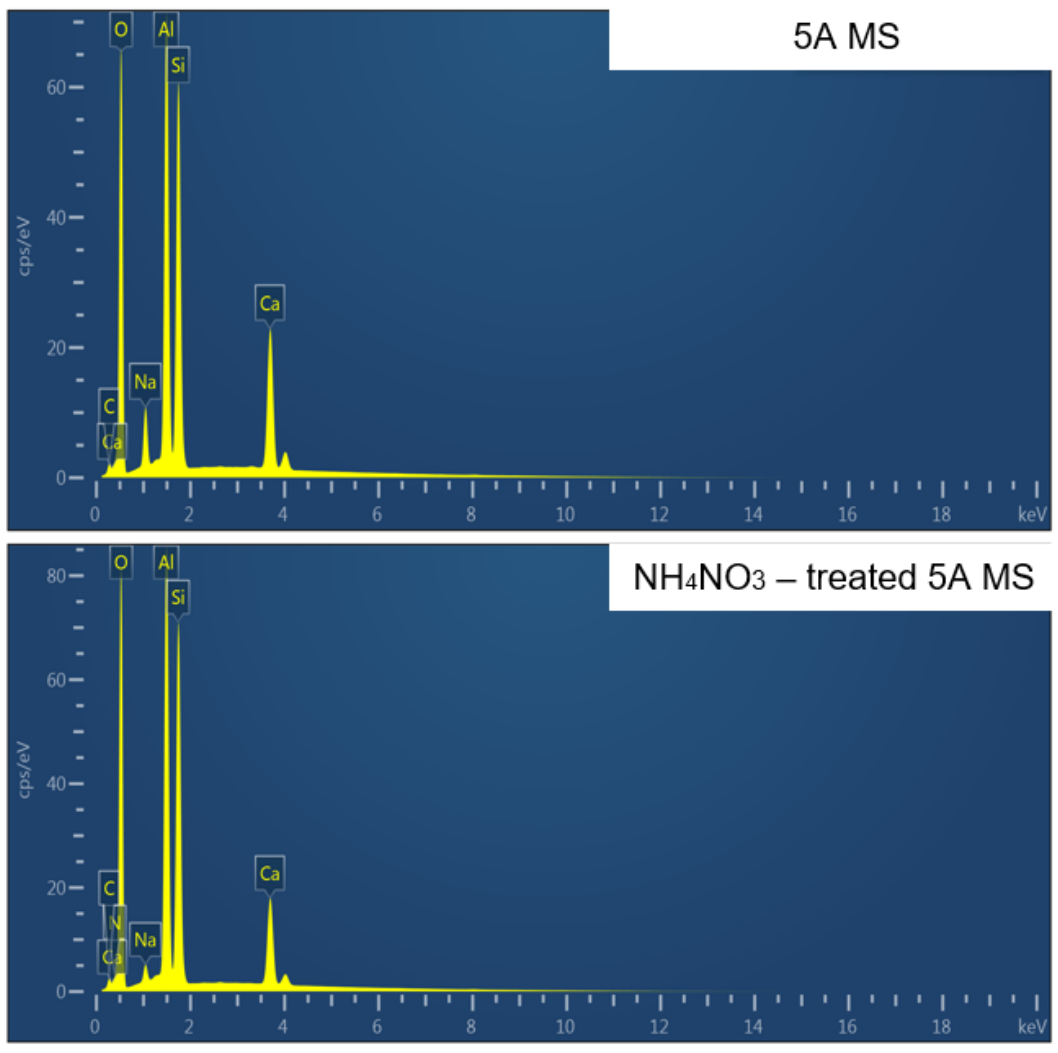

Element weight, %	5 Å MS	NH_4NO_3 – treated 5 Å MS
O	51.02	53.23
Na	3.26	1.09
Al	16.97	18.45
Si	16.99	18.53
Ca	11.77	8.49
N	0.0	0.2

Figure S8. Characterization of elemental composition of NH_4NO_3–treated 5 Å MS and its comparison with untreated 5 Å MS.

6.3. Characterization of Molecular Sieves after the Diels−Alder Reaction

Physical properties of the molecular sieves before and after reaction were studied by powder diffraction and FT-IR methods.

Powder XRD

Powder XRD measurements were performed on Stoe STADI P Transmission Powder Diffractometer. The comparison of diffraction data from the molecular sieves before and after the reaction revealed no significant changes in crystalline or chemical structure. The slight 2θ shifts of the main diffraction peaks indicate expansion of zeolite framework, which can be explained by the presence of adsorbed molecules (for example, water) (Figure S9). This also might be a consequence of substitution of bulkier Na^+ with H^+ in zeolite framework.

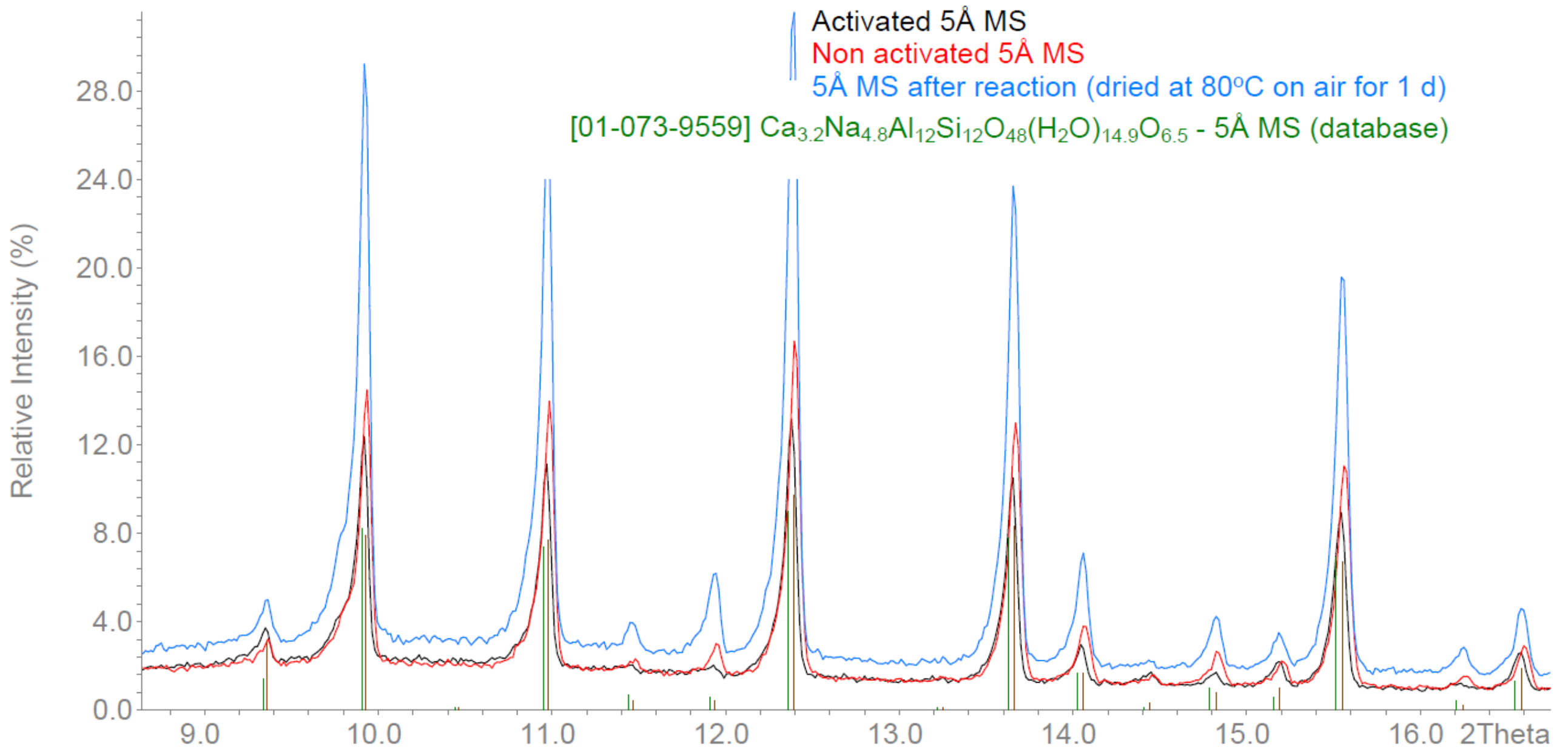

Figure S9. Comparison of 5 Å MS before and after the Diels−Alder reaction.

6.4. Intramolecular Competition ^{13}C Kinetic Isotope Effect at Natural Abundance

For the determination of ^{13}C KIE at natural abundance 100 mg of alcohol **4a-R** were used. Major (*endo*) isomer was separated using preparative LC (Kromasil 100-5-C18, acetonitrile/water = 50:50, 42.5 mL/min, 13.5MPa, 308 K; retention time (*endo*) = 62.5 min).

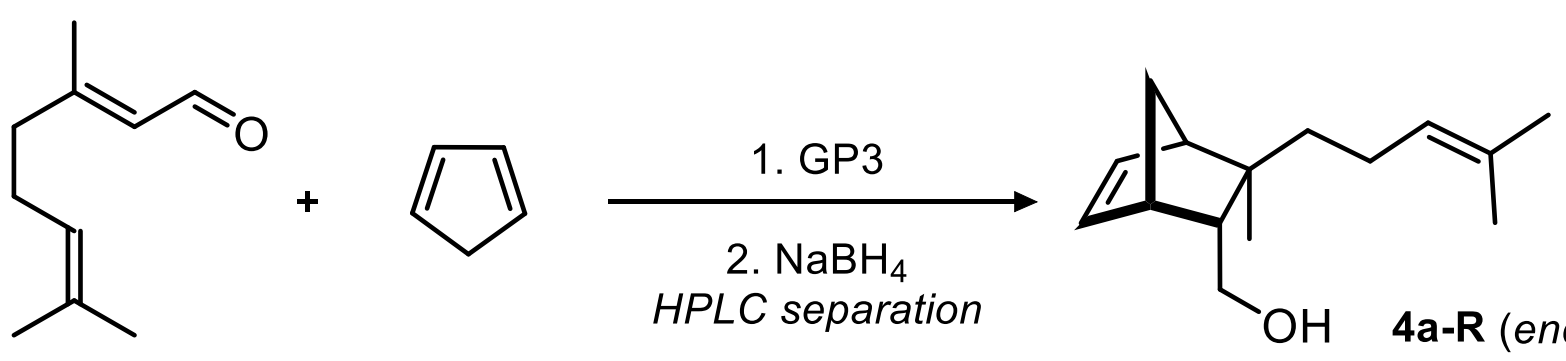

4a-R (*endo*)

***Table S2**. Crude dataset (integrals and relative integrals) for the ^{13}C KIE measurements.*

Integrals (a.u. × 10^{-12})					Relative Integrals					KIE (^{12}C/^{13}C)				
#	C3	C4	C5	C6	#	C3	C4	C5	C6	#	C3	C4	C5	C6
1	1.7224	1.6377	1.6320	1.6692	1	1.0000	1.0000	0.9966	0.9691	1	1.000	1.000	1.003	1.032
2	1.7246	1.6264	1.6319	1.6574	2	1.0000	1.0000	1.0034	0.9610	2	1.000	1.000	0.997	1.041
3	1.7222	1.6346	1.6220	1.6587	3	1.0000	1.0000	0.9923	0.9631	3	1.000	1.000	1.008	1.038
4	1.7302	1.6261	1.6198	1.6526	4	1.0000	1.0000	0.9961	0.9551	4	1.000	1.000	1.004	1.047
5	1.7229	1.6271	1.6154	1.6608	5	1.0000	1.0000	0.9928	0.9639	5	1.000	1.000	1.007	1.037
6	1.7290	1.6252	1.6301	1.6565	6	1.0000	1.0000	1.0030	0.9580	6	1.000	1.000	0.997	1.044
7	1.7199	1.6255	1.6208	1.6540	7	1.0000	1.0000	0.9971	0.9617	7	1.000	1.000	1.003	1.040
8	1.7172	1.6267	1.6228	1.6495	8	1.0000	1.0000	0.9976	0.9606	8	1.000	1.000	1.002	1.041
					av	1.000 ± 0.000	1.000 ± 0.000	0.997 ± 0.004	0.962 ± 0.004	av	**1.000 ± 0.000**	**1.000 ± 0.000**	**1.003 ± 0.004**	**1.040 ± 0.004**

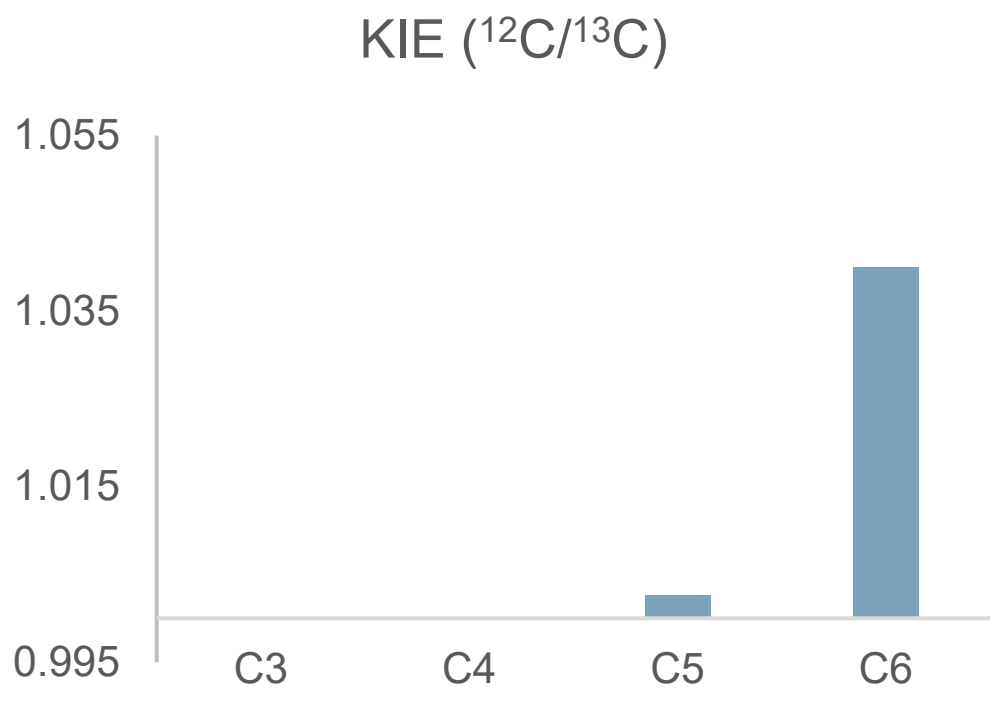

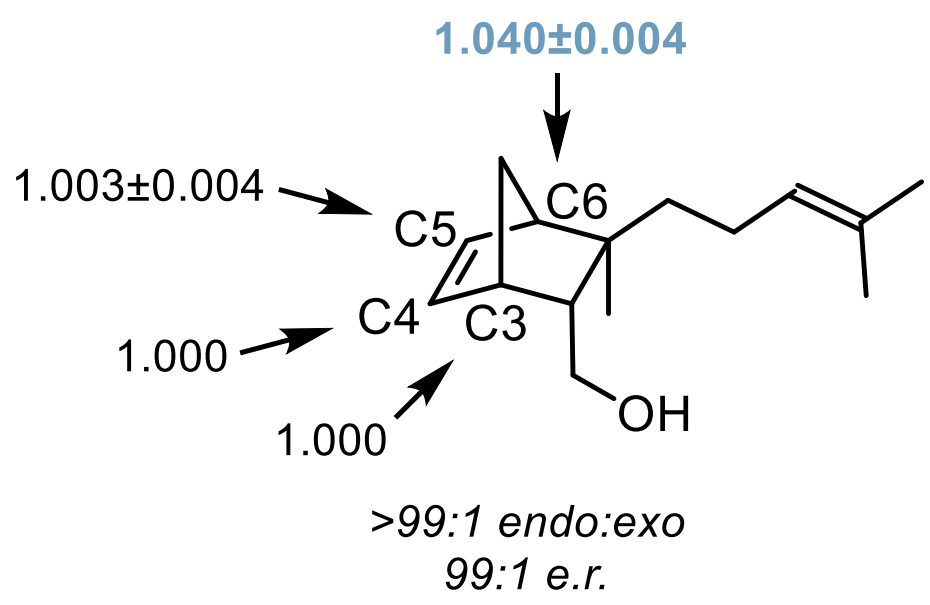

Figure S10. ^{13}C KIEs measurements for **4a-R**.

Quantitative ^{13}C NMR spectra were acquired on Bruker AvanceNeo 600 MHz NMR spectrometer at 298 K equipped with a cryogenically-cooled BBO probe using an inversegated decoupling ^{13}C NMR sequence after a pi/6 pulse (^{13}C pulse length 3.33 µs; bruker pulse sequence: zgig30). A relaxation delay of 120 s (7.5*T1(C5), longest T1 of carbons of interest) was used. The average of eight consequent measurements was taken.

The observed KIEs support highly asynchronous concerted [4+2] cycloaddition mechanism and points towards the significant polarization of the enal component.[27]

7. Total Syntheses of (−)-β-Santalene, (−)-(*Z*)-β-Santalol, and (−)-Camphene

7.1. Upscaling of the Diels−Alder Reactions

*Upscaling of the Diels−Alder reaction between **1a** and **2** (100 mmol), IDPi-Catalyst and Molecular Sieves Recovery*

Activated molecular sieves 5 Å (35.0 g) and IDPi catalyst **3f** (1.48 mol%, 1.48 mmol, 2.47 g) were loaded into a flame-dried 250 mL round-bottom flask equipped with a stirring bar. MTBE (1.7 M, 60.0 mL) was added and the obtained mixture was stirred for 15 min at rt. Then the reaction vessel was cooled down in dry ice bath and cyclopentadiene **2** (2.5 equiv., 250.0 mmol, 20.6 mL) was added. The resulting mixture was stirred for 10 min in dry ice before adding geranial **1a** (1.0 equiv., 100.0 mmol, 17.1 mL). The reaction vessel was placed in −40 °C cryostat filled with EtOH as cooling agent and stirred for 7 d.

After completion of the reaction, it was quenched by the addition of triethylamine (0.03 equiv., 0.4 mL) and allowed to warm up to rt. Molecular sieves were removed by filtration through a fritted glass filter and obtained solution was concentrated under reduced pressure.

The crude product was purified by column chromatography *n*-pentane/MTBE (9:1) to afford 14.7 g (67.33 mmol, 67.3% yield) of the pure product **4a** with 87:13 *endo*/*exo* ratio and 99:1 e.r. (*endo*) as yellowish oil.

The catalyst was recovered by washing the chromatography column with *n*-pentane/MTBE (1:1) and acidified over Dowex® resin. After drying in high vacuum at 40 °C for 24 h, 2.40 g (97.2%) of the IDPi catalyst were recovered.

The molecular sieves obtained after reaction were re-activated at 130 °C in high vacuum, 4 d (33.9 g, 96.5%), and tested in the standard Diels−Alder reaction set up according to GP3 as well as the recovered IDPi **3f**.

Table S3. Recovery tests of MS and **3f**

Recovered 5 Å MS			
Run	1	2	3
Yield **4a**, %	67	60	58
e.r.	99:1		

Recovered 3f			
Run	1	2	3
Yield **4a**, %	63	59	65
e.r.	99:1		

*Upscaling of the Diels−Alder reaction between **1b** and **2** (10 mmol)*

Analogously to the upscaling procedure for **1a** and **2** using molecular sieves 5 Å (3.5 g) and IDPi catalyst **3f** (1.5 mol%, 0.15 mmol, 247.0 mg), MTBE (1.7 M, 6.0 mL), cyclopentadiene **2** (2.5 equiv., 25.0 mmol, 2.06 mL) and prenal **1b** (1.0 equiv., 10.0 mmol, 0.96 mL).

The crude product was purified by flash column chromatography *n*-pentane/MTBE (7:1) to afford 891.0 mg (5.94 mmol, 59.3% yield) of **4b** with 89:11 *endo*/*exo* ratio and 95:5 e.r.

7.2. Citral as a Geranial Source

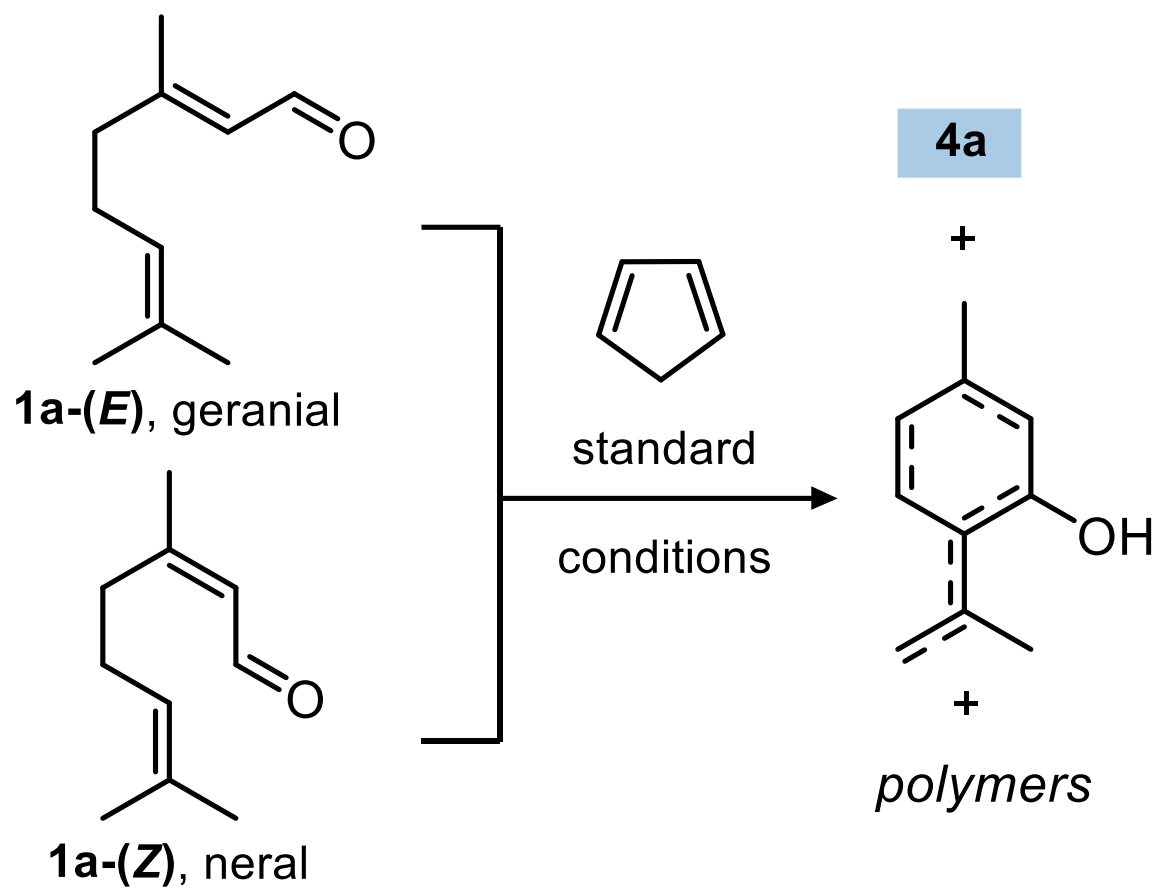

		citral	
**1a**-(*E*)	95	65	5
**1a**-(*Z*)	5	35	95
conv., %	full	77	79
yield **4a**, %	59	24	traces

Figure S11. The Diels−Alder reaction of citral and cyclopentadiene.

As part of our continued efforts to develop a more accessible synthesis of (−)-(*Z*)-β-santalol, we turned our attention to citral—a commercially available 65:35 mixture of geranial (**1a-(*E*)**) and neral (**1a-(*Z*)**)—as a substrate for the Diels–Alder reaction. Under standard reaction conditions, we found that the geranial component of citral could be converted to the desired Diels–Alder adduct without any loss in enantioselectivity, albeit in reduced yield. No corresponding Diels–Alder product from neral was detected; instead, neral underwent rapid cyclization followed by decomposition, consistent with previous observations in the presence of IDPi catalysts[28] and montmorillonite clay or zeolite-β.[29] Despite the lower yield, these results suggest that commercially available citral can be used in place of pure geranial to obtain the target enantiopure intermediate **4a**.

7.3. Intermediate Alcohols 4a-R and 5

((1*S*,2*R*,3*R*,4*R*)-3-methyl-3-(4-methylpent-3-en-1-yl)bicyclo[2.2.1]hept-5-en-2-yl)methanol (4a-R)

To a stirred solution of aldehyde **4a** obtained in previous step (1.0 equiv., 13.78 mmol, 3.02 g) in EtOH (0.17 M, 80 mL) was added $NaBH_4$ (1.5 equiv., 20.68 mmol, 782.0 mg) at rt and stirring was continued for 3 h. The reaction mixture was diluted with Et_2O (80 mL), washed with H_2O (3x150 mL) and saturated aqueous NaCl (200 mL), dried over anhydrous $MgSO_4$ and concentrated under reduced pressure to give crude alcohol which was purified by column chromatography *n*-pentane/MTBE (4:1) to afford the pure product in 92.3% yield (2.79 g, 12.68 mmol) as colorless oil.

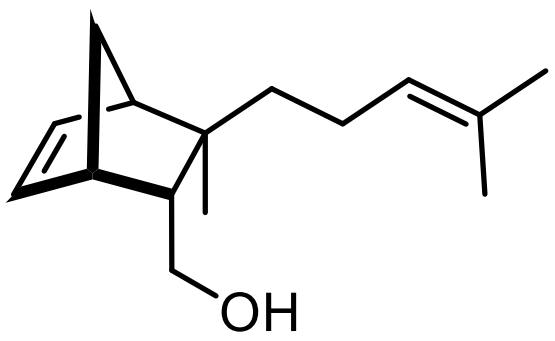

The product obtained as mixture of isomers *endo*/*exo* = 87:13, NMR data for the major (*endo*) diastereomer is reported.

^{1}H NMR (501 MHz, $CDCl_3$) δ 6.23 (dd, *J* = 5.7, 3.1 Hz, 1H), 6.18–6.13 (m, 1H), 5.13 (tdd, *J* = 7.2, 2.9, 1.5 Hz, 1H), 3.45 (dd, *J* = 10.5, 6.1 Hz, 1H), 3.27–3.19 (m, 2H+1H), 2.90–2.85 (m, 1H), 2.46–2.41 (m, 1H), 2.04 (dq, *J* = 12.7, 6.7 Hz, 2H), 1.68 (bs, 3H), 1.66–1.58 (m, 3H+1H), 1.58–1.49 (m, 1H), 1.51–1.38 (m, 2H), 0.75 (s, 3H).

^{13}C NMR (126 MHz, $CDCl_3$) δ 138.6, 133.9, 131.3, 125.1, 64.8, 53.3, 51.4, 46.7, 45.5, 44.8, 43.8, 25.8, 23.9, 18.9, 17.8.

HRMS *m/z* (GC-EI): calcd. for $C_{15}H_{24}O_1$ ($[M]^+$): 220.182165; found 220.182270.

$[\boldsymbol{\alpha}]_D^{25}$ = –17.59 (c = 0.71, $CHCl_3$)

((1*R*,2*R*,3*R*,4*S*)-3-methyl-3-(4-methylpent-3-en-1-yl)bicyclo[2.2.1]heptan-2-yl)methanol (5)

The procedure is adopted from Baldovini and Solladié.[30]

To a solution of $Ni(OAc)_2\cdot4H_2O$ (0.20 equiv., 585.0 mg, 2.35 mmol) in 95% ethanol (100 mL) was added in one portion a solution of $NaBH_4$ (0.16 equiv., 71.1 mg, 1.88 mmol) and NaOH (0.008 equiv., 3.8 mg, 0.094 mmol) in aqueous ethanol (35 mL EtOH + 0.5 mL of H_2O). The reaction flask was purged with H_2 and the unsaturated alcohol **4a-R** from the previous step (1.0 equiv., 2.59 g, 11.75 mmol) was added as a solution in 20 mL of EtOH. The obtained reaction mixture was stirred at rt for 3 h under H_2 atmosphere.

The reaction mixture was then poured into 1 M HCl (150 mL) and extracted with diethyl ether (2x100 mL). The organic phases were washed with brine, dried over $MgSO_4$ and evaporated to afford

the crude product which was purified by column chromatography *n*-pentane/MTBE (4:1) to afford the pure product in 82.0% yield (2.14 g, 9.63 mmol) as colorless oil.

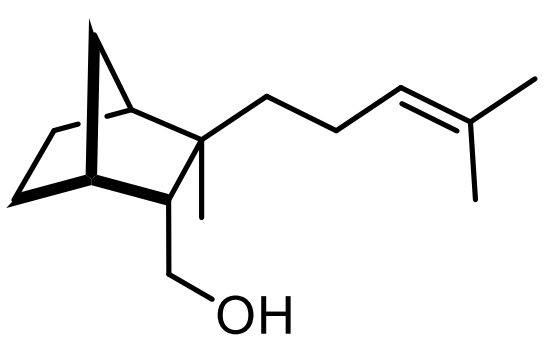

^{1}H NMR (501 MHz, $CDCl_3$) mixture of two diastereomers *endo*/*exo* = 88:12, NMR data for the major diastereomer is reported. δ 5.08 (dddd, *J* = 8.5, 5.7, 2.9, 1.4 Hz, 1H), 3.61 (d, *J* = 7.8 Hz, 2H), 2.30–2.24 (m, 1H), 1.96–1.85 (m, 1H+2H), 1.67 (d, *J* = 1.5 Hz, 3H), 1.65–1.54 (m, 3H+3H), 1.51–1.44 (m, 1H), 1.40–1.23 (m, 4H), 1.19 (dt, *J* = 9.7, 1.7 Hz, 1H), 0.81 (s, 3H).

^{13}C NMR (126 MHz, $CDCl_3$) δ 131.1, 125.0, 61.3, 51.7, 47.2, 44.7, 39.9, 39.3, 37.0, 25.7, 24.6, 22.9, 20.7, 17.6, 17.0.

HRMS *m/z* (API-MS): calcd. for $C_{15}H_{26}O_1Na_1$ ($[M+Na]^+$): 245.187584; found 245.187410.

$[\alpha]_D^{25}$ = –3.55 (c = 0.56, $CHCl_3$)

One-Pot Procedure for Reduction of Aldehyde and C=C Bond

To a solution of $Ni(OAc)_2{\cdot}4H_2O$ (0.20 equiv.) in 95% ethanol was added in one portion a solution of $NaBH_4$ (0.16 equiv.) and NaOH (0.008 equiv.) in aqueous ethanol. The reaction flask was purged with H_2 and the unsaturated aldehyde (1.0 equiv.) was added as a solution in EtOH. The obtained reaction mixture was stirred at rt for 3 h under H_2 atmosphere.

Excess of $NaBH_4$ (2 equiv.) was added and the reaction was left overnight.

The reaction mixture was then poured into 1 M HCl (150 mL) and extracted with diethyl ether (2x100 mL). The organic phases were washed with brine, dried over $MgSO_4$ and evaporated to afford the crude product, which was purified by column chromatography *n*-pentane/MTBE (4:1) to afford the pure product in overall 70.6% yield (2.35 g, 10.59 mmol) starting with 15.0 mmol of aldehyde **4a**.

7.4. Intermediate Alcohol 8

((1*R*,2*R*,4*S*)-3,3-dimethylbicyclo[2.2.1]heptan-2-yl)methanol (8)

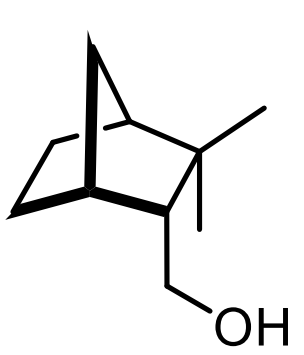

Obtained from 891.0 mg (5.94 mmol) of **4b** using the one-pot reduction procedure described above. The crude product was purified using flash chromatography *n*-pentane/MTBE (4:1). Yield 78.0% (712.4 mg, 4.63 mmol).

Mixture of two diastereomers *endo*/*exo* = 83:17, NMR data for the major diastereomer is reported.

^{1}H NMR (501 MHz, CD_2Cl_2) δ 3.64–3.54 (m, 2H), 2.26–2.22 (m, *J* = 4.6, 2.1 Hz, 1H), 1.77–1.72 (m, 1H), 1.65 (dt, *J* = 9.6, 2.3 Hz, 2H), 1.60–1.53 (m, 2H), 1.38–1.33 (m, 1H), 1.32–1.24 (m, 1H), 1.19 (dt, *J* = 9.7, 1.7 Hz, 1H), 0.99 (s, 3H), 0.84 (s, 3H).

^{13}C NMR (126 MHz, CD_2Cl_2) δ 61.3, 53.1, 49.6, 40.3, 37.4, 37.1, 32.8, 24.9, 20.6.

HRMS *m/z* (API-MS): calcd. for $C_{10}H_{18}Na_1O_1$ ($[M+Na]^+$): 177.12499; found 177.12496.

The data are in agreement with the previous reports.[6]

7.5. Intermediate Carbonate 5-C

Methyl (((1*R*,2*R*,3*R*,4*S*)-3-methyl-3-(4-methylpent-3-en-1-yl)bicyclo[2.2.1]heptan-2-yl)methyl) carbonate (5-C)

To a solution of the alcohol **5** (1.0 equiv., 1.43 g, 6.42 mmol) and pyridine (6.0 equiv., 38.52 mmol, 3.13 mL) in DCM (20 ml) cooled in an ice bath methyl chloroformate (1.5 equiv., 0.75 mL, 9.63 mmol) was slowly added. After the addition was complete, stirring was continued at 0 °C overnight. The reaction mixture was carefully washed with 1 M HCl (2x100 mL) and then with saturated solution of $NaHCO_3$ (150 mL). Aqueous layer was extracted with DCM (100 mL), organic layers were combined and washed with brine (200 mL) and finally dried over anhydrous Na_2SO_4. The solvent was evaporated to afford the crude product which was purified by column chromatography *n*-pentane/MTBE (9:1) to afford the pure product in 88.7% yield (1.59 g, 5.68 mmol).

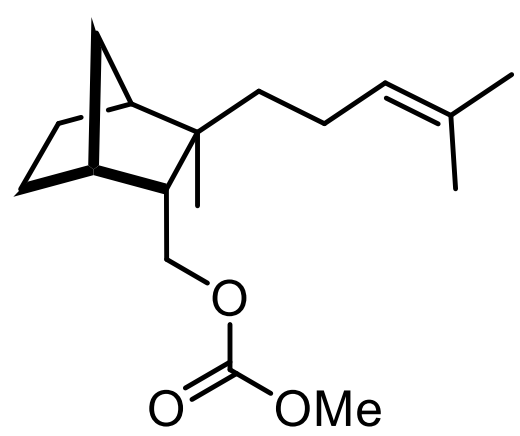

Mixture of two diastereomers *endo*/*exo* = 90:10, NMR data for the major diastereomer is reported.

^{1}H NMR (501 MHz, $CDCl_3$) δ 5.08 (tdp, *J* = 6.9, 4.2, 1.4 Hz, 1H), 4.16–4.11 (m, 2H), 3.76 (s, 3H), 2.28–2.22 (m, 1H), 1.96–1.85 (m, 1H+2H), 1.83–1.75 (m, 1H), 1.67 (bs, 3H), 1.65–1.58 (m, 2H+3H), 1.41–1.24 (m, 5H), 1.20 (dt, *J* = 9.8, 1.7 Hz, 1H), 0.84 (s, 3H).

^{13}C NMR (126 MHz, $CDCl_3$) δ 156.3, 131.5, 125.3, 67.6, 55.0, 48.1, 47.6, 44.8, 40.4, 40.1, 37.4, 26.1, 24.9, 23.2, 21.2, 18.0, 17.6.

HRMS *m/z* (API-MS): calcd. for $C_{17}H_{28}O_3Na_1$ ($[M+Na]^+$): 303.193064; found 303.193094.

$[\alpha]_D^{25}$ = –14.36 (c = 0.59, $CHCl_3$)

7.6. (−)-β-Santalene 6

(1*S*,2*R*,4*R*)-2-methyl-3-methylene-2-(4-methylpent-3-en-1-yl)bicyclo[2.2.1]heptane (6)

The carbonate **5-C** obtained in the previous step (1.12 g, 4.0 mmol) was pyrolyzed at 580 °C (10 ml/min, quartz tube, N_2) in a tube oven. Obtained oil was purified by column chromatography *n*-pentane/MTBE (100:1, R_f = 0.95) to afford the pure product in 63.7% (520.5 mg, 2.55 mmol or 87.3% brsm) yield as yellowish oil. After washing the chromatography column with *n*-pentane/MTBE (10:1), 302.6 mg (27.0%, 1.08 mmol) of the remaining carbonate were recovered.

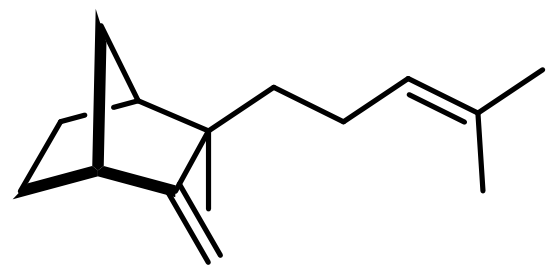

Mixture of two diastereomers *endo*/*exo* = 92:8, NMR data for the major diastereomer is reported.

^{1}H NMR (501 MHz, CD_2Cl_2) δ 5.10 (tp, *J* = 7.1, 1.4 Hz, 1H), 4.72 (bs, 1H), 4.47 (bs, 1H), 2.65 (dd, *J* = 4.5, 1.8 Hz, 1H), 2.14 (bd, *J* = 4.1 Hz, 1H), 2.06–1.88 (m, 2H), 1.73–1.59 (m, 3H+3H+3H), 1.46–1.34 (m, 2H), 1.29–1.16 (m, 4H), 1.05 (s, 3H).

^{13}C NMR (126 MHz, CD_2Cl_2) δ 167.1, 131.4, 125.5, 99.7, 47.4, 45.2, 45.1, 41.7, 37.5, 30.2, 25.9, 24.1, 24.0, 22.9, 17.7.

HRMS *m/z* (API-MS): calcd. for $C_{15}H_{25}$ ($[M+H]^+$): 205.19508; found 205.19502.

$[\alpha]_D^{25}$ = –102.16 (c = 0.69, $CHCl_3$).

The spectroscopic data and physical properties are in accordance with the literature.[31–33]

7.7. (−)-Camphene 9

(1*S*,4*R*)-2,2-dimethyl-3-methylenebicyclo[2.2.1]heptane (9)

To a stirred solution of alcohol **8** (1.0 equiv., 350.0 mg, 2.27 mmol) and TEA (4.0 equiv., 1.27 mL, 9.08 mmol) in dry DCM (5.0 mL) at 0 °C was added dropwise MsCl (1.05 equiv., 0.19 mL, 2.38 mmol). After the addition of MsCl had been completed, the reaction mixture was allowed to warm up to rt. Resulting reaction mixture was diluted with DCM (10 mL), and the resulting solution was washed with water (3x30 mL), 5% NaOH (aq.) (30 mL), water (30 mL), and brine (30 mL). The organic layer was dried over anhydrous Na_2SO_4 and concentrated in vacuo. The obtained crude was used directly in the next step without purification.

The elimination procedure is adopted from Chapuis et al.[34]

The crude obtained in the previous step was dissolved in DMF (10.0 mL) under Ar using Young tube as a reaction vessel. To the obtained vigorously stirred solution at rt were then added NaI

(~2 equiv., 680.5 mg, 4.54 mmol) and DBU (~3 equiv., 1.02 mL, 6.81 mmol), and the tube was immersed in the preheated to 90 °C oil bath. The reaction mixture was stirred for 3 d, cooled down to rt and poured onto 2% HCl (aq.) (50 mL). The product was extracted with Et_2O (3x30 mL), washed with water (100 mL) and brine (100 mL).

The obtained organic layer was dried over anhydrous Na_2SO_4, concentrated and purified by column chromatography (pure *n*-pentane, R_f = 0.9) to afford the pure product in 41.2% yield (127.4 mg, 0.94 mmol) over two steps.

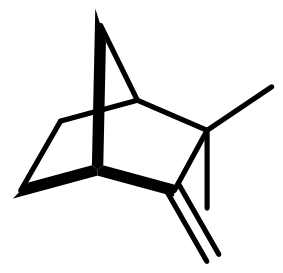

^{1}H NMR (501 MHz, CD_2Cl_2) 5.32 (s, 1H), 5.11 (s, 1H), 3.27 (dd, *J* = 4.4, 1.9 Hz, 1H), 2.51 (d, *J* = 4.0 Hz, 1H), 2.38–2.21 (m, 3H), 2.06–1.93 (m, 1H), 1.83 (dq, *J* = 9.6, 2.5 Hz, 2H), 1.66 (s, 3H), 1.63 (s, 3H).

GC (29.5 m BGB-176, injection temperature: 220 °C; 80 °C–iso 5 min, 1 °C/min, 220 °C; iso 10 min, 0.5 bar He)

HRMS *m/z* (GC-EI): calcd. for $C_{10}H_{16}$ ($[M]^+$): 136.124650; found 136.124660.

t_{R1} = 12.97 min, t_{R2} = 13.24 min; e.r. = 95:5 (90% e.e.)

The spectroscopic data and physical properties are in accordance with the literature.[35]

7.8. (–)-(*Z*)-β-Santalol 7

(*Z*)-2-methyl-5-((1*S*,2*R*,4*R*)-2-methyl-3-methylenebicyclo[2.2.1]heptan-2-yl)pent-2-en-1-ol (7)

The procedure is adopted from Clanton at al.[36]

To solution of potassium *tert*-butoxide (3.0 equiv., 444.8 mg, 3.96 mmol) in THF (15.0 mL) cooled down to −78 °C, *n*-BuLi in hexanes (2.5 M, 3.0 equiv., 1.60 mL, 3.96 mmol) was added dropwise. The resulting yellow solution was stirred for 15 min before adding dropwise 2,2,6,6-tetramethylpiperidine (3.0 equiv., 0.67 mL, 3.96 mmol). The obtained solution was further stirred for 15 min before adding solution (−)-β-santalene **6** (1.0 equiv., 270.0 mg, 1.32 mmol) in 2 mL of THF. The reaction mixture was stirred for 1 hour at −78 °C. Then triisopropylborate (3.0 equiv., 0.91 mL, 3.96 mmol) was added and the reaction was allowed to warm to rt. Aqueous hydrogen peroxide solution (35%, 6.0 equiv., 0.77 mL, 7.93 mmol) was added slowly and the reaction was stirred for an additional 1 hour. The reaction was diluted with water (70 mL) and extracted with MTBE (3x30 mL), washed with 1 M HCl (20 mL), and brine (20 mL). The organic layer was dried over anhydrous Na_2SO_4, concentrated and purified by column chromatography: starting with *n*-pentane/MTBE

(100:1) delivered (−)-β-santalene **6** (R_f = 0.95, 68.5 mg, 0.34 mmol, 25.7% recovered) and moving to *n*-pentane/EA = 4:1 to afford the pure product **7** in 48.2% (65.3% based on recovered starting material) yield (R_f = 0.45, 140.1 mg, 0.64 mmol).

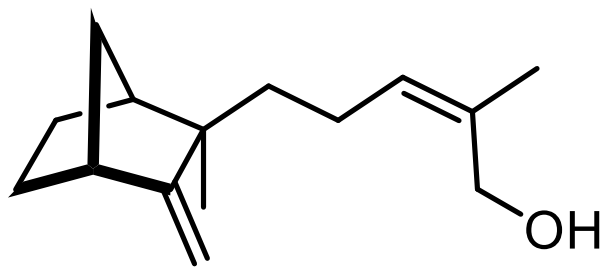

^{1}H NMR (501 MHz, $CDCl_3$) mixture of *endo*/*exo* = 92:8 and *Z/E* = 97:3. *Z*/*E* ratio confirmed via direct comparison with the synthesized **7-(*E*)** (see below). δ 5.29 (td, *J* = 7.5, 1.6 Hz, 1H), 4.73 (s, 1H), 4.45 (s, 1H), 4.14 (s, 2H), 2.66 (d, *J* = 3.7 Hz, 1H), 2.14–1.96 (m, 3H), 1.78 (q, *J* = 1.4 Hz, 3H), 1.73–1.58 (m, 3H), 1.52–1.33 (m, 2H), 1.31–1.11 (m, 3H+1H(OH)), 1.04 (s, 3H).

^{13}C NMR (126 MHz, $CDCl_3$) δ 166.2, 134.6, 128.9, 99.8, 61.7, 47.3, 45.12, 45.07, 41.9, 37.4, 30.1, 24.0, 23.6, 22.8, 21.3.

HRMS *m/z* (API-MS): calcd. for $C_{15}H_{24}O_1Na_1$ ($[M+Na]^+$): 243.171934; found 243.171850.

HPLC (Chiralpak IG-3, *n*-heptane/2-propanol = 99.5:0.5, 294 K, 210 nm)

t_{R1} = 15.31 min (minor diastereomer, not baseline separation), t_{R2} = 18.48 min (major), t_{R3} = 20.81 min (major); e.r. (major) = 99:1 (98% e.e.)

$[\alpha]_D^{25}$ = –104.47 (c = 0.52, $CHCl_3$)

The spectroscopic data and physical properties are in accordance with the literature.[37–39]

7.9. (–)-(*E*)-β-Santalol 7-(*E*)

(*E*)-2-methyl-5-((1*S*,2*R*,4*R*)-2-methyl-3-methylenebicyclo[2.2.1]heptan-2-yl)pent-2-en-1-ol (7-(*E*))

To a vigorously stirred solution of (−)-β-santalene **6** (1.0 equiv., 30.0 mg, 0.147 mmol) in DCM (1.5 mL) at rt was added *t*-BuOOH (1.0 equiv., ~5.5 M in decane, 29.0 µL, 0.147 mmol) followed by the addition of SeO_2 (1.0 equiv., 16.2 mg, 0.147 mmol). The resulting mixture was stirred for 3 h and then diluted with DCM (5 mL), washed with 10% NaOH (aq.) (10 mL) and water (10 mL). The organic phase was dried over anhydrous Na_2SO_4 and concentrated. The obtained crude was dissolved in EtOH (3 mL) and excess of $NaBH_4$ (~2 equiv., 12.0 mg) was added. The resulting mixture was stirred for 3 h. The reaction mixture was then diluted with Et_2O (5 mL), washed with H_2O (3x10 mL) and saturated aqueous NaCl (10 mL), dried over anhydrous $MgSO_4$ and concentrated under reduced pressure to give crude alcohol which was purified by column chromatography *n*-pentane/EA (5:1, R_f = 0.40) to afford pure **7-(*E*)** in 31.2% yield (10.1 mg, 0.045 mmol).

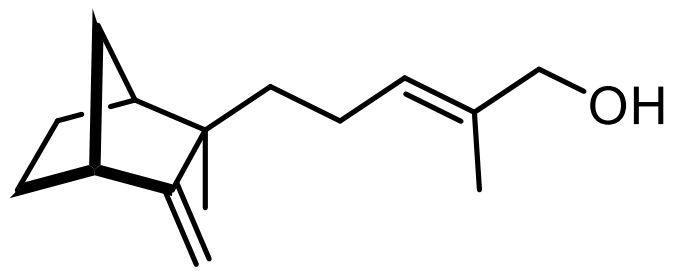

^{1}H NMR (501 MHz, $CDCl_3$) δ 5.39 (tt, *J* = 5.8, 1.4 Hz, 1H), 4.73 (s, 1H), 4.46 (s, 1H), 3.99 (s, 2H), 2.66 (d, *J* = 4.6 Hz, 1H), 2.12 (d, *J* = 4.1 Hz, 1H), 2.02 (dtt, *J* = 19.8, 13.4, 7.4 Hz, 2H), 1.72–1.61 (m, 3H+3H), 1.48–1.37 (m, 3H), 1.28–1.16 (m, 3H), 1.05 (s, 3H).

^{13}C NMR (126 MHz, $CDCl_3$) δ 166.4, 134.6, 127.1, 99.8, 69.2, 46.9, 44.9, 44.7, 40.9, 37.2, 29.9, 29.8, 23.9, 23.4, 22.8.

The spectroscopic data and physical properties are in accordance with the literature.[40,41]

8. Computational Studies

8.1. Methods

Possible TS conformations were explored by the artificial force induced reaction (AFIR) method[42] implemented in the global route reaction mapping (GRRM)[43] program. An extensive conformational search has been performed on possible catalyst substrate orientations at AIMNet2 (model: aimnet2_wb97m_0.jpt)[44] via Orca 6.1.1,[45] using SC-AFIR with constraint. The obtained approximately 1000 structures were sorted by energy. The energies of the lowest 200 structures were re-evaluated at g-xTB.[46] The lowest ten geometries were then further optimized at r^2SCAN-3c theory,[47] and the most stable conformers were further optimized at their transition states. All geometries were optimized at the r^2SCAN-3c level of theory using Orca 6.1.1 program. Thermal free energy corrections have been performed at the same level of theory, and the temperature was set at 223.15 K. Transition state structures were verified by the corresponding intrinsic reaction coordinates. Solvation effect has been accounted by using SMD (Chloroform) solvation model. All single point energy is calculated at SMD(Chloroform)-ωB97M-V/def2-TZVPP level of theory.[48,49] RI approximation was used with RIJCOSX with def2/J as an auxiliary basis set. The visualizations of the molecular geometries were generated using the ChimeraX[50] version 1.11 followed by rendering with Blender[51] version 4.3. Conversion of enantiomeric ratio and ΔΔG was performed based on the Boltzmann distribution as follows: $\Delta\Delta \mathrm{G} = \mathrm{RT} \cdot \ln \frac{\mathrm{pdt}(R)}{\mathrm{pdt}(S)}$.[52]

8.2. Benchmark Experiment for the Computational Study

The computational study was performed on the reaction of **1b**, whose reduced size permits an extensive conformational search of the catalyst–substrate complex. The experimental reference was obtained using **1b**, diene **2** and catalyst **3f** according to GP3 in $CHCl_3$ at −50 °C. Under these conditions **4b** was obtained in 19% yield with 84:16 *endo*/*exo* ratio and 95:5 e.r.

8.3. Results and Discussion

The calculated (DFT) energy diagram is shown in Figure S12. Intermediates **I** and **II** correspond to the pre- and post-reaction complex, respectively, connected by the IRC pathway from each transition state. The calculated results at SMD($CHCl_3$)-ωB97M-V/def2-TZVPP//r^2SCAN-3c level of theory suggest the reaction proceeds in asynchronously concerted manner, regardless of the enantiomers. The calculated energy difference between **TS1** and **TS1′** agreed well with the experimental one (1.4 kcal/mol (*calc*) vs. 1.3 kcal/mol (*exp*)).

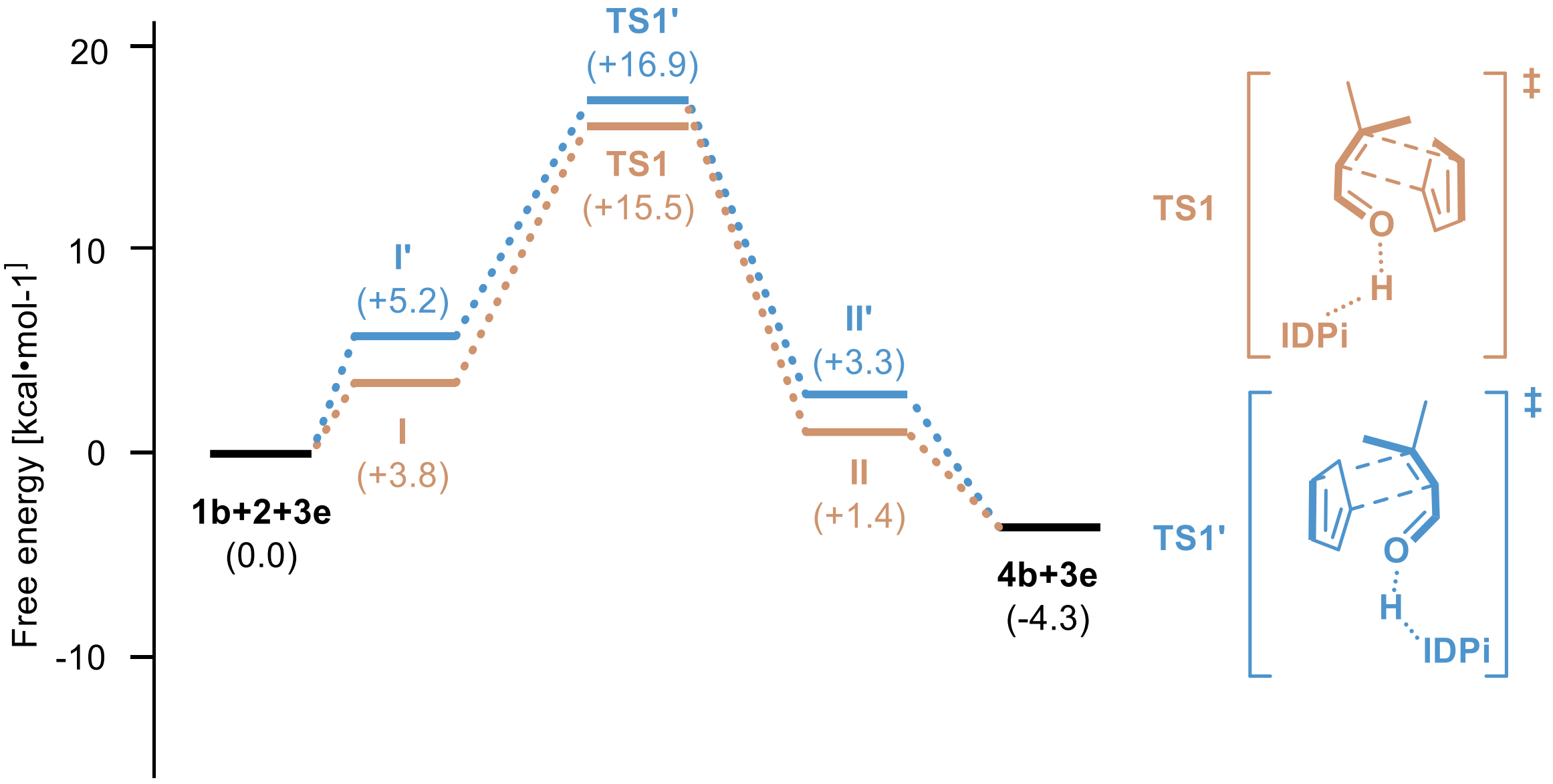

Figure S12. The calculated energy diagram of the proposed catalytic cycle.

For a better understanding of the origin of enantioselectivity, distortion-interaction analysis was performed following the Houk-Bickelhaupt protocol[53] (Table S4). Gas-phase single point energies of the optimized **TS1** and **TS1′**, leading to the major enantiomer and minor enantiomer, respectively, were calculated at the ωB97M-V/def2-TZVPP level of theory. Then they were decomposed into the catalyst fragment and the substrate fragment. The results suggest that the distortion of the catalyst would be the major factor in controlling the enantioselectivity. An overlay of the anionic fragments of **TS1** and **TS1′** illustrates that the BINOL substituents are more expanded in **TS1′** to accommodate the substrates, thereby leading to increased distortion of the anionic fragment (Figure S13).

Table S4. Summary of the distortion-interaction analysis of **TS1** and **TS1′**

**TS**	**ωB97M-V/def2-TZVPP (in hartree)**	**Relative energy ΔΔE (kcal/mol)**
**TS1**	-7258.521884	-1.19
**TS1′**	-7258.519992	
Substrate fragment		
subst- **TS1**	-464.9605533	+0.27
subst- **TS1′**	-464.9609877	
Catalyst fragment		
cat- **TS1**	-6793.380704	-2.82
cat- **TS1′**	-6793.376212	
Total distortion		-2.55
Total interaction		+1.36

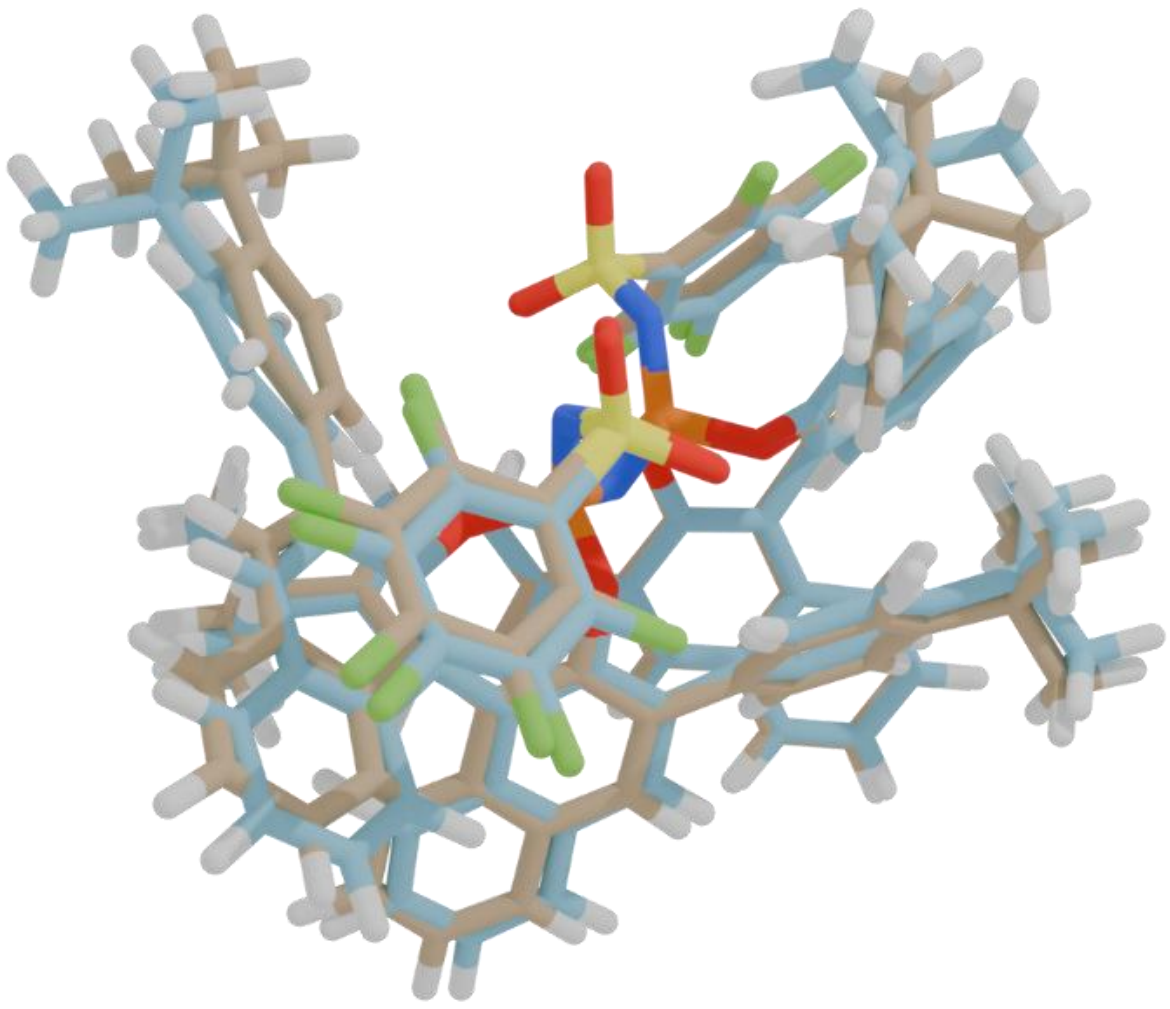

Figure S13. Overlay of the anionic fragments of **TS1** (beige) and **TS1′** (light blue).

Table S5. Energy table of the optimized structures. Energies are given in Hartree. Computed single point energies (E), Gibbs free energy corrections (Gcorr), Gibbs free energies (G), and negative imaginary frequencies for transition states are provided. Small imaginary frequencies are associated with remote, low-amplitude floppy motions of peripheral groups and are not involved in the bond-making/bond-breaking process.

Structures	E (solv) in Hartree	Gcorr in Hartree	G (solv) in Hartree	Imaginary frequency
**1b**	-270.5432118	0.09524496	-270.4479668	-
**2**	-194.091694	0.07305413	-194.0186398	-
**3f**	-6793.955497	1.37396706	-6792.58153	-
**I**	-7258.620148	1.57812596	-7257.042022	-
**I′**	-7258.618228	1.57842383	-7257.039805	-
**TS1**	-7258.605167	1.5816995	-7257.023467	-216.22
**TS1′**	-7258.604132	1.58288881	-7257.021243	-224.30
**II**	-7258.631361	1.58539585	-7257.045965	-
**II′**	-7258.628747	1.58580515	-7257.042942	-
**4b**	-464.6662117	0.19290686	-464.4733048	-

8.4. Cartesian Coordinates of the Optimized Structures

1b

O	1.00391870982148	-0.23566825211198	1.24127743342751
C	-0.10249890101257	-0.21855399266762	0.72961302539149
H	-0.48416411269518	0.71916344263561	0.27036055223645
C	-0.97375822354237	-1.38628627426766	0.68387099200096
H	-0.55231344840655	-2.27928038515141	1.14243435046583
C	-2.20728811164660	-1.43853994917361	0.14038802238895
C	-2.91238501672379	-0.29246028402783	-0.52265285847814
H	-3.15670830032816	-0.55927983124749	-1.55848010901150
H	-3.86861888169900	-0.10518444749982	-0.01831454965685
H	-2.34359013448673	0.63653321535737	-0.53300239500330
C	-2.99352809822490	-2.71464496205656	0.16580821534021
H	-2.44629959066717	-3.52672134809898	0.64907507804928

H	-3.94376070452776	-2.56707439460497	0.69494369557406
H	-3.25270098586069	-3.02348509708503	-0.85509515272496

2

C	-1.75197763501567	-0.89306661949500	0.00684673426272
H	-2.03773256146522	-1.26644576085007	1.00192819451462
H	-1.36565338690487	-1.76491791858329	-0.54277171479941
C	-2.90659680010802	-0.25143275418260	-0.70242412846920
H	-3.83279729558917	-0.75997962480638	-0.94044508164711
C	-2.59168397651752	1.02867211319119	-0.98086401470263
H	-3.22530468799901	1.74720737104622	-1.48902033091870
C	-0.73733602562955	0.20784119958944	0.09053647214199
H	0.23849760665363	0.10163420221071	0.54834225491170
C	-1.24411443845027	1.31403481676832	-0.48818466302326
H	-0.74508164897431	2.27213882511144	-0.58279855227072

3f

O	1.78555113208700	2.82279565375440	-1.47756325027522
O	0.62226234628474	1.75443835893182	0.48316215001226
O	2.62681020985059	-2.19570124487376	-0.41661549858940
O	3.03018057737743	-1.31359505122166	-2.76071587818651
S	-0.18036483278324	-3.18946646135017	-0.96465287378139
S	-0.68428187955204	1.13894245235072	-3.39468828271341
O	-0.12153747938299	-2.82134314701325	0.44398939996097
O	-1.45067043713914	-3.41806124668490	-1.64112738182847
O	-0.21651250088864	2.19099997644639	-4.30470850605411
O	-0.30767757732995	-0.24930529576805	-3.76603957861509
N	0.61501387072068	-2.03105998589689	-1.91768599370113
N	-0.43223960752490	1.44883098117173	-1.84227950579486
P	2.01279291703206	-1.21041400522847	-1.52221516523953
N	1.96432142323483	0.27286024501529	-1.09451209642983
P	0.94870184354341	1.49376114203338	-1.07281516100453
C	4.51883231504976	2.13983209676797	-2.14168274586039

C	5.50085455947447	1.14323175739192	-2.07248232069204
C	5.97529366917295	0.52573016961510	-3.21789784715353
C	5.49301908483801	0.86765430795868	-4.48751715615774
C	4.48031716114882	1.82910348176931	-4.54513050994771
C	3.99526707522340	2.45243027606182	-3.39845465443492
H	5.88835987003326	0.83989397876462	-1.10303021186431
H	6.74459583108181	-0.23381340676559	-3.11055672519289
H	4.05727257806923	2.12113450387517	-5.50063360076020
H	3.22266038166407	3.20789472843714	-3.49390914818837
C	-2.22015196185195	1.98359833957645	0.91175053031873
C	-1.89751823201238	0.67308662258262	1.29415890537407
C	-2.81504804234003	-0.35502889055174	1.14537735813110
C	-4.09692348797115	-0.13548148313582	0.62287131686583
C	-4.41417429384773	1.17135901626262	0.24918333728808
C	-3.49511804688124	2.20619540868397	0.38343432503189
H	-0.92146266268383	0.44813241075482	1.71036667389669
H	-2.51162466851212	-1.35556211725886	1.44072745200582
H	-5.39085843333207	1.40233220582484	-0.16389393514424
H	-3.76696764118010	3.20187775152736	0.04212452067090
C	2.15698736104068	-2.39227468488949	-5.28350936515545
C	2.25283312847257	-1.01197053063734	-5.50266199744947
C	1.41476432366765	-0.37776851693141	-6.40494224291625
C	0.44340183777829	-1.07800562555501	-7.12866653664518
C	0.35637075202002	-2.45494282301813	-6.91090605082257
C	1.19363539548088	-3.09965620636486	-6.01018674899240
H	2.98364849986488	-0.42056230300558	-4.96261819791075
H	1.50431878319778	0.69926878108525	-6.51437688183232
H	-0.38431487305088	-3.04803281654147	-7.43548068803088
H	1.07271562255320	-4.16648681334978	-5.84771133268854
C	3.35589764354490	-0.69051836418581	1.92714829130501
C	2.23823073911423	-1.40670837775878	2.35979134609048
C	1.35236617340659	-0.85564822445456	3.28005689350578
C	1.54501714064565	0.42336917565601	3.80639717688909

C	2.65581314461152	1.14332419898213	3.34774888547718
C	3.53988134880899	0.60469223787254	2.42813568416248
H	2.05455171574691	-2.40898641326049	1.99222062665018
H	0.50445438845924	-1.45649360309982	3.59090410183179
H	2.84116642597092	2.15122092046775	3.70785409263491
H	4.37261981120250	1.20652097512874	2.07348113677162
C	0.10249415198054	3.01355583821722	0.79829435997794
C	2.79649038281698	3.18963889403223	-0.58431343650109
C	3.99484845395106	-2.00139417080706	-0.15252289655807
C	3.49778682661788	-2.58108084453828	-3.12682985541468
C	-1.28911617781473	3.12332511449119	1.04690703600847
C	3.06066944294763	-3.11662681472730	-4.36646964001944
C	4.13658736142757	2.86061575719458	-0.90975331252714
C	4.35376538534777	-1.26139411558894	1.00001373021392
C	0.71924547277941	7.78240541135686	1.31442929448094
C	1.25992767327135	6.54961818489073	1.03957015544998
C	0.46765341712838	5.37860170856689	1.11180548234729
C	-0.92093085263152	5.51178184800097	1.42360634275573
C	-1.44068606880474	6.79560176668650	1.72149364002042
C	-0.63861172113796	7.90767771965281	1.67467482102633
H	1.34168744460505	8.66975980996942	1.24651572216236
H	2.30366062515894	6.46629357632736	0.75535692435153
C	0.98482345419981	4.07505815281172	0.84832301262222
C	-1.75529967866802	4.37572829533212	1.39643104391287
H	-2.49508150046080	6.88439127756985	1.97062725727821
H	-2.80521708381007	4.49832204589527	1.64867814720339
C	5.49421381666964	4.74172486469095	3.37195656880954
C	5.80794731390922	4.18414706196400	2.15809172433062
C	4.79538150751037	3.86728123322089	1.21881553654576
C	3.43302909276155	4.16479744374369	1.53368240076941
C	3.14302777817185	4.72365551152661	2.80280301800279
C	4.14733478065746	5.00370117268075	3.69792513688302
H	6.15054272136273	3.02904777820703	-0.23153079588329

H	6.84122230895551	3.96425391369576	1.90103428212155
C	5.10786399751381	3.23302794623632	-0.00130158517373
C	2.42225613895497	3.83182871225612	0.58144477225945
H	2.11174652656198	4.92873266566039	3.06771240308766
H	3.90036924343730	5.42827110651277	4.66666398743206
C	8.59652182226553	-2.36208332881501	-1.57951927356368
C	7.27181101018828	-2.65181923695725	-1.80198773697835
C	6.27797243345993	-2.26641296527364	-0.86987752161597
C	6.67920186794577	-1.53861082014402	0.29463840875241
C	8.05570327389824	-1.27263917460570	0.50008378894188
C	8.99588950785174	-1.67585629862522	-0.41421371411150
H	9.34192266432547	-2.66155440996503	-2.31037388878869
H	6.97762186608792	-3.17595143544381	-2.70462981458652
C	4.88540573541301	-2.51444564961998	-1.06987628908873
C	5.70606177652613	-1.06743769418033	1.20169909175340
H	8.35136011862741	-0.72903992268354	1.39380431334953
H	6.03402435905954	-0.52720243772147	2.08611708552064
C	5.54570434946193	-7.16597645093509	-3.29333640441135
C	4.78770880659616	-6.42758695409447	-4.16646573812404
C	4.36918554135588	-5.11509669975957	-3.83389200395579
C	4.78540509542654	-4.54880534420584	-2.58932208466226
C	5.54392334696110	-5.34695780874807	-1.69896399578060
C	5.91002902648758	-6.62631316377417	-2.04196280281540
H	3.27158559922302	-4.79925546778704	-5.65885587415856
H	4.47688410284941	-6.84611661071091	-5.12005896292812
H	5.82622198865033	-4.93966647086304	-0.73324388934307
H	6.47983082171930	-7.22909689599395	-1.34119480204275
C	3.54110427950392	-4.37000333969780	-4.69786403549382
C	4.38257916923228	-3.21705795260193	-2.27561263060718
H	-1.05032178469201	8.88784032123187	1.89619031991767
H	6.27840960357358	4.97421137991997	4.08626676894085
H	10.04696360467030	-1.45923269030960	-0.24861981688697
H	5.84730305131439	-8.17627259161511	-3.55275394135823

C	-5.07629431520131	-1.30075765516980	0.48410693797052
C	-6.42455861750280	-0.86465531513896	-0.10121763092081
H	-6.31782291861073	-0.45049289528696	-1.10995179293760
H	-7.08590832724787	-1.73415179302412	-0.17494904227712
H	-6.92260549282618	-0.12014230456707	0.52965009032233
C	-5.33155797674259	-1.91267035292813	1.87565041866214
H	-5.75486203162092	-1.16589275072023	2.55626081834512
H	-6.03795759251239	-2.74709272835131	1.79610642226095
H	-4.40944270011571	-2.29487172097420	2.32372486525753
C	-4.46660972423562	-2.36950500490348	-0.43970691887839
H	-4.25149824724759	-1.95632575554244	-1.43132214282830
H	-3.52931706140759	-2.76848180822832	-0.04374080904037
H	-5.16522236737468	-3.20531801803591	-0.56282397556419
C	0.62728423959077	1.03724282728896	4.86604689188818
C	-0.53354031417319	0.10949878247797	5.24377070067604
C	0.03340838497526	2.35720845333715	4.34362372245900
C	1.45897080962799	1.31793113682981	6.13277064404978
H	-0.17757602055572	-0.83536352114925	5.66883883533177
H	-1.15781378681702	0.59964088423413	5.99825870105907
H	-1.17212690849837	-0.11233917000886	4.38130177591250
H	-0.61009729936943	2.17641546827493	3.47791752842401
H	-0.57205377195013	2.83409577529780	5.12324587912321
H	0.81198640330678	3.06347586347424	4.03851539182615
H	1.90655589590307	0.39418295035425	6.51533184951066
H	2.26846265982903	2.02723215178101	5.93289299108020
H	0.82158869601838	1.74398829143137	6.91633573300824
C	-0.50574492581685	-0.33052065493328	-8.06458076995561
C	-1.37060214945727	-1.28289842288356	-8.89942161202660
C	0.29878859598658	0.55727586579793	-9.03195585081906
C	-1.43585525354325	0.55259765811731	-7.20972498753592
H	-2.03607246474173	-1.88547289424849	-8.27228887871536
H	-2.00184808698011	-0.69921967865648	-9.57768098127335
H	-0.75894303521044	-1.95820268673626	-9.50846708921024

H	0.98482952662271	-0.04673736170687	-9.63603745673260
H	-0.38251770877838	1.08484726457884	-9.70911737793303
H	0.88697443078295	1.31152511961799	-8.50189753110455
H	-2.00505069683236	-0.06695922708879	-6.50715071026596
H	-0.87372090062714	1.28679470315385	-6.62603381198720
H	-2.14361645372517	1.08870322838610	-7.85358887287099
C	6.12133214337163	0.23887984570178	-5.73433143827271
C	6.07949185482066	-1.29757602439908	-5.64212725281300
C	7.59085093745302	0.69932151076599	-5.81421493640477
C	5.41015004794145	0.66095811251459	-7.02579991145923
H	5.04770254587866	-1.66028901573753	-5.61757106042472
H	6.57272870965544	-1.73819937643368	-6.51621725774669
H	6.59293264881294	-1.66591571990622	-4.74848937172607
H	8.16061542985525	0.37042957531900	-4.93897718013547
H	8.06978096255012	0.28390685235108	-6.70877401902029
H	7.65217113124171	1.79178185403148	-5.86500565852968
H	4.35623812396248	0.36199455396146	-7.02413397398062
H	5.46859197398570	1.74285178672221	-7.18629004829790
H	5.89088027554464	0.17401691318989	-7.88079107801426
C	0.85626159296946	-4.65071498704826	-1.21388087784761
C	1.60270937317667	-5.24643982194629	-0.19451840751372
C	0.85441466576664	-5.25912363223037	-2.47193125355602
C	2.29814985016124	-6.42890378944231	-0.42256092905673
C	1.54184557093728	-6.44015306608161	-2.70453781720039
C	2.25239511198766	-7.03631739626147	-1.67008262432511
F	1.68094993520732	-4.73029389504264	1.02789372713617
F	2.99936036424543	-6.99218654271582	0.56079677150822
F	2.88357034426061	-8.18634336599554	-1.87097070468784
F	1.51782103517266	-7.00536640369621	-3.91143194819047
F	0.19127699041512	-4.71629961376737	-3.48842773595920
C	-2.49920823206991	1.20760760653889	-3.36864738138661
C	-3.31612059221999	0.11307172621949	-3.64849119217021
C	-3.10939434610018	2.41584665037220	-3.02866149655563

C	-4.70230981707005	0.22748184913338	-3.59846628142250
F	-2.82760022128484	-1.08266730752971	-3.97057216386572
C	-4.48878929999663	2.53622064900211	-2.97017574729966
F	-2.38292468713579	3.48843005981384	-2.72479128862975
C	-5.28732048916829	1.43750368376072	-3.25779563113644
F	-5.47347889456618	-0.82811423559537	-3.87060383535454
F	-5.05450623632506	3.68991126060661	-2.60524891726580
F	-6.61392933103209	1.54147049080034	-3.18733183599619
H	0.02142863811152	-1.56154348882888	-2.63192987539656

I

O	1.568165828948	2.489886350462	-2.056806767609
O	0.487926650502	1.623815519255	0.055213371286
O	2.327856132066	-2.366702541970	-0.446670456779
O	2.501932902200	-1.855873491694	-2.916685984956
S	-0.470795438047	-3.405026540428	-0.737122073621
S	-0.928729078282	2.060912972891	-3.643082001681
O	-0.384175949223	-2.941899175429	0.654139437308
O	-1.795718116305	-3.852950992963	-1.211084960163
O	0.099306471438	1.913432067201	-4.674624893289
O	-2.325177549235	1.739438943339	-4.082595069038
N	0.122794351224	-2.371765336071	-1.826003538534
N	-0.696344781382	1.265934571964	-2.278330158945
P	1.525700579923	-1.617826174857	-1.642646373976
N	1.587704957768	-0.060863764952	-1.462269499686
P	0.716052646587	1.228315130417	-1.497535656170
C	4.256127947367	1.771948964519	-2.755236586176
C	5.239824351680	0.784795717990	-2.618509462332
C	5.635480509764	0.015814190632	-3.700738504013
C	5.070746855017	0.192928705143	-4.970223966040
C	4.062161297409	1.152241683766	-5.089793834846
C	3.656468950331	1.926882299638	-4.007294967444
H	5.685291660675	0.601648165352	-1.644283291530

H	6.404330736857	-0.735241898911	-3.541978822178
H	3.575781883194	1.320798528176	-6.044805066827
H	2.876253974026	2.664448075231	-4.153462604001
C	-2.201262193156	1.785875786910	0.993128673231
C	-1.665150324898	0.635394485143	1.589483207413
C	-2.475583529894	-0.436027331805	1.924841789128
C	-3.855119272341	-0.420014155323	1.689683348411
C	-4.378765475872	0.711206345958	1.057474987498
C	-3.571159402308	1.791722539380	0.715108628219
H	-0.607107030024	0.577728014794	1.815578088434
H	-2.008759194483	-1.302460359032	2.383055558547
H	-5.437237761415	0.770784813184	0.826218379230
H	-4.014588968528	2.647120086774	0.213722574270
C	1.603215667839	-3.158560138138	-5.308696180954
C	1.675488755234	-1.791294726557	-5.610399672403
C	0.875228510022	-1.233917814411	-6.596790050920
C	-0.032513021891	-2.001189062479	-7.334011974251
C	-0.101818489075	-3.364322010802	-7.029333680267
C	0.689269160098	-3.929179196286	-6.036821859830
H	2.370256630372	-1.147569726949	-5.084153016567
H	0.966349727982	-0.167593772770	-6.776779729266
H	-0.800361784945	-4.006752885348	-7.555641480718
H	0.575121251735	-4.985210213352	-5.806251750753
C	3.266817110117	-0.578717355049	1.599248833492
C	2.196387607569	-1.220018266315	2.227257571782
C	1.439301799810	-0.558342790530	3.189399793648
C	1.706838507995	0.762987274557	3.555616682917
C	2.757824225957	1.408821076107	2.892205101656
C	3.519371482941	0.757049947056	1.936248805981
H	1.950944004534	-2.246481936817	1.981471395796
H	0.629566381921	-1.106103855786	3.659883645455
H	2.995890634418	2.444148128205	3.119488384448
H	4.312599218287	1.299264185403	1.428165298973

C	-0.025579512372	2.891231697401	0.305379784027
C	2.597033102032	2.977798190290	-1.256427511092
C	3.698219327875	-2.151386958125	-0.351569193913
C	2.973266935344	-3.136021008094	-3.165986793136
C	-1.374135095174	2.984328664266	0.737626541500
C	2.506800003214	-3.801701558419	-4.331448507909
C	3.928108424390	2.634794409458	-1.601053341906
C	4.168335746935	-1.273203851911	0.657438484835
C	0.423322525259	7.700468858138	0.154910775832
C	0.990281567253	6.459925985187	-0.009670480066
C	0.261704105918	5.284353145541	0.294759920300
C	-1.093830872708	5.411740394111	0.733119365744
C	-1.637879173672	6.707430765179	0.915051717619
C	-0.896330556635	7.827769263960	0.637315085221
H	0.992480124657	8.590394753385	-0.096745157604
H	2.005071338306	6.369830337506	-0.384355465371
C	0.813747144738	3.978230639683	0.143621929316
C	-1.867195589606	4.254246910899	0.965040549740
H	-2.665121715127	6.795983504968	1.258714785615
H	-2.879994140724	4.371887352915	1.341634995300
C	5.405873613534	5.061127003915	2.353878764588
C	5.682838700090	4.356852183452	1.209173647346
C	4.642118018621	3.909709809248	0.358329246344
C	3.286563735412	4.225715614725	0.689504808576
C	3.035614913131	4.939409473899	1.886743641382
C	4.068435009096	5.345506478620	2.697504445434
H	5.961574219464	2.914229746973	-1.026482501201
H	6.709280356202	4.118659711032	0.941612698707
C	4.924009834771	3.131000203822	-0.784532181095
C	2.246929685469	3.752935121757	-0.169341265769
H	2.011991037929	5.160239790709	2.167627674147
H	3.850702881747	5.886127956063	3.614053745546
C	8.176967126281	-2.805749282726	-2.065570208835

C	6.835556451030	-3.096226964369	-2.139233839015
C	5.920932604848	-2.564663217839	-1.197161137236
C	6.425114375468	-1.692220199850	-0.182329719479
C	7.815659820301	-1.426863508478	-0.125827808158
C	8.676726486584	-1.971323997543	-1.045085993216
H	8.856800515002	-3.220207165211	-2.804380505560
H	6.467637665690	-3.733284019825	-2.935279328145
C	4.513046693867	-2.809973494203	-1.249033115942
C	5.533451994425	-1.082271856884	0.725298384282
H	8.186065064576	-0.769408179885	0.656846485757
H	5.938702611532	-0.441742484462	1.504724079596
C	5.097982749213	-7.686304065978	-2.951406862493
C	4.286199815114	-7.055212780241	-3.859725803253
C	3.862010230662	-5.719786998727	-3.649248869115
C	4.324962982413	-5.018802896481	-2.493571713241
C	5.140382366380	-5.706683022833	-1.562627997904
C	5.513128236558	-7.010370409401	-1.784977476257
H	2.706927959126	-5.604855500991	-5.459066257524
H	3.935300588758	-7.578111164188	-4.745574659430
H	5.459830331994	-5.193889891633	-0.660814738651
H	6.124949126851	-7.527833509091	-1.052096413019
C	2.992899921983	-5.075971860200	-4.554114376304
C	3.919531777267	-3.664485749242	-2.305761679191
H	-1.330352402954	8.814749027714	0.765939802228
H	6.212646240470	5.392111449688	3.001027655411
H	9.739555331514	-1.754156938103	-0.995143635320
H	5.402290924691	-8.715747249048	-3.114941075203
C	-4.720867119987	-1.579531964885	2.184344742696
C	-6.178960897456	-1.446631381266	1.730058823802
H	-6.252588797551	-1.395028636523	0.637641945045
H	-6.749330959452	-2.319242458009	2.066255281026
H	-6.658850211571	-0.555700727083	2.149423810652
C	-4.690390307592	-1.576791131875	3.726414424222

H	-5.058257997075	-0.623905402104	4.122283128286
H	-5.320560864556	-2.383326444286	4.119908405716
H	-3.672995572424	-1.727760110234	4.100503696700
C	-4.168661192631	-2.921899437678	1.673775953553
H	-4.175206623271	-2.955871798734	0.581575942653
H	-3.138115152346	-3.097261797480	1.993273482383
H	-4.784248862462	-3.747234487775	2.050527305898
C	0.939084609177	1.496495867718	4.658039111588
C	-0.223815171851	0.665577928690	5.213304086736
C	0.369655911756	2.821151178512	4.117571770850
C	1.916953330202	1.801599925790	5.810285011854
H	0.124674753090	-0.267873085898	5.668346279420
H	-0.742988318953	1.238284362215	5.989288854548
H	-0.954993158384	0.424261110268	4.433713132882
H	-0.330660697774	2.642759666509	3.295615425945
H	-0.166379495705	3.352334024822	4.912773539081
H	1.162327219317	3.479788672857	3.749946375726
H	1.394178970646	2.315126595050	6.625960882110
H	2.350325336560	0.876391988431	6.205249091034
H	2.738527995185	2.443176307328	5.475410135139
C	-0.854429769830	-1.377006256341	-8.462177297998
C	-2.129964996817	-2.183575956480	-8.749751702684
C	0.021969999764	-1.358845874659	-9.730915435890
C	-1.264506858937	0.065242945538	-8.117611722264
H	-2.738524958609	-2.296789122540	-7.845673116505
H	-2.729587632230	-1.662948513469	-9.504117532158
H	-1.907477999457	-3.179124206965	-9.146791863076
H	0.319679559509	-2.374708713314	-10.012889013613
H	0.932382540855	-0.772558639836	-9.565917381398
H	-0.528726016402	-0.913609289751	-10.568458326241
H	-1.790823082559	0.109455873358	-7.159174244858
H	-0.405821451923	0.739480440859	-8.058677758427
H	-1.929112942625	0.454020034888	-8.896973647570

C	5.603091756417	-0.610658968800	-6.159203831990
C	5.516115192259	-2.119711365281	-5.868392069084
C	7.079456822538	-0.225119269899	-6.380386387268
C	4.828504527933	-0.325879035758	-7.451711261104
H	4.476863577077	-2.434913316616	-5.734490790412
H	5.937603825667	-2.687598909737	-6.706117543485
H	6.072015640639	-2.391048986987	-4.965859847191
H	7.689580832346	-0.462672061693	-5.502845244746
H	7.488956564293	-0.771863506743	-7.238069654286
H	7.174571443562	0.848373383239	-6.576792269348
H	3.768471234873	-0.585157602808	-7.353266811880
H	4.906687214134	0.725585551277	-7.748929697198
H	5.243214496713	-0.931223756512	-8.264871347790
C	0.571347582305	-4.906987019509	-0.878217496458
C	1.382039205624	-5.384112112089	0.150791586559
C	0.497615855305	-5.665045637258	-2.047338316943
C	2.060146563636	-6.593314013641	0.029801728640
C	1.162107808485	-6.874884064488	-2.176430587183
C	1.933891630197	-7.350932614704	-1.124758705636
F	1.541731769224	-4.725634472556	1.298058863240
F	2.827306977090	-7.035447108857	1.029779258989
F	2.552199973916	-8.525375453078	-1.225062234779
F	1.060822499804	-7.585205964055	-3.304307428266
F	-0.227525165886	-5.252725736430	-3.091030093611
C	-1.026114357277	3.834631829938	-3.214467078609
C	-2.149793065426	4.305418200321	-2.533940635123
C	-0.063680149968	4.768705966716	-3.598489342679
C	-2.342930587908	5.655812657183	-2.288645869701
F	-3.094556113084	3.466185900664	-2.096891547471
C	-0.238527853821	6.124843242812	-3.341680631838
F	1.049735993316	4.421211121860	-4.239154893377
C	-1.387602012879	6.572782475595	-2.706286235567
F	-3.442336723546	6.074577814731	-1.657326218542

F	0.690624341019	7.003619741672	-3.722219542027
F	-1.571897114495	7.873422091656	-2.499172823317
O	-3.994201172882	0.480728434694	-2.666643594154
H	-3.303841050010	1.105751404141	-3.100186721944
C	-3.411530888037	-0.606037179826	-2.286829901580
H	-2.321672567991	-0.597200547745	-2.206223661184
C	-4.109681353536	-1.768543932545	-1.916723601509
H	-3.474065744454	-2.561666430178	-1.536740985087
C	-5.438980532650	-2.014209101982	-2.114115157886
C	-6.403918078919	-1.006845361250	-2.643748574882
H	-7.382961297104	-1.454006420273	-2.830995929082
H	-6.519723166604	-0.183094100434	-1.928030112833
H	-6.024173306305	-0.546841338806	-3.561518159882
C	-5.994814739981	-3.350486990659	-1.757357884776
H	-5.223704992514	-4.051377660556	-1.431625266890
H	-6.740233609473	-3.239743944772	-0.956702045077
H	-6.530470927171	-3.770372118737	-2.617166959230
C	-2.601684165443	-3.282390870332	-4.414623970739
H	-2.393897990844	-3.556599228429	-3.370280486071
H	-1.901711341644	-3.893921642441	-5.005070566388
C	-4.015171841494	-3.507085646387	-4.838191320993
H	-4.526437061909	-4.460736181740	-4.774792853178
C	-4.518970160626	-2.351128963035	-5.333352965576
H	-5.515151574476	-2.209832830335	-5.740060203155
C	-2.379995208110	-1.833295835828	-4.694943430140
H	-1.434891449292	-1.326285934844	-4.536984595229
C	-3.506514054907	-1.313403475294	-5.244886865057
H	-3.640210904145	-0.285054215480	-5.562243555310

I′

O	1.159600594318	2.601941264880	-1.883005119013
O	0.026452285438	1.384158283618	0.010593600193
O	2.448592980712	-2.119894719606	-0.400012394674

O	2.853985450807	-1.574877108364	-2.841893204985
S	-0.004965992546	-3.640036065384	-1.024436295270
S	-1.137416519312	2.102780408324	-3.746206861717
O	-0.208012687810	-3.217742876310	0.365179822206
O	-1.186327382039	-4.307358077810	-1.668969974432
O	0.037457245805	2.193629068077	-4.623051581732
O	-2.420924802526	1.732583954906	-4.368172778528
N	0.495826665104	-2.510042802815	-2.034118450526
N	-0.920337937547	1.155689204412	-2.452670395651
P	1.707318832715	-1.494872138991	-1.701493547043
N	1.446255362064	0.032875955384	-1.572347086215
P	0.408322316458	1.203978887378	-1.556239331788
C	3.977967504674	2.261493760755	-2.358308396886
C	5.112245342123	1.459956960009	-2.173908717598
C	5.714377818204	0.813978550951	-3.239532328272
C	5.216741012840	0.932756434591	-4.542772793302
C	4.061975307979	1.700276211693	-4.714886656888
C	3.448418320932	2.353183443511	-3.648580945934
H	5.517250239571	1.325051686142	-1.175054316608
H	6.593746879473	0.207868206288	-3.041681585935
H	3.620531046121	1.816105153866	-5.699310391721
H	2.560129439733	2.943942492370	-3.838981471017
C	-2.750238766157	1.124180452895	0.710612202927
C	-2.126863837551	-0.054327199251	1.136054761429
C	-2.848698508261	-1.232147837792	1.298247402936
C	-4.221960150934	-1.291263638849	1.056304565637
C	-4.834568522206	-0.119727379019	0.590348341007
C	-4.119644040147	1.055137843974	0.413063479549
H	-1.071481160055	-0.063390356222	1.377159843920
H	-2.304902121569	-2.109185491086	1.630543766710
H	-5.897922814243	-0.113961697292	0.366025021735
H	-4.630459094807	1.933397662678	0.028390802634
C	2.509240605095	-3.079532405982	-5.281899596383

C	2.358306582344	-1.738818459667	-5.653205461761
C	1.649539151653	-1.389444517057	-6.798117953661
C	1.064643031585	-2.350633508797	-7.623431072160
C	1.184458511320	-3.687645931176	-7.224821543833
C	1.879595872105	-4.044685824422	-6.082186480828
H	2.815367261571	-0.950269605702	-5.066111630259
H	1.565092539230	-0.334211220796	-7.035806588856
H	0.722766173552	-4.474320351639	-7.814411160478
H	1.926198065258	-5.091787207686	-5.797062731928
C	2.770060310103	-0.341753412248	1.831912214293
C	1.787516588113	-1.229609057586	2.278085077568
C	0.806339216696	-0.815360624798	3.172537712351
C	0.757988344923	0.495644289714	3.651990831211
C	1.725122415845	1.386382247653	3.170617587654
C	2.708181720667	0.982314315175	2.281842841618
H	1.787823735319	-2.259672155225	1.944141495299
H	0.073742700794	-1.547648914374	3.495071632144
H	1.722440191083	2.422816530604	3.494150704899
H	3.429006905652	1.709730674702	1.918105700204
C	-0.665520568278	2.541699095898	0.347378711348
C	2.041994136891	3.120192130465	-0.944155449929
C	3.733214147780	-1.674001723031	-0.104475302470
C	3.576260602770	-2.752636543783	-2.984632828441
C	-2.044661636137	2.423424546887	0.665083631004
C	3.386186966583	-3.509954943665	-4.172009237158
C	3.431354305580	2.995401543957	-1.197373976940
C	3.896297217868	-0.778976450746	0.982399800946
C	-0.836862793155	7.357525850367	0.753537631449
C	-0.105508648913	6.223776708395	0.494733331503
C	-0.689616142769	4.939763578756	0.622193136713
C	-2.072503988816	4.849424698215	0.970287811099
C	-2.789886576350	6.037781044948	1.256103125870
C	-2.185759068571	7.265098557258	1.156621448034

H	-0.376059875888	8.333722342318	0.635458028814
H	0.929407625690	6.304375162330	0.178211407424
C	0.035758756830	3.735523760820	0.378159991143
C	-2.703601281953	3.589492385203	1.005415859507
H	-3.838603750993	5.958569267511	1.530152517018
H	-3.743463537351	3.543266377664	1.316959542086
C	4.255089234300	5.305080562547	3.010505897873
C	4.709004933425	4.751933213475	1.840012810803
C	3.806765903317	4.212402124472	0.889746789944
C	2.400564018513	4.279170429576	1.143690887320
C	1.969269685950	4.834454980174	2.373649924214
C	2.871875824915	5.333926199428	3.281596883191
H	5.352889842149	3.538129451645	-0.449385293156
H	5.774069958212	4.705517629999	1.626754851814
C	4.280498020116	3.574676511928	-0.275544603526
C	1.504861947597	3.734290321355	0.170776625317
H	0.909610005519	4.859948498753	2.601530219606
H	2.514824863927	5.750669236202	4.218920911701
C	8.440630446028	-1.459407085707	-1.192315549808
C	7.196861618575	-1.983807538074	-1.451297969993
C	6.084675397494	-1.655745401223	-0.638627162995
C	6.280313197170	-0.742569550317	0.444850651783
C	7.578686774884	-0.232803369790	0.692311964696
C	8.638572280242	-0.582443966570	-0.106224514096
H	9.277203243014	-1.719306647826	-1.834431165544
H	7.058832863621	-2.649874244543	-2.295853588554
C	4.765545672894	-2.146626189984	-0.886259670293
C	5.181361464748	-0.343923467072	1.235595041011
H	7.716010536651	0.453495481299	1.524262580504
H	5.356719346118	0.321939749963	2.077011038797
C	6.435967434574	-6.838832396736	-2.347213570486
C	5.656740375822	-6.371543243652	-3.375349453702
C	4.976464139969	-5.133590678748	-3.264752895038

C	5.147801871503	-4.349035562666	-2.083550903672
C	5.937057216743	-4.871637623859	-1.030656325048
C	6.560033395059	-6.089954286294	-1.158438330864
H	4.063230758352	-5.251231177819	-5.206278563524
H	5.528525887644	-6.955681310463	-4.282892163491
H	6.035544579245	-4.302566440194	-0.111675101205
H	7.147705295089	-6.482537070366	-0.333975795426
C	4.128259995709	-4.670533727746	-4.290706545068
C	4.485061492570	-3.088412838188	-1.996546722476
H	-2.750563975461	8.169658414944	1.360983956666
H	4.957180284346	5.709709914461	3.733375486954
H	9.627508615948	-0.178978965801	0.089905965938
H	6.938254100538	-7.797474644132	-2.435398492418
C	-5.053394423698	-2.540144326278	1.354457056819
C	-4.174564909809	-3.756723187239	1.671836879646
H	-3.587349315696	-3.607594367141	2.583656780887
H	-4.809849291785	-4.634761587306	1.829159438125
H	-3.481808420200	-3.982058940978	0.853072991654
C	-5.947884543858	-2.899165467796	0.156240109385
H	-5.337318541555	-3.116671652162	-0.724240420781
H	-6.546463414444	-3.787546642761	0.388627415627
H	-6.643283440179	-2.090943231936	-0.092673438953
C	-5.942015557122	-2.239602131466	2.578239273278
H	-6.546876859507	-3.117373587491	2.835274570119
H	-5.327645991803	-1.976753459584	3.446066772712
H	-6.619860345816	-1.402842888192	2.380014524413
C	-0.256598921957	0.965237219585	4.696542083158
C	-1.289639323794	-0.117044653451	5.032801264573
C	-1.013573391643	2.205833442201	4.189326740719
C	0.508367910377	1.329829641978	5.984669527954
H	-0.820561398303	-1.006346319973	5.467621190542
H	-2.001048102307	0.273870075168	5.768262454746
H	-1.858542231463	-0.418670477273	4.145947957779

H	-1.598470911621	1.968873269604	3.295770713409
H	-1.702413535265	2.565992826845	4.962356057917
H	-0.333253131864	3.026230266224	3.939759052557
H	1.071954744323	0.467754044186	6.357617278793
H	1.216895040761	2.146335389139	5.811043282736
H	-0.193366468739	1.648249773760	6.764720991884
C	0.355285887113	-2.001187688154	-8.931467638977
C	-1.013978015212	-2.701735878634	-9.000296996780
C	1.230994583034	-2.490913984474	-10.102414609157
C	0.141484441367	-0.490441506193	-9.085786726459
H	-1.628763018009	-2.459616639589	-8.128328091108
H	-1.549210732731	-2.388528421454	-9.904078090288
H	-0.913854122325	-3.790456318175	-9.039999722968
H	1.389544052305	-3.573123813962	-10.053008584303
H	0.749773475990	-2.259147248011	-11.060201272510
H	2.212387880732	-2.004863519497	-10.081778625512
H	-0.432911377344	-0.073144650388	-8.251626367747
H	1.092272751308	0.049418444635	-9.146393884292
H	-0.409228181594	-0.290470566948	-10.010908056745
C	5.975303679869	0.279698069677	-5.700730890606
C	6.126025529268	-1.233460008607	-5.456853404948
C	7.376750243623	0.918487072524	-5.781902657895
C	5.274360875506	0.481162213571	-7.049477274606
H	5.149997264959	-1.727850428170	-5.449078131875
H	6.726201389336	-1.683978913951	-6.256146268745
H	6.620473490316	-1.446679260773	-4.503984623499
H	7.946937992554	0.749718815775	-4.862837921300
H	7.941224120868	0.486384841630	-6.616648401017
H	7.300903727195	2.000011545257	-5.937913580872
H	4.274108437172	0.035185060259	-7.055498456693
H	5.187601416744	1.542356372289	-7.306829551144
H	5.857587941945	-0.005489410781	-7.838686732626
C	1.240973630326	-4.967209627451	-1.041250621454

C	1.990126471419	-5.324024943037	0.080216394210
C	1.431587916220	-5.699490693753	-2.214511256376
C	2.868490572732	-6.401988861364	0.039839865797
C	2.301457032150	-6.777788168604	-2.262413436645
C	3.009276233928	-7.142872499587	-1.124522340985
F	1.896944783852	-4.672189635195	1.236902627221
F	3.568719464447	-6.735918842497	1.125164057949
F	3.818868012210	-8.196982765007	-1.149828942682
F	2.459766131442	-7.465899753316	-3.395666478391
F	0.775863639741	-5.387609143700	-3.332778692467
C	-1.463070317335	3.782497514802	-3.085212186047
C	-2.694254776028	4.032163242371	-2.478076684459
C	-0.600453820259	4.863209595076	-3.263786837798
C	-3.082070967832	5.311433254404	-2.110956093737
F	-3.560240471335	3.041901984445	-2.234130631687
C	-0.972292697360	6.149534739641	-2.885176577574
F	0.605254353324	4.728335394269	-3.813132995871
C	-2.222419035612	6.379370212495	-2.330275026357
F	-4.279013312875	5.519168603915	-1.552865461210
F	-0.131687336238	7.171737682410	-3.064916102946
F	-2.591580680103	7.616338383431	-2.004269621248
O	-3.264507416218	-3.000674617045	-2.207786392413
H	-2.431399908608	-3.549579524030	-1.933563670992
C	-2.925607967805	-1.774007747094	-2.403712264817
H	-1.903959723691	-1.475772646171	-2.152147686170
C	-3.796844850691	-0.781566176098	-2.883593287704
H	-3.353245250768	0.208776123554	-2.898833628800
C	-5.046401048038	-0.936700461132	-3.415610710159
C	-5.830484893840	-2.203348155182	-3.486458073610
H	-5.218440095258	-3.098582175862	-3.407313668377
H	-6.550559798672	-2.208420832211	-2.655293657452
H	-6.418469450063	-2.232123594741	-4.409237088732
C	-5.739174012796	0.277824990035	-3.939924007530

H	-6.712849469426	0.395246626232	-3.444944880111
H	-5.146633895060	1.184938534924	-3.807188530000
H	-5.956986794947	0.147607742153	-5.008235696914
C	-2.683130210531	-0.954615495291	-5.979079454840
H	-2.361366869761	-0.578882891371	-6.962845766733
H	-3.198967926455	-0.106784910990	-5.513229692765
C	-3.517456562628	-2.191251907484	-6.101544990996
H	-4.454465266758	-2.246343090386	-6.644166745853
C	-2.884251156119	-3.212549522501	-5.480731508751
H	-3.233903433138	-4.237421744342	-5.414304653049
C	-1.514459494722	-1.404142278227	-5.165899615723
H	-0.695968486755	-0.760404718576	-4.868203407559
C	-1.641054920250	-2.726602330361	-4.898326450584
H	-0.933760831328	-3.328895018681	-4.338803396342

TS1

O	1.552947552903	2.527062501124	-2.006756888077
O	0.471568955130	1.649011348096	0.094738269701
O	2.326628331407	-2.324452384960	-0.418780389434
O	2.438561318720	-1.830505049184	-2.895248958561
S	-0.457926958631	-3.412933304063	-0.641317229850
S	-0.945938677567	2.188607838646	-3.595496442002
O	-0.334199131339	-2.952236248681	0.747248578274
O	-1.790977844150	-3.870826307735	-1.079793161843
O	0.092124083964	2.036796400727	-4.619830290225
O	-2.340669694838	1.931977778220	-4.056132628763
N	0.101055839005	-2.375738148919	-1.743759907166
N	-0.726833856801	1.347413481000	-2.251149779302
P	1.490352747070	-1.589694902680	-1.598374977423
N	1.530347249161	-0.032680221623	-1.442913131873
P	0.679450404474	1.272601102091	-1.466363885520
C	4.217226874671	1.794947961084	-2.747537640111
C	5.205777420113	0.808851561553	-2.641152726214

C	5.569897050120	0.040778633343	-3.735098146732
C	4.966065541786	0.216205892993	-4.986683975363
C	3.954690447437	1.175476127089	-5.076072757695
C	3.582653748479	1.951167408790	-3.982451274810
H	5.679513511451	0.625051747574	-1.680592956110
H	6.344451623569	-0.708944851257	-3.599983759523
H	3.438733291242	1.344389680948	-6.015374869648
H	2.798808429653	2.688006356589	-4.107452261306
C	-2.205552819495	1.823878507334	1.024365518213
C	-1.690307760570	0.660305204776	1.612285660132
C	-2.508950364556	-0.424862079857	1.881554711477
C	-3.874542685714	-0.412007589384	1.575210049287
C	-4.378048488153	0.740693912878	0.964338846227
C	-3.564238272356	1.836835780373	0.696473356109
H	-0.640897296288	0.600075323229	1.876915330755
H	-2.057348532646	-1.300125836770	2.337617433980
H	-5.423268609621	0.800043917239	0.680200902850
H	-3.990490900841	2.707249815495	0.206348603500
C	1.468280123020	-3.176606043842	-5.244044831314
C	1.502247098193	-1.813852464156	-5.566959695337
C	0.697289409580	-1.294914942623	-6.574748359854
C	-0.164453101147	-2.103087556900	-7.320516912904
C	-0.220206791273	-3.458366982071	-6.970872853992
C	0.563499253297	-3.980529423935	-5.952347614039
H	2.176584664892	-1.142091728239	-5.048923879668
H	0.764512226240	-0.229993702950	-6.770305489073
H	-0.886586641538	-4.131096767187	-7.502789639534
H	0.467248890972	-5.032152254736	-5.696350989198
C	3.289881646668	-0.548645633112	1.614322985818
C	2.222811527985	-1.196665953053	2.241851936853
C	1.465920774767	-0.543471526932	3.209600946303
C	1.733255259833	0.774896341724	3.586140309431
C	2.783615426756	1.426167956689	2.927102283706

C	3.542871931188	0.783850926130	1.962919263365
H	1.977277234171	-2.221376199807	1.990101310987
H	0.656251855930	-1.095679868804	3.675015700603
H	3.022965348511	2.458904800446	3.163861792739
H	4.334175273823	1.331300800030	1.457660062019
C	-0.020172649337	2.919399469889	0.368326403592
C	2.591439570653	2.995639736425	-1.210873925985
C	3.696592638841	-2.105391222786	-0.350583672577
C	2.908847969231	-3.110410906297	-3.141940313598
C	-1.365713592258	3.021110014369	0.806818427626
C	2.409058767933	-3.793648694794	-4.283273450050
C	3.915565219605	2.650273643926	-1.580160060896
C	4.181693783374	-1.231918713258	0.655197801321
C	0.482953860985	7.724694240709	0.303287274355
C	1.034155716206	6.481100224147	0.110678241352
C	0.295476636446	5.307774390609	0.399673510553
C	-1.055927302924	5.442833504266	0.849444078008
C	-1.583593116646	6.740983813512	1.059670761892
C	-0.831293613567	7.858060963424	0.798335582683
H	1.060164540242	8.612506852319	0.062718168736
H	2.044293588693	6.387520354826	-0.275293287204
C	0.832565777749	3.998263052825	0.220237044987
C	-1.843783642280	4.290914664937	1.061240280467
H	-2.607686405474	6.834335886200	1.411356983388
H	-2.855582968164	4.412653876147	1.439611560201
C	5.468011405997	5.044986123726	2.365120906015
C	5.722514184312	4.347700012678	1.210978933866
C	4.665723273735	3.908127383550	0.376037411951
C	3.316731741788	4.224666576626	0.733333505196
C	3.089483812080	4.929769513992	1.940415974014
C	4.137519313628	5.328798148997	2.734976110923
H	5.959701178714	2.919820506814	-1.037549855826
H	6.743424621787	4.109423912464	0.923014215794

C	4.926710055840	3.136726139626	-0.776858882704
C	2.260477086302	3.763156455164	-0.111812947414
H	2.071758068011	5.150136862889	2.242137435852
H	3.937221325092	5.863211714279	3.659118639816
C	8.145092003772	-2.749837305047	-2.142411804192
C	6.803025368516	-3.042003612139	-2.194025030386
C	5.903684851827	-2.513956464672	-1.235544514761
C	6.423557807872	-1.643520466705	-0.226532604207
C	7.814504892860	-1.376243993501	-0.193365395698
C	8.660678721741	-1.916946656897	-1.128604403292
H	8.812961958144	-3.161439516481	-2.893635340403
H	6.422570108269	-3.677729539469	-2.985338738480
C	4.495513867826	-2.761177684854	-1.264153490190
C	5.547372255282	-1.038509967440	0.699764855667
H	8.197318501290	-0.720193199283	0.584487041885
H	5.965847182680	-0.401123425630	1.474771536776
C	5.077568891057	-7.641757233773	-2.950736446762
C	4.238102103207	-7.023524553263	-3.842560677962
C	3.806818535185	-5.690930282122	-3.628868765607
C	4.292739538876	-4.977609789229	-2.490529967084
C	5.135211784270	-5.653304450064	-1.574865910950
C	5.513470488637	-6.955275363096	-1.798214929054
H	2.598737493138	-5.603252624663	-5.403750080776
H	3.870063856583	-7.555047013057	-4.716272727571
H	5.471341386340	-5.132600871766	-0.683843994332
H	6.146530874316	-7.463039431107	-1.076692081105
C	2.904234439554	-5.063582628933	-4.511784662254
C	3.883057733826	-3.624087080859	-2.303576422702
H	-1.253132957741	8.847260366912	0.948584241948
H	6.287116308865	5.370522815617	2.999418970996
H	9.723817662235	-1.698020100960	-1.096284127310
H	5.387310054392	-8.669352349478	-3.115574236263
C	-4.763648404858	-1.588479025271	1.981804144733

C	-6.137481470886	-1.531222305609	1.301724845434
H	-6.032089770806	-1.495565792658	0.210962055426
H	-6.714295292829	-2.425619108963	1.561613614332
H	-6.721821794665	-0.661549179860	1.620211523915
C	-4.964464087278	-1.519491561628	3.509557213011
H	-5.432325871277	-0.571856304777	3.798083624349
H	-5.606561558951	-2.341618340013	3.848059413601
H	-4.005564901186	-1.595574492455	4.032566009468
C	-4.106119227943	-2.931928898399	1.622677088716
H	-3.923777321701	-3.011165804047	0.547840701036
H	-3.144676508072	-3.078109048151	2.121354376224
H	-4.763660208850	-3.756091227710	1.922929764204
C	0.968205922993	1.497544858201	4.697375090335
C	-0.196356344410	0.663188606886	5.243959240490
C	0.401993529138	2.830569162670	4.174815653026
C	1.948413862147	1.785523589008	5.852195607373
H	0.150370588405	-0.275153366264	5.690176264134
H	-0.714785838984	1.229225175385	6.025309483538
H	-0.928170098088	0.429863880327	4.462409240771
H	-0.302558081860	2.665611849771	3.353600001737
H	-0.128708245432	3.354086615574	4.978601909938
H	1.195520972671	3.490853166231	3.812062614252
H	1.427904411201	2.289353531661	6.675337077814
H	2.380462920477	0.854319234338	6.234338407192
H	2.770758298018	2.429750567386	5.524329641222
C	-0.943978498518	-1.568293429853	-8.523396200122
C	-2.354978804721	-2.177185281018	-8.595004344891
C	-0.165717994286	-1.963641389540	-9.795236789705
C	-1.080808592403	-0.039753279884	-8.486726896607
H	-2.914865678922	-1.979548199131	-7.675360574180
H	-2.904899685542	-1.737268525978	-9.434170505200
H	-2.331865771228	-3.258974471443	-8.755643719936
H	-0.072286841494	-3.052017990362	-9.875259484303

H	0.843372617706	-1.538218065835	-9.778212793313
H	-0.682584327513	-1.594831487180	-10.689451513546
H	-1.553323325372	0.303080290869	-7.559988054893
H	-0.112650851084	0.461546716215	-8.577636080219
H	-1.698573665210	0.292575664487	-9.327651646626
C	5.461027167187	-0.589298364488	-6.190281156506
C	5.369373805145	-2.097959003402	-5.899199531785
C	6.934098282695	-0.215588922993	-6.450858165278
C	4.656051617655	-0.296382400390	-7.462368915688
H	4.330856147991	-2.406124921039	-5.743400836013
H	5.769004161532	-2.668383619670	-6.745988134052
H	5.941884735922	-2.373755838405	-5.008485090551
H	7.565942466347	-0.458776871608	-5.590492079936
H	7.315828405332	-0.764732580965	-7.319755881502
H	7.032490101420	0.857326544534	-6.648650286093
H	3.597137933536	-0.549946690494	-7.339284960884
H	4.732214501536	0.755684293593	-7.757842678950
H	5.047573929977	-0.901321930446	-8.287324183045
C	0.587518955263	-4.909208675190	-0.816580969490
C	1.432719404547	-5.384373003489	0.184989915174
C	0.474930596663	-5.668586898635	-1.981377592875
C	2.104980823041	-6.594917984337	0.043914681667
C	1.135631544822	-6.877654411229	-2.131852204044
C	1.940817276704	-7.353418601103	-1.105345157276
F	1.631281233977	-4.722956805387	1.324225665046
F	2.903348742750	-7.037358782800	1.018684661713
F	2.555066552957	-8.528255161622	-1.225194098384
F	1.000626064392	-7.586777272311	-3.257476289384
F	-0.285558179569	-5.257634897961	-3.002137912624
C	-0.994638930453	3.953897543313	-3.116958613976
C	-2.114605416080	4.434603958790	-2.437350128208
C	-0.013363006829	4.876426316042	-3.479247580784
C	-2.284163902821	5.784351645383	-2.170615843797

F	-3.079973273821	3.607346451836	-2.024223881019
C	-0.164668681427	6.231264109356	-3.201718148557
F	1.097622474267	4.519581561955	-4.120227803989
C	-1.309781679595	6.690258386573	-2.567322588812
F	-3.380693845437	6.213559996776	-1.540838371602
F	0.784260436222	7.098781967129	-3.560331925365
F	-1.471824903825	7.991095454366	-2.340958361359
O	-4.183944867901	0.468100560934	-2.931328378083
H	-3.544336419786	1.188716050957	-3.196380668279
C	-3.526770163346	-0.441861951108	-2.240825736202
H	-2.574351395739	-0.137665551426	-1.804337703337
C	-3.982240550581	-1.723245195686	-2.046500683656
H	-3.304993110335	-2.343989814535	-1.469138411196
C	-5.105151903898	-2.355398925766	-2.638951573100
C	-6.338251968418	-1.589468993912	-3.054116866677
H	-6.950798095692	-2.182756485672	-3.741116193745
H	-6.944020297821	-1.411761584831	-2.154690131476
H	-6.111921937409	-0.624881126009	-3.501718417783
C	-5.419433789711	-3.731649226151	-2.094749389661
H	-4.517843409396	-4.311698532699	-1.884527094373
H	-5.956483793044	-3.605085549970	-1.144627388968
H	-6.074307749330	-4.296367566220	-2.764512152640
C	-2.887585880811	-3.335441130672	-4.129766301124
H	-2.742501022813	-3.837939690407	-3.172397539832
H	-2.521323440048	-4.030978880388	-4.906364092252
C	-4.285612330339	-2.911134074680	-4.511580644446
H	-5.071115226671	-3.623772990230	-4.744044700631
C	-4.157183135038	-1.694406901469	-5.191233807758
H	-4.956387762505	-1.190145670911	-5.723852440211
C	-2.118516180980	-2.075123841934	-4.283163400523
H	-1.100126239920	-1.948827344885	-3.934573476931
C	-2.860447488908	-1.172096477582	-5.005317133885
H	-2.526475417919	-0.189708722973	-5.314907189854

TS1′

O	1.106656164026	2.636937187322	-1.778841551224
O	0.021399212658	1.380473938662	0.117066922657
O	2.406054553150	-2.046464141886	-0.354280089753
O	2.658947494309	-1.618672897367	-2.845216212561
S	-0.042199974788	-3.623008810472	-0.746538807438
S	-1.263697196957	2.269566137331	-3.564767515660
O	-0.123425970387	-3.159508059176	0.641533950750
O	-1.253651147013	-4.325583690306	-1.266410017446
O	-0.123143303303	2.338725556356	-4.490403270185
O	-2.583403703440	1.964599759649	-4.150860143848
N	0.358579326909	-2.512657423926	-1.829338018188
N	-1.031321697475	1.277483316394	-2.312592330207
P	1.585433956508	-1.484289366123	-1.633624340913
N	1.332634237765	0.050873771207	-1.598815329631
P	0.332624814006	1.247996199867	-1.468684309245
C	3.908910662403	2.339324747771	-2.358279589368
C	5.076787662333	1.576921209815	-2.222088800904
C	5.644058515988	0.931904348154	-3.307141884877
C	5.073843624554	1.008487430228	-4.583587836310
C	3.893976890957	1.746037806395	-4.709287471739
C	3.317129254253	2.400936254034	-3.623450336736
H	5.533620360345	1.465322938592	-1.243381033717
H	6.548605133072	0.353218117402	-3.144318569828
H	3.401086106664	1.836402808409	-5.671817098106
H	2.405755037333	2.965317439987	-3.779624907172
C	-2.752649712207	1.120960225814	0.797012674894
C	-2.171888816595	-0.085071900985	1.206001117747
C	-2.917393952970	-1.259847210195	1.253852579357
C	-4.269066488922	-1.287239871246	0.901498873525
C	-4.835226199202	-0.083328648672	0.462177059708
C	-4.098633611625	1.089087724058	0.405140528485

H	-1.133325346727	-0.118575622024	1.515373493376
H	-2.409570409391	-2.162323544920	1.575555009429
H	-5.877481746605	-0.051692078891	0.158005416684
H	-4.570030005449	1.994216439442	0.034582005020
C	2.295932592856	-3.184587411845	-5.240958157309
C	2.169071026485	-1.863340387404	-5.682623061288
C	1.477080064373	-1.563234643670	-6.853711492302
C	0.882882223740	-2.557344401695	-7.632449776683
C	0.955128043374	-3.869746399678	-7.147508745592
C	1.638179280347	-4.177402017011	-5.982633785442
H	2.639021710988	-1.054731515326	-5.132659642118
H	1.426771222850	-0.522787790924	-7.157973052842
H	0.479325217762	-4.678694315181	-7.694994264566
H	1.662892027956	-5.207365404933	-5.637722974853
C	2.827800044070	-0.271165785295	1.847960686083
C	1.877469112588	-1.174522758238	2.330468845164
C	0.922937149516	-0.778675872190	3.260502361290
C	0.874730318892	0.528940713547	3.747839551315
C	1.815909041836	1.433063934015	3.240356448769
C	2.770041004129	1.047892862073	2.312189325088
H	1.879096376171	-2.203121586708	1.995032027435
H	0.213788806309	-1.523983281821	3.604292105429
H	1.813744897205	2.466535724377	3.572600915421
H	3.468544394821	1.786356412791	1.928129762824
C	-0.656220018400	2.534695190650	0.500546887786
C	2.011902030278	3.147916376074	-0.860398161515
C	3.700537695681	-1.594912678675	-0.131917951613
C	3.407209831916	-2.778623321714	-2.973777156813
C	-2.031680114450	2.412300112177	0.828253955977
C	3.195312160379	-3.574621860472	-4.132326575999
C	3.392490898712	3.056691129097	-1.172578595537
C	3.914511716952	-0.693696852938	0.941467680654
C	-0.817854484252	7.339309743267	1.034006312058

C	-0.094095852127	6.213649388036	0.724450053194
C	-0.672592518406	4.925144721544	0.834761608003
C	-2.047560873861	4.825990716248	1.211448855555
C	-2.757393610981	6.005634759291	1.548014590378
C	-2.155814340780	7.235590886453	1.469353583424
H	-0.360224017674	8.318526616846	0.929141525779
H	0.931039444619	6.305819960908	0.381212347316
C	0.047883134331	3.727215299571	0.541984381500
C	-2.682960538881	3.567716542370	1.214283354767
H	-3.799535553951	5.917941283056	1.843639362415
H	-3.721886053194	3.513340381912	1.528365970887
C	4.344987125949	5.354314404442	3.014378377812
C	4.759552654719	4.821762912314	1.820114527287
C	3.829073288334	4.267497076908	0.906199920722
C	2.434316653465	4.297001311627	1.221636943226
C	2.045577700617	4.831468408369	2.474846603687
C	2.974947085267	5.346125384899	3.346452602238
H	5.330650193566	3.643563341595	-0.504310285600
H	5.814833722016	4.803243881088	1.559279612156
C	4.266323688567	3.650728129075	-0.283913133145
C	1.508754194409	3.740890950123	0.282627146705
H	0.997015749900	4.829463399569	2.749981362418
H	2.649487570699	5.745881794587	4.302479360395
C	8.352442804281	-1.406075506002	-1.435252506646
C	7.098234137890	-1.934115974927	-1.628384576337
C	6.023142249887	-1.589451978929	-0.774062348420
C	6.267936758013	-0.659618897784	0.285488852315
C	7.575932338248	-0.145825354297	0.464041919809
C	8.598758353477	-0.508994004322	-0.375963748432
H	9.159615678546	-1.679489479410	-2.108723088538
H	6.923604337717	-2.617093640099	-2.452347606629
C	4.693675778975	-2.084237215713	-0.952191264519
C	5.208944912201	-0.253512876758	1.126111509428

H	7.750995509645	0.553947185976	1.277522831085
H	5.425801191033	0.418765847419	1.952664216147
C	6.351216335260	-6.803571742277	-2.315739549270
C	5.528502653953	-6.380504189722	-3.329005881177
C	4.837546743559	-5.147501402939	-3.234042746366
C	5.042056919763	-4.319720092914	-2.088415220547
C	5.876005915574	-4.797964919973	-1.048848612466
C	6.508566907466	-6.013081332976	-1.158468511376
H	3.862009581211	-5.334576763038	-5.140559110662
H	5.373547975087	-6.996548387417	-4.210917892458
H	5.999845602801	-4.198323396400	-0.152877822003
H	7.129782354502	-6.370911494538	-0.342841315896
C	3.948394300316	-4.727804998615	-4.243702781716
C	4.366595809678	-3.063645549229	-2.016826333913
H	-2.715002362010	8.133753726311	1.713522422427
H	5.068304432982	5.770618674556	3.709157012537
H	9.595351284457	-0.102436322083	-0.231883782111
H	6.861734134034	-7.759014576029	-2.389939368997
C	-5.136945624321	-2.538230317309	1.052528036897
C	-4.300036083521	-3.793289216008	1.330429065720
H	-3.766949747685	-3.724143881704	2.284145422801
H	-4.959677657049	-4.665913976892	1.386123453723
H	-3.562947899395	-3.977488434741	0.541079669768
C	-5.970087107041	-2.787592516129	-0.218095674810
H	-5.320863634097	-2.942761921763	-1.084202892020
H	-6.584623886907	-3.685495366319	-0.085783864518
H	-6.646988340098	-1.956289565873	-0.438155133684
C	-6.092290034141	-2.310916369027	2.242148617758
H	-6.728581268414	-3.191308382487	2.392352653379
H	-5.526904227632	-2.131487932882	3.162896518964
H	-6.739961385415	-1.444967011498	2.070153691281
C	-0.113238027267	0.981648621152	4.825028260140
C	-1.102332885518	-0.125319239993	5.208789563541

C	-0.922363632381	2.194972991435	4.333182646842
C	0.686631035289	1.381228127735	6.081150216267
H	-0.591194786026	-1.000171750747	5.624802289924
H	-1.791811340543	0.249827618483	5.972776907042
H	-1.701085839308	-0.446283913605	4.348639930498
H	-1.530375104582	1.933263892009	3.462162010701
H	-1.594673433458	2.542851624661	5.126177395000
H	-0.274644455758	3.031923558030	4.053463078629
H	1.288161040032	0.539783325211	6.441663363446
H	1.364103089286	2.216000226627	5.874178499154
H	0.004686810356	1.688022026748	6.883095636684
C	0.226143059954	-2.279051200251	-8.986069586285
C	-1.211187601526	-2.829498600580	-9.010257854753
C	1.053770345506	-2.986642506125	-10.078371486297
C	0.180224360218	-0.782482463171	-9.315272546686
H	-1.825139977406	-2.359438693651	-8.235436995791
H	-1.673657472358	-2.629983161509	-9.983767236341
H	-1.238748771581	-3.910551111374	-8.844049576187
H	1.079978302287	-4.069685420230	-9.922218890349
H	0.619369152972	-2.795970615959	-11.066769083847
H	2.086018662197	-2.620823209807	-10.077774340861
H	-0.389249716409	-0.214224026491	-8.571362227384
H	1.184559093446	-0.350447400714	-9.377766741565
H	-0.303597621683	-0.635933625428	-10.286553383381
C	5.776726828137	0.331349038444	-5.762170707296
C	5.959647523375	-1.171219870326	-5.474713804826
C	7.163762029761	0.980620115408	-5.942395748999
C	5.001246820363	0.483220891350	-7.075659313991
H	4.993710475233	-1.670797262017	-5.351427127206
H	6.486897270729	-1.652278111223	-6.306951919094
H	6.543874669135	-1.338325796958	-4.564608873554
H	7.785553733204	0.848324891440	-5.051354231356
H	7.687182336315	0.526674328248	-6.792252015350

H	7.066688893425	2.055454293285	-6.129683065590
H	4.010133725330	0.021171980053	-7.017868421916
H	4.881303873713	1.535235348991	-7.356203828237
H	5.550377751686	-0.015243903896	-7.881744590737
C	1.230347154597	-4.929989719417	-0.824749988523
C	2.045672471480	-5.272051469864	0.253880396586
C	1.349501532811	-5.683365153841	-1.993363915573
C	2.915690918871	-6.355482124329	0.179816312290
C	2.212030592142	-6.764832511993	-2.077614278633
C	2.984719991600	-7.115663276257	-0.978159162479
F	2.027480214810	-4.600073930242	1.402839138882
F	3.676030686414	-6.676526091177	1.228080691369
F	3.787299528932	-8.174522303513	-1.034463794652
F	2.299450774573	-7.472309828061	-3.207529037765
F	0.627081237276	-5.388460335321	-3.077590982521
C	-1.506018163873	3.945828670195	-2.863705733169
C	-2.718859729107	4.236670429964	-2.237166677612
C	-0.600533282342	4.992404278672	-3.036071824336
C	-3.047165704536	5.525711520690	-1.844734499761
F	-3.622596799297	3.282701335757	-2.003200644686
C	-0.911968708279	6.285700912088	-2.629269780398
F	0.590329571575	4.818230541475	-3.607279678471
C	-2.144747762378	6.558967012455	-2.055283688321
F	-4.230027139595	5.775253921635	-1.275208461614
F	-0.029990990464	7.274461299123	-2.800327012775
F	-2.456473358855	7.805092718232	-1.704607304989
O	-3.195883847569	-3.087963030046	-2.403064599024
H	-2.460940733724	-3.544393914106	-1.885131396438
C	-2.997988622121	-1.789010044076	-2.433142256767
H	-2.252926001764	-1.368520837372	-1.755297915527
C	-3.697026511576	-0.950377099866	-3.267450735861
H	-3.419713507598	0.096536498157	-3.192161008147
C	-4.607059903428	-1.323649264010	-4.288535245902

C	-5.462985054654	-2.565305491775	-4.209906659920
H	-4.933656988247	-3.431406157666	-3.821663546740
H	-6.301321973348	-2.351901361636	-3.532226191536
H	-5.891319326783	-2.801950578704	-5.189397060940
C	-5.307872865015	-0.168757894784	-4.967089302917
H	-6.136466216479	0.147941154819	-4.318769882852
H	-4.654156416061	0.695008455766	-5.103774601541
H	-5.742867606078	-0.461846137804	-5.926993016722
C	-2.200222961250	-0.791336897247	-5.705564270294
H	-1.698159993373	-0.680481502614	-6.682814053210
H	-2.535714661626	0.205131351113	-5.417985481326
C	-3.239329719329	-1.875432979502	-5.839397407619
H	-3.999001717750	-1.867560950464	-6.615030547167
C	-2.642072213091	-3.055543283630	-5.385824431569
H	-3.068560241911	-4.048225270693	-5.480819914953
C	-1.216796362143	-1.407183517636	-4.778714202398
H	-0.404693917547	-0.864605788603	-4.309650462458
C	-1.441629373100	-2.759157185350	-4.704502168994
H	-0.834115593058	-3.466477033054	-4.154870490878

II

O	1.577419430737	2.524315456603	-2.015915065768
O	0.464245018226	1.719755245116	0.098551182408
O	2.332483874954	-2.334741156639	-0.380390462725
O	2.439167981784	-1.791340486941	-2.843022450695
S	-0.447163123312	-3.381135113538	-0.567236105589
S	-0.855412203102	2.137535934017	-3.617783349012
O	-0.289372268107	-2.918156075586	0.817505313781
O	-1.789723206471	-3.828304135486	-0.978098437882
O	0.202933139680	2.010536788702	-4.619814917951
O	-2.242609726502	1.838663694833	-4.135682869130
N	0.092752323562	-2.341012177677	-1.685385208706
N	-0.684104488432	1.310027957703	-2.267935806325

P	1.495559471100	-1.576023574007	-1.541829841942
N	1.550295362531	-0.020958980263	-1.340605978430
P	0.709115144479	1.282898143725	-1.437736002863
C	4.253941330448	1.794510555571	-2.717627590497
C	5.213333696094	0.784459428831	-2.579261893716
C	5.600013807742	0.014636182814	-3.664405356700
C	5.050091165057	0.214800950017	-4.937065898379
C	4.067308095392	1.200281463230	-5.058570494564
C	3.669894662912	1.974906431165	-3.973346788913
H	5.646586728940	0.585789211595	-1.602382513576
H	6.349998503075	-0.755272439372	-3.505967017291
H	3.595771629019	1.389169020693	-6.017160303706
H	2.909324783152	2.733073685601	-4.119206780744
C	-2.194289768458	1.931698821333	1.070509754348
C	-1.662768121791	0.775696829682	1.661477494066
C	-2.474658928449	-0.301616197949	1.971009499467
C	-3.850840399792	-0.285662706765	1.713564745216
C	-4.373922417058	0.861483921722	1.111089505837
C	-3.564055932478	1.948645825372	0.795502132197
H	-0.605653000627	0.712873158903	1.892111769357
H	-2.011017197488	-1.177422218214	2.415376686984
H	-5.431090272065	0.924900582657	0.876458758800
H	-4.003712557681	2.814425580412	0.308600054671
C	1.469866868785	-3.001908897242	-5.237635413776
C	1.622625411081	-1.636563807260	-5.522974232613
C	0.843611956949	-1.018893488014	-6.487111682394
C	-0.113956587967	-1.722012675859	-7.226761630673
C	-0.272887023163	-3.078918667268	-6.931355781569
C	0.492348717449	-3.703344930926	-5.950942540664
H	2.367659731081	-1.045451266918	-5.004349116081
H	0.997484897875	0.042456086836	-6.660100927050
H	-1.018718321870	-3.669913684309	-7.452287036928
H	0.313509498608	-4.751958503145	-5.727812979327

C	3.346007485670	-0.562901871630	1.624551798033
C	2.274779971513	-1.181469740383	2.273609818634
C	1.530945865249	-0.494005672681	3.227839514361
C	1.816379639550	0.830226322607	3.569140209477
C	2.869326019971	1.452832649380	2.886382450520
C	3.616093227801	0.775714617616	1.935735465946
H	2.015268063416	-2.209012952242	2.048306045374
H	0.717861549388	-1.024062457459	3.712635584199
H	3.120596359489	2.489274775207	3.094586233225
H	4.409224326122	1.300486510947	1.409327247594
C	-0.019529832393	2.999577894268	0.339288816658
C	2.609139910280	3.019737244537	-1.222249185243
C	3.707310807040	-2.144970787897	-0.327745051889
C	2.881519600637	-3.074265119213	-3.129187974774
C	-1.357637494740	3.118408771303	0.796227350116
C	2.363971630974	-3.704612108424	-4.292008152603
C	3.936472656687	2.661883271306	-1.564312554927
C	4.219029643993	-1.279714282872	0.671816526393
C	0.504841776247	7.797276366101	0.128958516649
C	1.050677653648	6.545816051613	-0.025236490407
C	0.307267972940	5.385947748366	0.301986675347
C	-1.040973451456	5.539993151042	0.754644749785
C	-1.563117764577	6.846289320945	0.924146321123
C	-0.807292922153	7.951166240378	0.623621130816
H	1.085061095779	8.674857327998	-0.140142439724
H	2.059806919658	6.434651302867	-0.409530326675
C	0.837639198117	4.069348799369	0.160286254936
C	-1.828132629948	4.397940856911	1.015507815602
H	-2.584901096405	6.955541316313	1.278065673233
H	-2.832492133230	4.535995288169	1.407415028843
C	5.440632231018	5.109634771849	2.365651438328
C	5.709478059229	4.387161078582	1.230436756673
C	4.664293550598	3.947244325491	0.381513298828

C	3.312724468840	4.287916602208	0.704452966435
C	3.070325638889	5.021819803495	1.891130814485
C	4.107096356348	5.420814911276	2.700412518320
H	5.972931450440	2.922203459442	-0.993225016586
H	6.732621855661	4.128806026819	0.969126544097
C	4.938060909990	3.153604045761	-0.753045976455
C	2.266891380931	3.817524028628	-0.149571235287
H	2.050097990719	5.263415795949	2.166492393466
H	3.895057509392	5.976322021797	3.609334189457
C	8.127251945688	-2.891993150562	-2.149334558821
C	6.777983989151	-3.150452131189	-2.194469675280
C	5.896847519658	-2.602884546851	-1.229966355993
C	6.444172252803	-1.747559765798	-0.221972800024
C	7.841498724052	-1.515443544880	-0.195669549575
C	8.669069563977	-2.074941757419	-1.136461709321
H	8.780477332759	-3.318855683020	-2.904912538621
H	6.378277571156	-3.775510132044	-2.984599703748
C	4.482439757116	-2.815153479130	-1.251590571826
C	5.588797678806	-1.118967581470	0.708438538078
H	8.244762521000	-0.871309778109	0.581799885117
H	6.026525516120	-0.487466585416	1.477725416082
C	4.883803244491	-7.680817833080	-3.051238004384
C	4.058089159979	-7.014659525062	-3.921252143708
C	3.677809871230	-5.671613363898	-3.678022307033
C	4.200562327711	-5.000601630740	-2.529084835662
C	5.027724246959	-5.724782269638	-1.636824786007
C	5.357594368060	-7.034154146241	-1.890633570693
H	2.468019033835	-5.495994150553	-5.450040916402
H	3.663901780576	-7.515190355068	-4.801791899425
H	5.390302160720	-5.234172318560	-0.738984961440
H	5.979823535435	-7.578890077428	-1.186854173604
C	2.799178910582	-4.990548052948	-4.546929552691
C	3.838710699979	-3.639150604258	-2.305099219403

H	-1.224806453389	8.946338550567	0.743491993093
H	6.250586060958	5.434664426572	3.011811515411
H	9.737561381945	-1.883158756702	-1.108997992236
H	5.154430831320	-8.715247865444	-3.240280227296
C	-4.704810742186	-1.489386563396	2.109820308725
C	-6.168619257928	-1.326705177403	1.686165380774
H	-6.262454093911	-1.189679863494	0.602974209535
H	-6.731787371334	-2.225749432498	1.958184971963
H	-6.644787607598	-0.473715942982	2.182227577531
C	-4.657638675274	-1.648497519955	3.642082713687
H	-5.046316424177	-0.752679451809	4.138647154024
H	-5.264500377020	-2.507451069871	3.951804038459
H	-3.634706804407	-1.813288078103	3.994060922407
C	-4.136199032751	-2.757894662565	1.448876281490
H	-4.122638312942	-2.655187477184	0.360092381135
H	-3.109171172873	-2.964335621057	1.760970141507
H	-4.752362520657	-3.628014541615	1.704225194807
C	1.072320542867	1.585872760064	4.673340440578
C	-0.096079334033	0.778451730576	5.251568441948
C	0.518328845440	2.916678775031	4.132430393002
C	2.071037709411	1.883512680306	5.809953906949
H	0.244022753495	-0.155500320679	5.711660997168
H	-0.595772544443	1.367169780328	6.028275529296
H	-0.841757015037	0.540180454018	4.484909463595
H	-0.203403263707	2.747672428227	3.327035204671
H	0.009716604667	3.464651086696	4.934221701749
H	1.316442223521	3.556330276450	3.744083269414
H	1.569066368930	2.413246087891	6.628348670698
H	2.493609472429	0.954126027441	6.206764033366
H	2.899044010188	2.507316923919	5.457725867405
C	-0.906856389733	-0.997348834535	-8.316540088063
C	-1.943093635754	-1.907178942326	-8.987456427260
C	0.079958781864	-0.511785216692	-9.397359144302

C	-1.637632687361	0.221637277356	-7.719827950154
H	-2.673376217602	-2.295943223898	-8.268465913195
H	-2.493106133450	-1.337556068059	-9.744069387835
H	-1.470905631248	-2.757616027987	-9.491562182810
H	0.632403705730	-1.355835827941	-9.824237750122
H	0.807876177821	0.195445688559	-8.987977550462
H	-0.462619528639	-0.008597234890	-10.206202963285
H	-2.386321302927	-0.083659427916	-6.980745958072
H	-0.951429943884	0.913231925880	-7.222923450800
H	-2.156591121345	0.770890693005	-8.513927832047
C	5.569406713243	-0.595224244512	-6.127491202269
C	5.449757665568	-2.102990323599	-5.841890546627
C	7.054209027582	-0.239846707915	-6.342518165984
C	4.804683309297	-0.290511226246	-7.421352323039
H	4.403846201435	-2.396712911894	-5.709158937837
H	5.859793677890	-2.676751377300	-6.681245280769
H	5.999634210152	-2.388297853629	-4.940295213138
H	7.655766642103	-0.490117286066	-5.462553404815
H	7.455929660826	-0.794268168666	-7.198940143257
H	7.171646842687	0.831590158218	-6.537978278244
H	3.739080412702	-0.527444146286	-7.325963695661
H	4.906253397410	0.759539731546	-7.716490433412
H	5.208462619677	-0.902752711360	-8.234809447634
C	0.584547871582	-4.884624223453	-0.766113296906
C	1.439271181500	-5.372126021793	0.221560608490
C	0.435365890640	-5.647064644111	-1.925008581097
C	2.082990201059	-6.597181925276	0.074283690582
C	1.065242841650	-6.872077214410	-2.080159518106
C	1.879058919960	-7.359445751561	-1.066198865177
F	1.674545599967	-4.708205973040	1.352460427481
F	2.892865547592	-7.050274945288	1.034722997909
F	2.464111875592	-8.548731574164	-1.189327910275
F	0.887750249622	-7.586097214583	-3.195990936262

F	-0.335470680602	-5.227218667118	-2.933537778219
C	-1.001388653089	3.897425689990	-3.178804849375
C	-2.140249504334	4.330848293661	-2.497054843292
C	-0.058500902922	4.856142377398	-3.551328367319
C	-2.365225035920	5.674602037330	-2.241948917001
F	-3.059201528173	3.462881984916	-2.071313446789
C	-0.271234788039	6.205630810612	-3.289770833029
F	1.068453757356	4.538588288583	-4.183228566422
C	-1.434102606621	6.618557110842	-2.655369609306
F	-3.470585388451	6.061717748982	-1.602107278693
F	0.635107358778	7.110292426464	-3.663162522709
F	-1.653218962295	7.912063279394	-2.441999513440
O	-3.621261643381	-0.101554008463	-3.676353436937
H	-2.973963058312	0.769308900904	-3.776634763125
C	-3.198311690546	-0.971124699110	-2.867032301037
H	-2.306949671439	-0.734370548971	-2.266664692816
C	-3.719195772098	-2.311299678854	-2.802851776637
H	-3.412939861379	-2.739653387482	-1.846034236919
C	-5.169848728921	-2.741408201509	-3.202800243299
C	-6.098152071165	-1.623510349832	-3.684274431377
H	-7.072054308468	-2.055607068017	-3.943137564099
H	-6.264645383056	-0.881242319691	-2.895561114011
H	-5.707658009413	-1.103344917633	-4.559339878948
C	-5.834710250322	-3.433848312725	-2.004866815878
H	-5.224558073780	-4.246770945614	-1.601484599695
H	-6.010690328243	-2.719251850479	-1.194601451140
H	-6.805246090033	-3.845673920664	-2.302872521325
C	-3.561471855572	-4.438074880105	-3.850611236413
H	-3.601661876991	-4.887376157221	-2.856066841689
H	-3.151815879826	-5.155071041640	-4.568614477534
C	-4.872550677129	-3.796089006347	-4.345790775990
H	-5.722352135720	-4.458430921097	-4.532322268192
C	-4.357370414605	-3.019005465801	-5.537344583575

H	-4.944086575012	-2.755232033293	-6.410614520662
C	-2.765967989823	-3.123807479303	-3.886677208610
H	-1.721220024671	-3.132571250984	-3.580837602492
C	-3.117397546761	-2.603132921486	-5.248641526287
H	-2.488144366522	-1.938345062090	-5.824606279604

II′

O	1.105340137336	2.644658736154	-1.730197339620
O	0.025107053945	1.420565298066	0.185987799428
O	2.390990991219	-2.068666208634	-0.251564068329
O	2.563580603225	-1.680836776166	-2.753283986043
S	-0.080837167710	-3.552244200043	-0.509938063820
S	-1.239126989217	2.210312981935	-3.531723543870
O	-0.065416097279	-3.135257149902	0.891095903112
O	-1.378787260957	-4.197058182138	-0.940582492595
O	-0.081540092482	2.291248865592	-4.433714687950
O	-2.540232303954	1.866340092983	-4.128905791637
N	0.257692304225	-2.447886380521	-1.611232460078
N	-1.004497933734	1.234956824711	-2.258361745925
P	1.552566505777	-1.476700616706	-1.503429958111
N	1.365723456430	0.065395187546	-1.473590326137
P	0.345738000627	1.255873951924	-1.394942039476
C	3.902432079771	2.364101877878	-2.295999488161
C	5.063201280024	1.593258569081	-2.148212696295
C	5.623005190134	0.924920527870	-3.223903822234
C	5.051269899383	0.987517562187	-4.500987461726
C	3.881487642351	1.738692152982	-4.637984864607
C	3.312575569276	2.415531086603	-3.562256765258
H	5.518685709954	1.492400549651	-1.167583942490
H	6.520448291849	0.336886774181	-3.053828601723
H	3.388772089274	1.819290130727	-5.601285943902
H	2.407477433128	2.987357581805	-3.726753414534
C	-2.725830550072	1.130015395203	0.872516020822

C	-2.124872041271	-0.039737756337	1.349713340416
C	-2.834004932123	-1.236161155269	1.409241264949
C	-4.165243644853	-1.321111302303	0.995374276592
C	-4.751273833359	-0.152269046838	0.489863135342
C	-4.052972472053	1.043651067432	0.429390341395
H	-1.098602501437	-0.027971555055	1.700844910013
H	-2.316861008552	-2.109171789364	1.790209648345
H	-5.781085280050	-0.165683722045	0.142776186022
H	-4.536276773575	1.922683341894	0.014379183193
C	2.198495003073	-3.190022027568	-5.192298422999
C	2.048862891762	-1.848145673566	-5.562841385609
C	1.425755410083	-1.499472288766	-6.758494570044
C	0.936392310384	-2.461560874411	-7.642455747466
C	1.016630593896	-3.798190649477	-7.228765042193
C	1.618286659546	-4.154043365527	-6.033569097330
H	2.457342726483	-1.056452131048	-4.945231230040
H	1.356572006141	-0.443890885514	-6.999550917353
H	0.608438640303	-4.586073411619	-7.855679843359
H	1.645498351514	-5.202246466781	-5.748662177914
C	2.915211461705	-0.262463520938	1.892404047824
C	1.996448811189	-1.151782734421	2.454050323942
C	1.067865803375	-0.719116451534	3.393893712529
C	1.018464766123	0.612272244831	3.812348146023
C	1.928915697199	1.500557123294	3.226526893046
C	2.855932474364	1.078582270643	2.286661278776
H	2.004706240730	-2.197046104821	2.171207420688
H	0.382347021243	-1.453002894096	3.803628044634
H	1.922744211460	2.550225883414	3.503803930372
H	3.531252040400	1.802223556986	1.837644830917
C	-0.664763522316	2.574505020908	0.544706253079
C	2.000085636454	3.185680234320	-0.818382177273
C	3.703966178611	-1.644262026334	-0.076906531703
C	3.295519013306	-2.849100395034	-2.901127578227

C	-2.037124264659	2.438809156517	0.876052561164
C	3.066802463763	-3.623038133402	-4.073656254079
C	3.382644638929	3.098757282128	-1.122564659707
C	3.968753878456	-0.722773192663	0.966005341614
C	-0.896760871025	7.384541594396	0.974400401325
C	-0.155181024694	6.262866392067	0.693770791479
C	-0.716133859921	4.969015764071	0.828107909207
C	-2.092048363497	4.858236192791	1.200401955986
C	-2.820446650229	6.034814423118	1.505894415330
C	-2.235828797354	7.271215041784	1.403468895995
H	-0.452352225918	8.367687297342	0.851132613535
H	0.870701425755	6.362030333691	0.354490465907
C	0.023275159558	3.776330589006	0.561925406707
C	-2.709791848739	3.590726624942	1.231110588592
H	-3.863028856247	5.938923929788	1.797337109258
H	-3.750109879495	3.526679736141	1.539459904717
C	4.295409831842	5.484819098867	3.023595203357
C	4.720807962294	4.928316401935	1.844117609578
C	3.799770476887	4.350521541569	0.935278252944
C	2.402457474256	4.380552330164	1.240556858807
C	2.002410330492	4.940554754265	2.479065436820
C	2.923133223829	5.478144670701	3.346021259103
H	5.314168618223	3.707794604552	-0.455031403901
H	5.778037938047	4.909434098446	1.591397218609
C	4.248556550160	3.713471956506	-0.240459158288
C	1.485406191801	3.799643631231	0.308136038684
H	0.951953591786	4.940272013385	2.746631913656
H	2.588928673883	5.897560410961	4.290526469004
C	8.311286618119	-1.577722600410	-1.534354423785
C	7.042370966071	-2.087642692823	-1.672634733834
C	6.001096601105	-1.699302628425	-0.795802502601
C	6.297048216243	-0.747192615905	0.230654093701
C	7.618545493227	-0.252025017442	0.352291963352

C	8.607082508921	-0.655902887896	-0.509856572668
H	9.091284502491	-1.884846160429	-2.225052171960
H	6.830153945687	-2.790193760791	-2.470491271734
C	4.655927520860	-2.170121264679	-0.920803461752
C	5.274466356025	-0.298888677584	1.095281889942
H	7.832270689648	0.466139028643	1.140085977383
H	5.529548776222	0.393090828008	1.894300121611
C	6.173575481756	-6.927703595903	-2.301556042026
C	5.349253225419	-6.482750890344	-3.304412799402
C	4.684911515836	-5.236388225764	-3.196168776603
C	4.919602176395	-4.419586308353	-2.049143158628
C	5.757377649835	-4.917066348169	-1.022318369324
C	6.362011677445	-6.145459977122	-1.143460093834
H	3.715600679560	-5.377327383333	-5.105682781019
H	5.173748833636	-7.091183185193	-4.187849871085
H	5.905961835955	-4.321493571150	-0.127210461137
H	6.986008019306	-6.519241544122	-0.337202037255
C	3.803408406192	-4.786613747585	-4.199028927025
C	4.270611222733	-3.150861255819	-1.964822722964
H	-2.809346772051	8.166419550878	1.624019761516
H	5.012062383477	5.919237688642	3.714149545432
H	9.614446767116	-0.263009120800	-0.409592425048
H	6.663135260361	-7.893240542237	-2.385619181805
C	-4.993513276040	-2.601045200786	1.124611921340
C	-4.163684865491	-3.781424949324	1.643766501181
H	-3.772946050040	-3.589155400801	2.648648763089
H	-4.797110412798	-4.673316301483	1.700641335683
H	-3.320318707690	-4.008331230608	0.982853360479
C	-5.581413269978	-2.992998130757	-0.243012418102
H	-4.782955259606	-3.239177081730	-0.950192969569
H	-6.221960320864	-3.876456277304	-0.139604614858
H	-6.187097598286	-2.190080989180	-0.675171617756
C	-6.145353637721	-2.340163927232	2.115268431176

H	-6.744492415542	-3.248863068547	2.246324331166
H	-5.751952784519	-2.042551265445	3.093085075771
H	-6.809569240546	-1.544320340150	1.763293491150
C	0.058925216499	1.110679740048	4.895886147663
C	-0.880795014799	0.006110047748	5.393958180120
C	-0.804208763201	2.264272916029	4.354491442787
C	0.892869061323	1.618011810961	6.088892904410
H	-0.326617548353	-0.825769563052	5.841770119787
H	-1.547412540628	0.412809368805	6.161969320651
H	-1.506449474069	-0.386205958416	4.584114698197
H	-1.444283468074	1.922733026902	3.535263747852
H	-1.449401817191	2.654975854440	5.150042158320
H	-0.193318908539	3.092132896044	3.981376421967
H	1.525153496752	0.817280344148	6.487525664328
H	1.544729134810	2.447777482882	5.797120687222
H	0.233171173516	1.970225874211	6.890762294519
C	0.403737569957	-2.118830346679	-9.034599265974
C	-0.991015735151	-2.731188280246	-9.254728638535
C	1.381290125123	-2.707593814577	-10.072717289751
C	0.312786425001	-0.606121080850	-9.267256030904
H	-1.705660079876	-2.355713735956	-8.515976197014
H	-1.356028354142	-2.474620066129	-10.255729411620
H	-0.975741492985	-3.822675655263	-9.177523509789
H	1.449903536886	-3.796550404492	-9.984289299361
H	1.044429907699	-2.469022486009	-11.088411893590
H	2.386235295615	-2.293878340641	-9.935252622189
H	-0.346949436387	-0.118206100261	-8.541856995128
H	1.295807690548	-0.126179853808	-9.211998719809
H	-0.091229706379	-0.415171500128	-10.266897328818
C	5.733839488770	0.273904675407	-5.669867216177
C	5.879157648900	-1.226925971834	-5.353902818212
C	7.135265289943	0.884978064816	-5.867905018814
C	4.954821680479	0.419165064419	-6.982066008241

H	4.900893096262	-1.692945137502	-5.198745935374
H	6.373482070051	-1.741809568040	-6.186137389608
H	6.478172011948	-1.390063250025	-4.453021958198
H	7.757492297308	0.755762379183	-4.976614930596
H	7.644391433721	0.401521385100	-6.710168152917
H	7.063486603642	1.957768100248	-6.077080518596
H	3.954129537071	-0.019695571880	-6.909798196132
H	4.857015650147	1.468219575919	-7.281792086396
H	5.488342950506	-0.106457306638	-7.781350490734
C	1.112861812917	-4.909900050744	-0.701981458515
C	1.965777899708	-5.324724251301	0.321333928474
C	1.139981662370	-5.616703909139	-1.906020985147
C	2.779641707998	-6.441832890747	0.162686142554
C	1.950170339005	-6.728394725581	-2.073473622625
C	2.757158894861	-7.156159904561	-1.026192392638
F	2.038030460589	-4.693064189033	1.489857873944
F	3.574940967256	-6.835978351350	1.157575004237
F	3.505598329075	-8.245591797422	-1.163054595367
F	1.955855169284	-7.393553778465	-3.230785601463
F	0.376804190869	-5.244377831077	-2.935522963696
C	-1.520068966136	3.890237748421	-2.852872419085
C	-2.744906980896	4.165613357739	-2.243220350086
C	-0.635997733755	4.953592659621	-3.034783655282
C	-3.104246815344	5.453233388777	-1.874160838483
F	-3.630138663881	3.196172960204	-2.002984166862
C	-0.979372484175	6.246531066340	-2.653314842850
F	0.564553244986	4.796495146472	-3.590474189198
C	-2.222810880616	6.502436873693	-2.094626496139
F	-4.296093029664	5.685823808752	-1.316184591285
F	-0.117079055342	7.251045325988	-2.832817040525
F	-2.563926268046	7.746994503690	-1.766485382819
O	-2.743976228543	-3.190753324900	-2.688278075706
H	-2.140373237818	-3.607002890232	-1.896702459897

C	-2.701930284983	-1.928707742863	-2.725815277592
H	-2.174443303776	-1.394383296682	-1.924508591155
C	-3.189084533660	-1.175802201145	-3.848308043642
H	-3.258693826111	-0.125746446227	-3.553513470625
C	-4.419470177619	-1.616563889275	-4.717051663504
C	-5.127122791659	-2.907787683244	-4.299329303426
H	-4.466685798930	-3.775533655868	-4.320500616697
H	-5.546730780189	-2.822970072013	-3.291102044983
H	-5.959265103275	-3.090992476689	-4.989236418920
C	-5.450669251149	-0.478398856775	-4.697098020107
H	-5.868999271169	-0.365351643520	-3.690267460045
H	-5.017146486841	0.483557801867	-4.982136651274
H	-6.276750383698	-0.705826696441	-5.380165301042
C	-2.716924668989	-0.623189337065	-6.117486639368
H	-2.089437899730	-0.606773555560	-7.013245998777
H	-3.111998723036	0.373138144364	-5.921771720922
C	-3.754459391399	-1.758241809867	-6.145257463515
H	-4.494582379533	-1.748864477575	-6.950604801193
C	-2.838392107017	-2.962554750273	-6.094923295510
H	-3.069850711167	-3.940338710608	-6.503291644857
C	-1.936400000793	-1.209498224785	-4.929164615513
H	-1.063509142599	-0.671422794025	-4.560416984319
C	-1.766120254953	-2.636186756092	-5.361006921529
H	-0.954129737035	-3.282463432436	-5.048543417034

4b

O	-3.94688896151172	3.25567154704082	-0.10950416553766
C	-3.92492383982512	2.81215379250471	-1.23606527996053
H	-3.90243049490443	3.50153646115667	-2.11388672299924
C	-3.93028326394387	1.34980144683150	-1.56730386770314
H	-3.63930807674108	0.81058387436466	-0.65826828279124
C	-5.33540591834335	0.80532903529540	-2.06342934194583
C	-6.40147455445553	1.89054506754639	-2.25155824737503

H	-7.31731082765205	1.44267548305000	-2.65493590436738
H	-6.65448464686758	2.35806457478717	-1.29327810626145
H	-6.07993236414005	2.67618895065791	-2.94011827563936
C	-5.88122476428780	-0.22842140754746	-1.07307509350362
H	-5.19095110417264	-1.06298900747366	-0.92063825019983
H	-6.06395573076494	0.23710078309833	-0.09811854568432
H	-6.83289038095260	-0.63497218486930	-1.43509672859567
C	-3.52588110360075	-0.42171971003884	-3.09952691685764
H	-3.50321722941907	-1.12674402878250	-2.26289270390496
H	-3.03127238670507	-0.86051423428409	-3.97104037161654
C	-4.92850941188029	0.12614667252877	-3.43048596767911
H	-5.67870819064192	-0.58591935919577	-3.78619433859585
C	-4.55260422386454	1.23861956782964	-4.38971850758225
H	-5.18056847896373	1.61027322003435	-5.19185127086539
C	-2.96066911765929	0.96973486777744	-2.74551648809920
H	-1.90359655490036	1.02904605392261	-2.47544553549730
C	-3.38818045389659	1.75025026213693	-3.97554139996399
H	-2.87018422987458	2.61227564162429	-4.38100643676943

9. NMR Spectra

9.1. NMR Spectra of Enal Substrates 1

^{1}H NMR spectrum of 1c

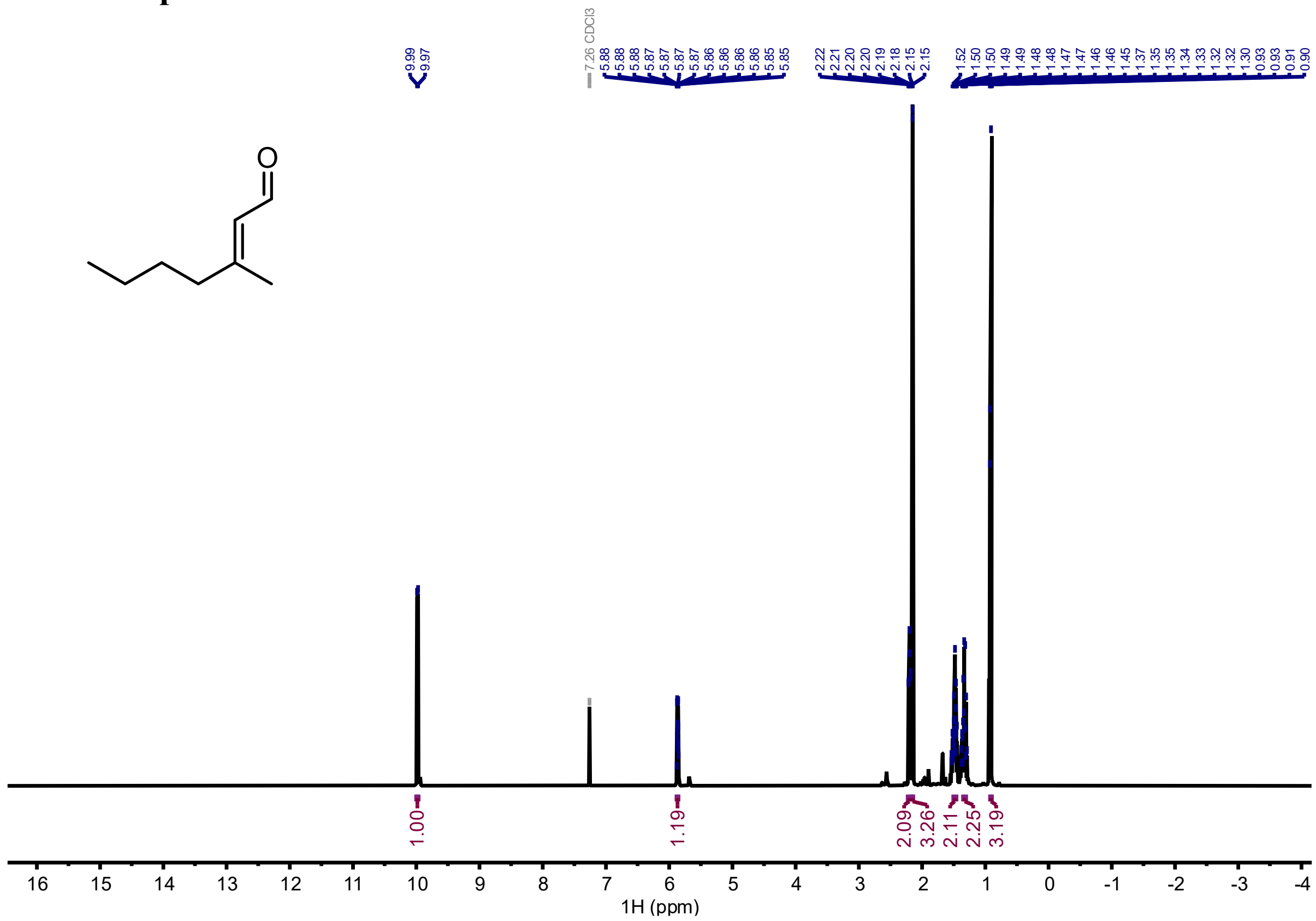

^{1}H NMR spectrum of 1d

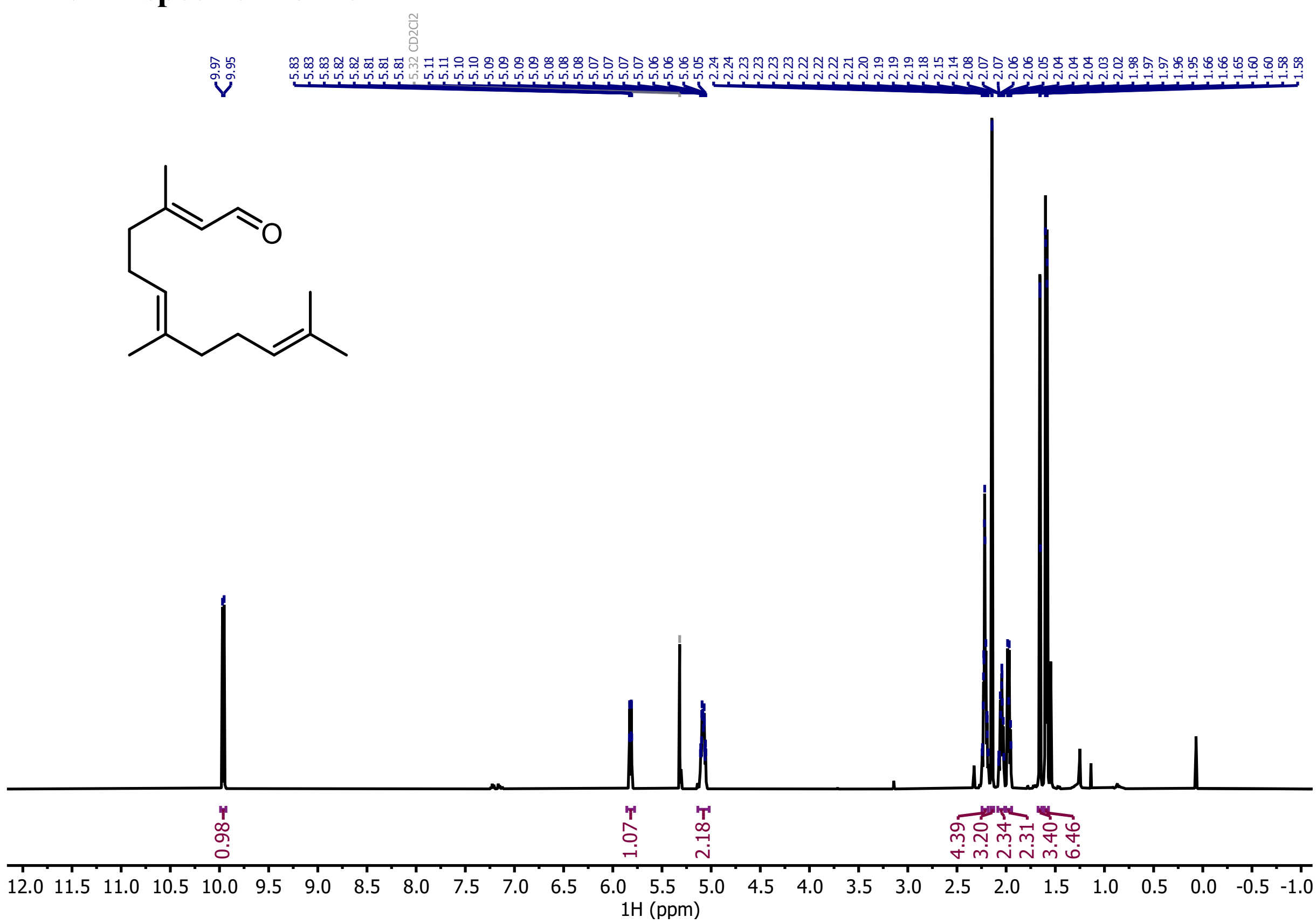

^{1}H NMR spectrum of 1e

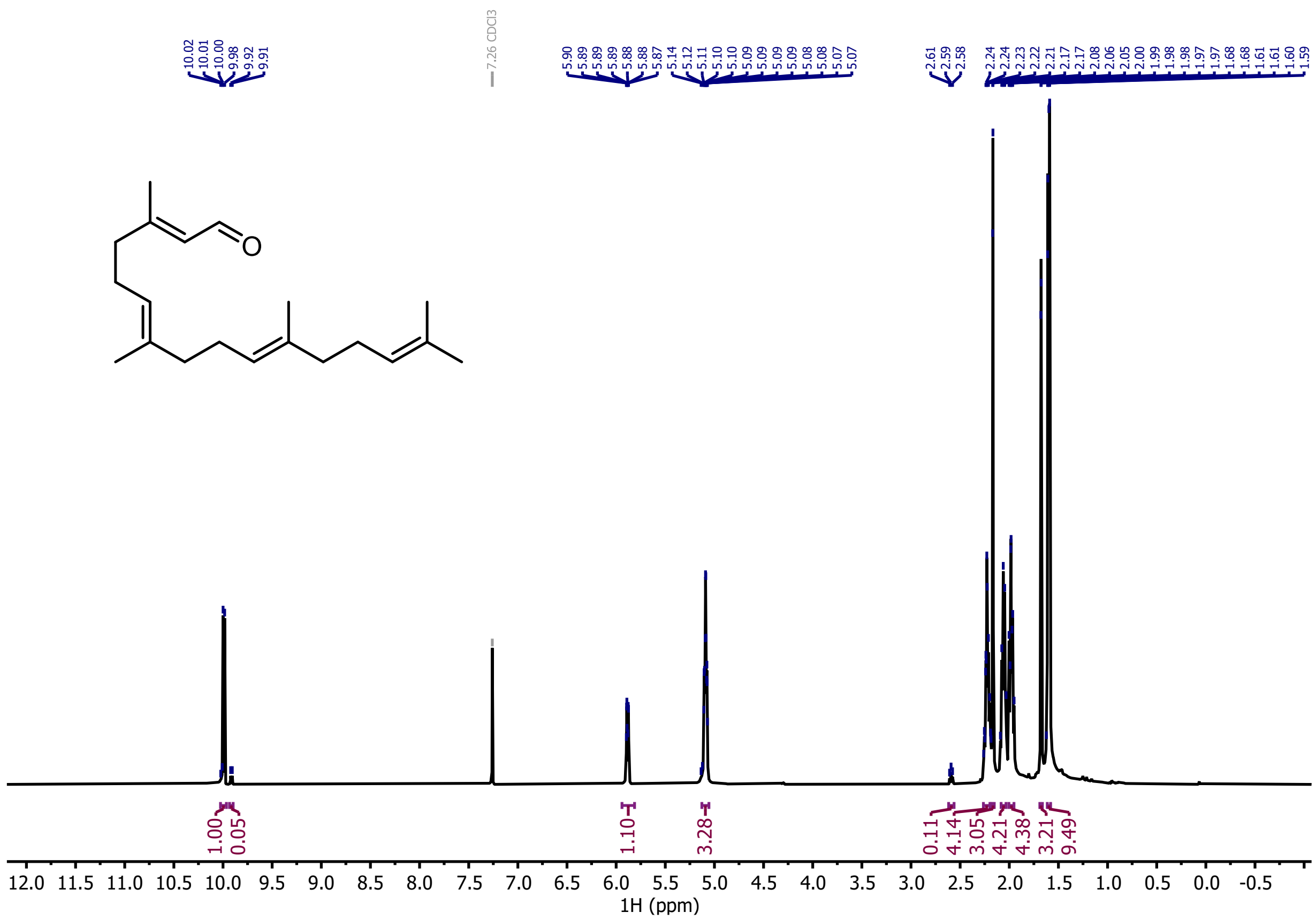

^{1}H NMR spectrum of 1f

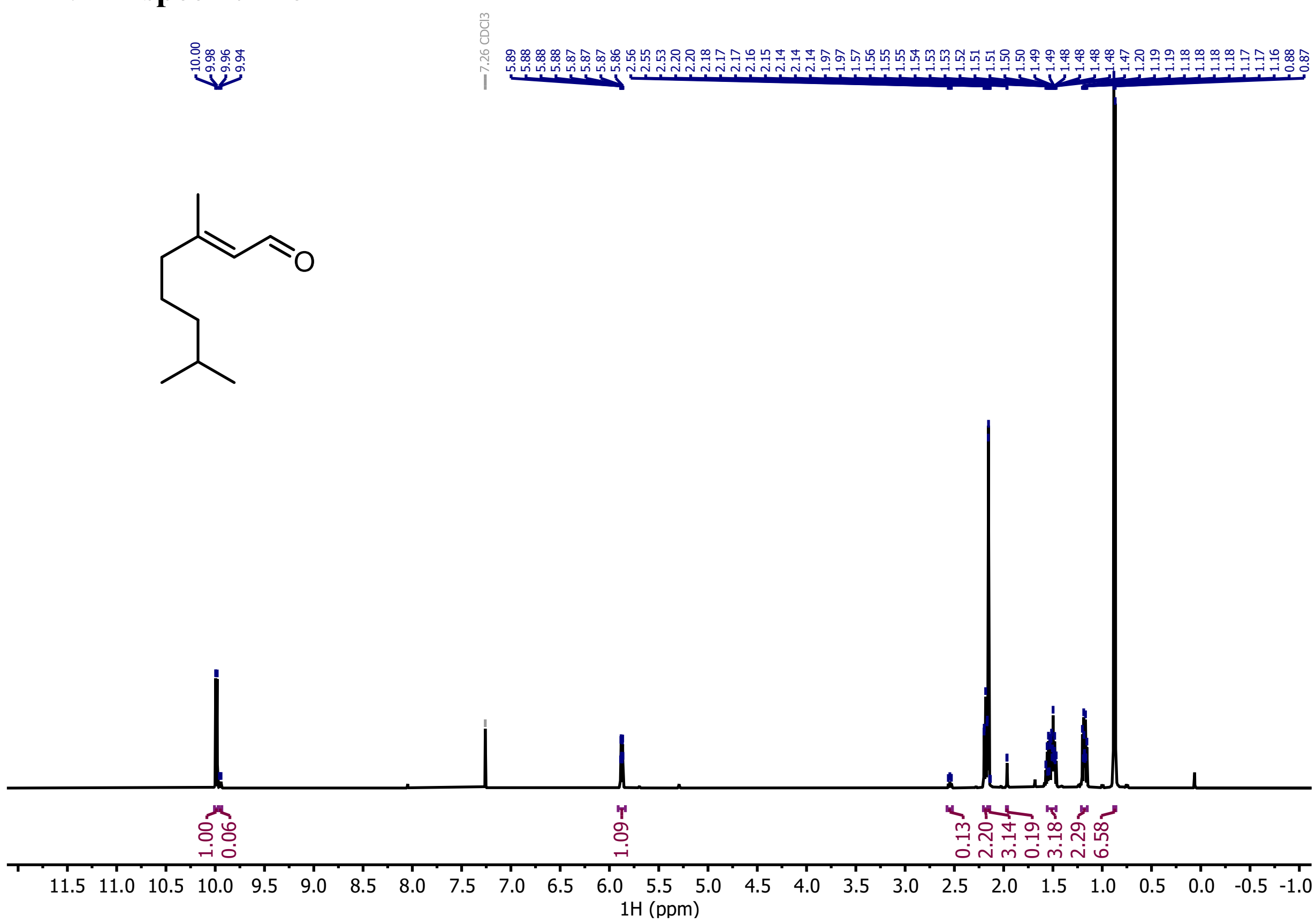

^{1}H NMR spectrum of 1h

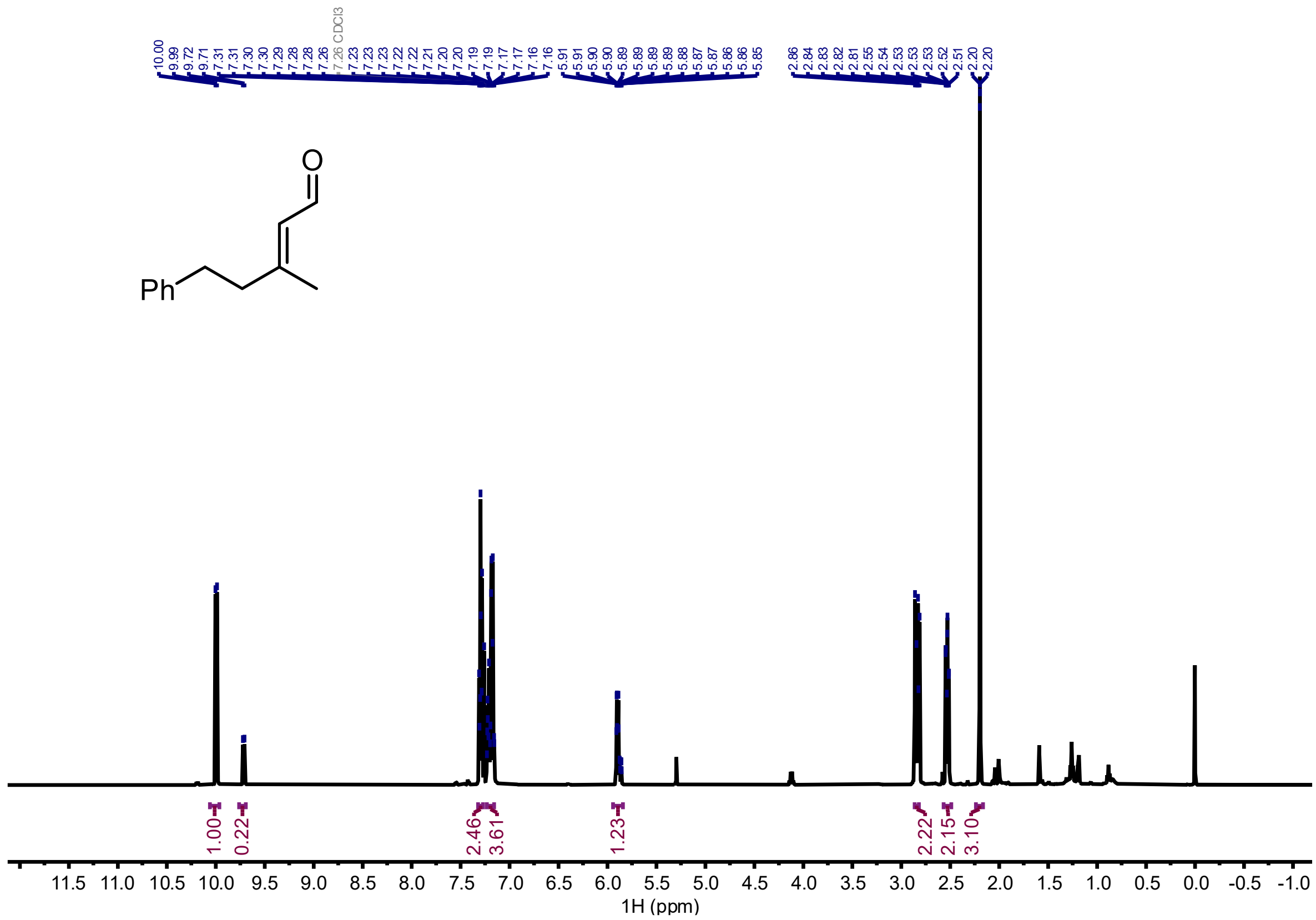

^{1}H NMR spectrum of 1i

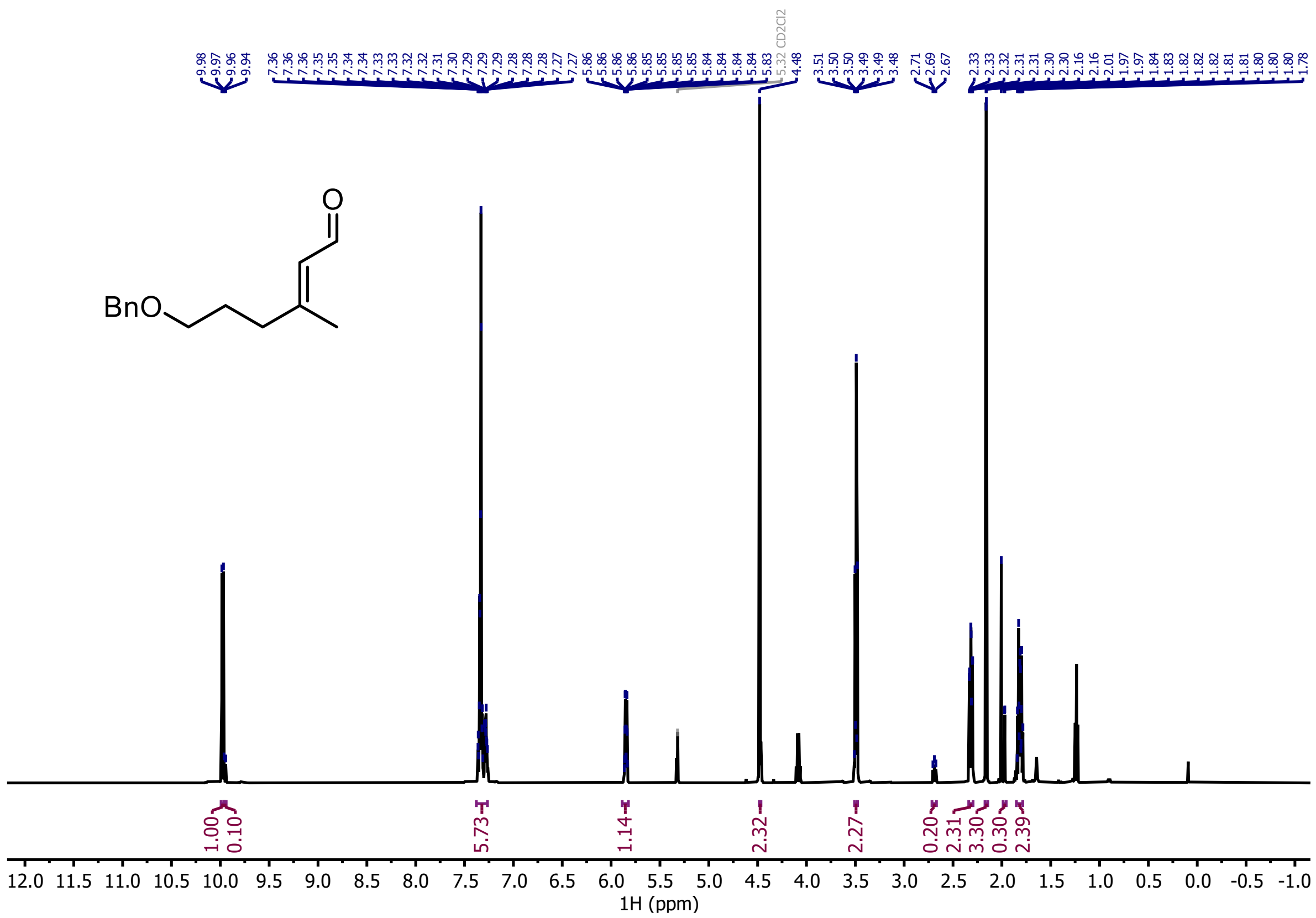

^{13}C NMR spectrum of 1i

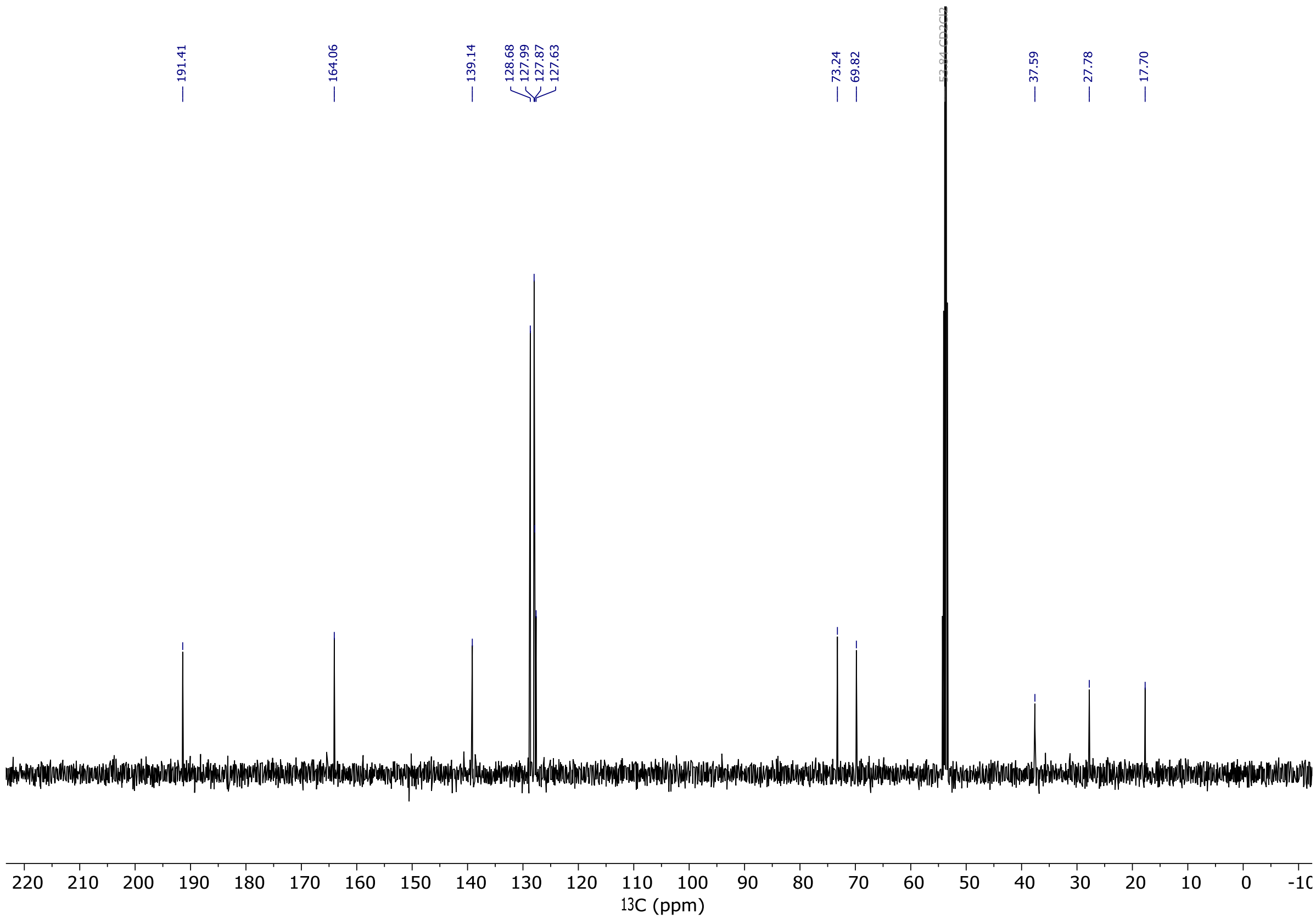

^{1}H NMR spectrum of 1j

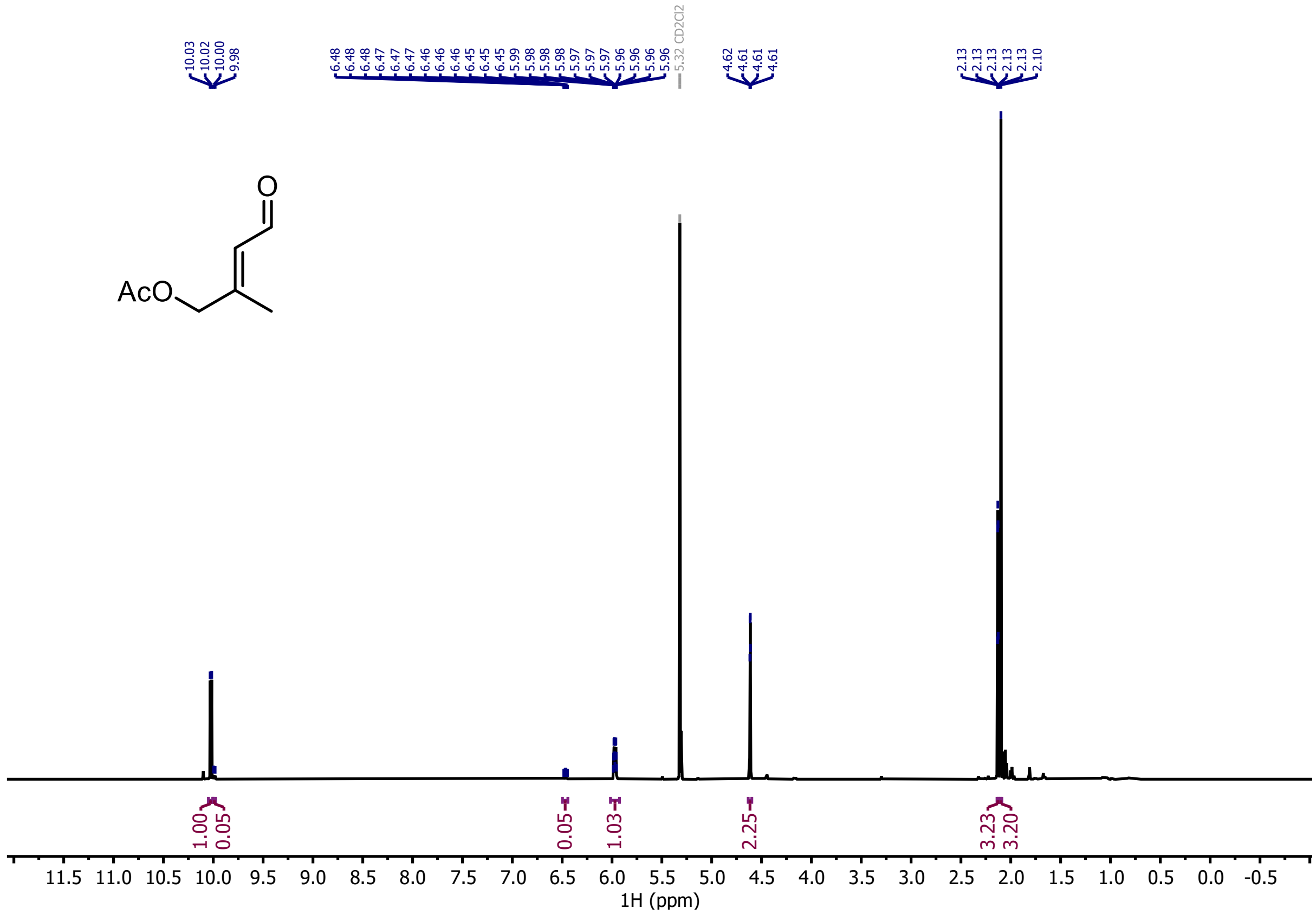

^{1}H NMR spectrum of 1k

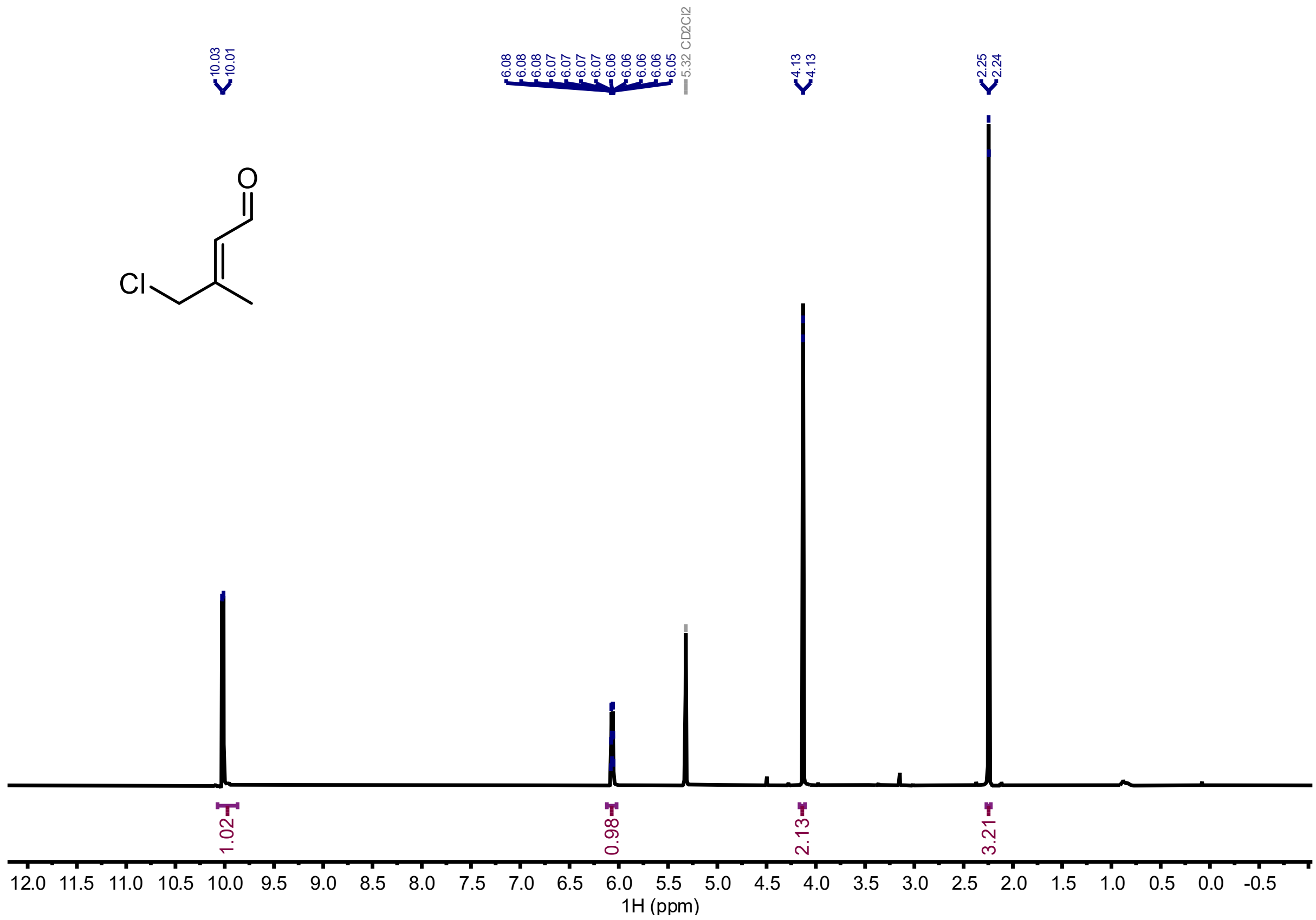

9.2. NMR Spectra of Diels−Alder Products 4

^{1}H NMR spectrum of 4a

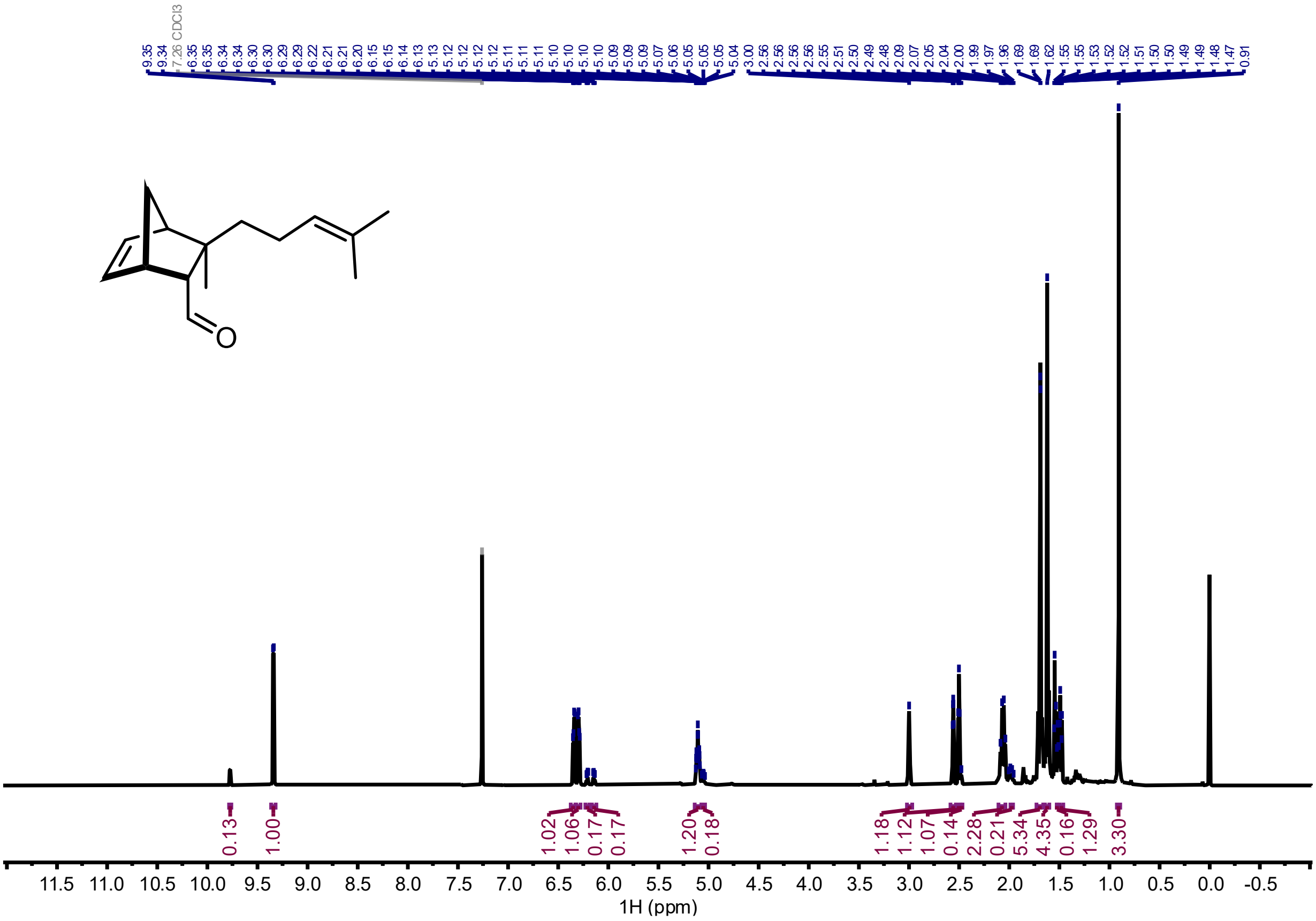

^{13}C NMR spectrum of 4a

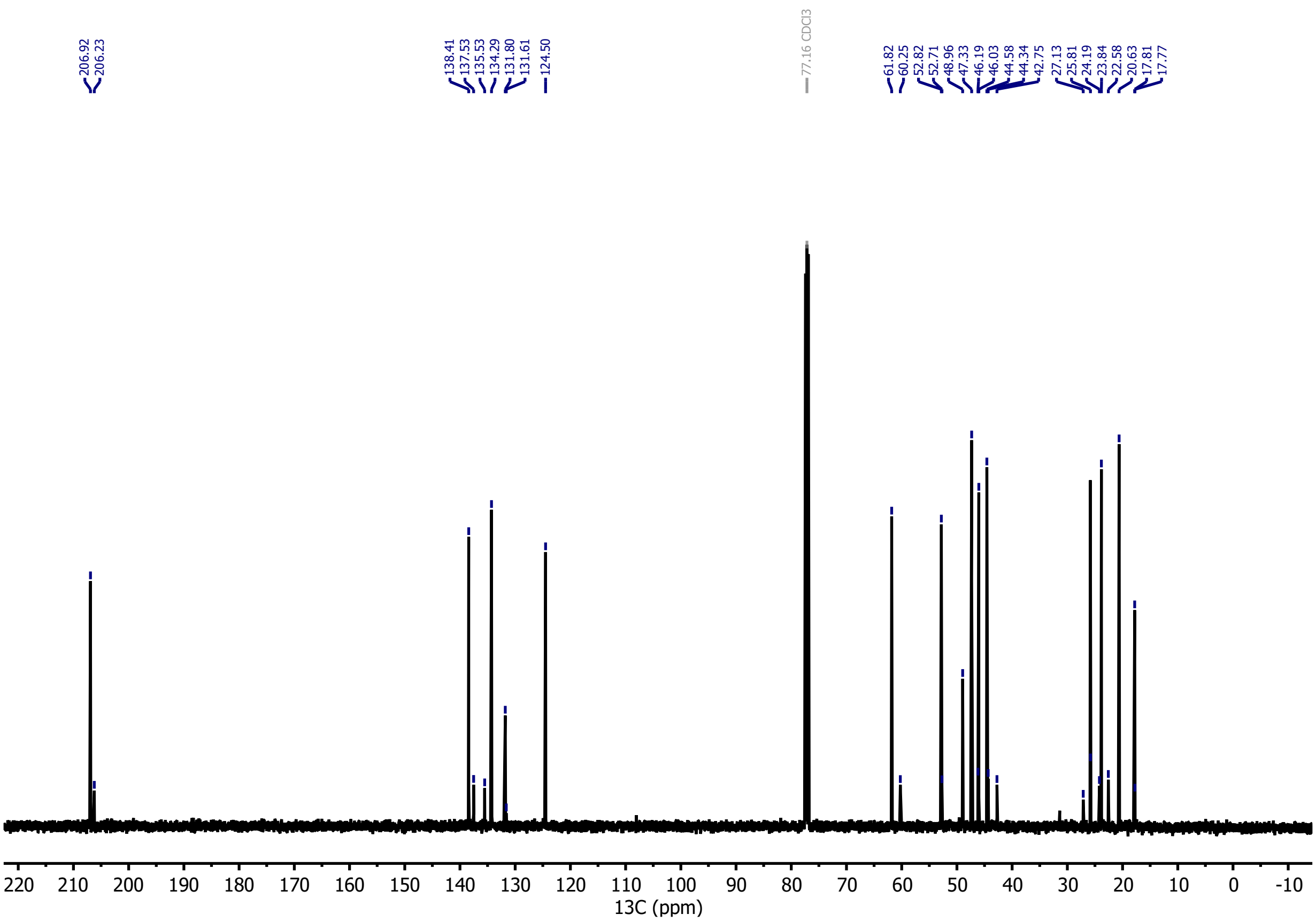

NOESY spectrum of 4a

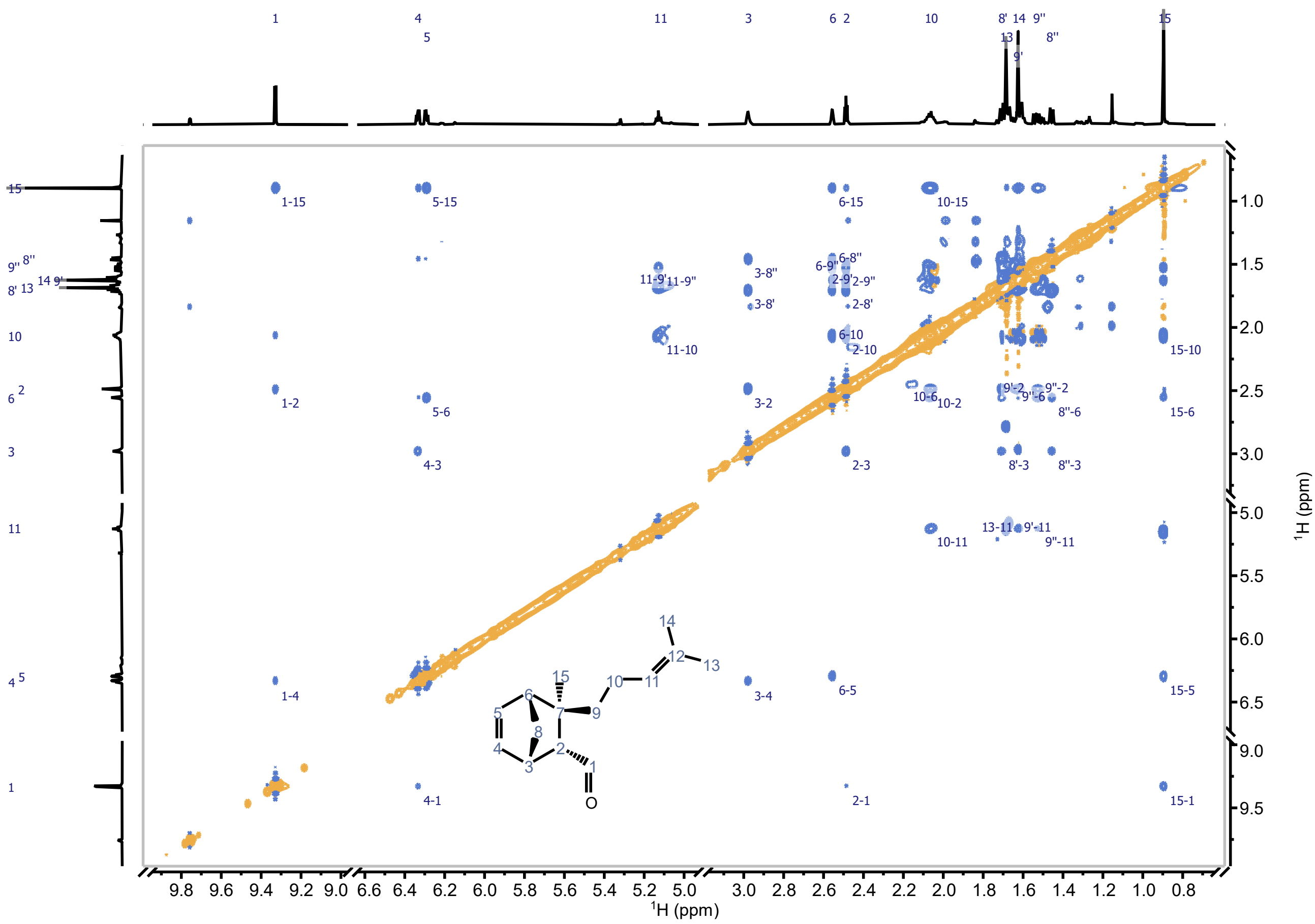

^{1}H NMR spectrum of 4a-S

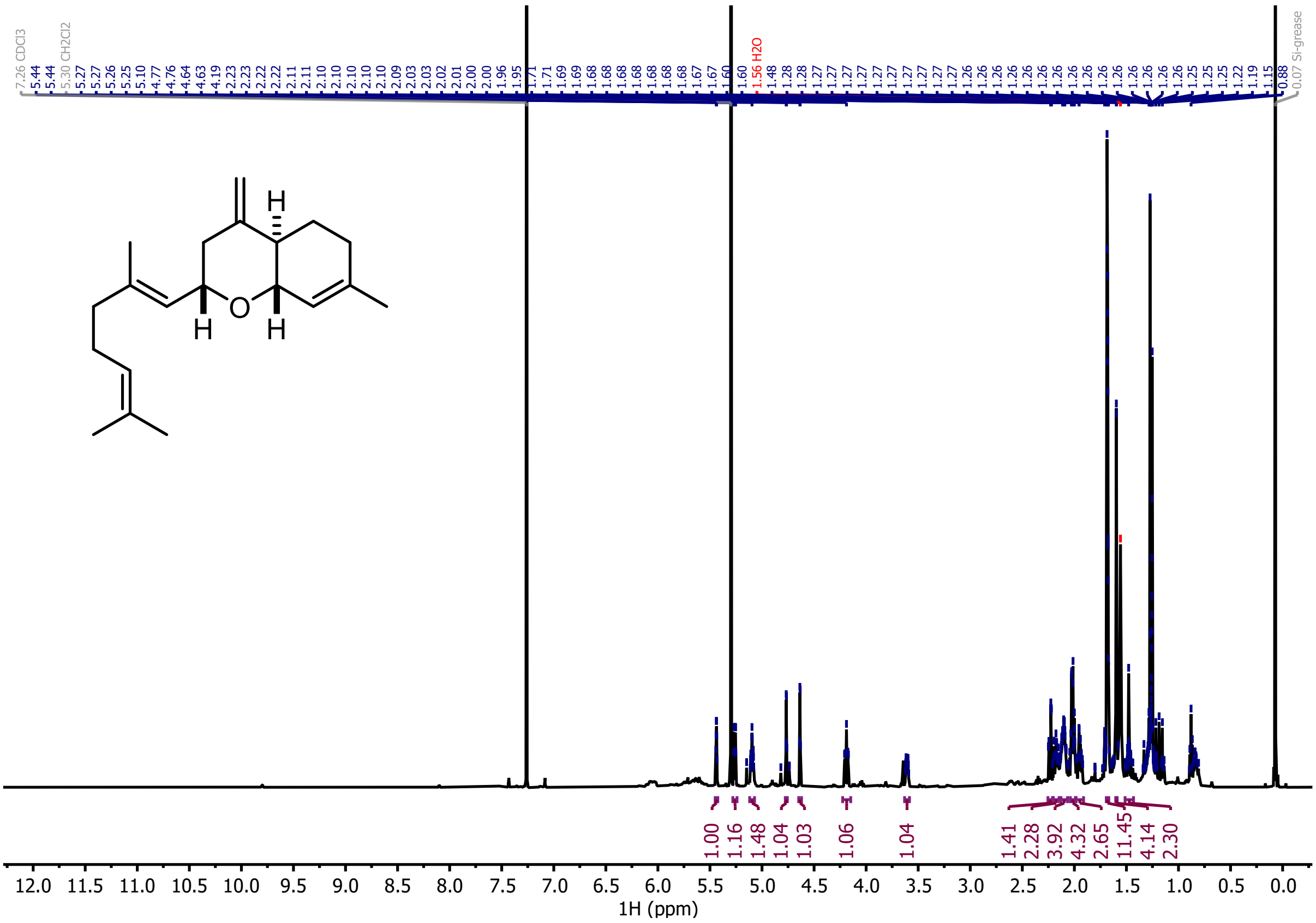

^{13}C NMR spectrum of 4a-S

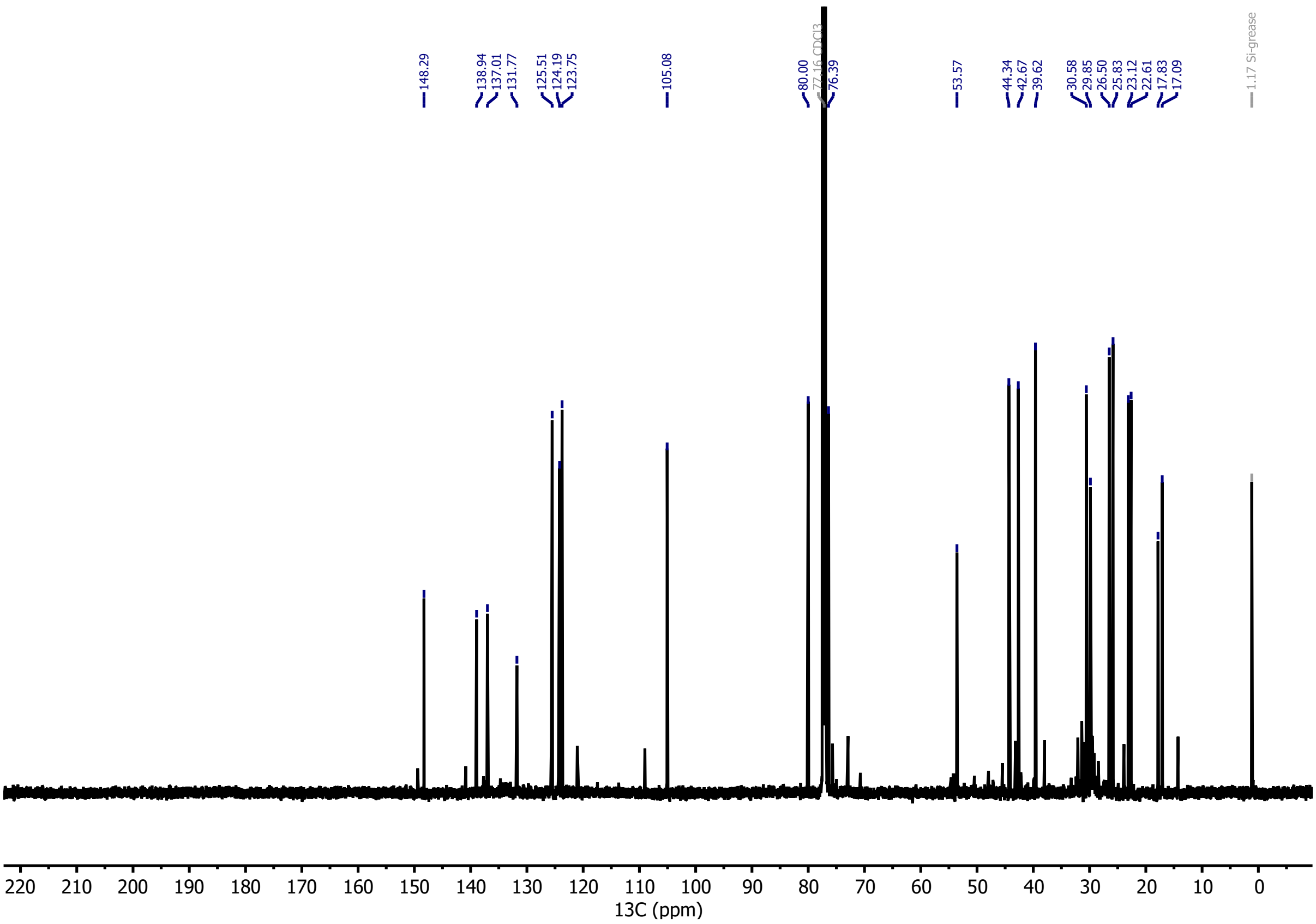

^{1}H NMR spectrum of 4b

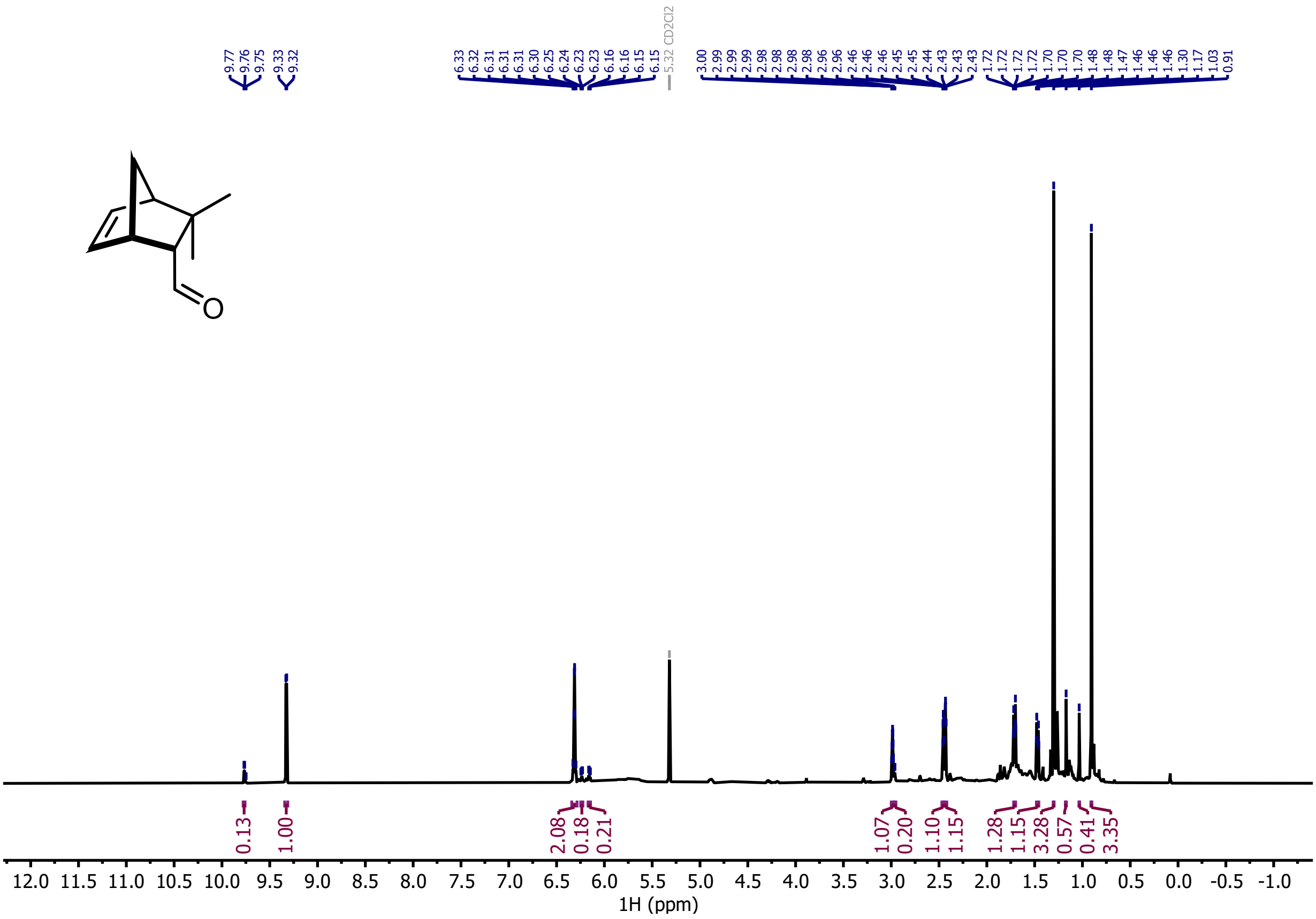

^{13}C NMR spectrum of 4b

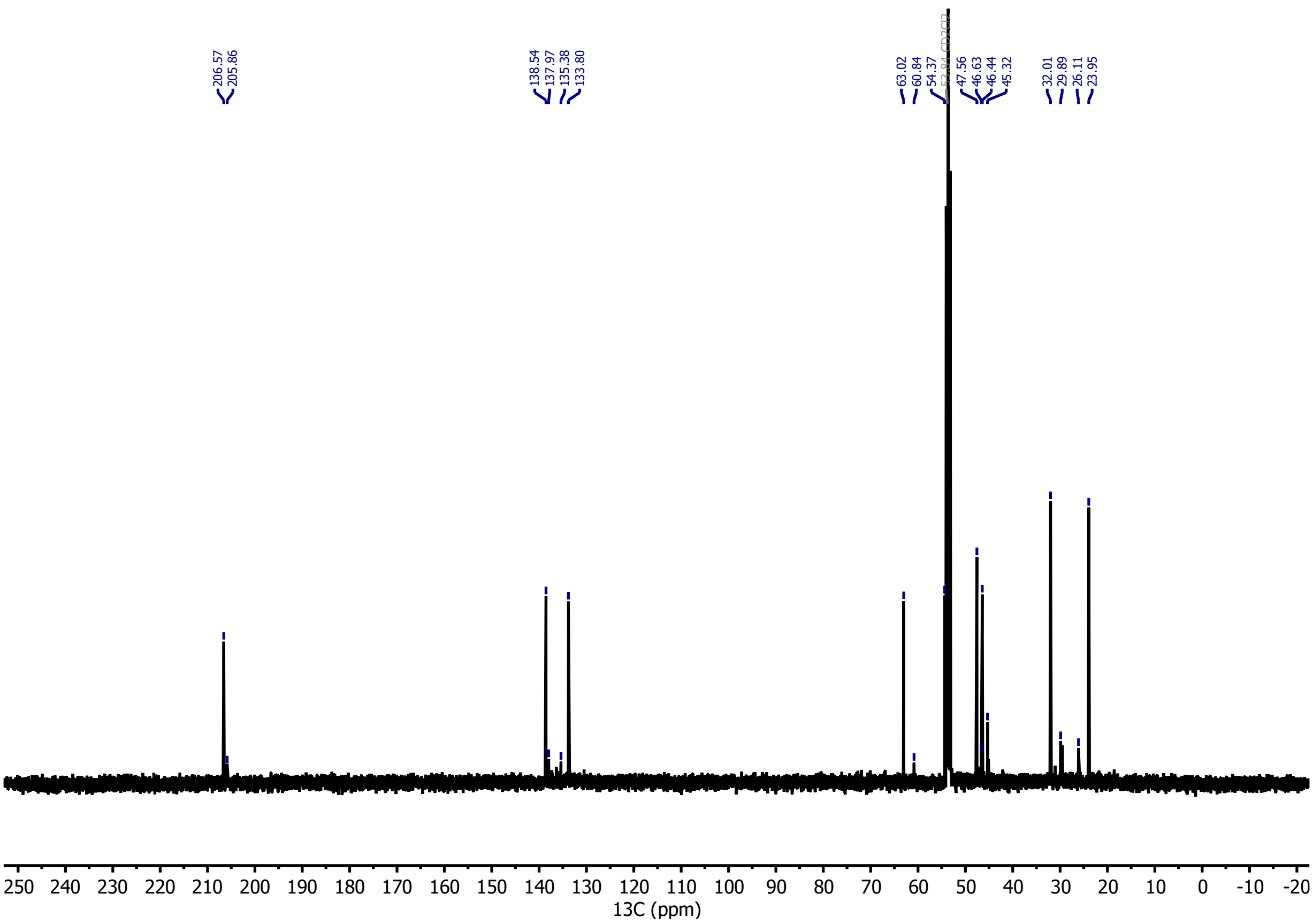

^{1}H NMR spectrum of 4c

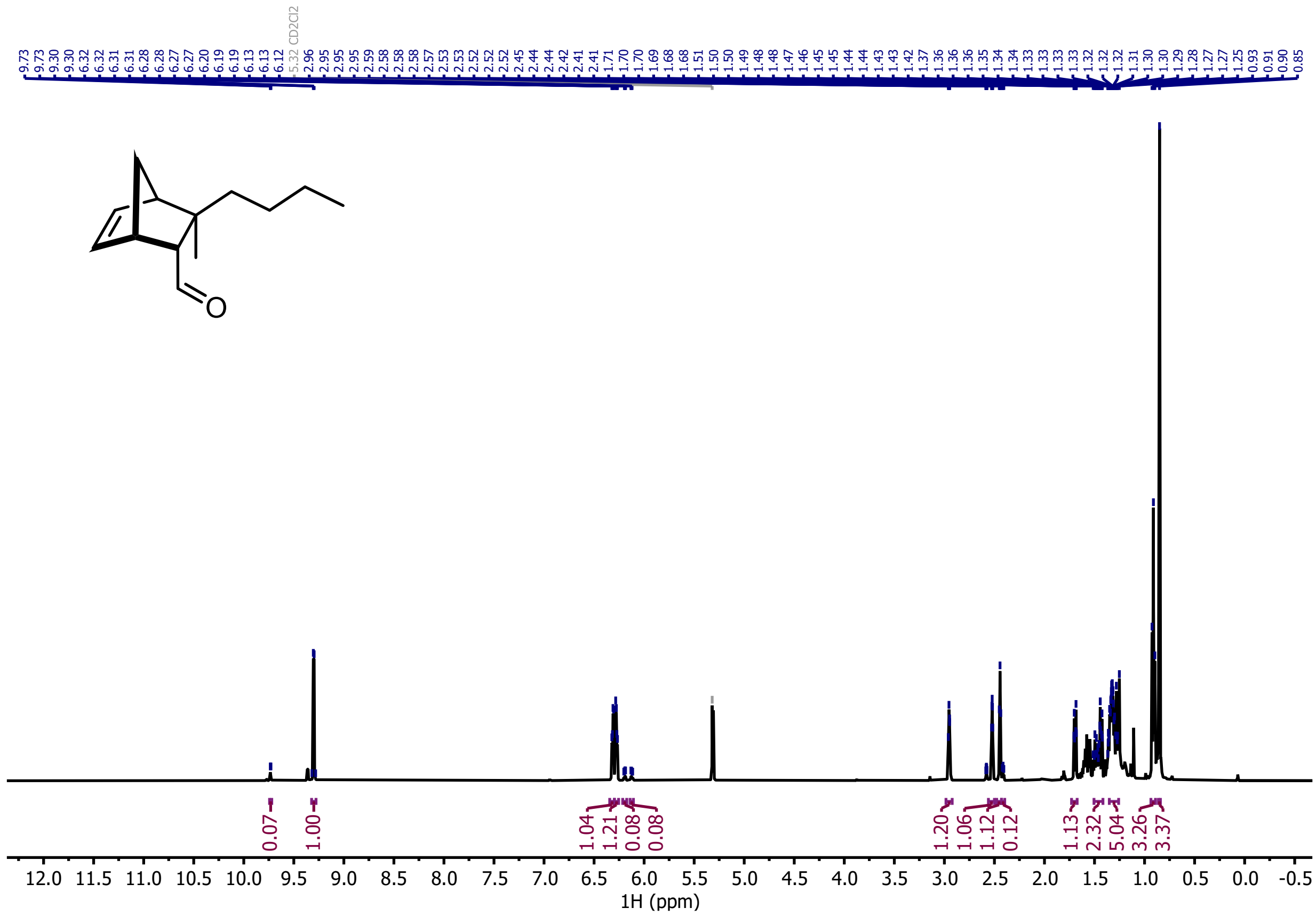

^{13}C NMR spectrum of 4c

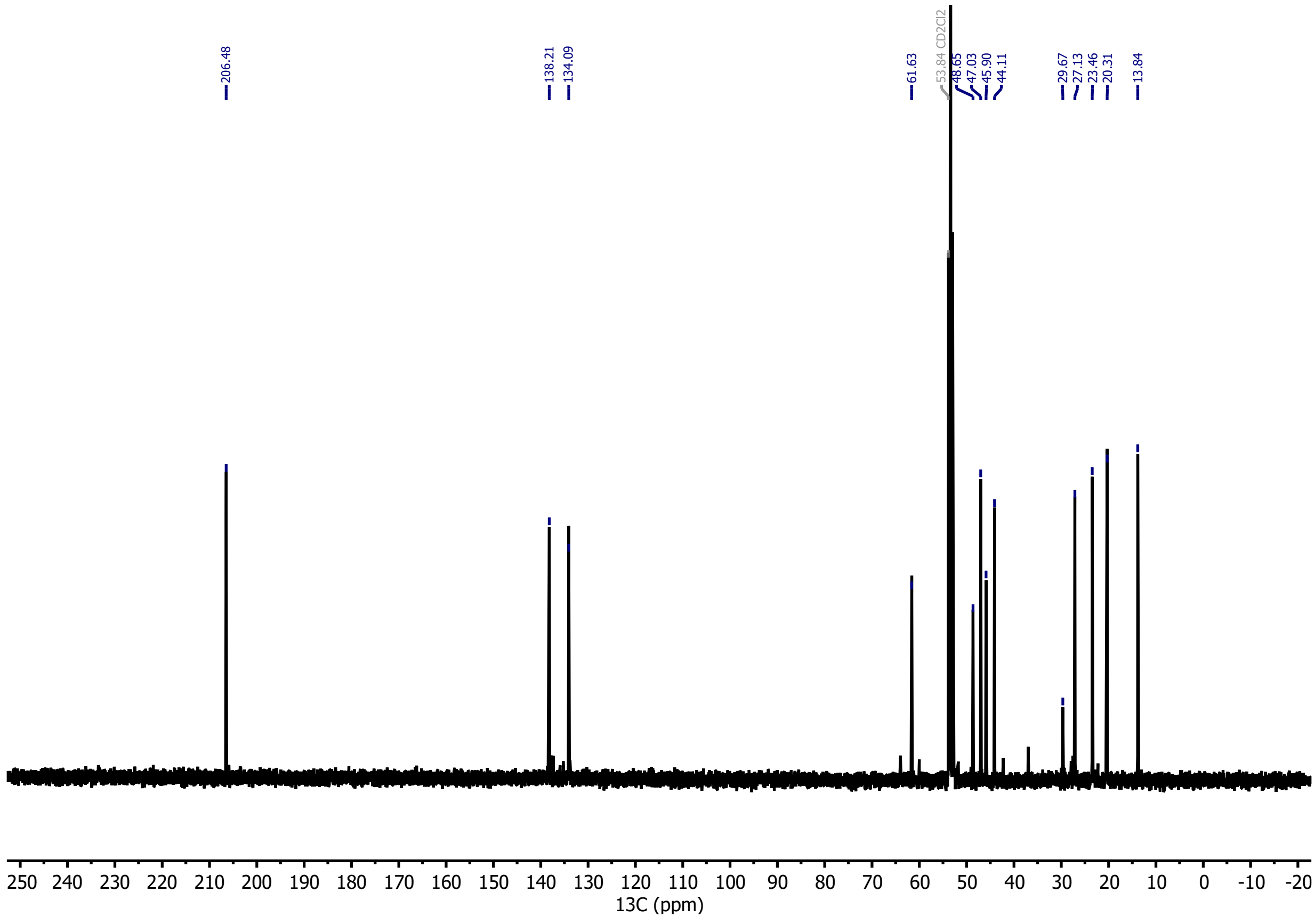

^{1}H NMR spectrum of 4d

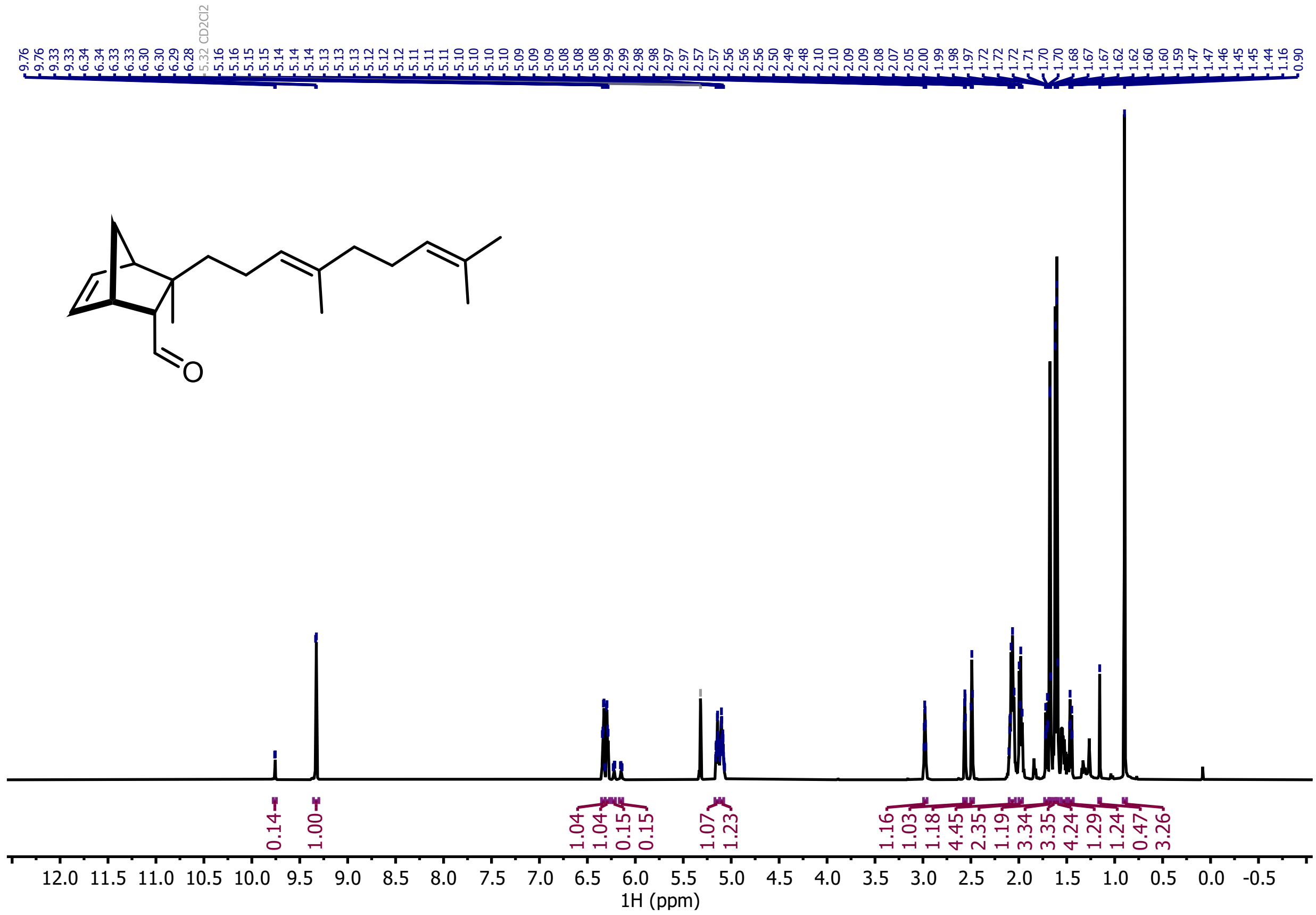

^{13}C NMR spectrum of 4d

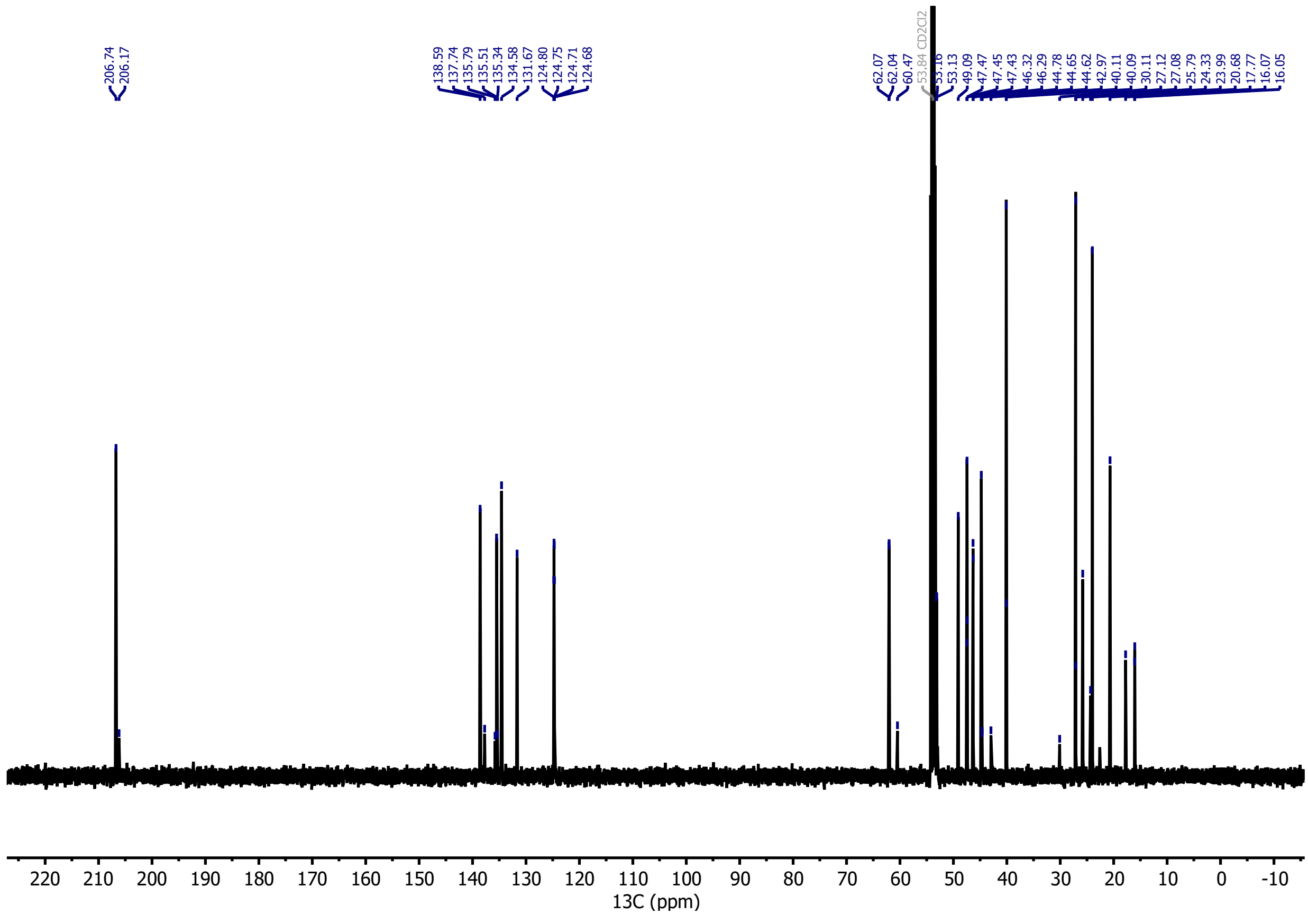

^{1}H NMR spectrum of 4e

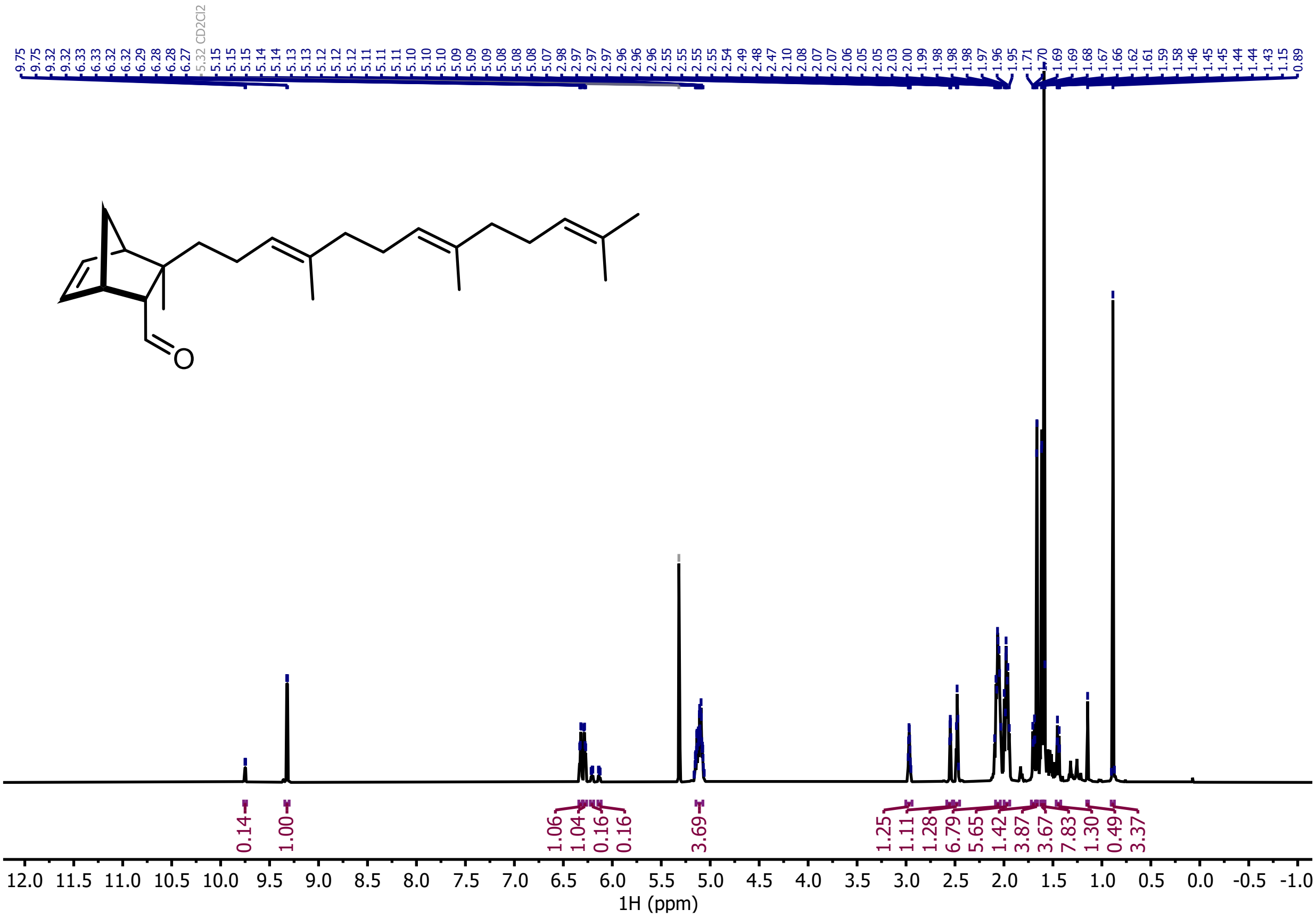

^{13}C NMR spectrum of 4e

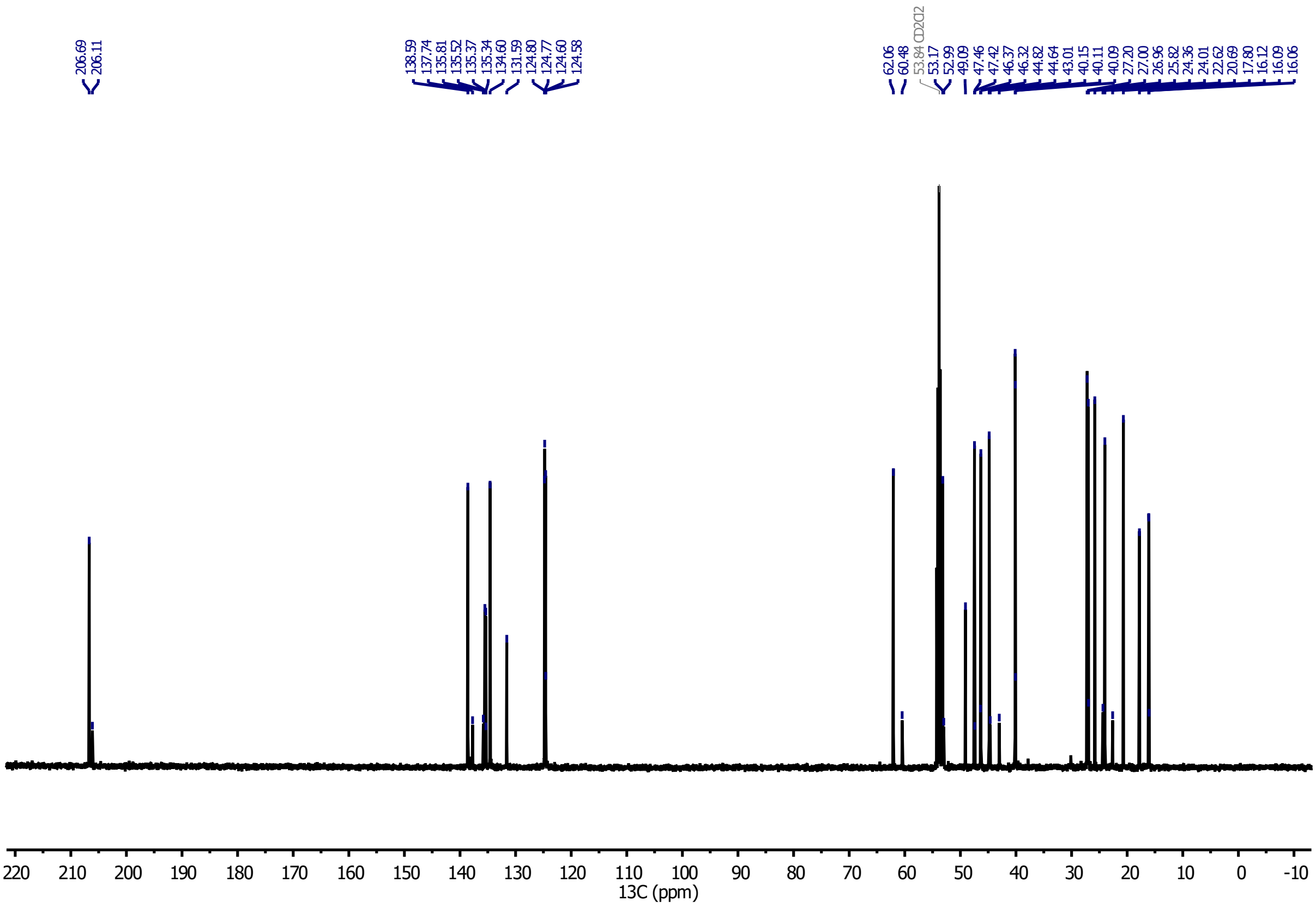

^{1}H NMR spectrum of 4f

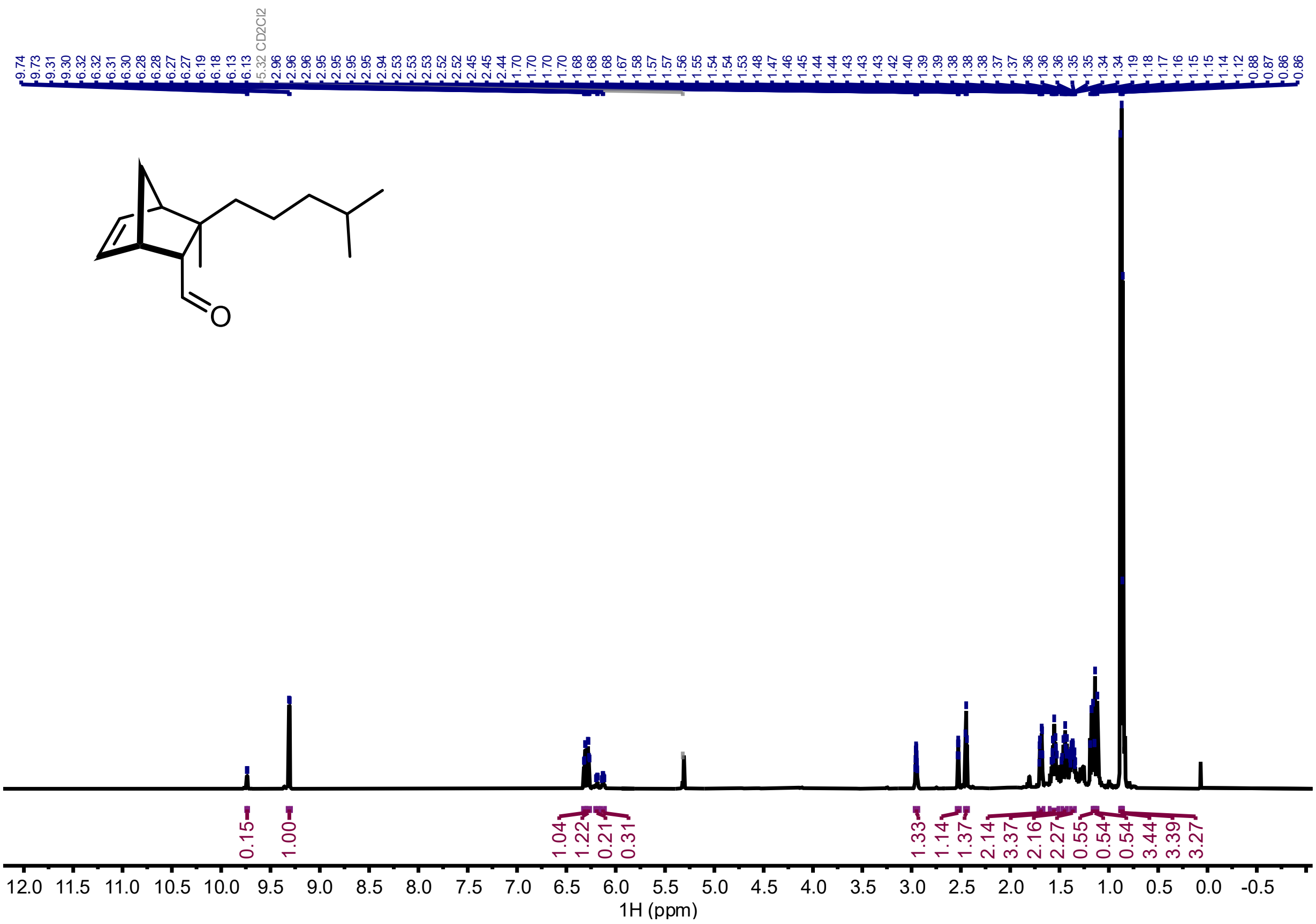

^{13}C NMR spectrum of 4f

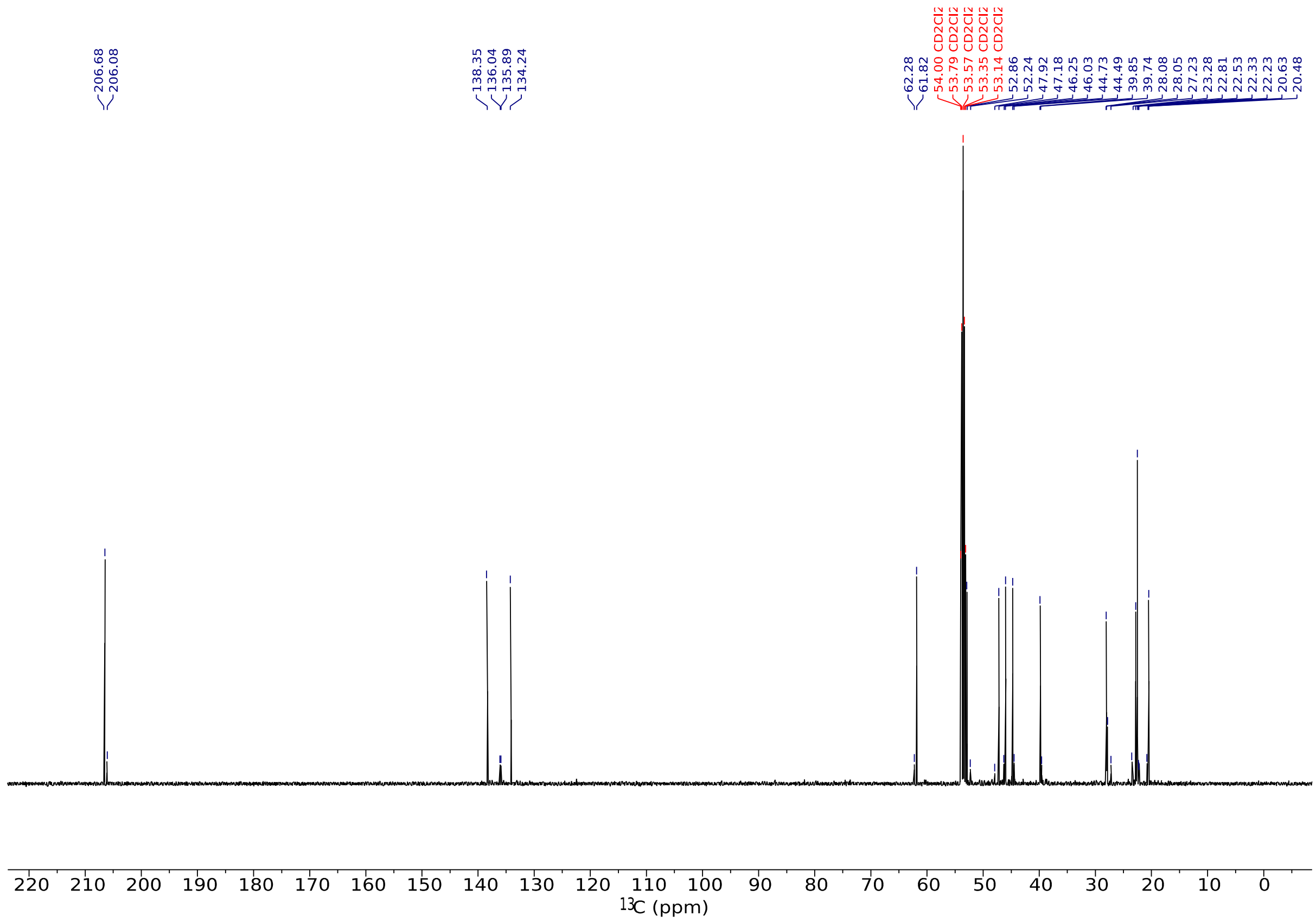

^{1}H NMR spectrum of 4g

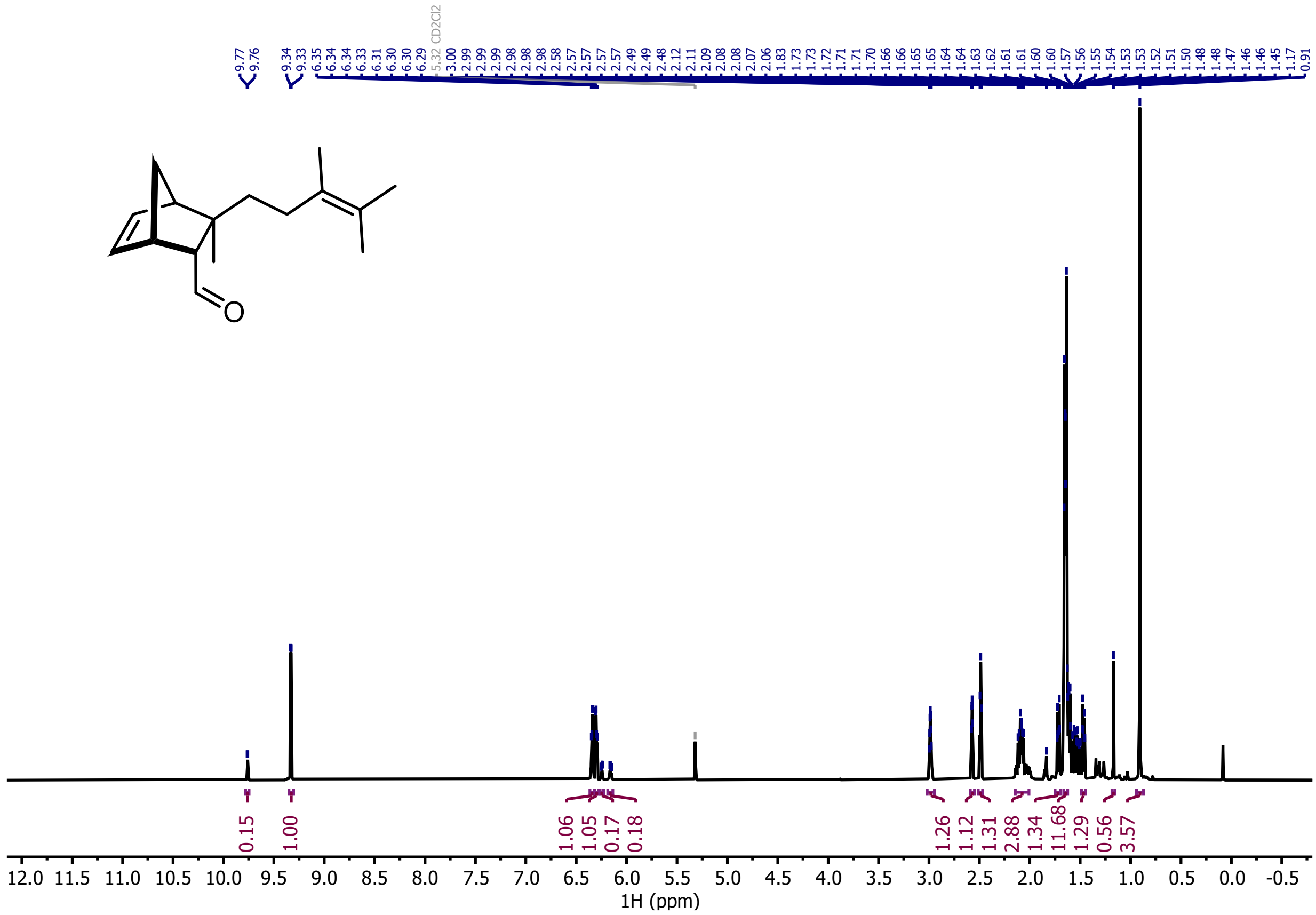

^{13}C NMR spectrum of 4g

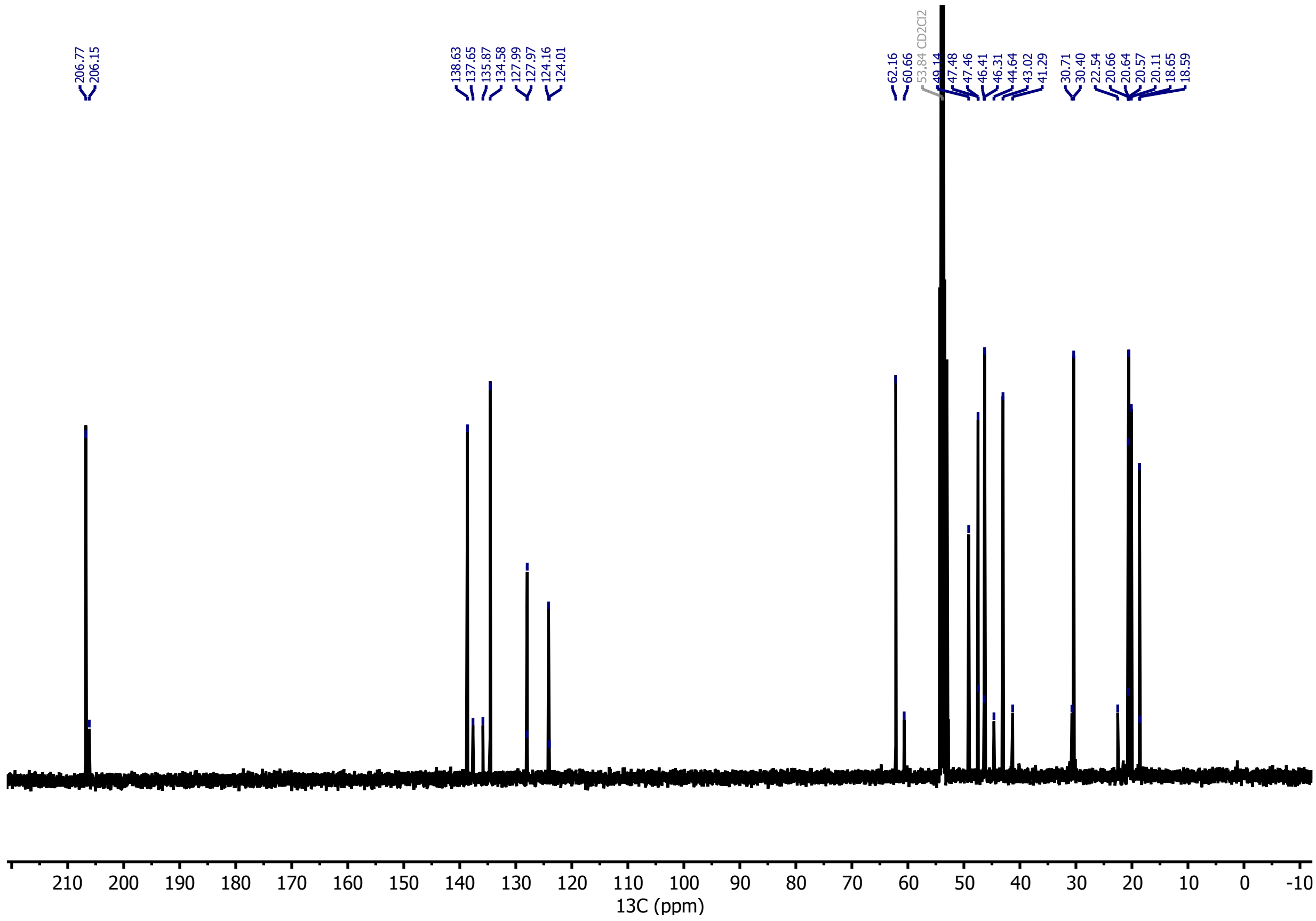

^{1}H NMR spectrum of 4h

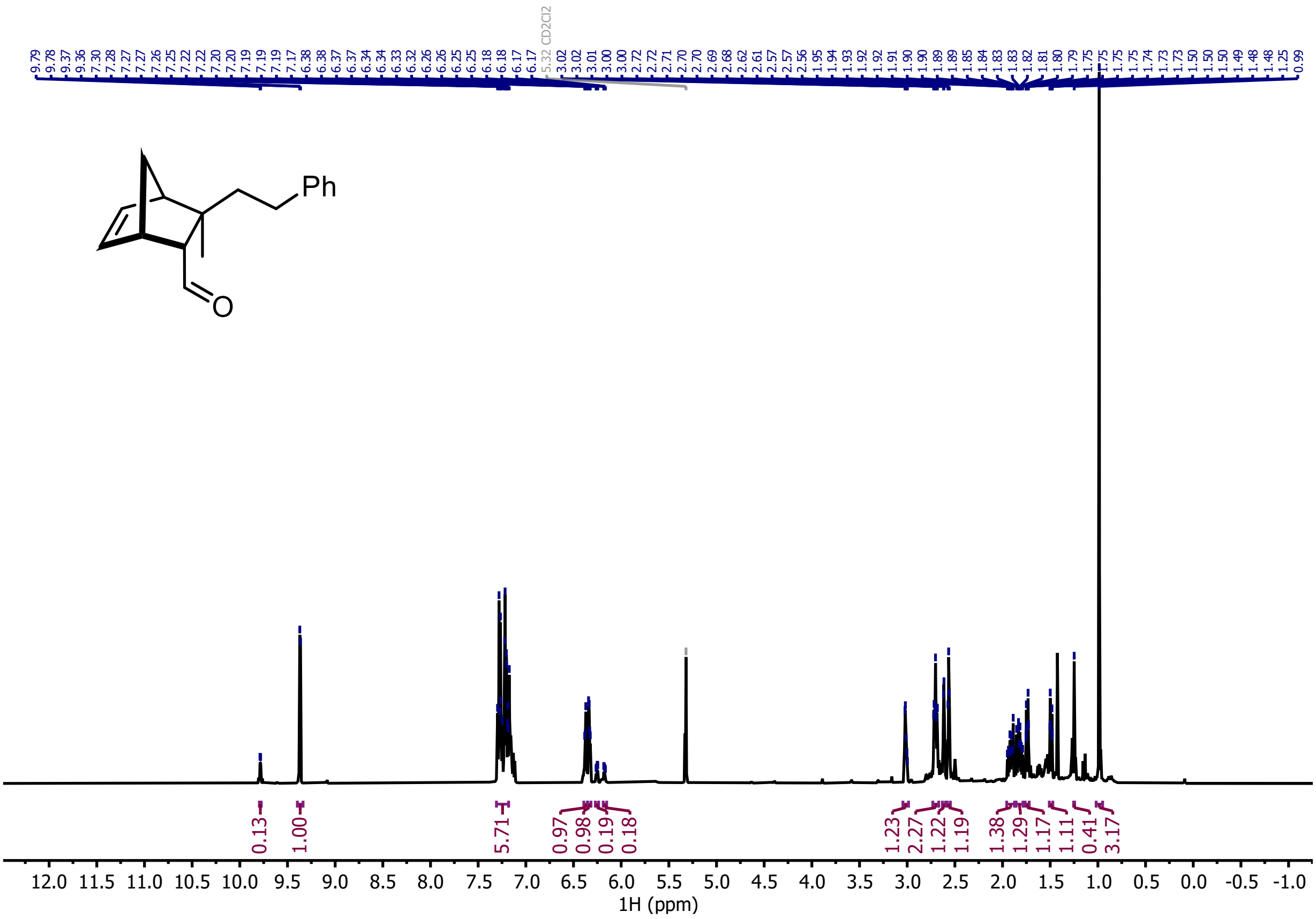

^{13}C NMR spectrum of 4h

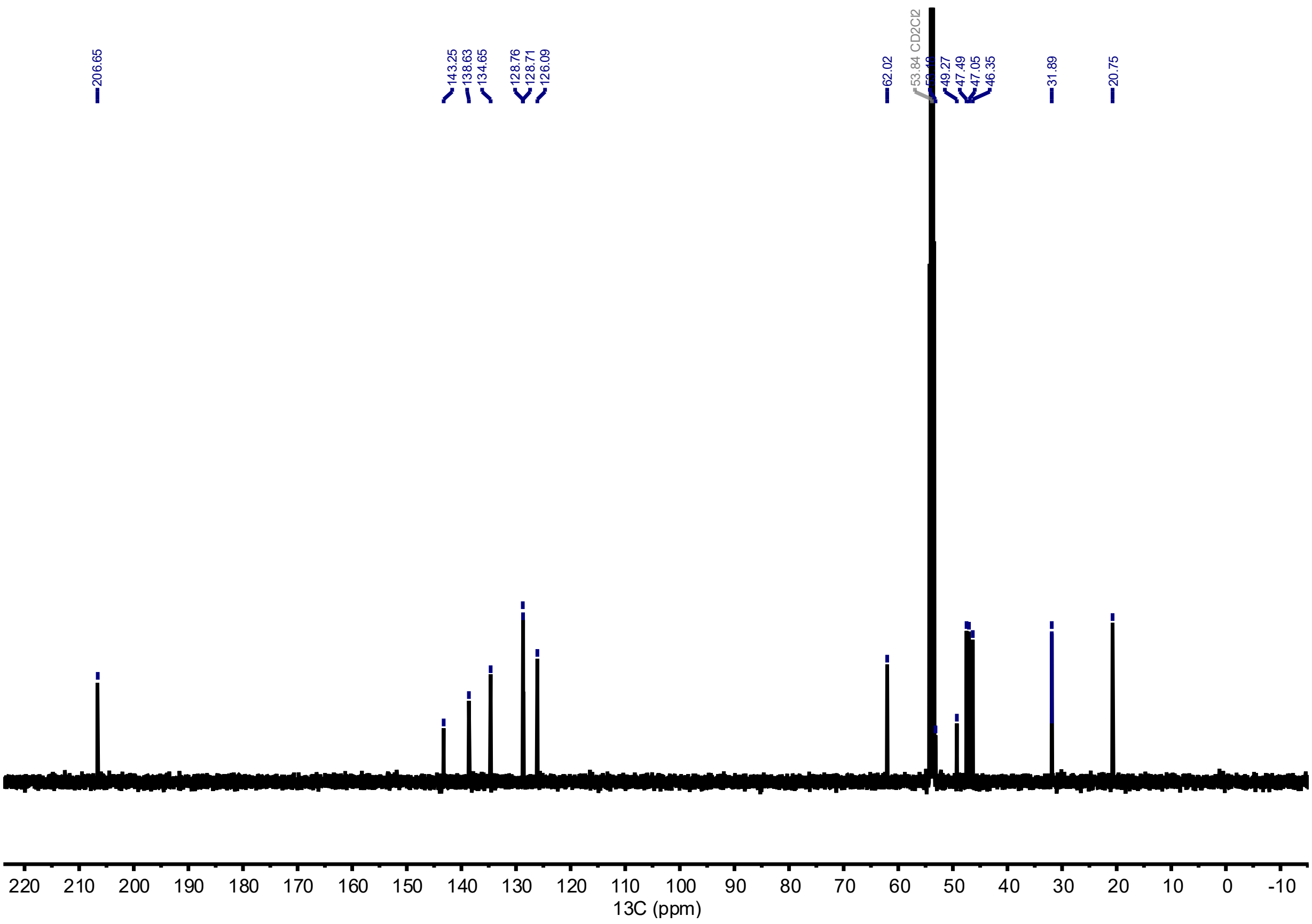

^{1}H NMR spectrum of 4i

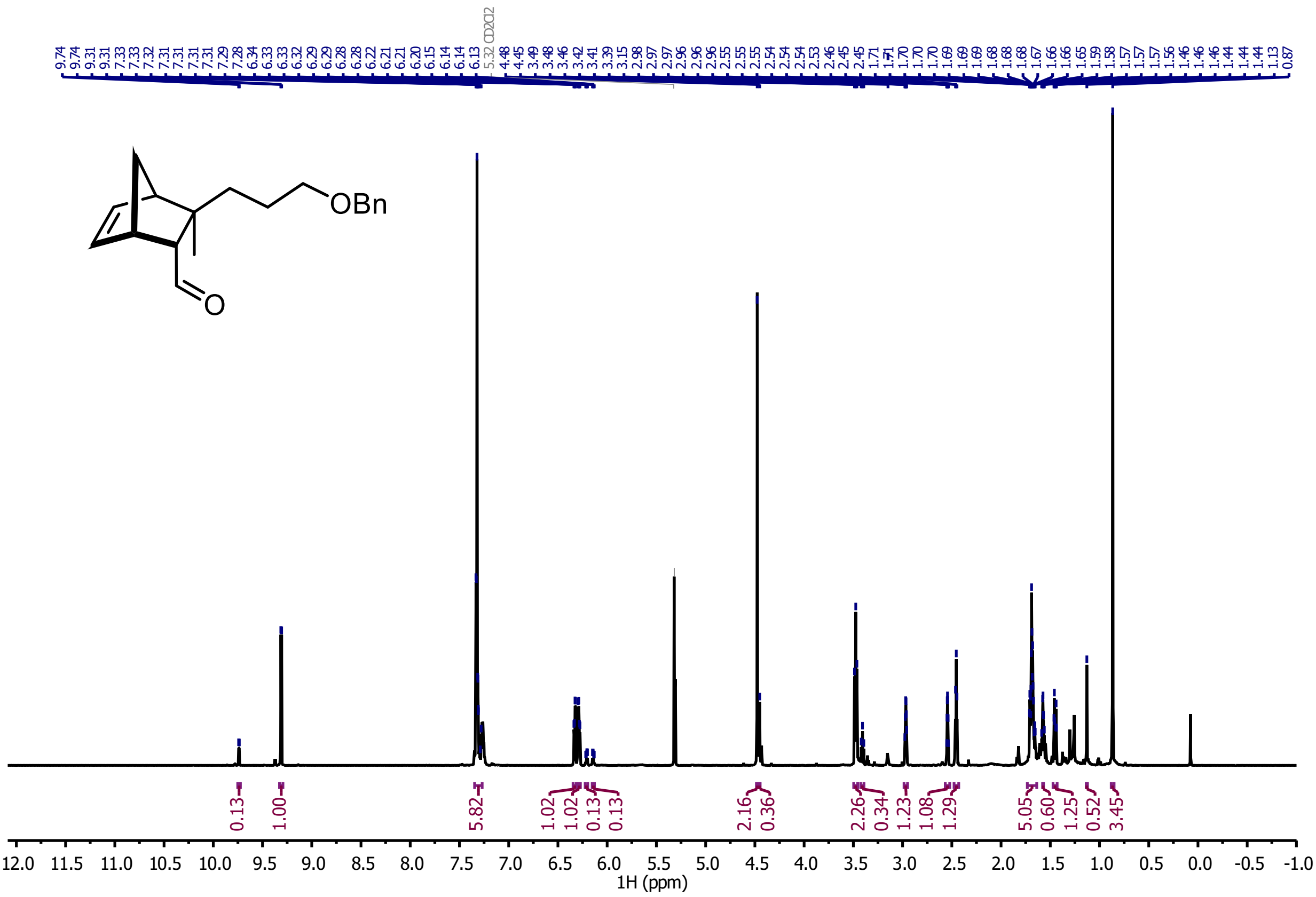

^{13}C NMR spectrum of 4i

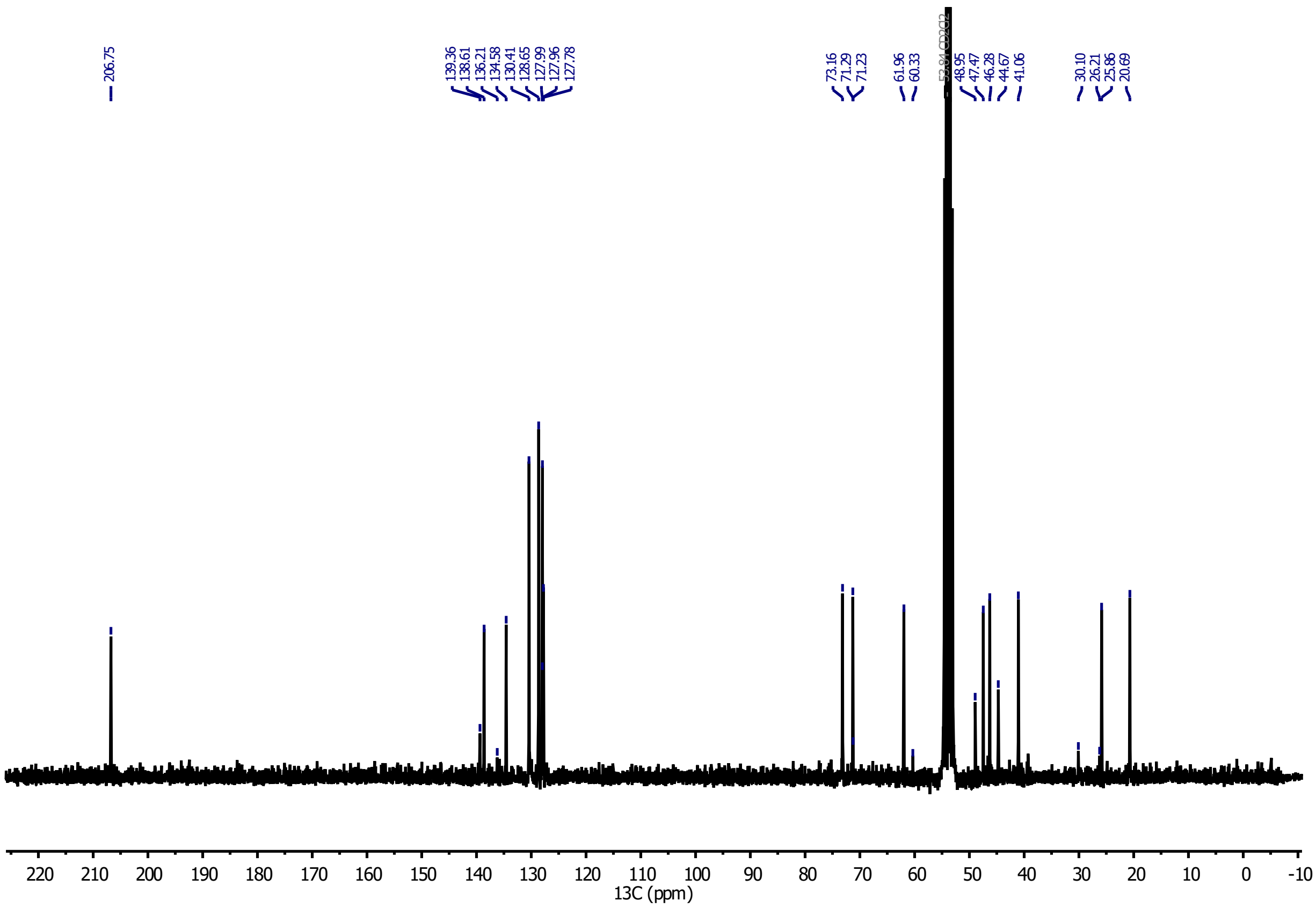

^{1}H NMR spectrum of 4j

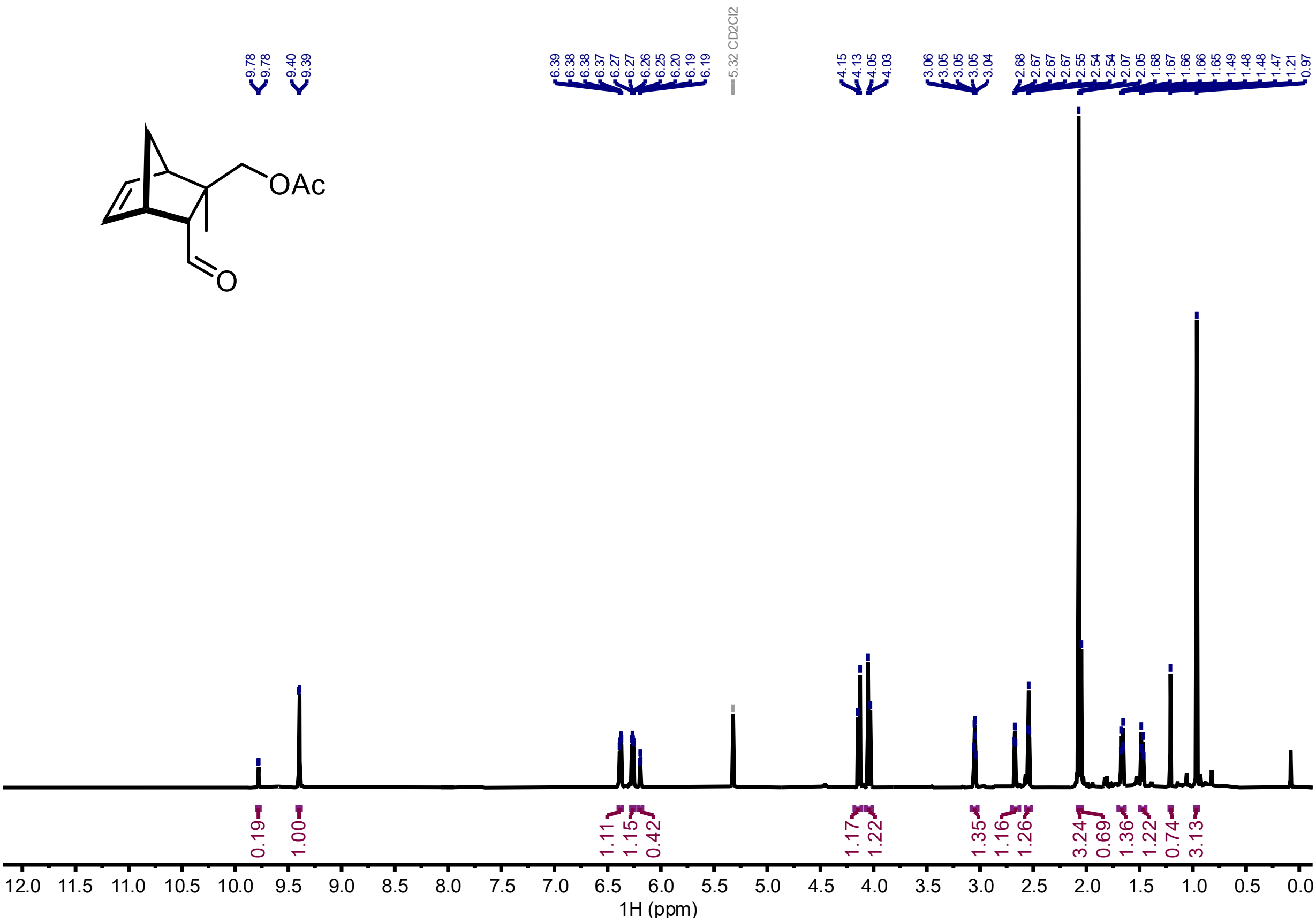

^{13}C NMR spectrum of 4j

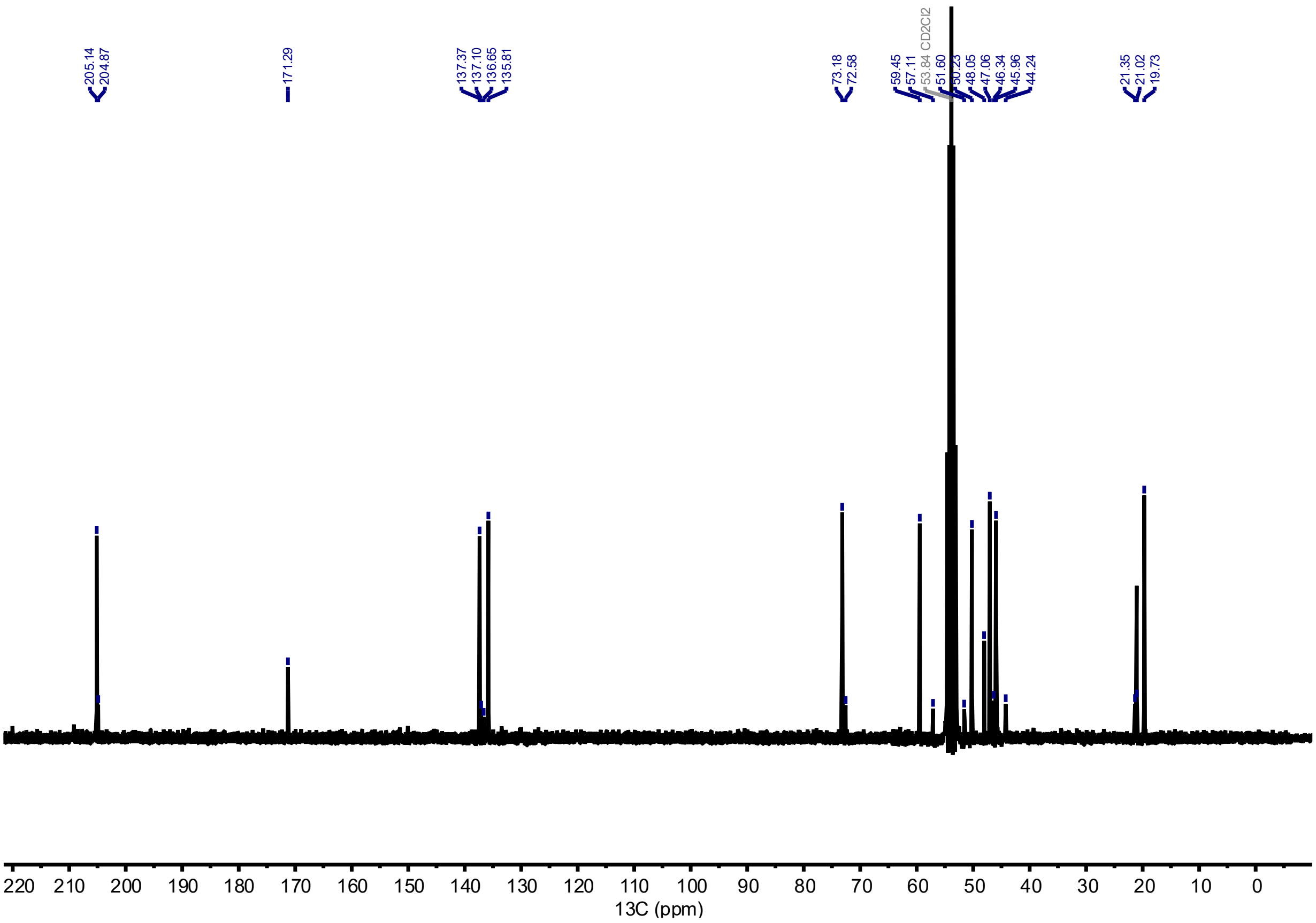

^{1}H NMR spectrum of 4k

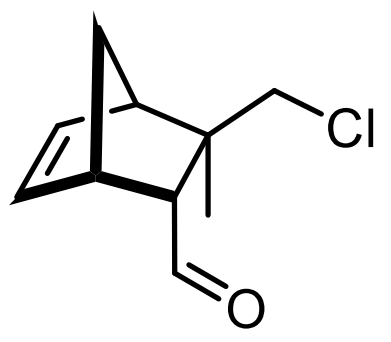

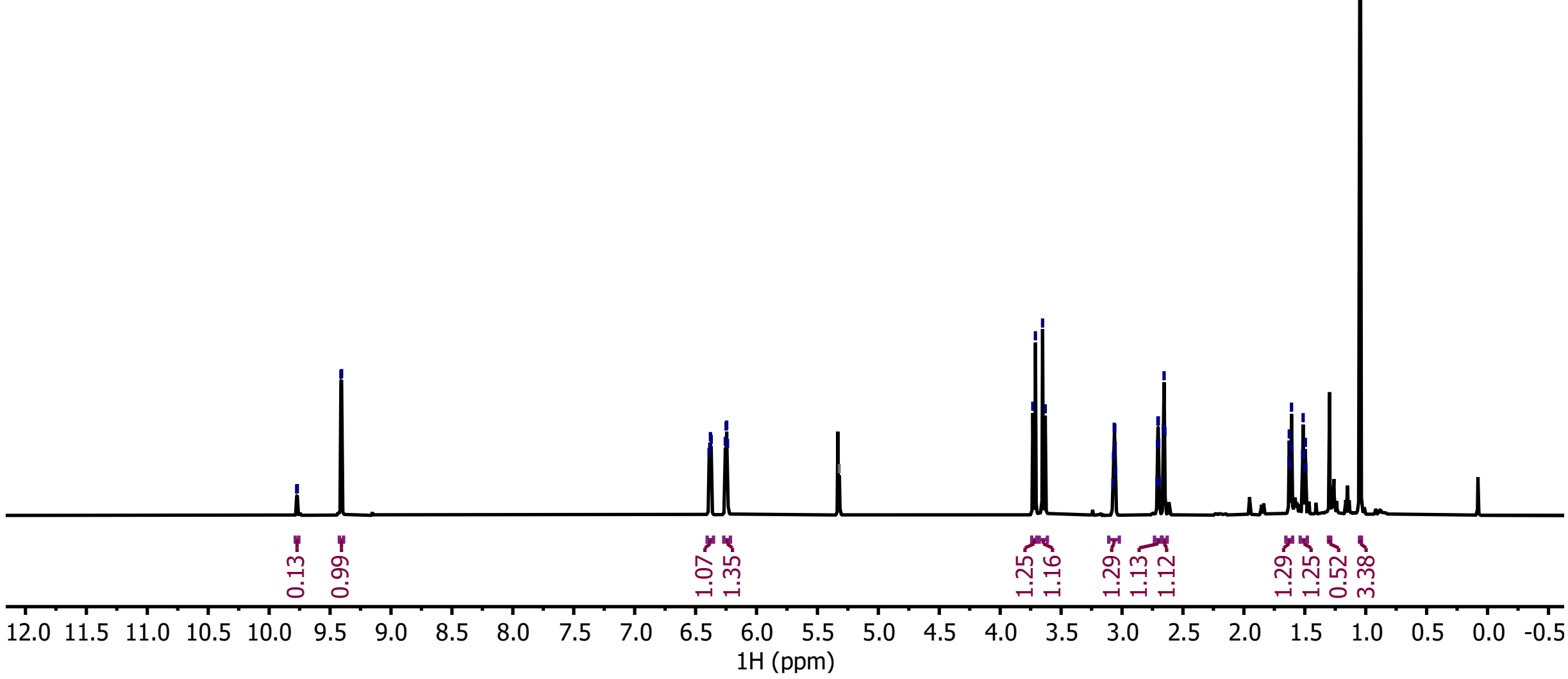

^{13}C NMR spectrum of 4k

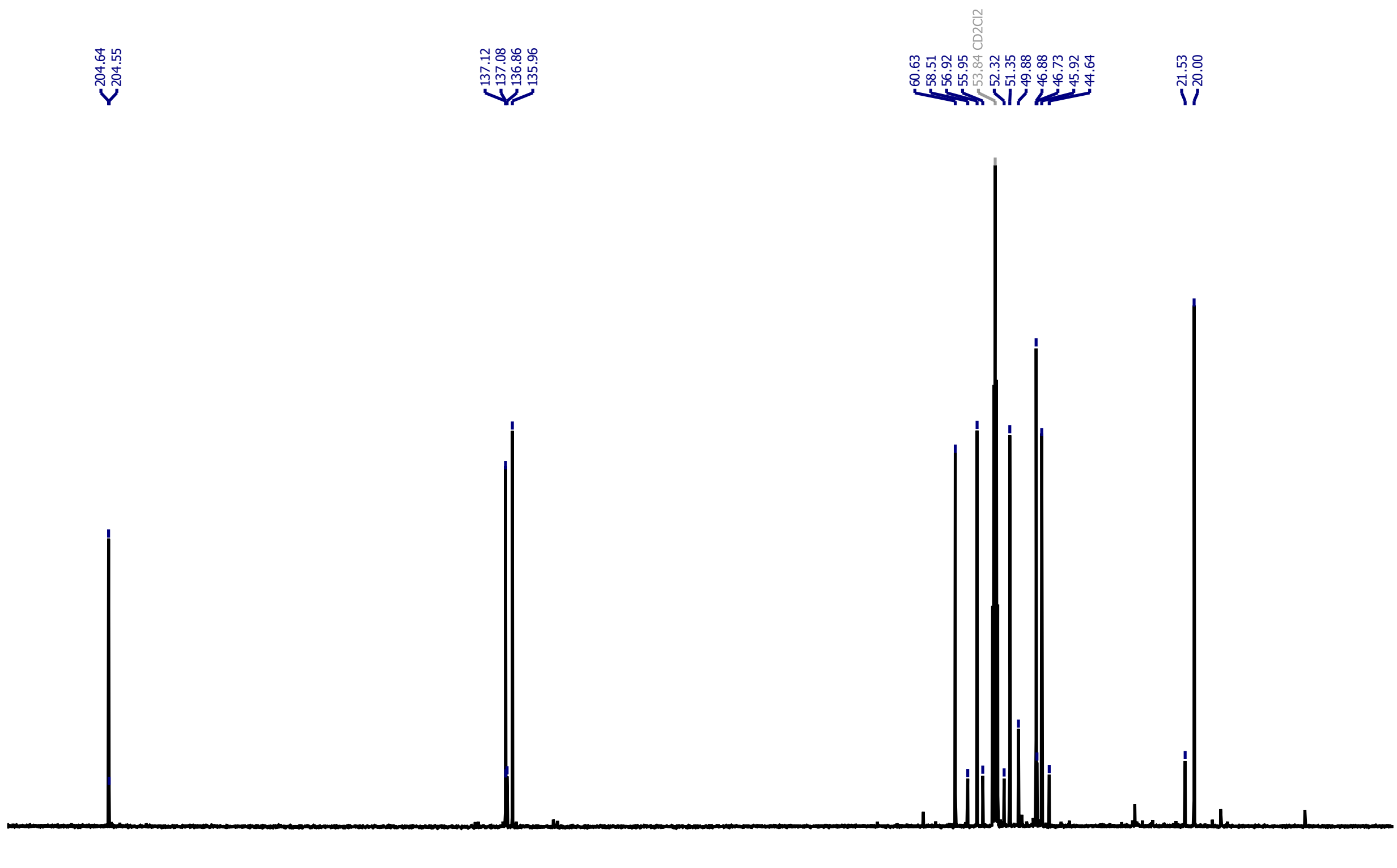

9.3. NMR Spectra of the Total Syntheses Intermediates

^{1}H NMR spectrum of 4a-R

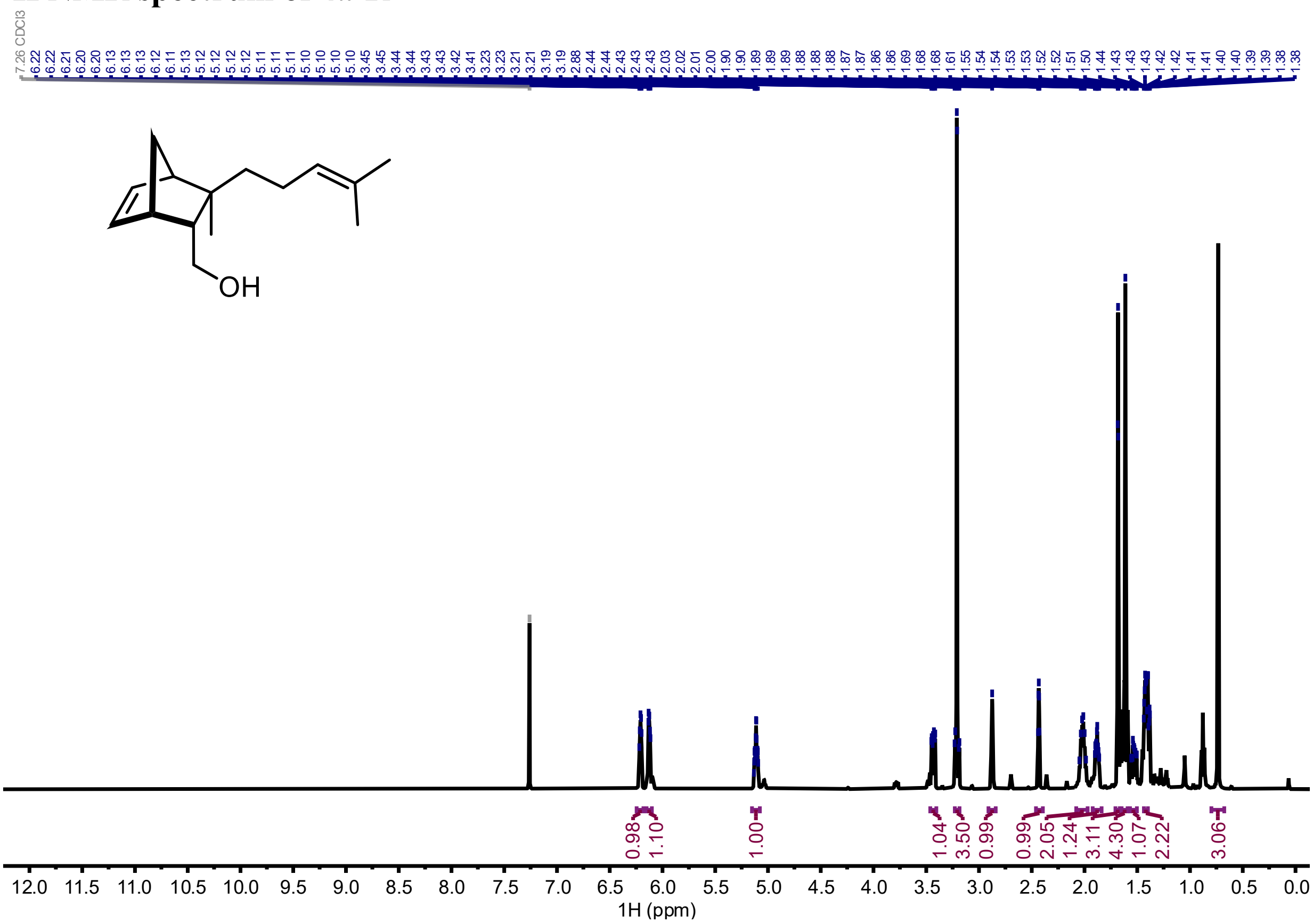

^{13}C NMR spectrum of 4a-R

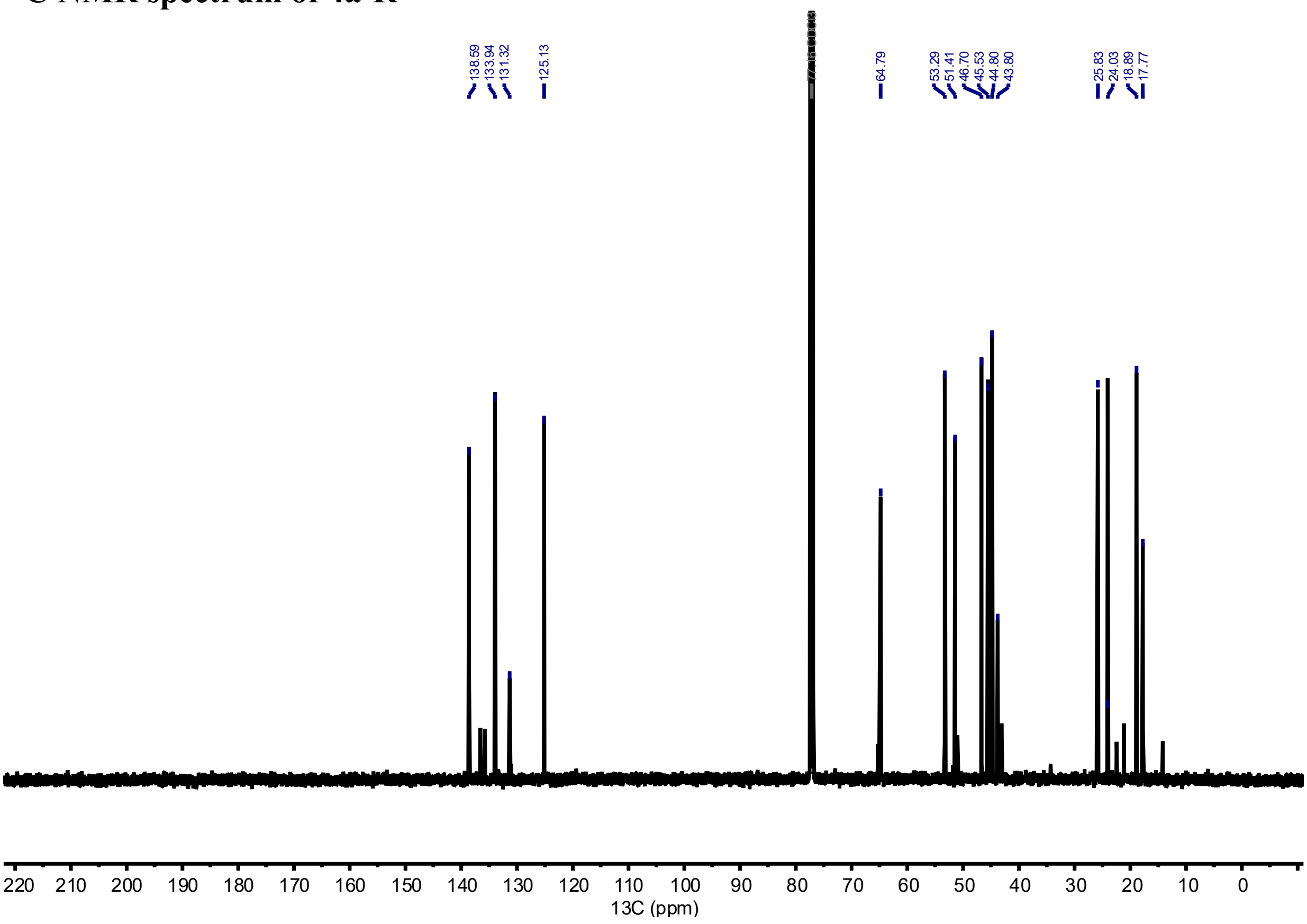

^{1}H NMR spectrum of 5

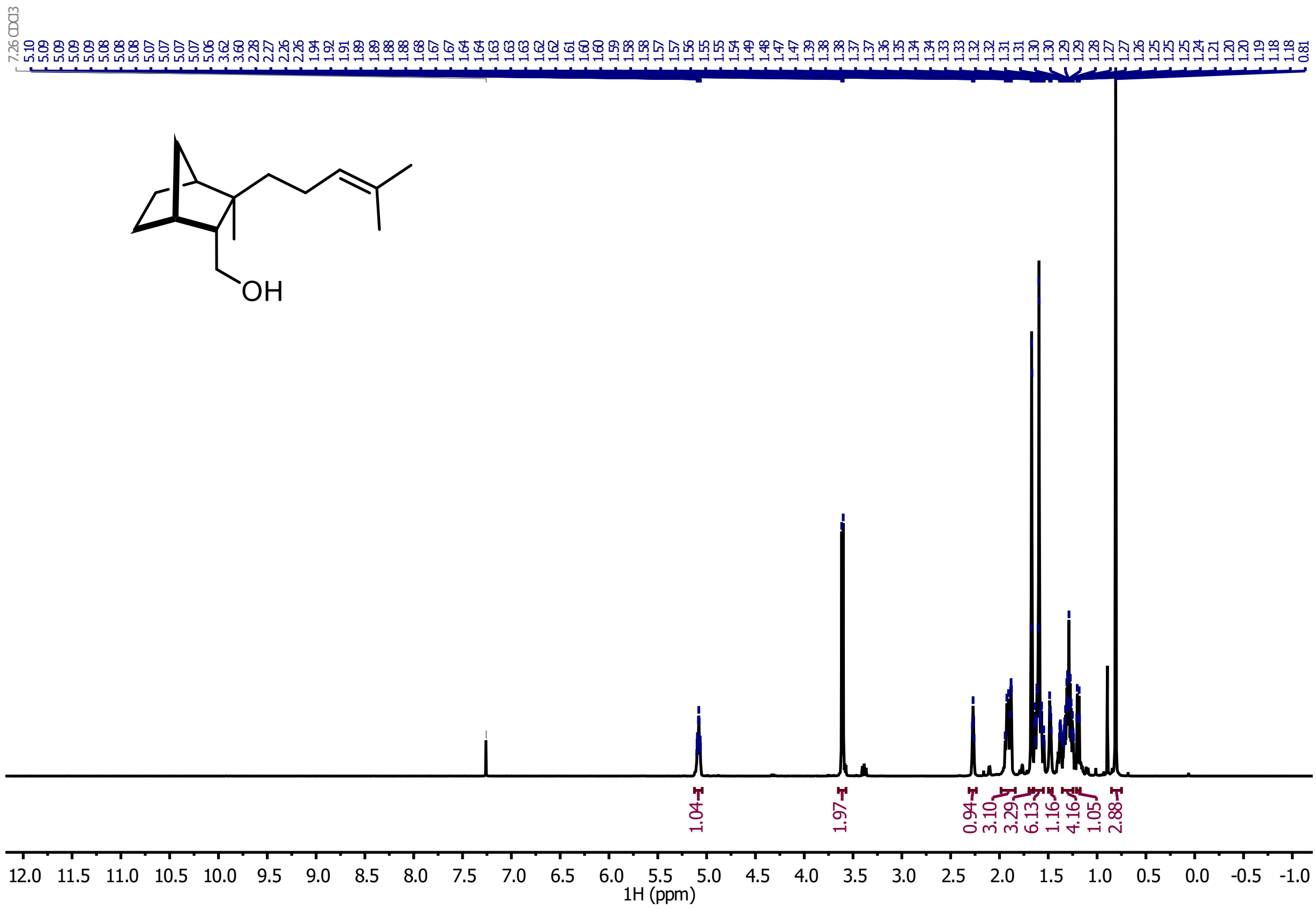

^{13}C NMR spectrum of 5

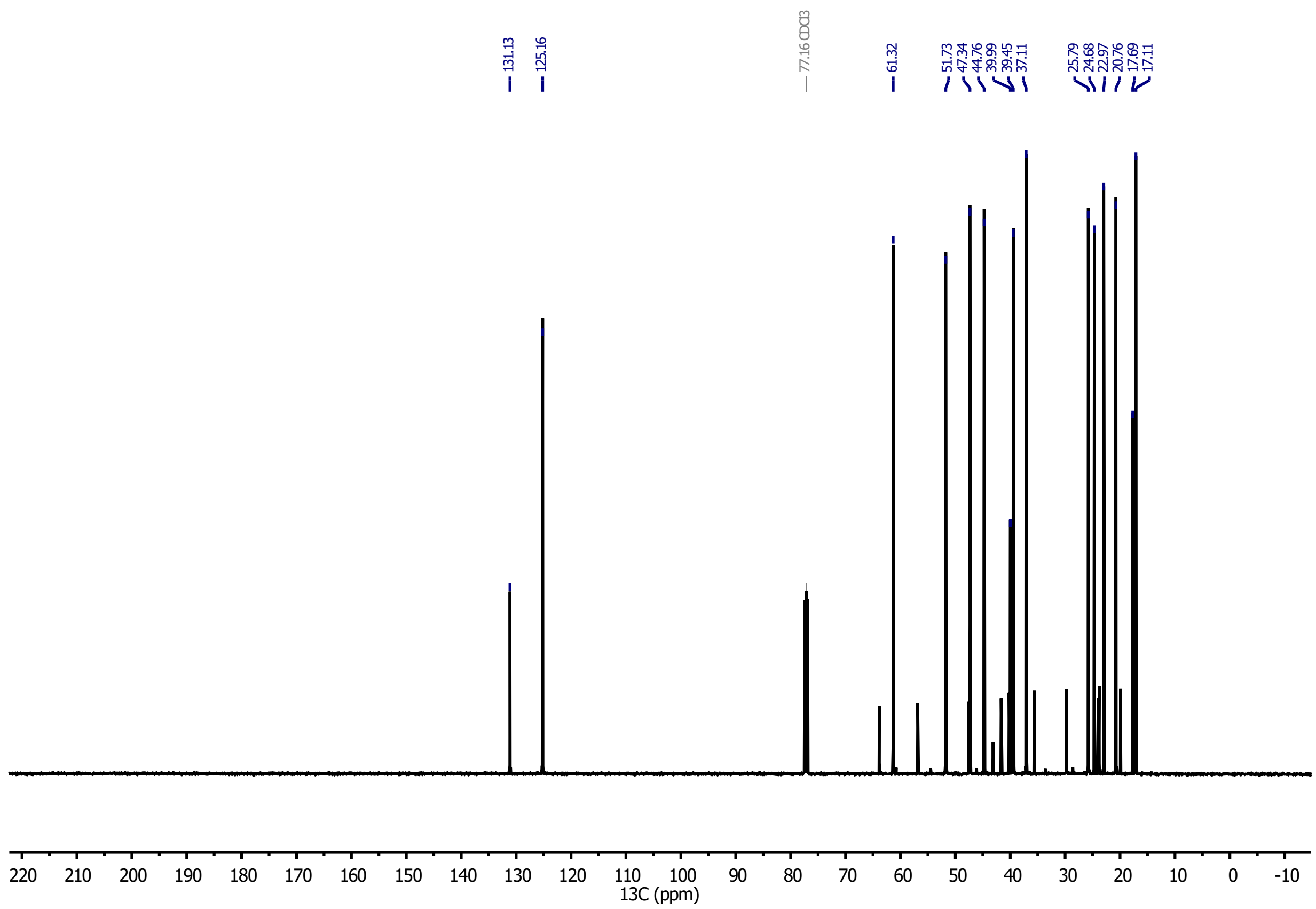

^{1}H NMR spectrum of 8

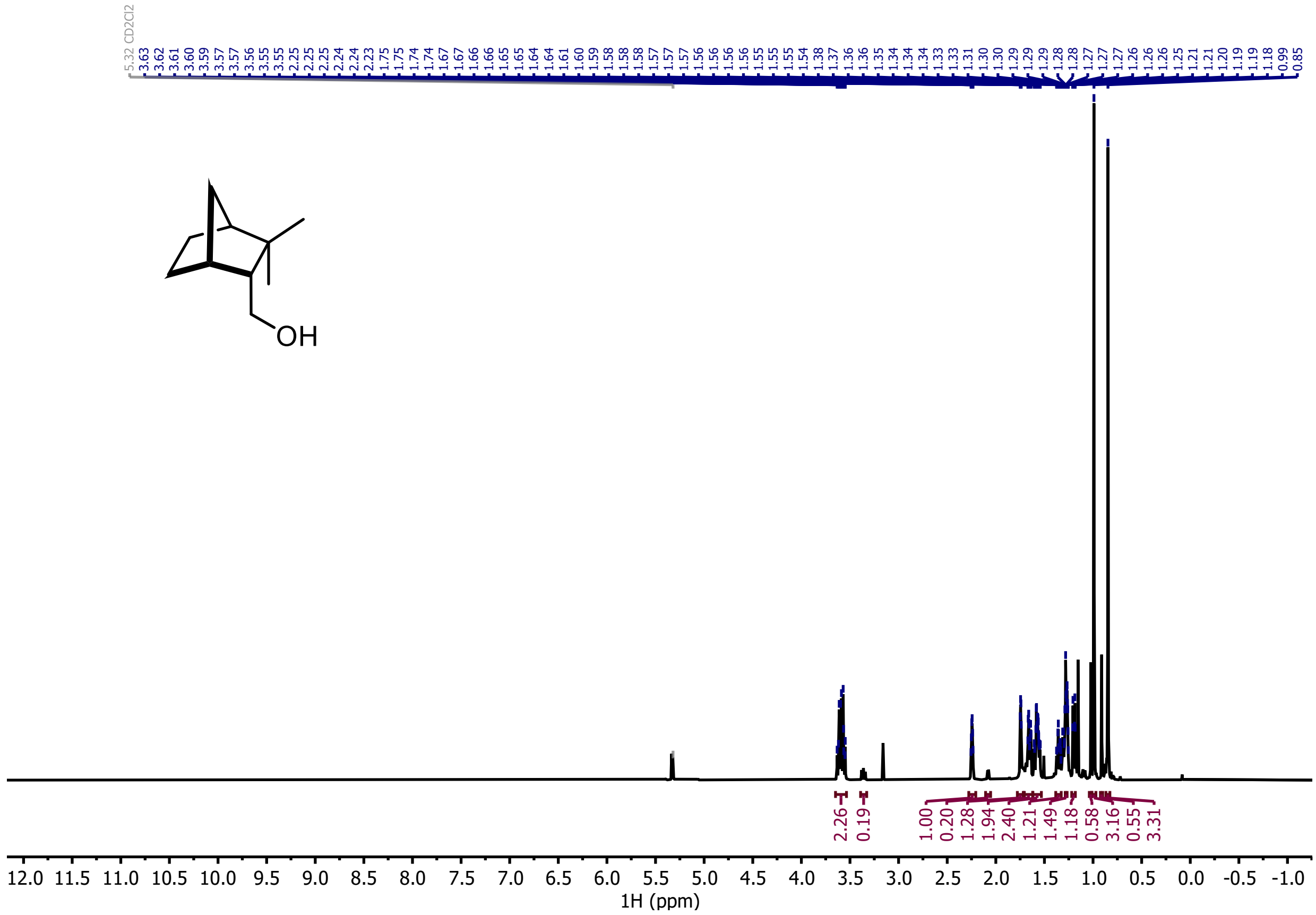

^{13}C NMR spectrum of 8

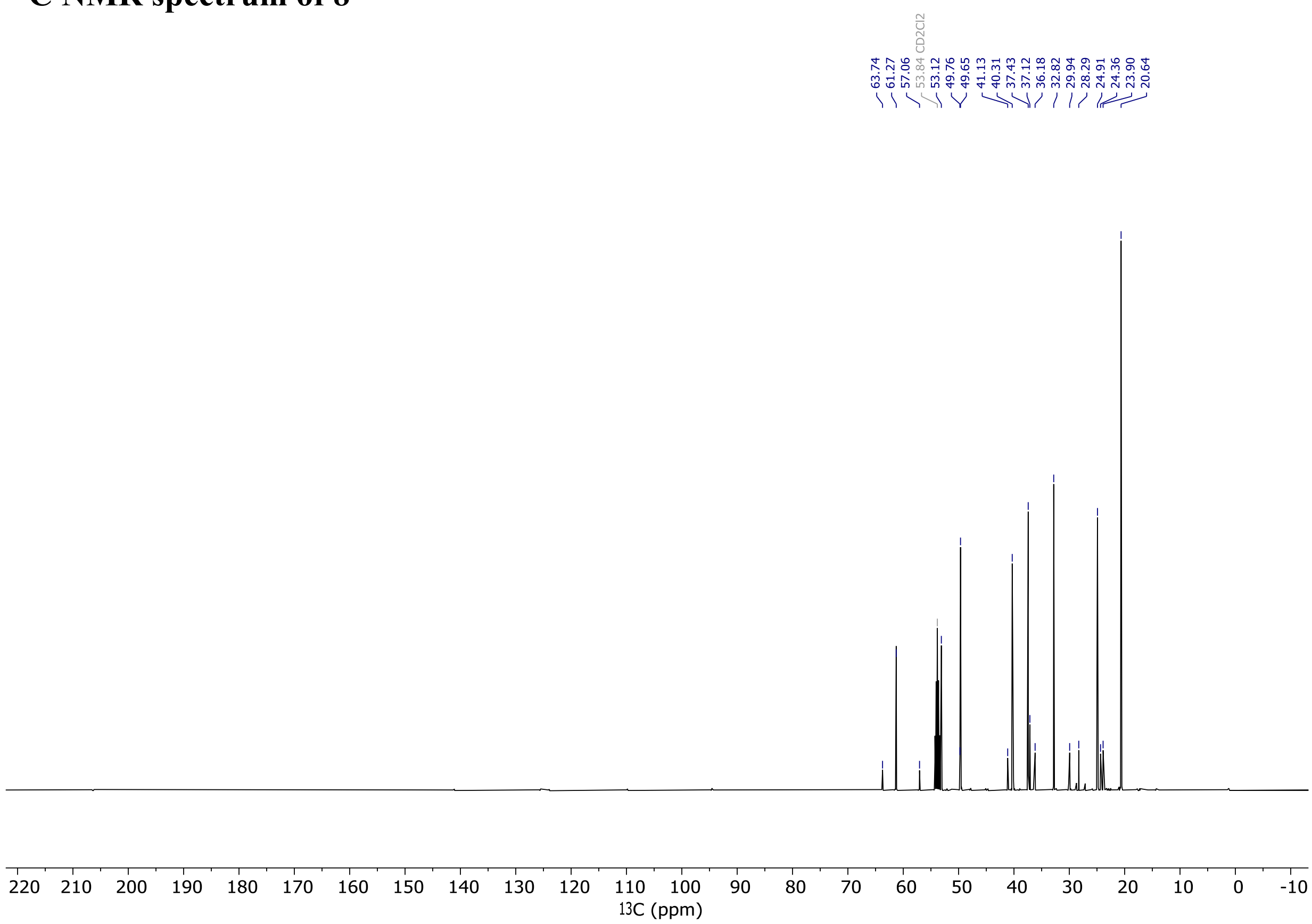

^{1}H NMR spectrum of 5-C

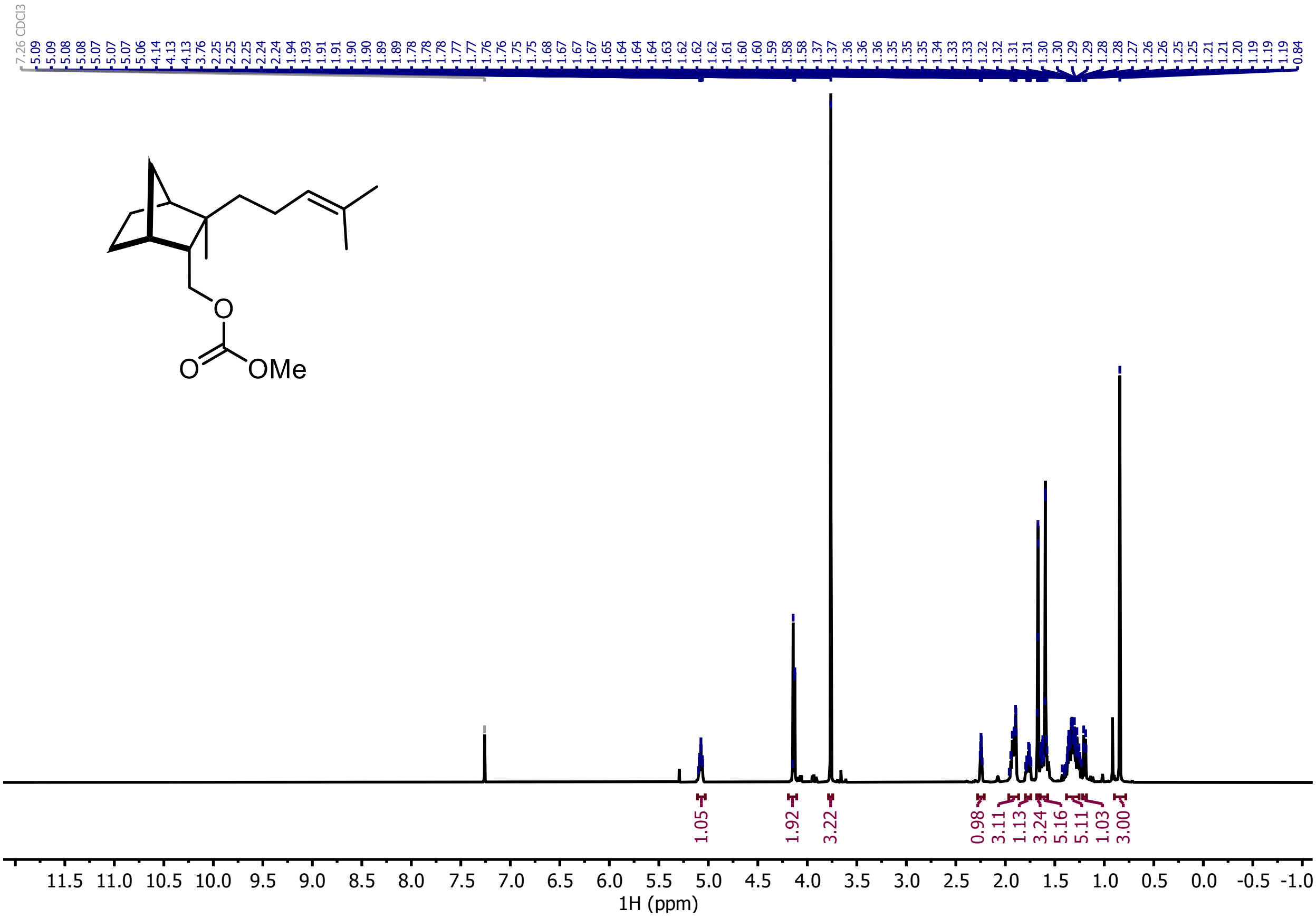

^{13}C NMR spectrum of 5-C

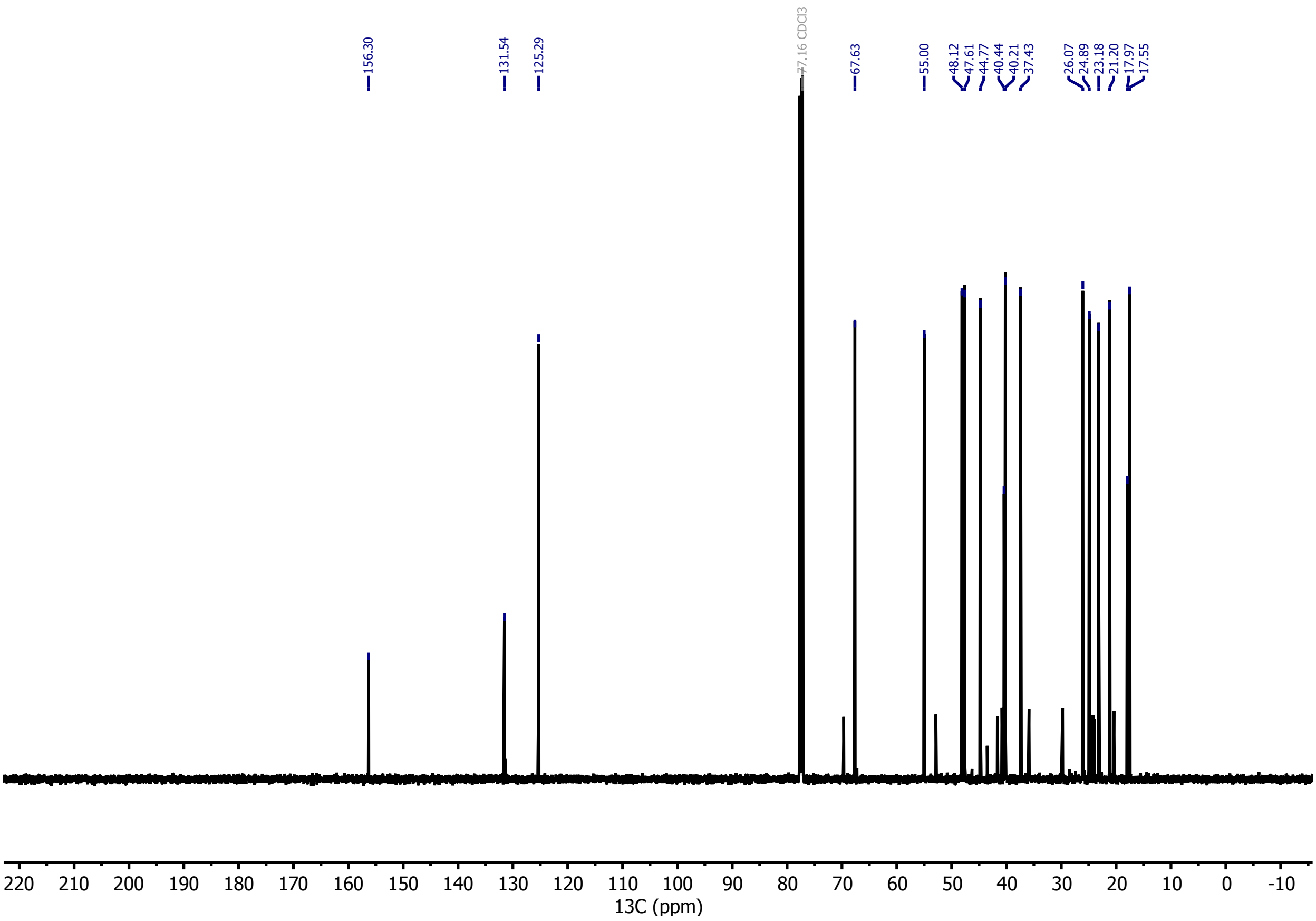

^{1}H NMR spectrum of 6

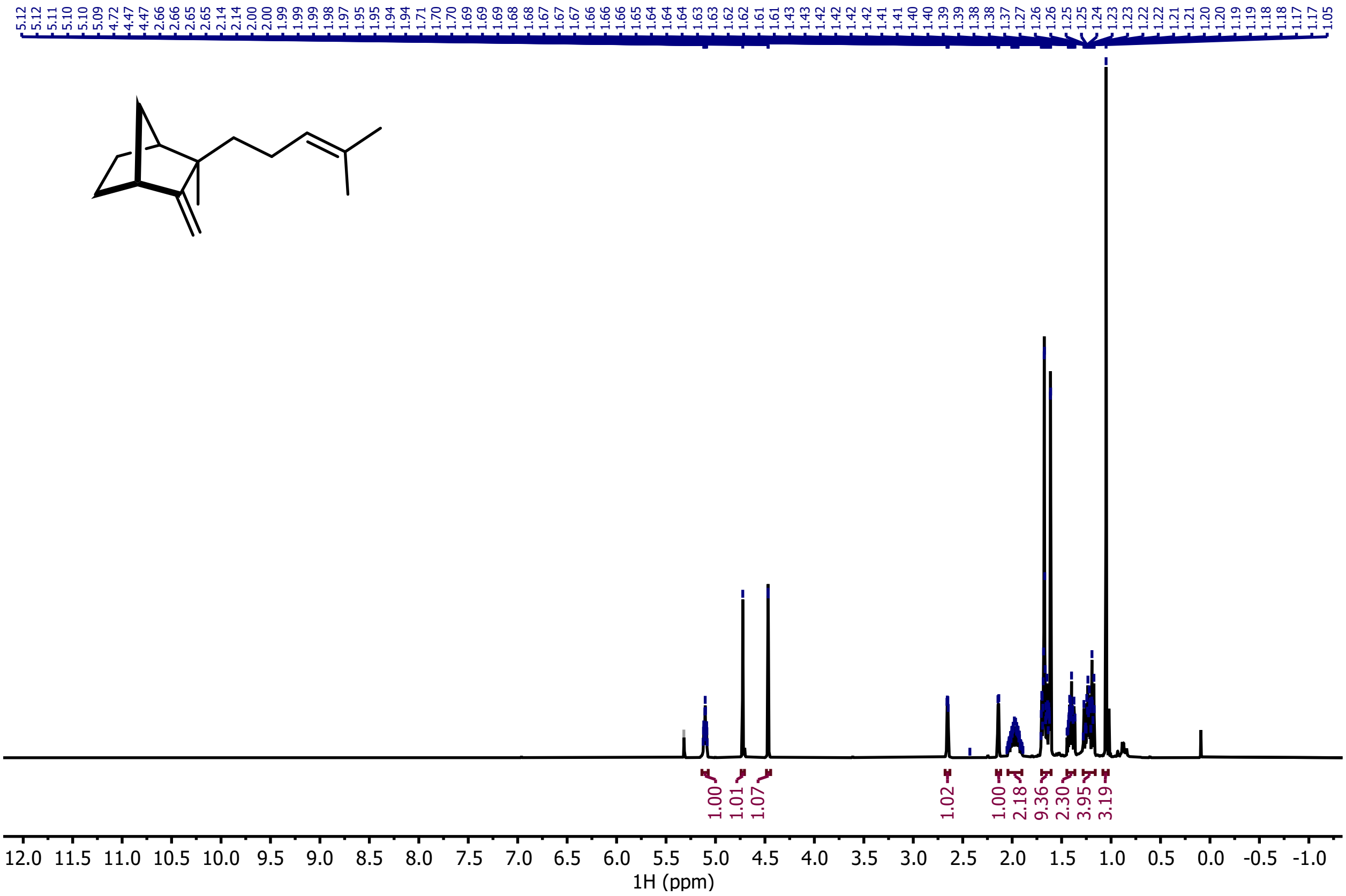

^{13}C NMR spectrum of 6

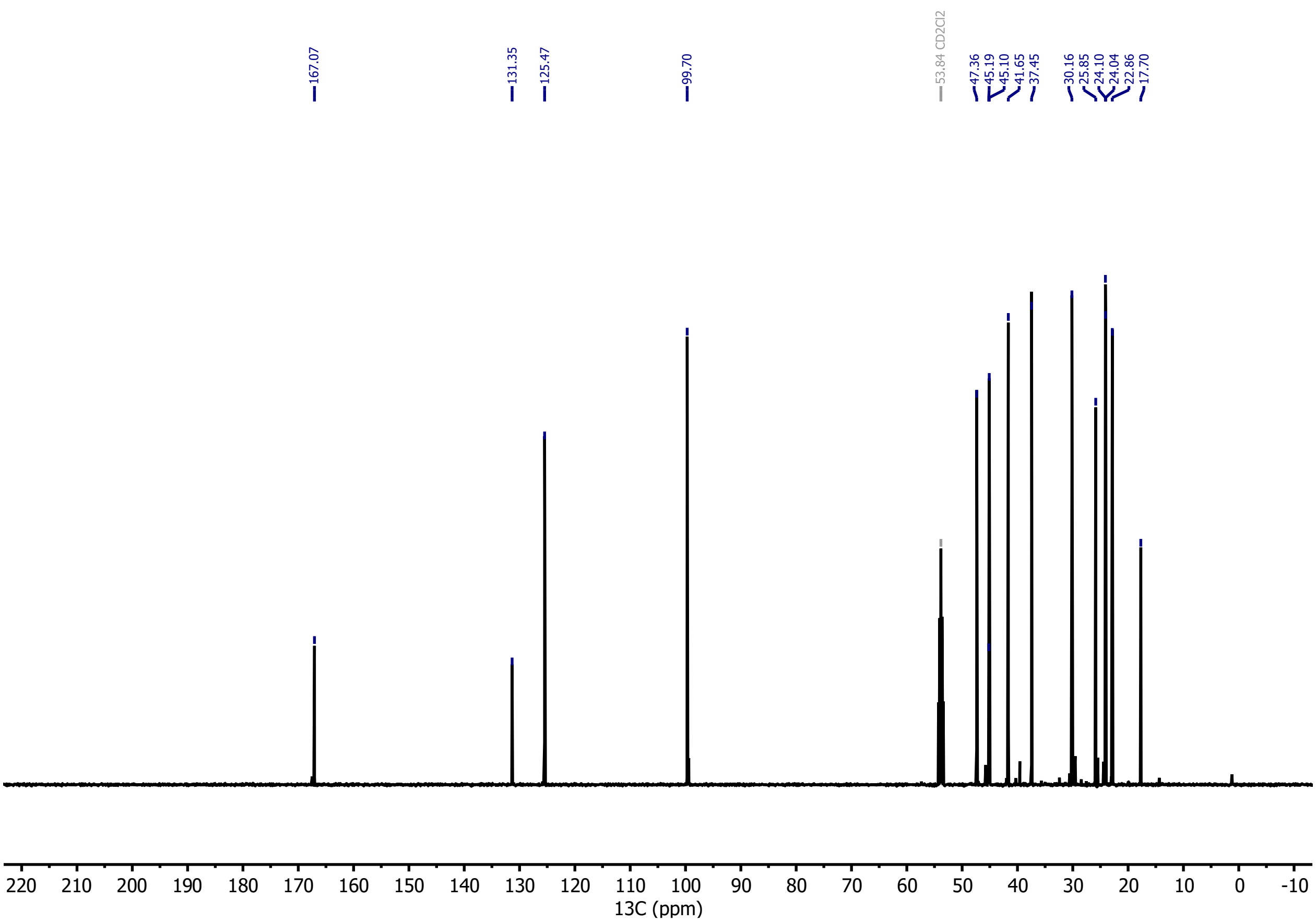

^{1}H NMR spectrum of 9

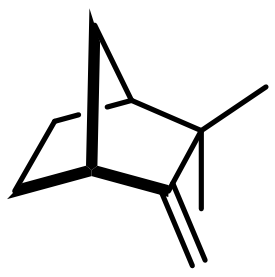

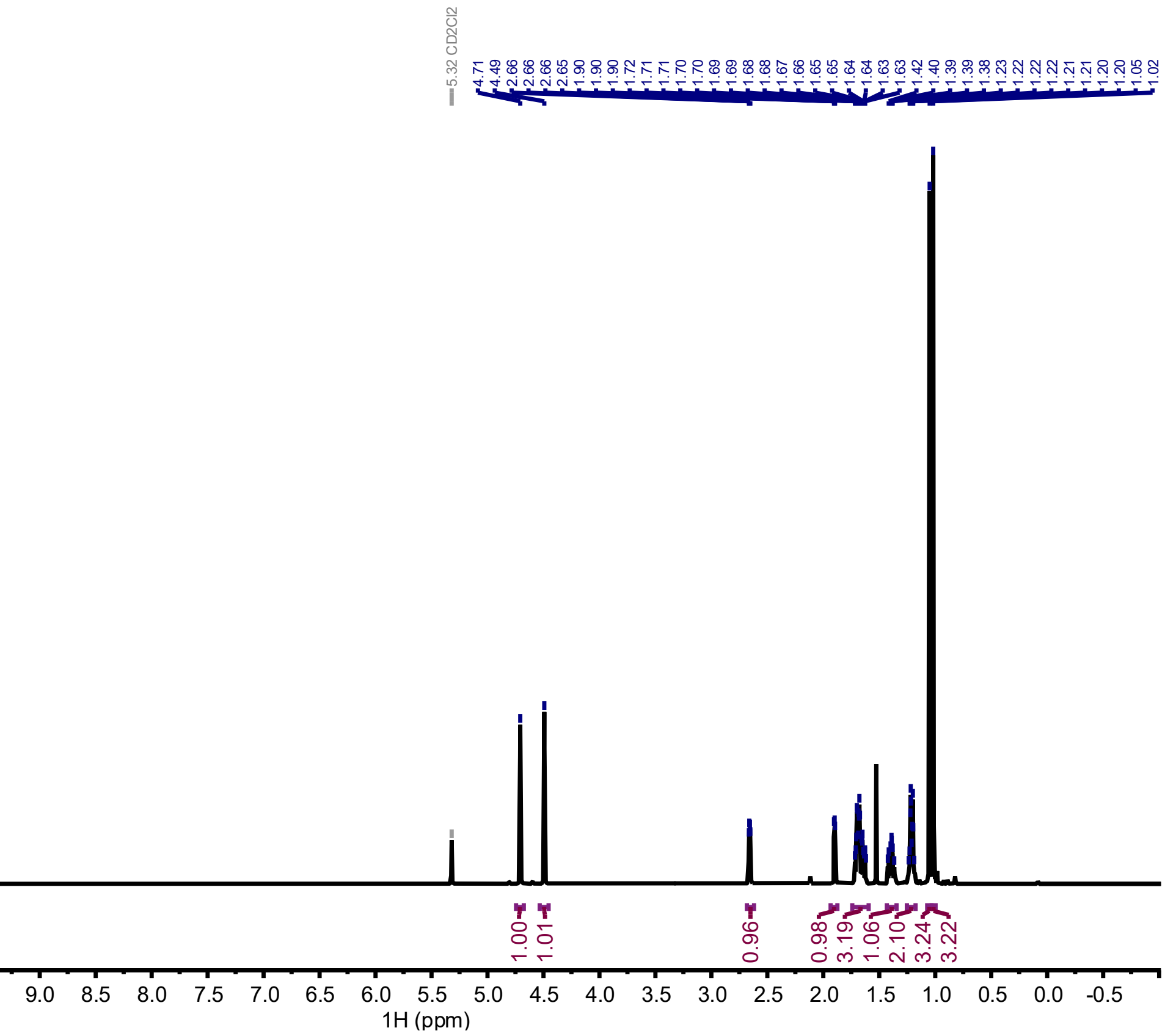

^{1}H NMR spectrum of 7

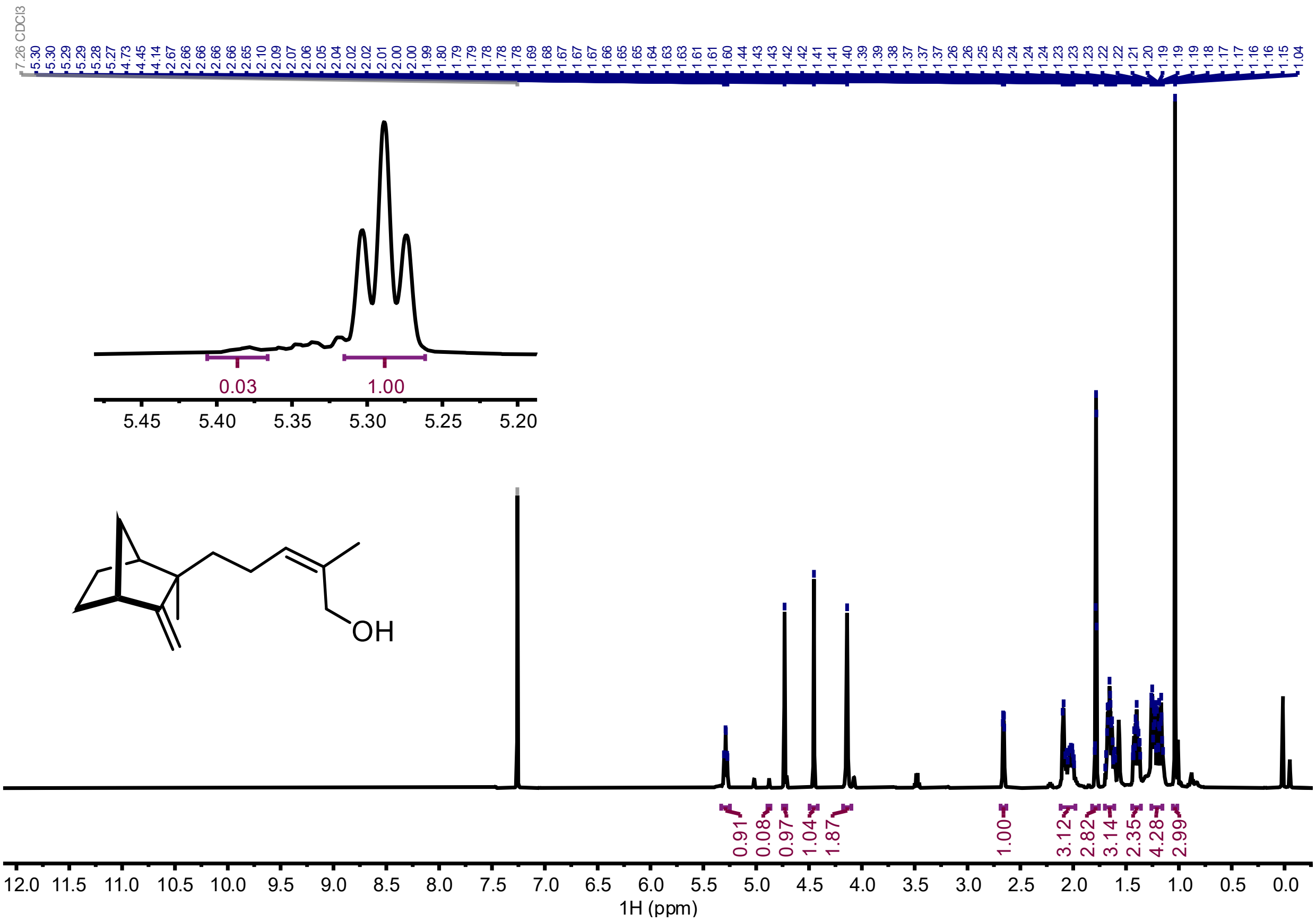

^{13}C NMR spectrum of 7

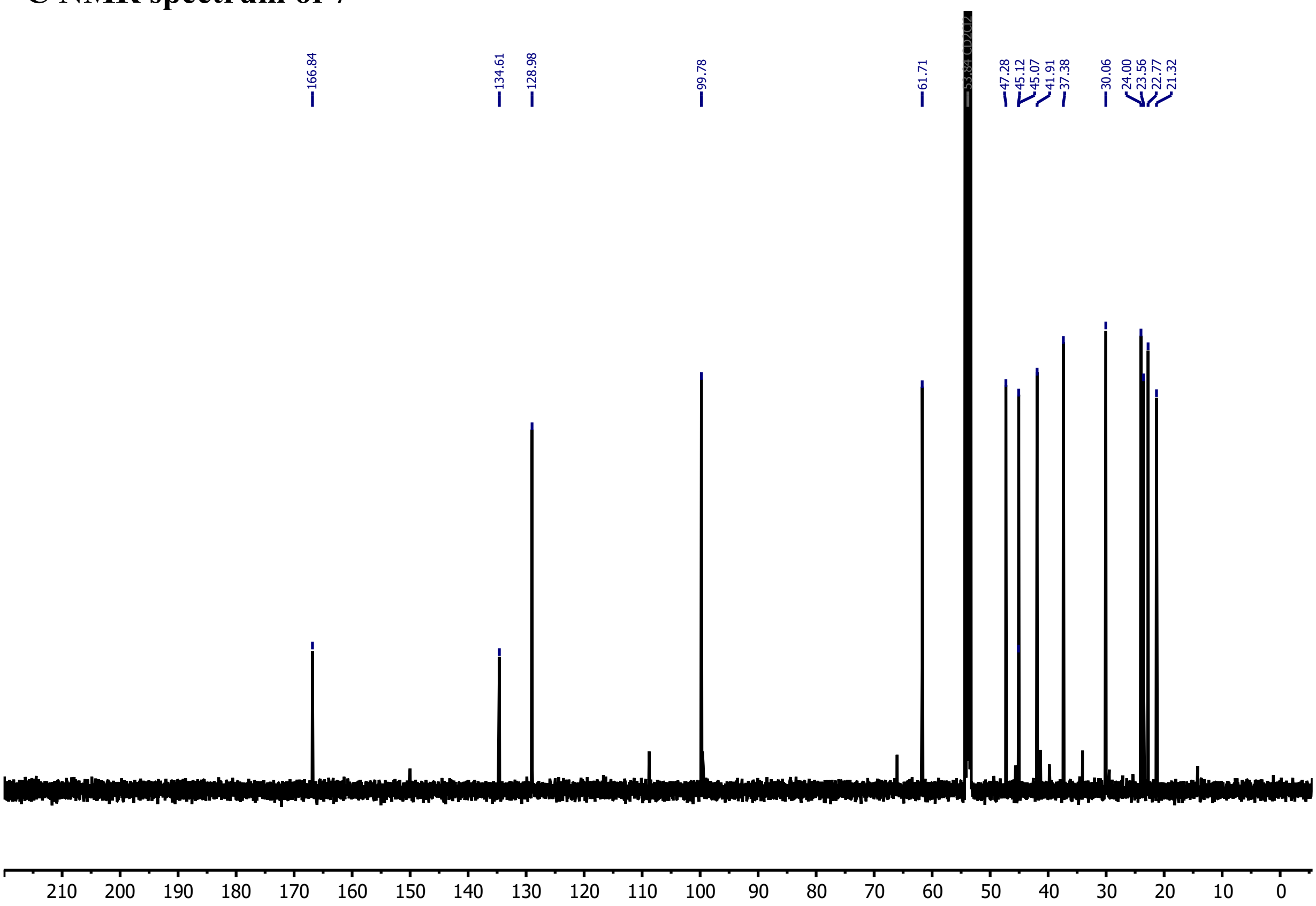

^{1}H NMR spectrum of 7-(*E*)

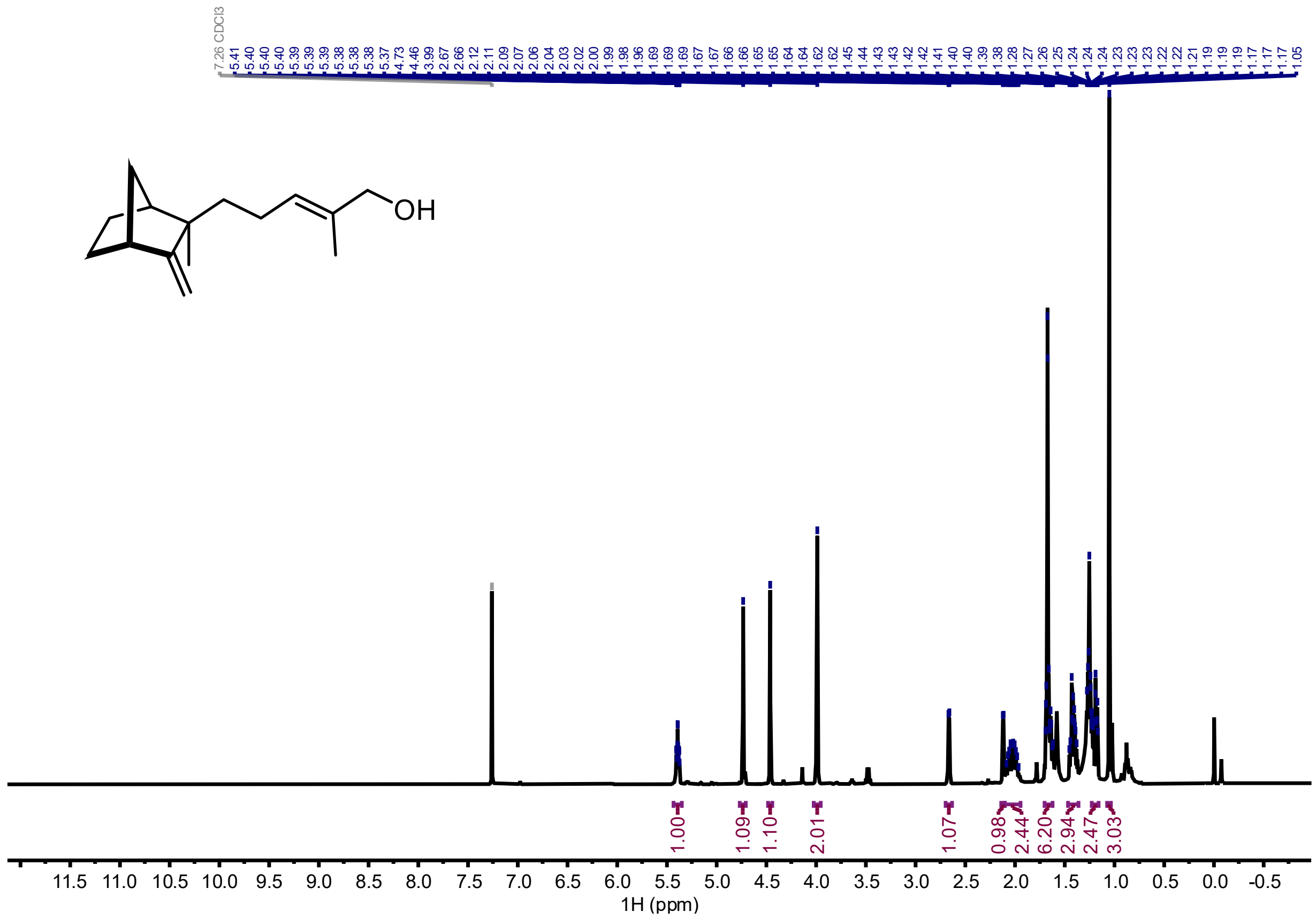

^{13}C NMR spectrum of 7-(*E*)

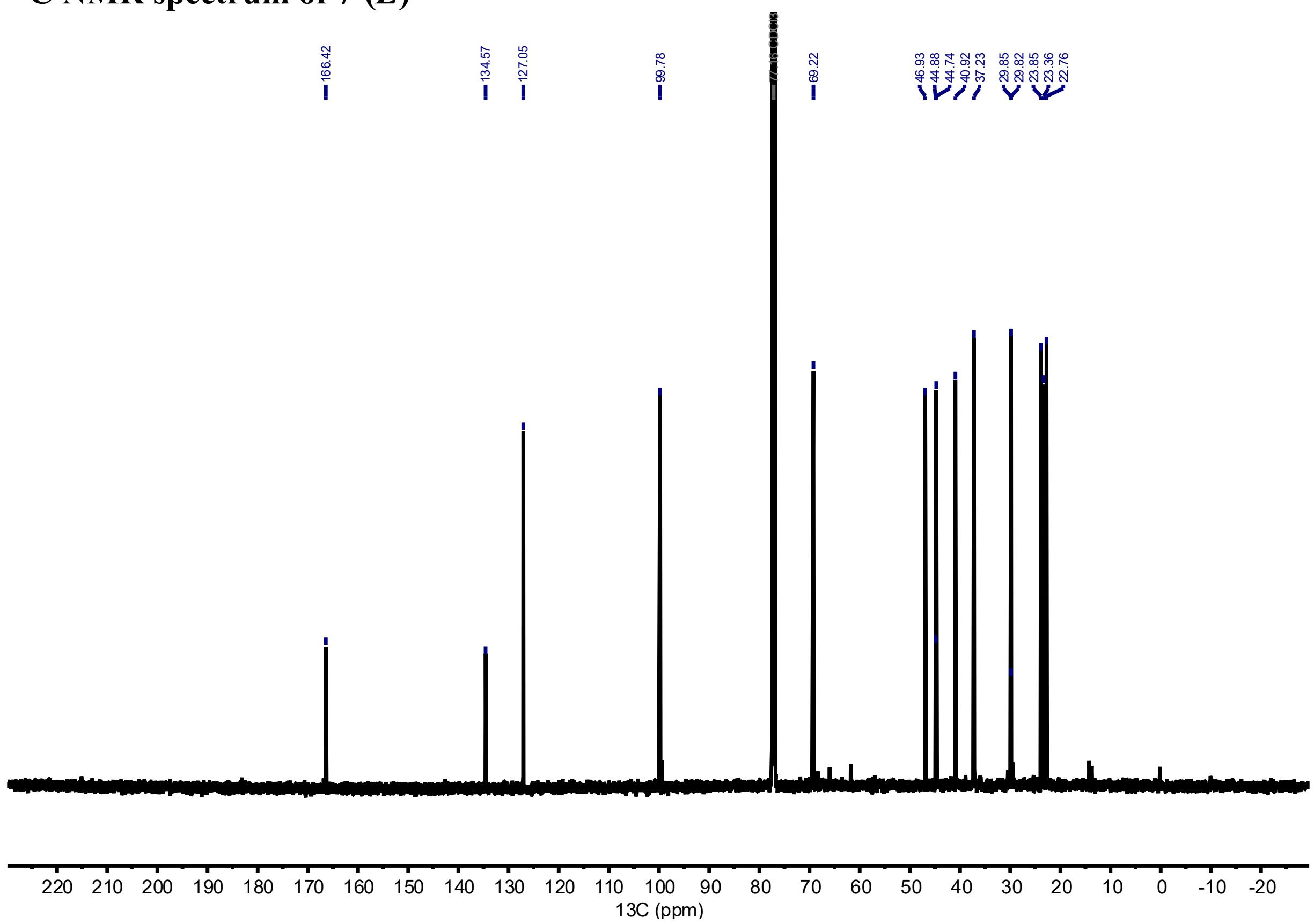

Comparison of ^{1}H NMR spectra of 7-(*Z*) and 7-(*E*)

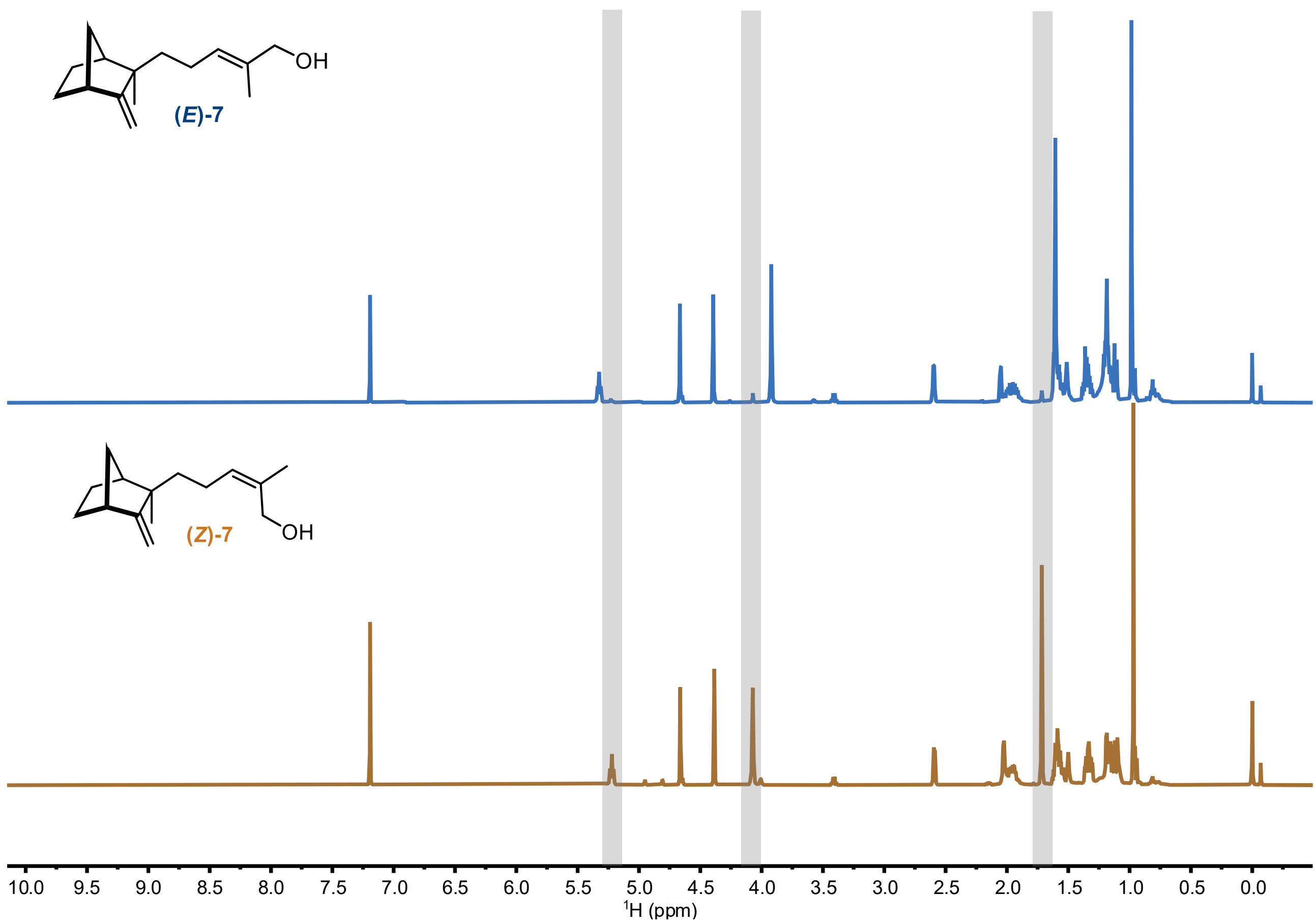

9.4. NMR Spectra of Catalysts

^{1}H NMR spectrum of 3f

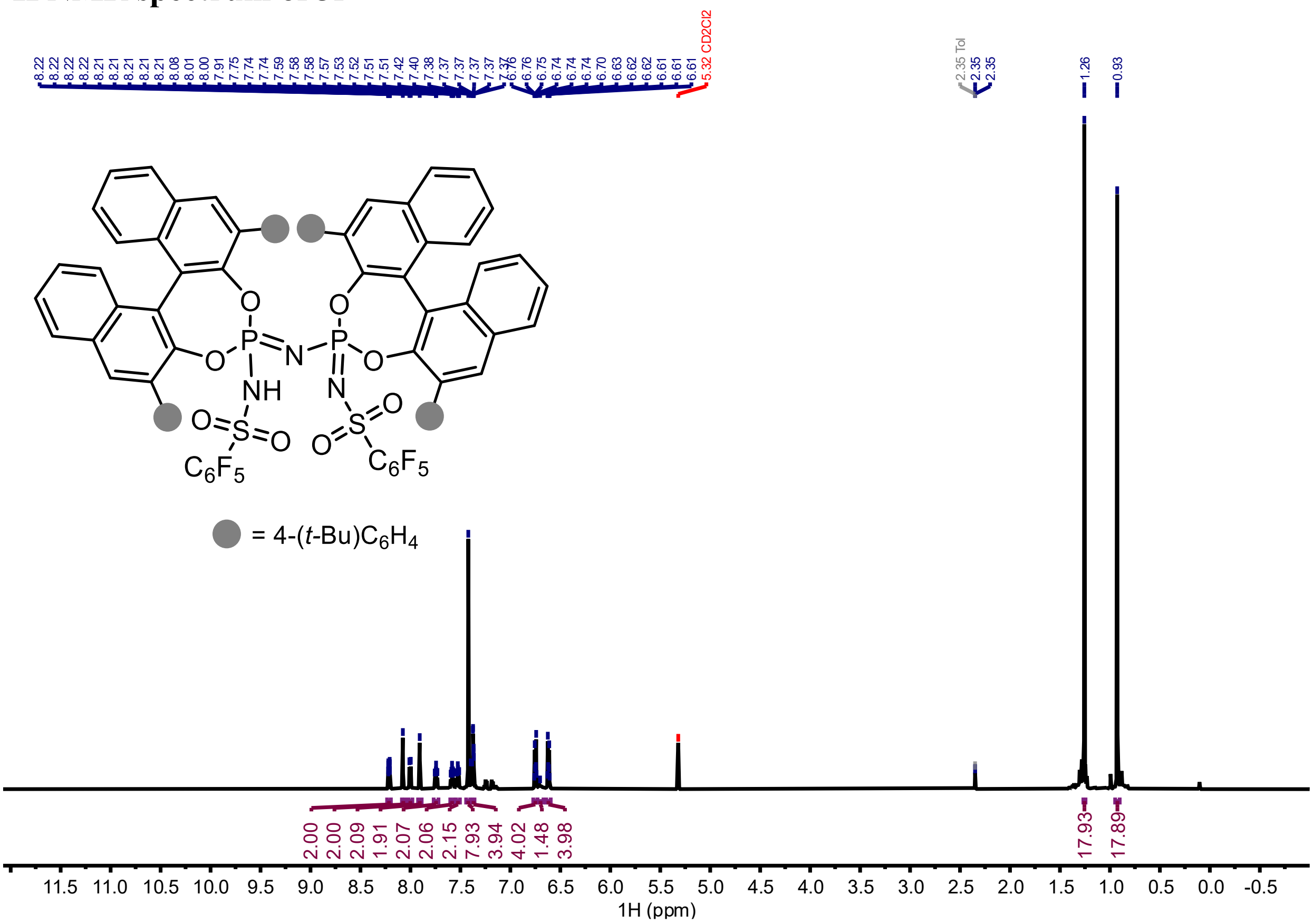

^{13}C NMR spectrum of 3f

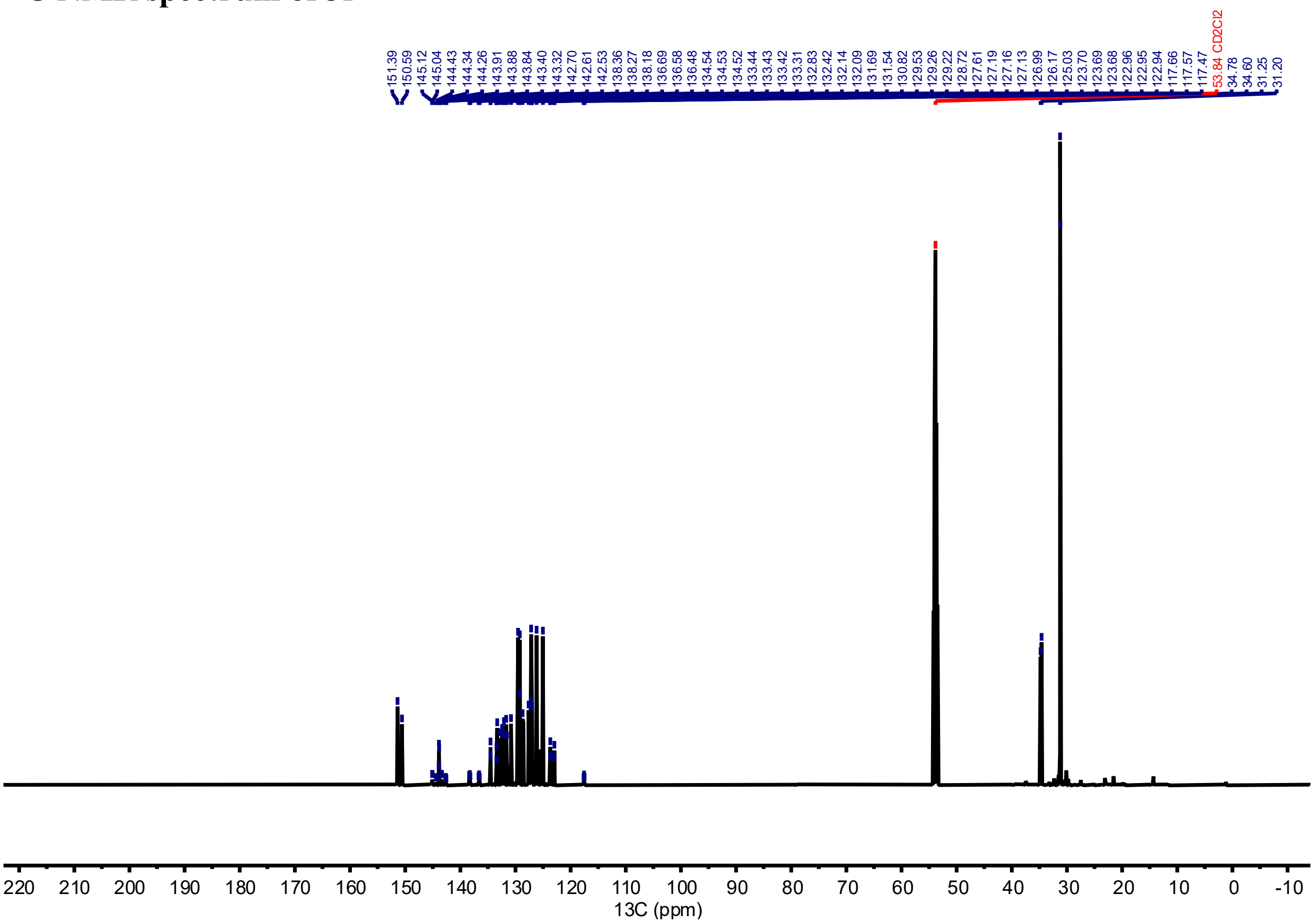

^{19}F NMR spectrum of 3f

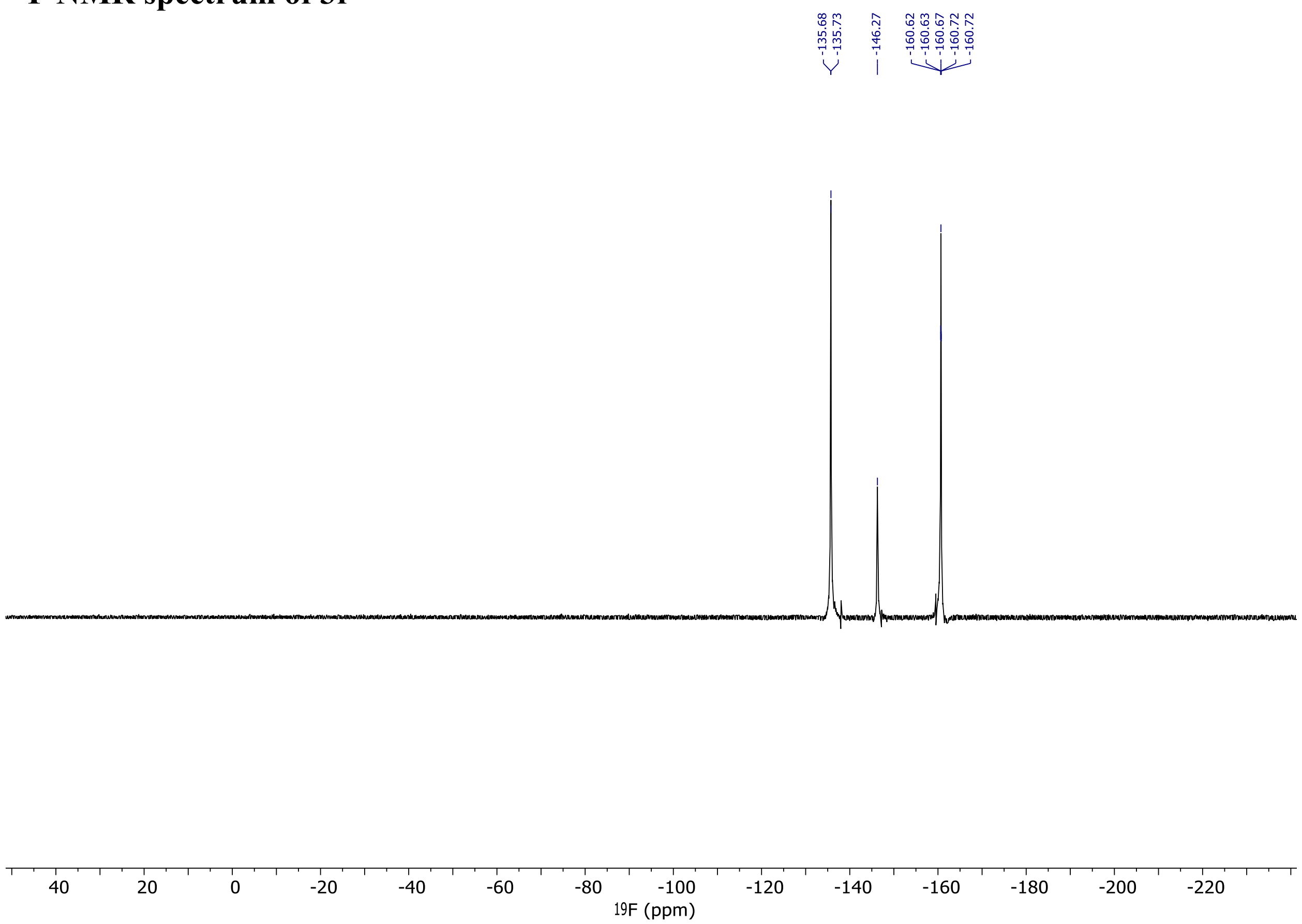

^{31}P NMR spectrum of 3f

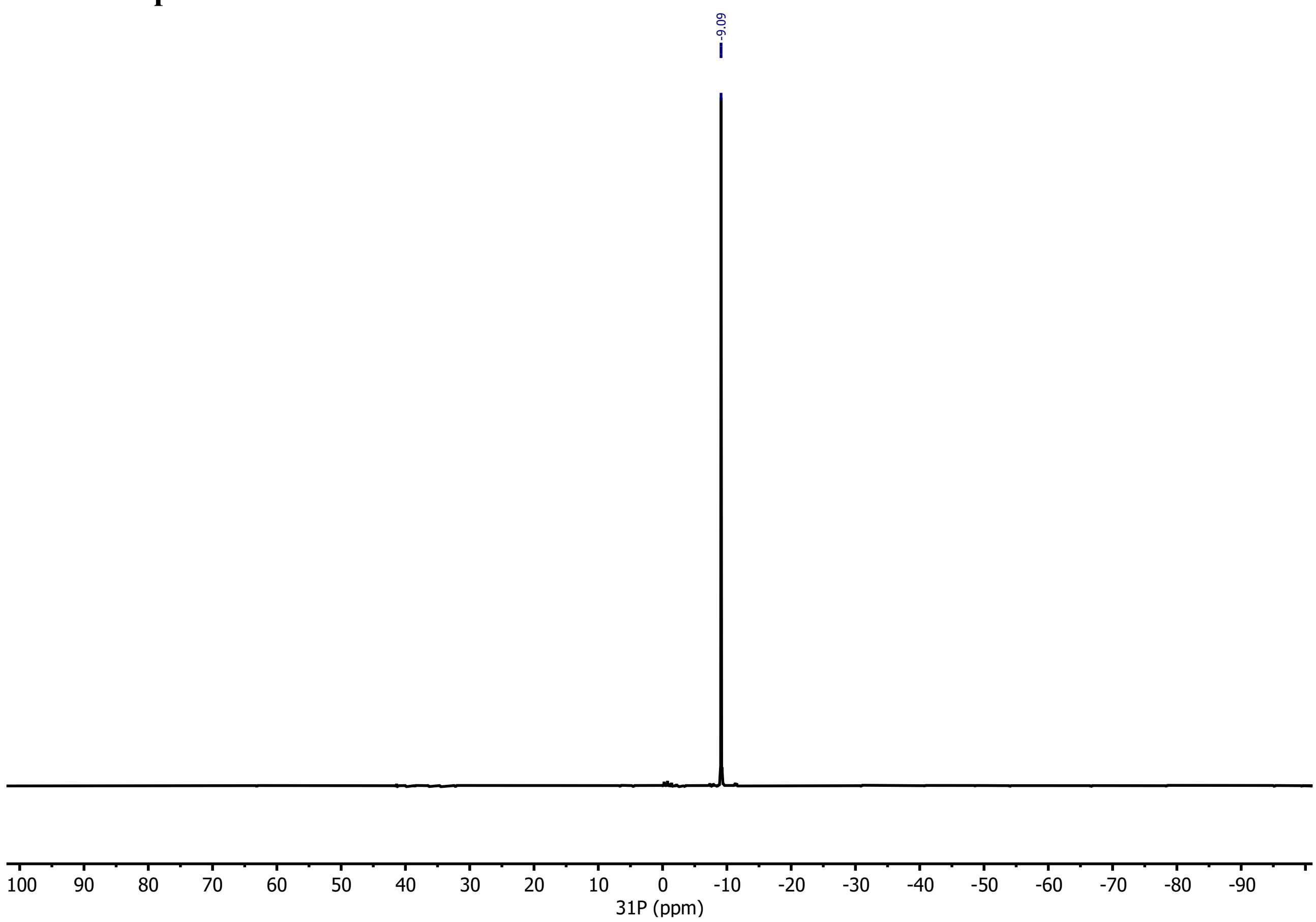

^{1}H NMR spectrum of oxazaborolidine precursor for catalyst (A)

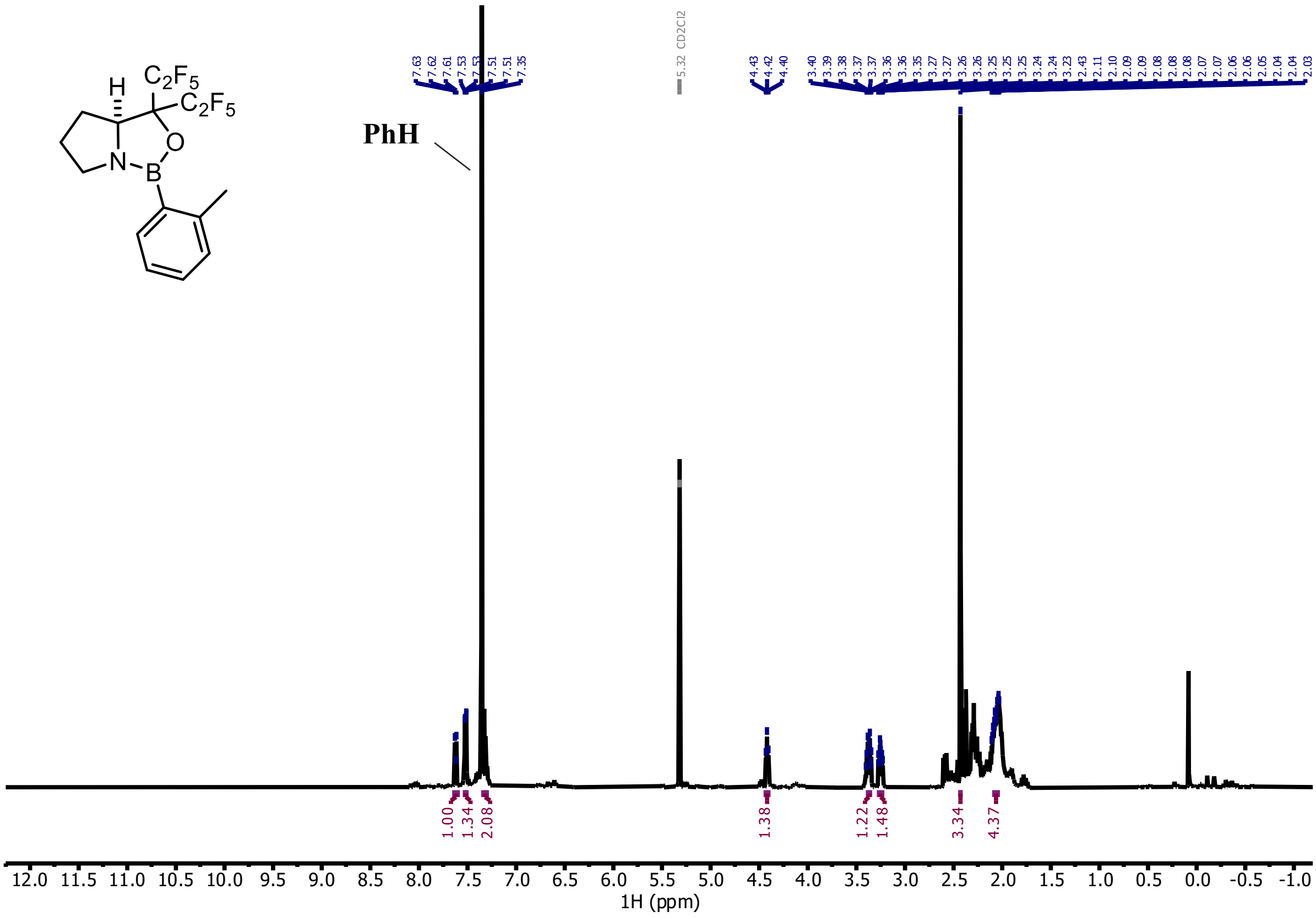

^{1}H NMR spectrum of the prolinol catalyst (B)

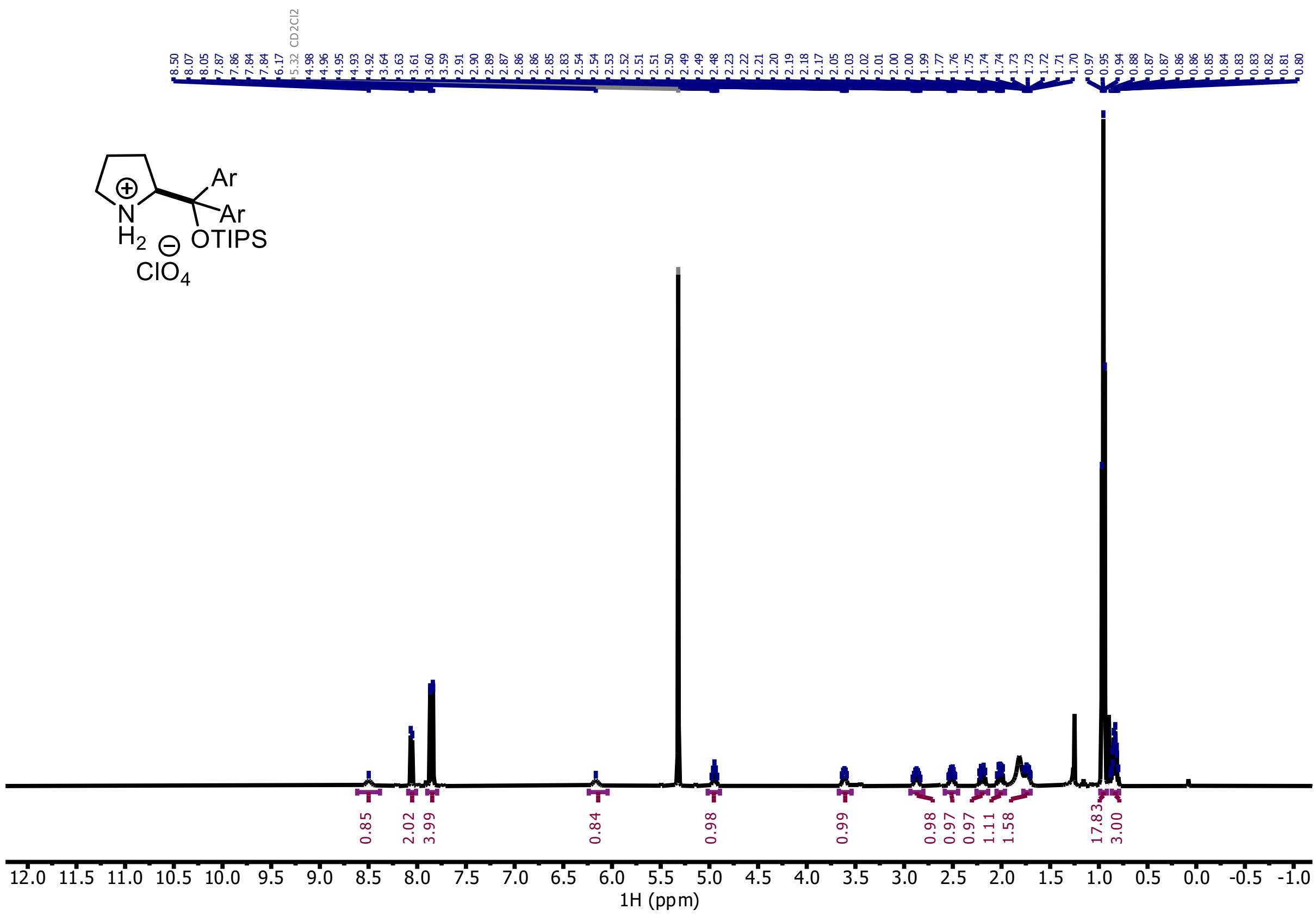

10. GC and HPLC traces

GC (29.5 m BGB-178, injection temperature: 220 °C; 100 °C – iso 5 min, 1 °C/min, 230 °C iso 10 min, 0.5 bar H_2)

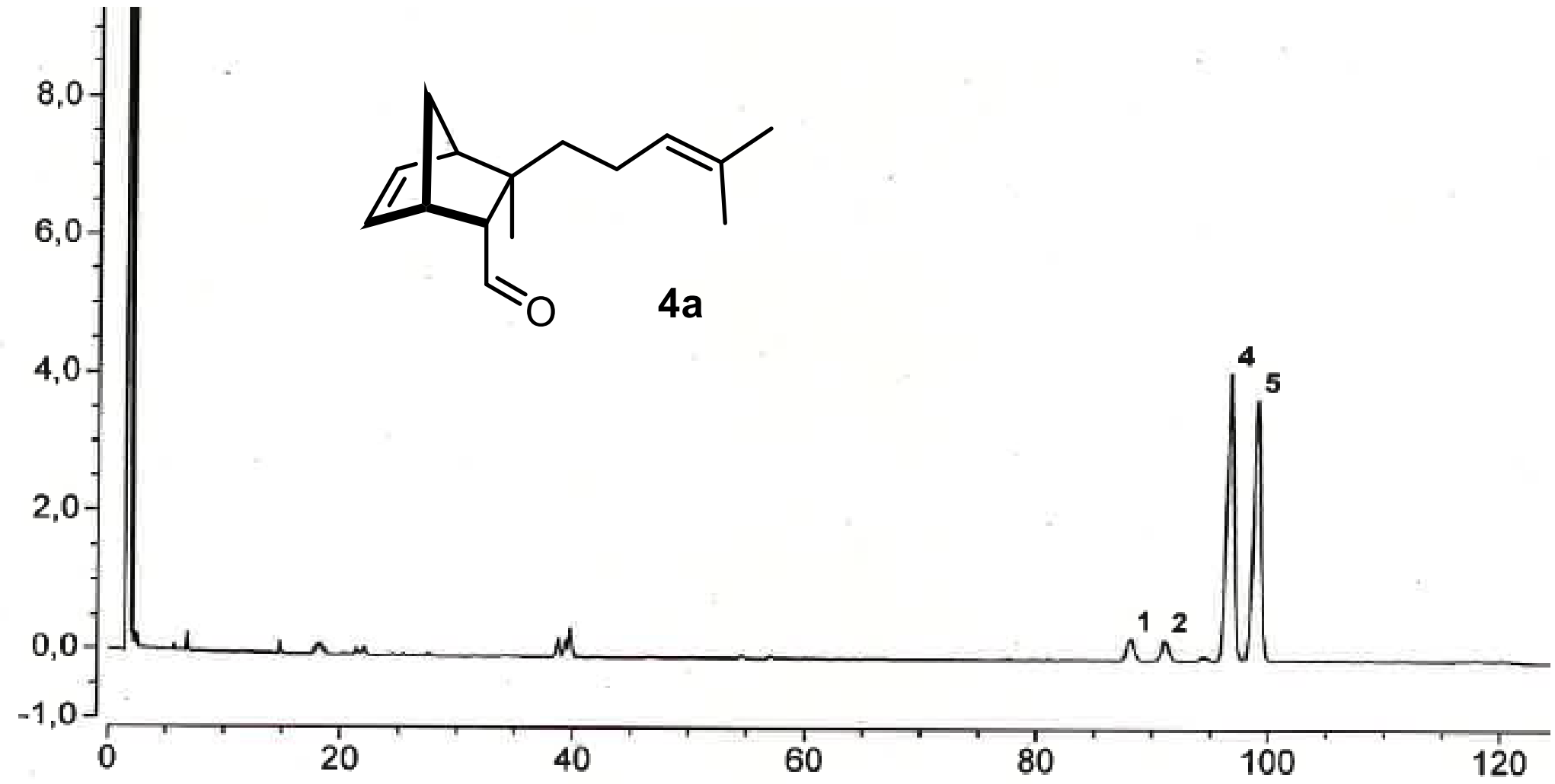

No.	Ret.Time min	Rel.Area %	Peak Name
1	88,08	3,87	*
2	91,07	3,49	*
4	96,65	48,14	**
5	98,93	44,50	**

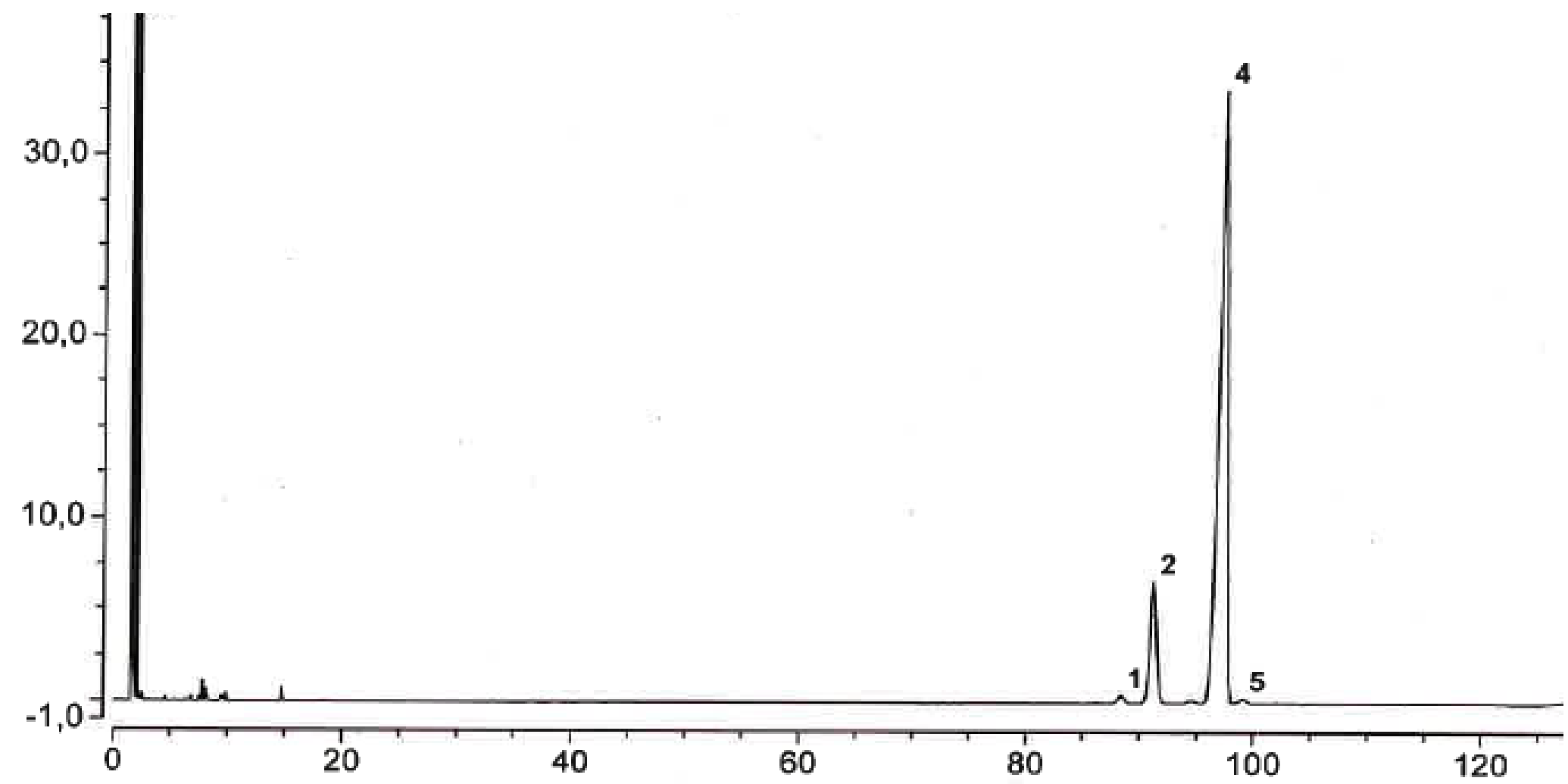

No.	Ret.Time min	Rel.Area %	Peak Name
1	88,41	0,98	*
2	91,15	13,20	*
4	97,38	85,19	**
5	99,10	0,63	**

GC (29.5 m BGB-178, injection temperature: 220 °C; 70 °C – iso 5 min, 1 °C/min, 110 °C; 8 °C/min, 220 °C iso 10 min, 0.5 bar H_2)

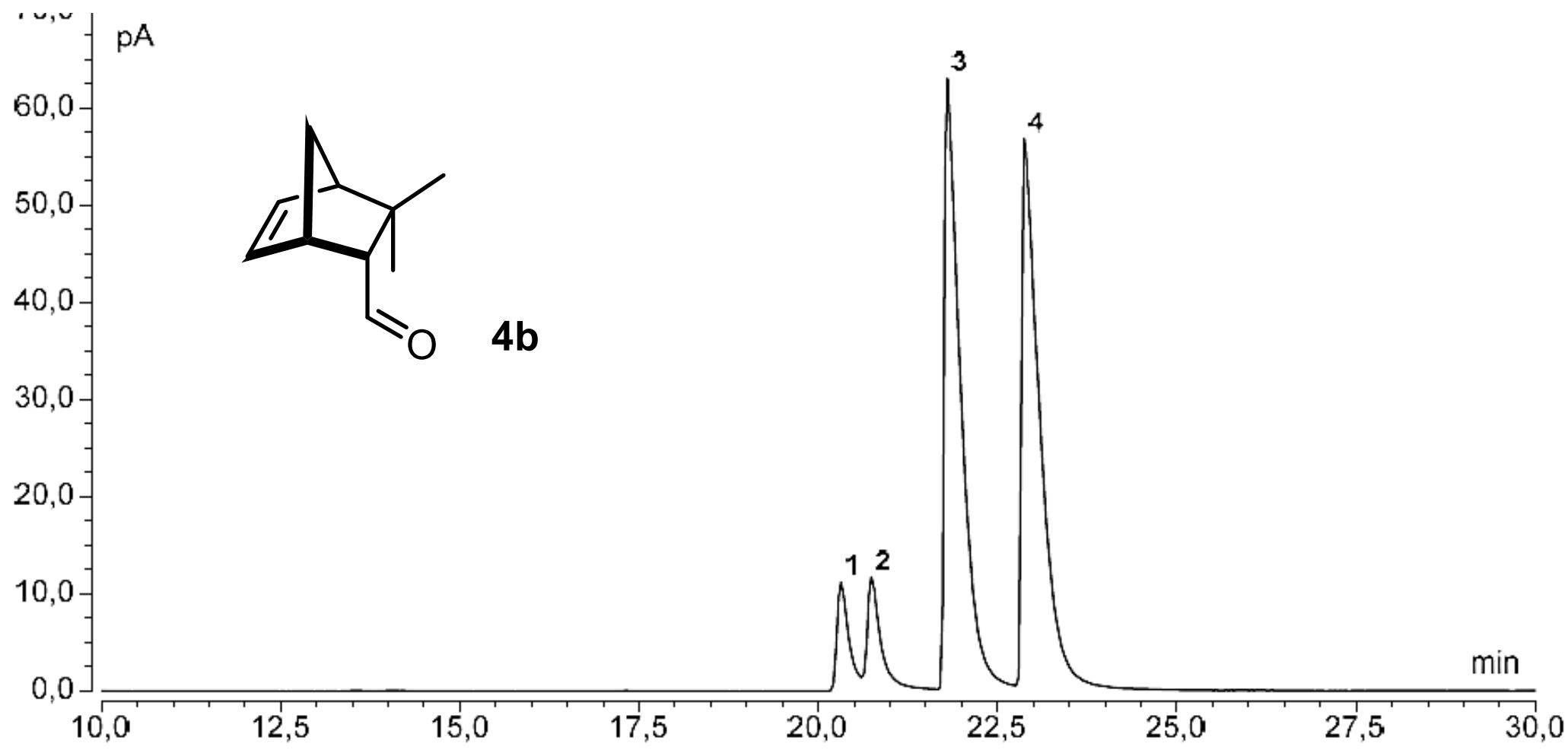

No.	Ret.Time min	Rel.Area %	Peak Name
1	20,32	5,51	Component 1
2	20,74	6,67	Component 2
3	21,80	43,71	Component 3
4	22,87	44,11	Component 4

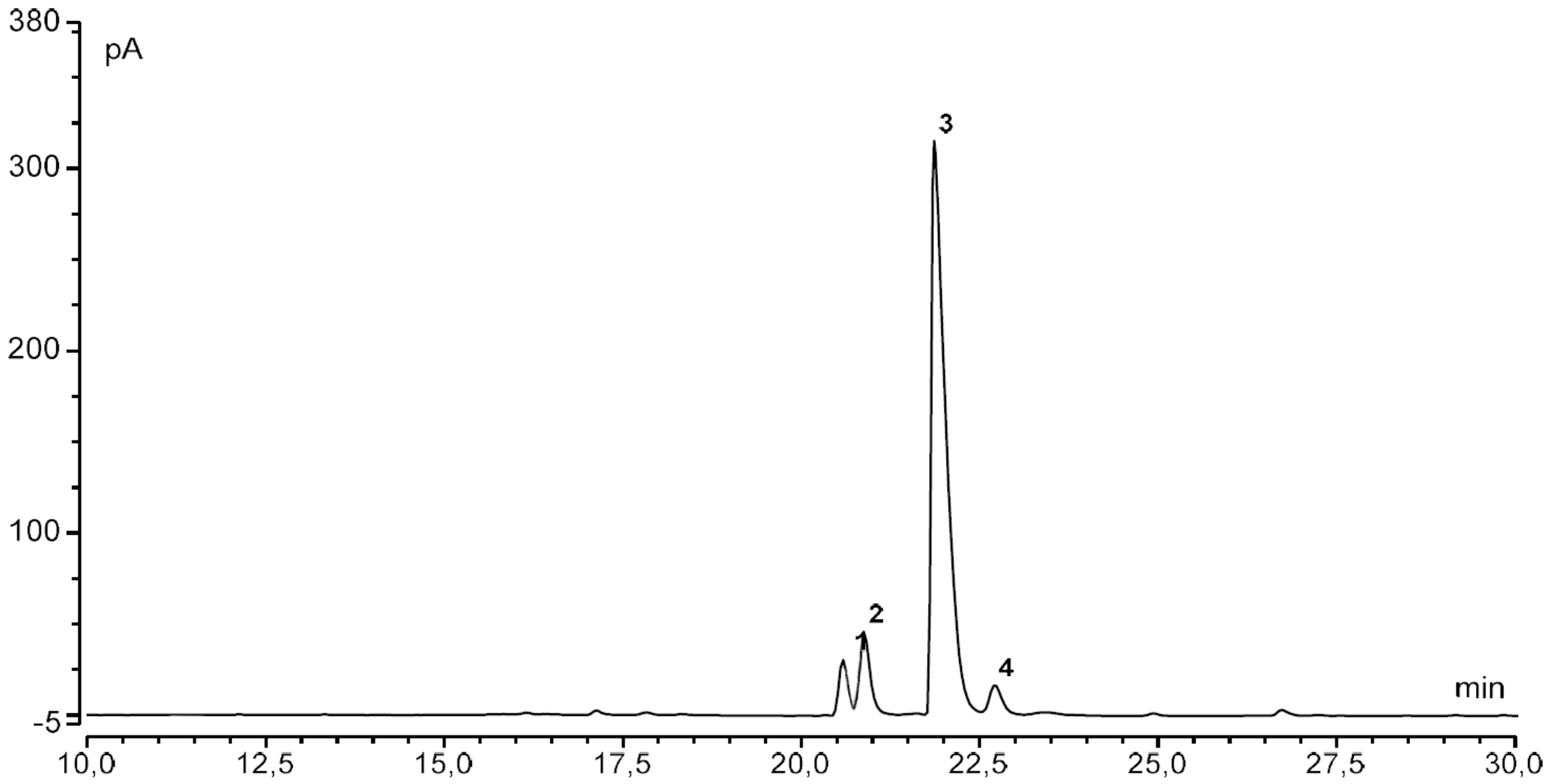

No.	Ret.Time min	Rel.Area %	Peak Name
1	20,58	5,56	Component 1
2	20,97	7,93	Component 2
3	22,43	82,89	Component 3
4	22,91	3,62	Component 4

GC (29.5 m BGB-178, injection temperature: 220 °C; 100 °C – iso 5 min, 2 °C/min, 180 °C; 5 °C/min, 220 °C iso 10 min, 0.5 bar H_2)

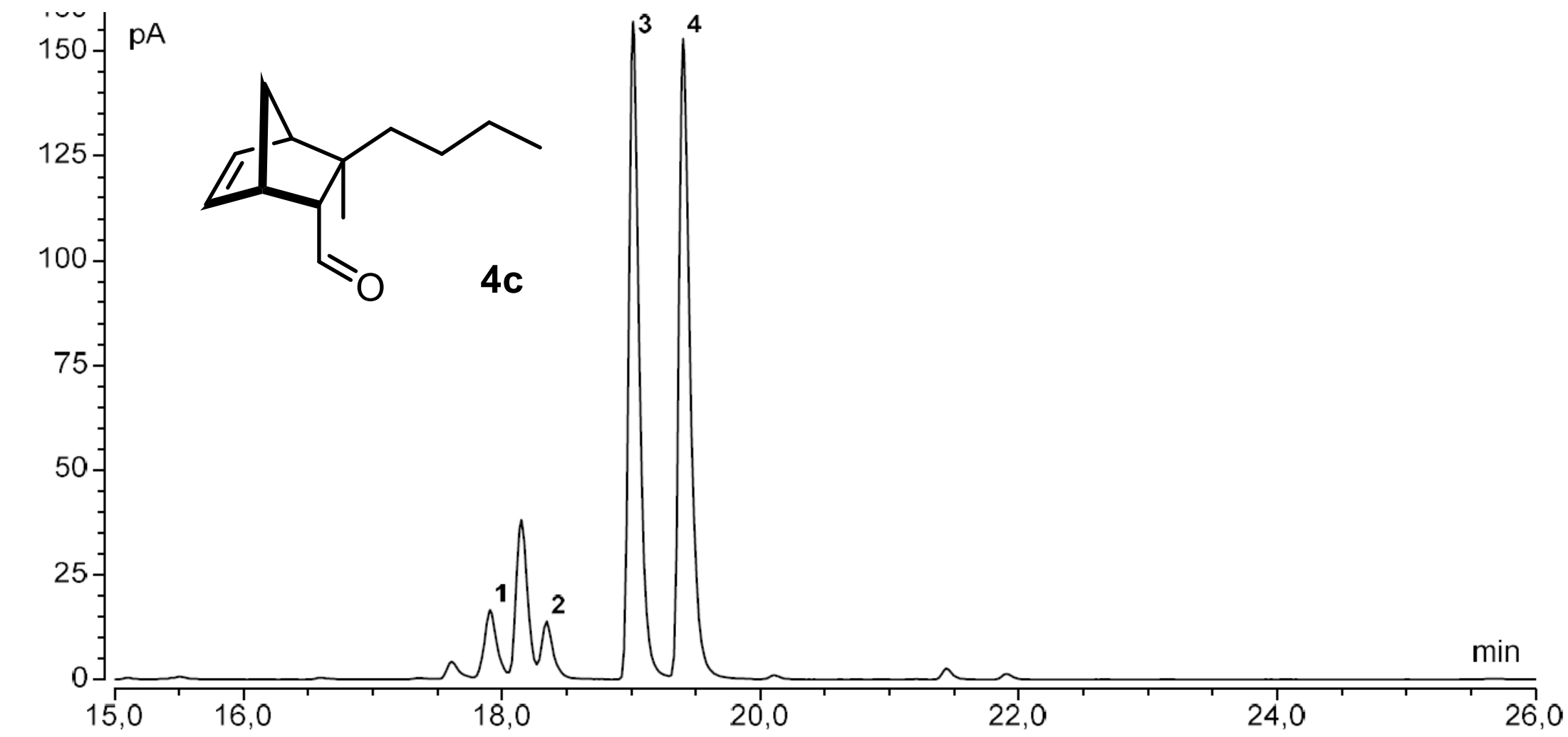

No.	Ret.Time min	Rel.Area %	Peak Name
1	17,91	5,29	Component 1
2	18,34	4,09	Component 2
3	19,01	44,27	Component 3
4	19,40	46,35	Component 4

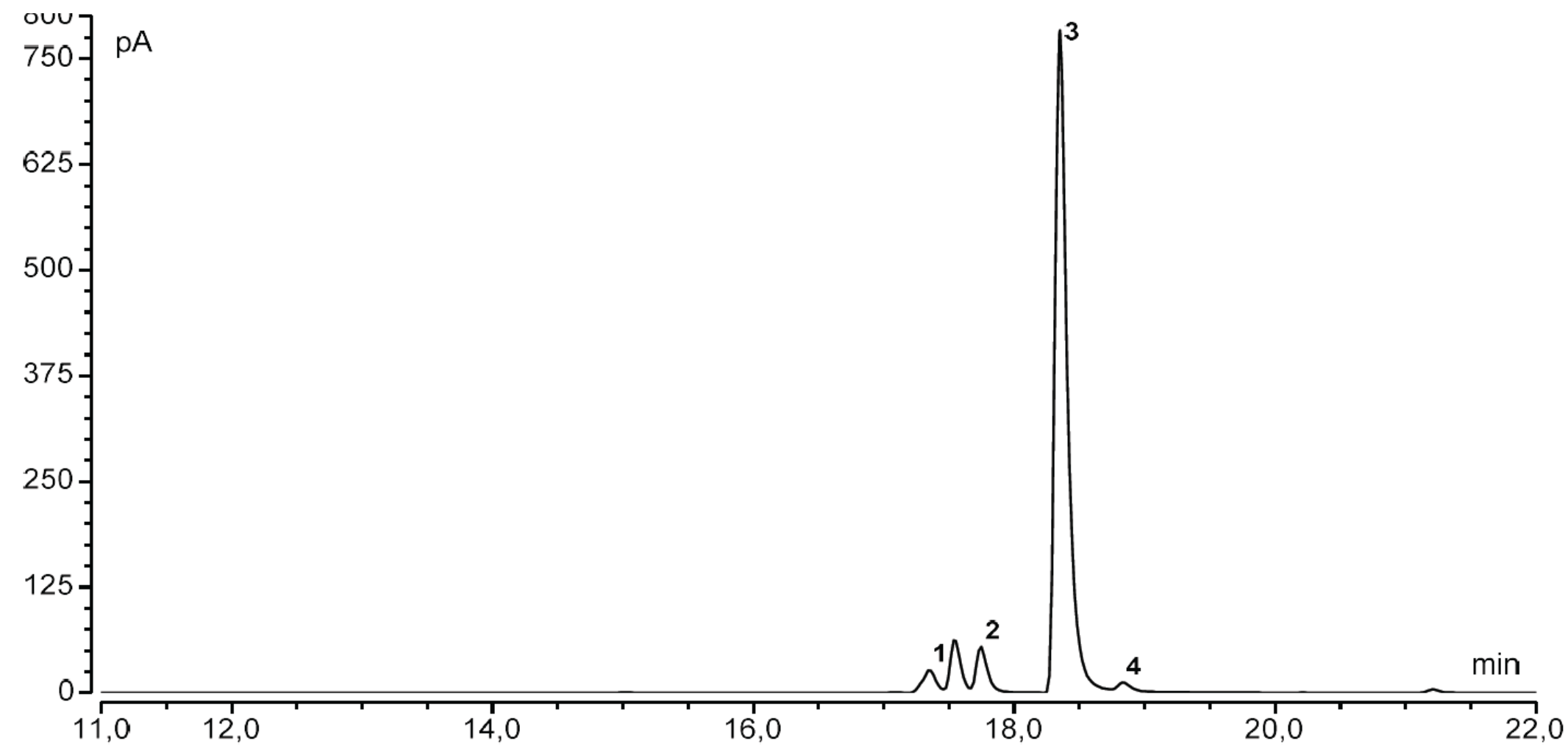

No.	Ret.Time min	Rel.Area %	Peak Name
1	17,35	3,22	Component 1
2	17,74	5,47	Component 2
3	18,35	89,55	Component 3
4	18,83	1,76	Component 4

HPLC (Chiralpak IB-N3, 100% *n*-heptane, 298 K, 220 nm)

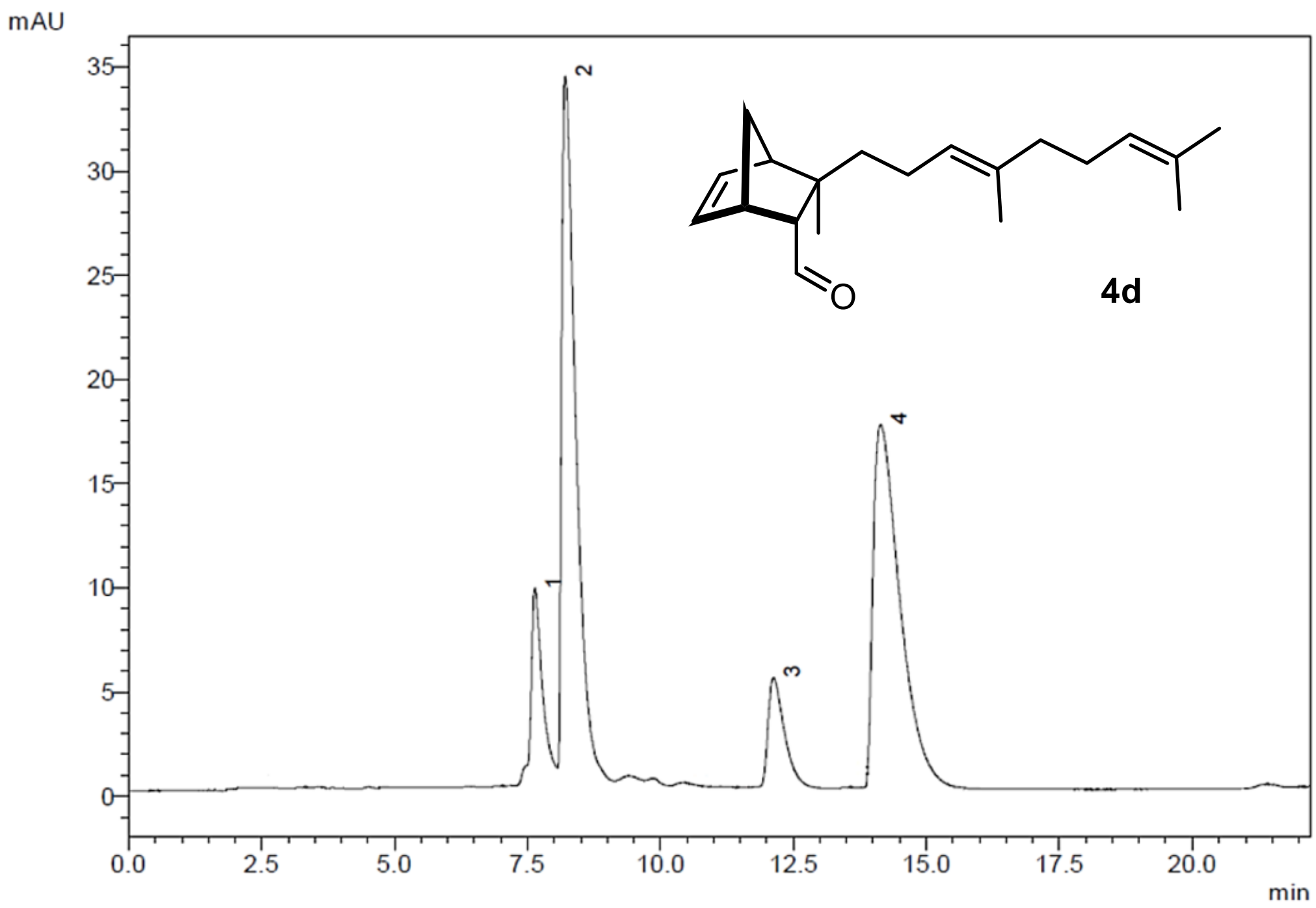

Peak #	Ret. Time	Area %	Name
1	7.64	10.25	E1/D1
2	8.21	41.99	E1/D2
3	12.13	8.05	E2/D1
4	14.14	39.71	E2/D2
Total		100.00	

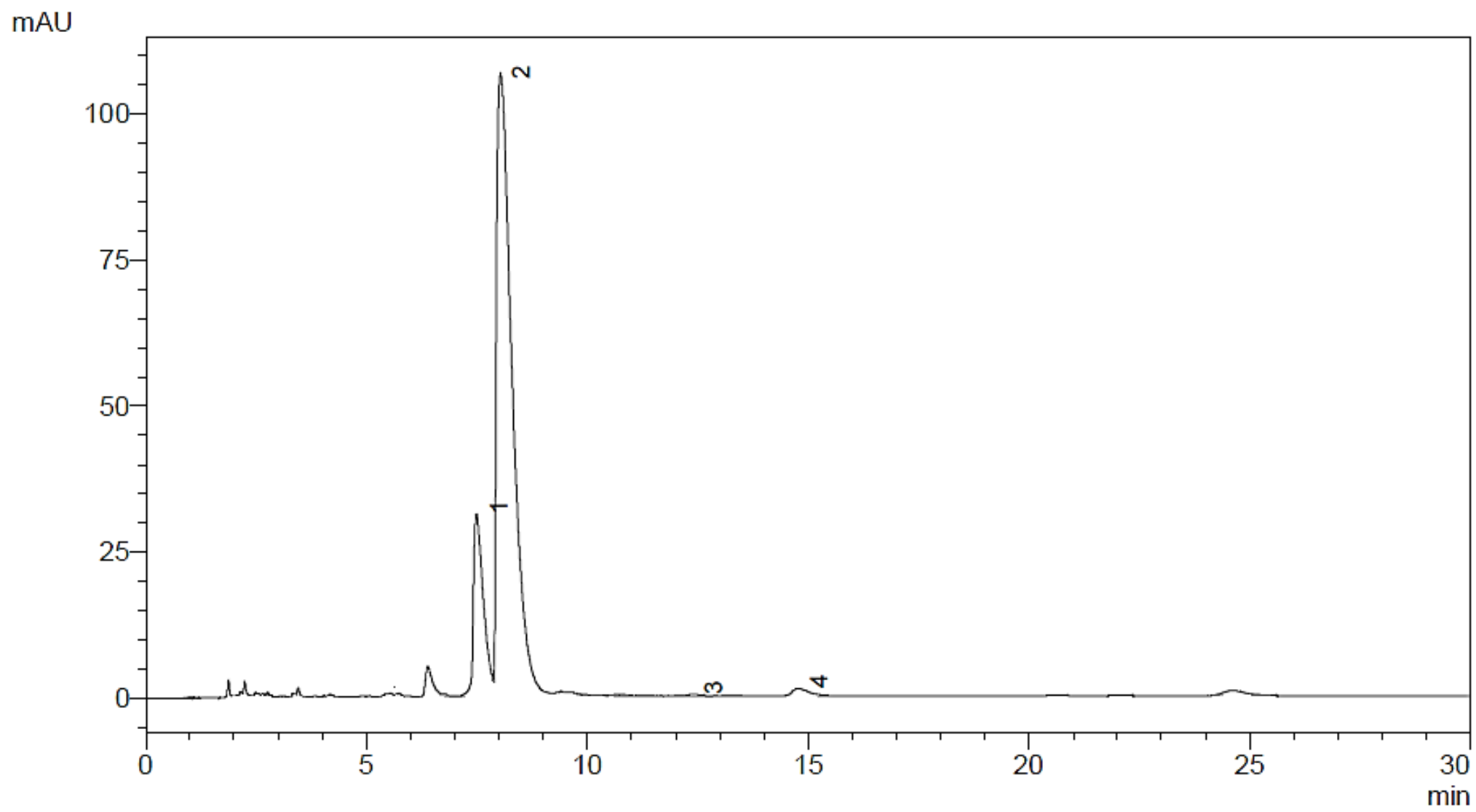

Peak #	Ret. Time	Area %	Name
1	7.53	15.44	E1/D1
2	8.04	83.34	E1/D2
3	12.40	0.14	E2/D1
4	14.77	1.08	E2/D2
Total		100.00	

HPLC (Chiralpak IB-N3, 100% *n*-heptane, 298 K, 220 nm)

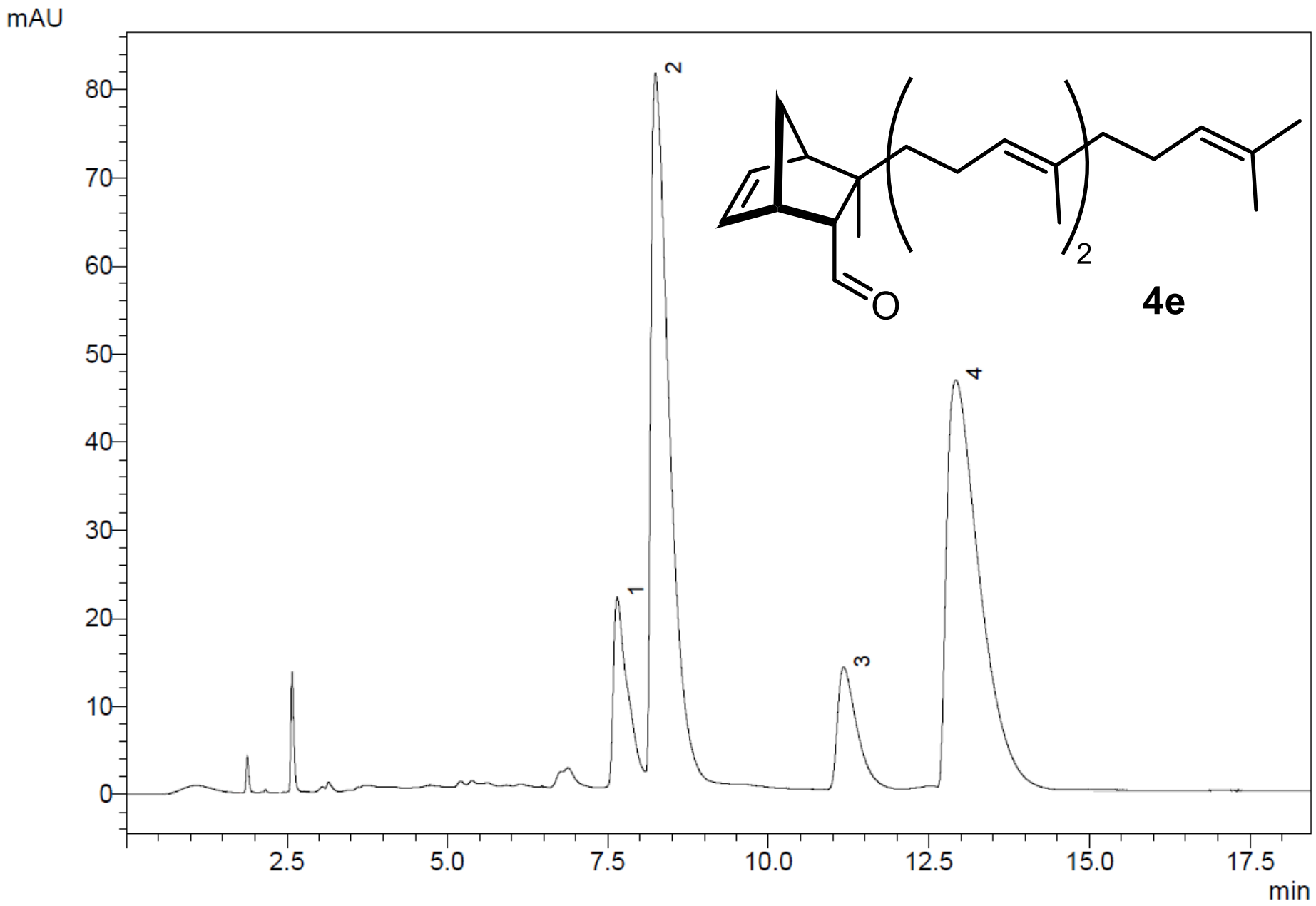

Peak #	Ret. Time	Area %	Name
1	7.64	8.97	E1/D1
2	8.24	41.75	E1/D2
3	11.17	7.69	E2/D1
4	12.92	41.59	E2/D2
Total		100.00	

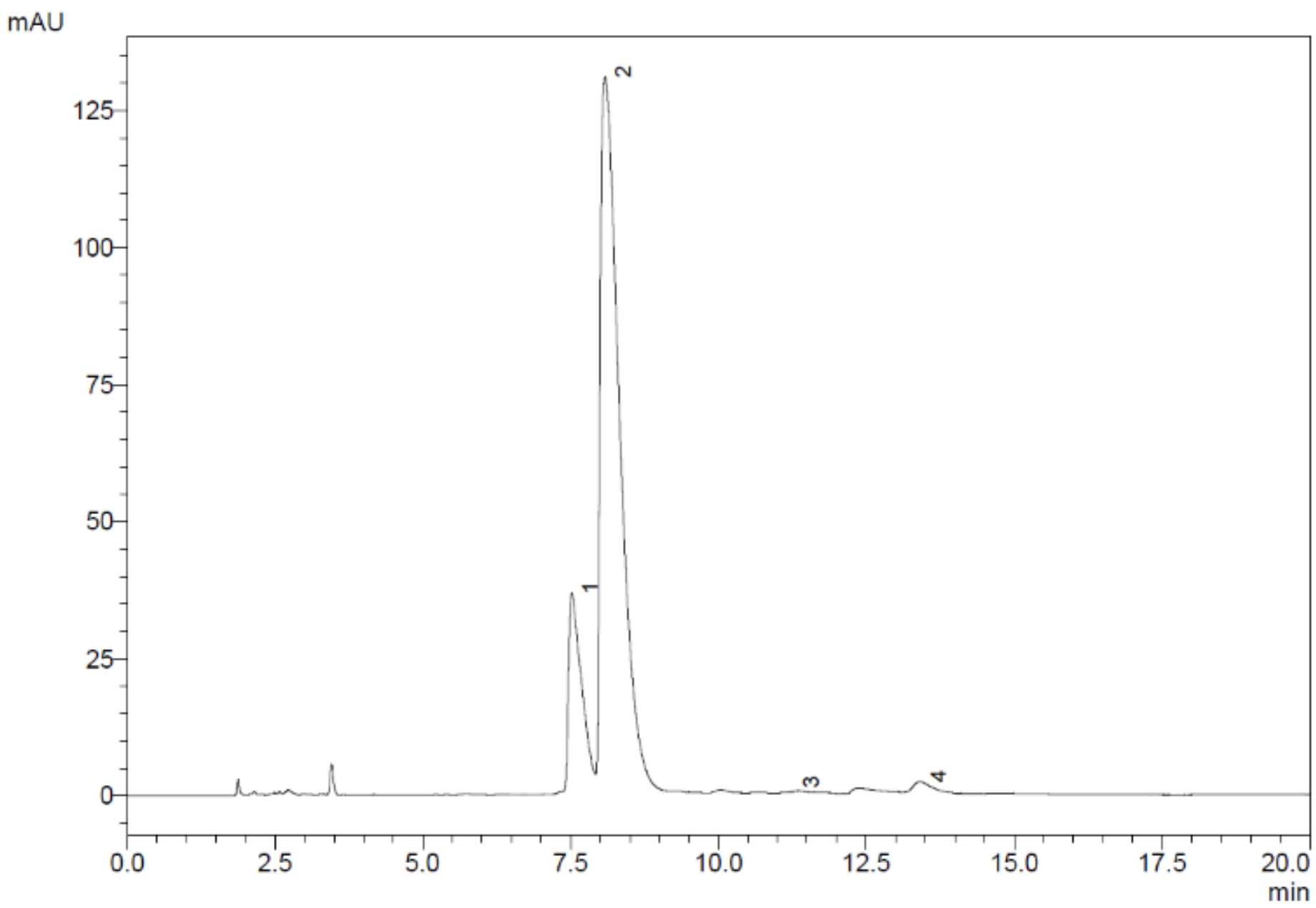

Peak #	Ret. Time	Area %	Name
1	7.52	15.91	E1/D1
2	8.08	82.47	E1/D2
3	11.34	0.18	E2/D1
4	13.40	1.44	E2/D2
Total		100.00	

GC (29.5 m BGB-178, injection temperature: 220 °C; 100 °C – iso 5 min, 2 °C/min, 180 °C; 5 °C/min, 220 °C iso 10 min, 0.5 bar H_2)

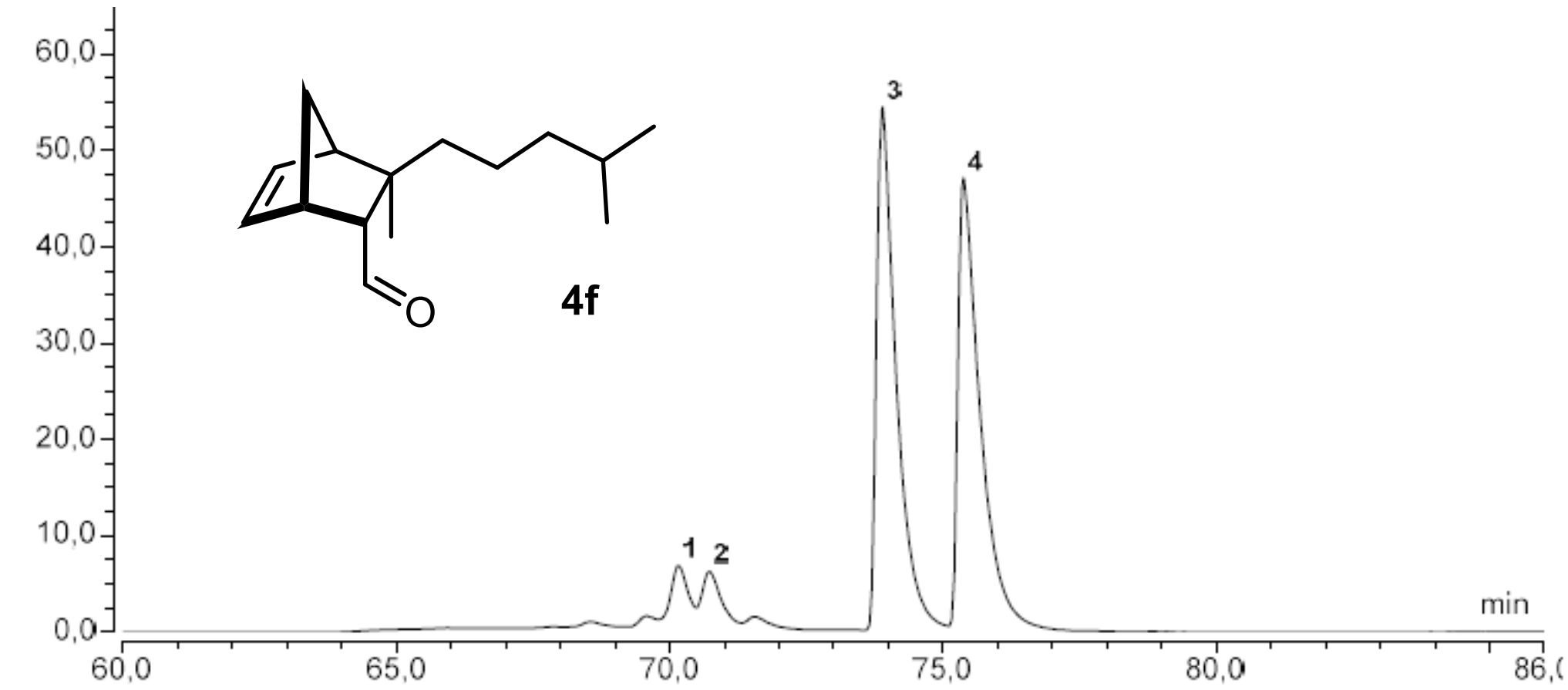

No.	Ret.Time min	Rel.Area %	Peak Name
1	70,16	5,68	Component 1
2	70,73	6,06	Component 2
3	73,90	44,76	Component 3
4	75,38	43,50	Component 4

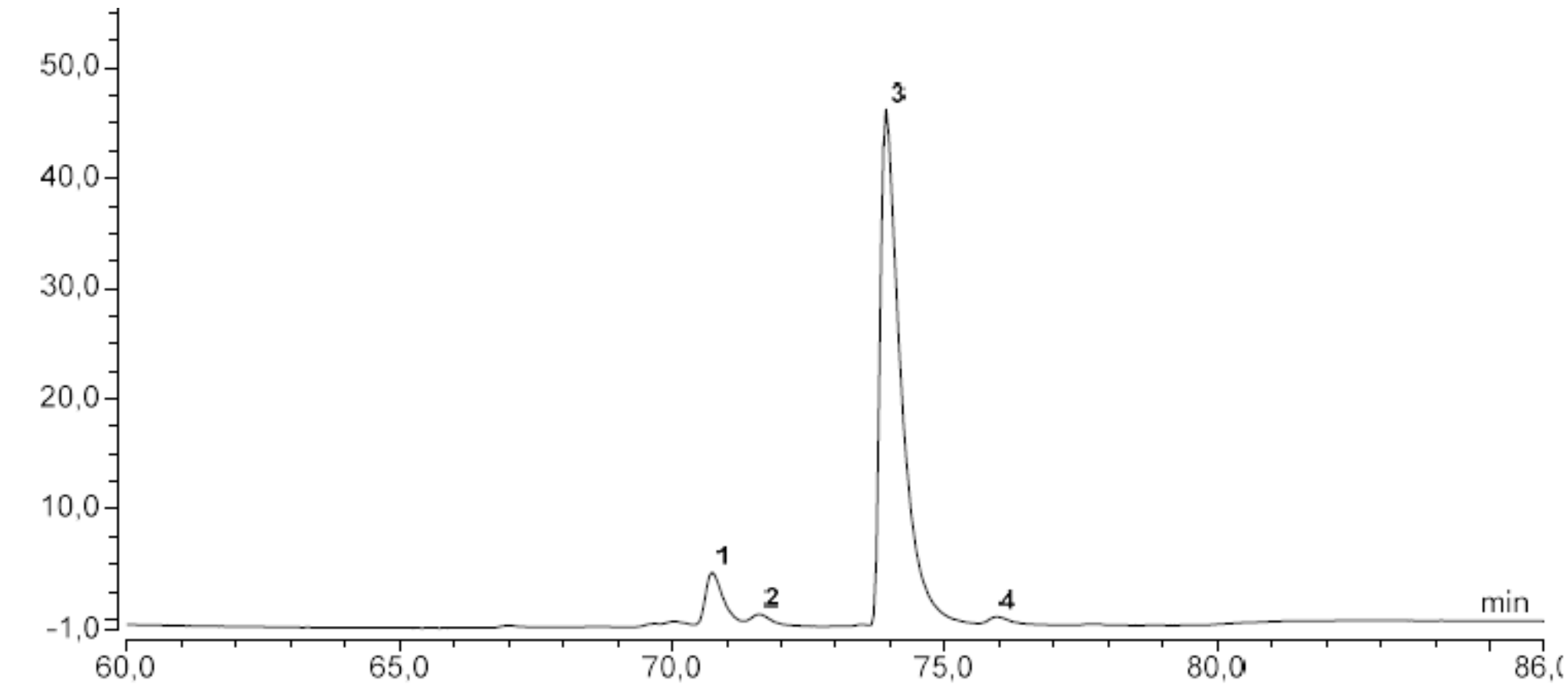

No.	Ret.Time min	Rel.Area %	Peak Name
1	70,73	7,79	Component 1
2	71,61	1,86	Component 2
3	73,93	89,15	Component 3
4	75,94	1,20	Component 4

2D HPLC (1st dimension: PVA-SIL, 100% *n*-heptane, 308 K; 2nd dimension: Chiralcel OD-3, *n*-heptane/2-propanol = 99.5:0.5, 298 K; 210 nm)

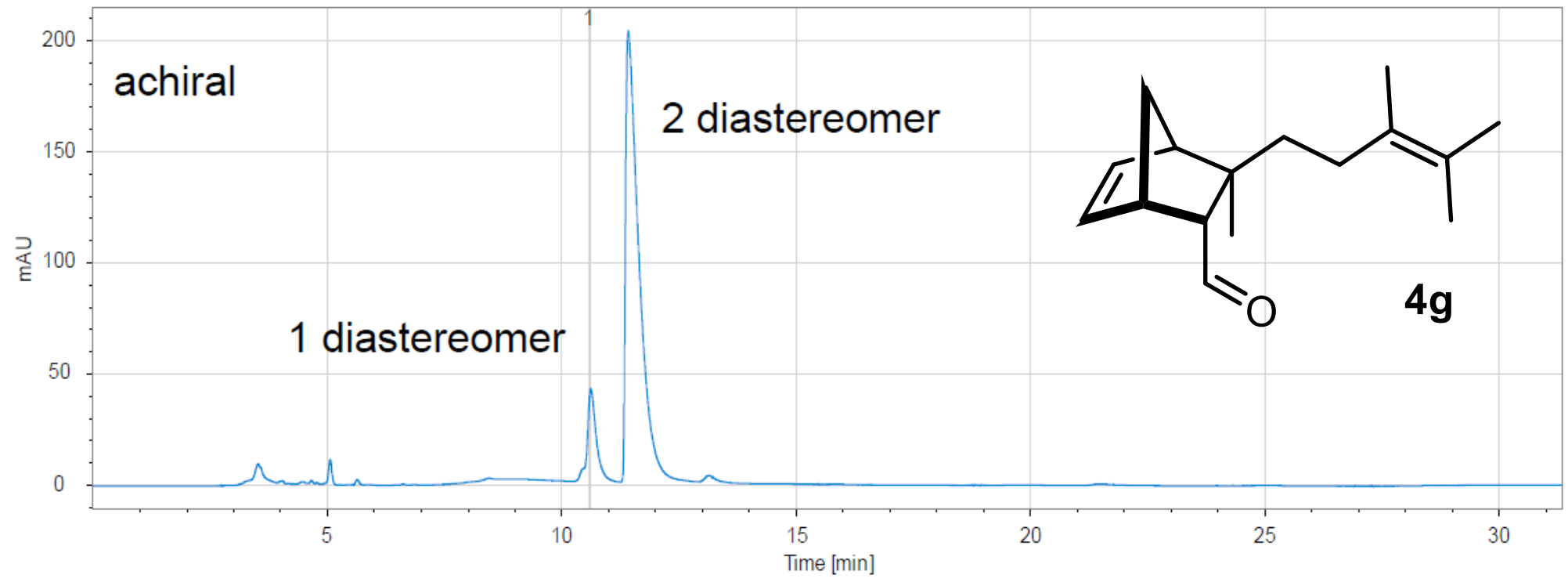

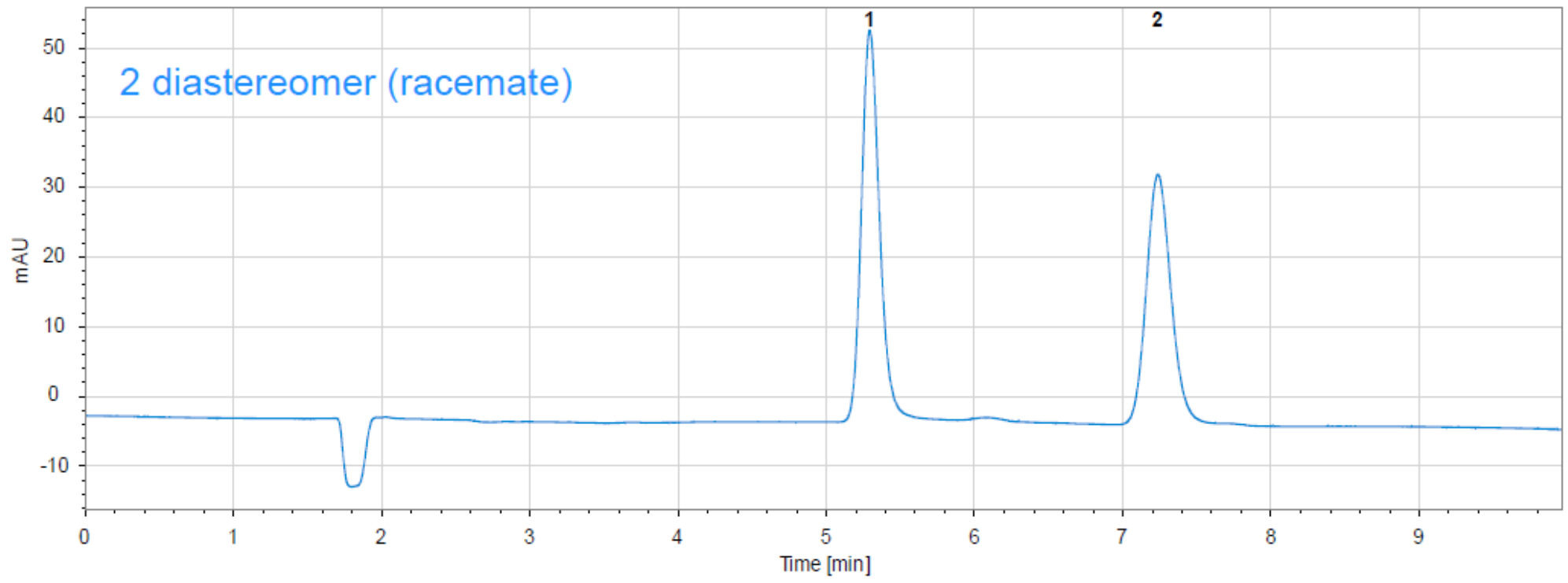

Compound	Cut	Ret.Time	Area	Width	Height	Symmetry
1	1	5.293	458.570<br>**458.570**	0.137	55.601	0.846
2	1	7.237	410.476<br>**410.476**	0.191	35.833	0.845

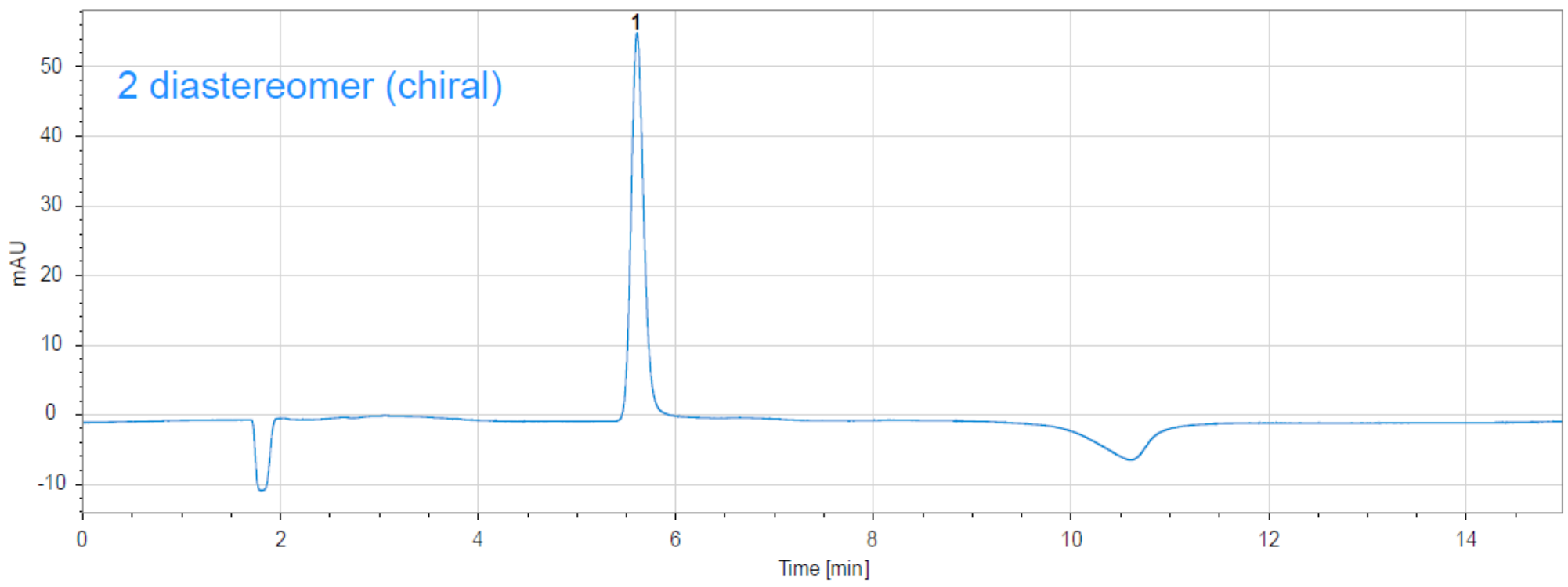

Compound	Cut	Ret.Time	Area	Width	Height	Symmetry
1	1	5.607	495.992<br>**495.992**	0.135	55.485	0.793

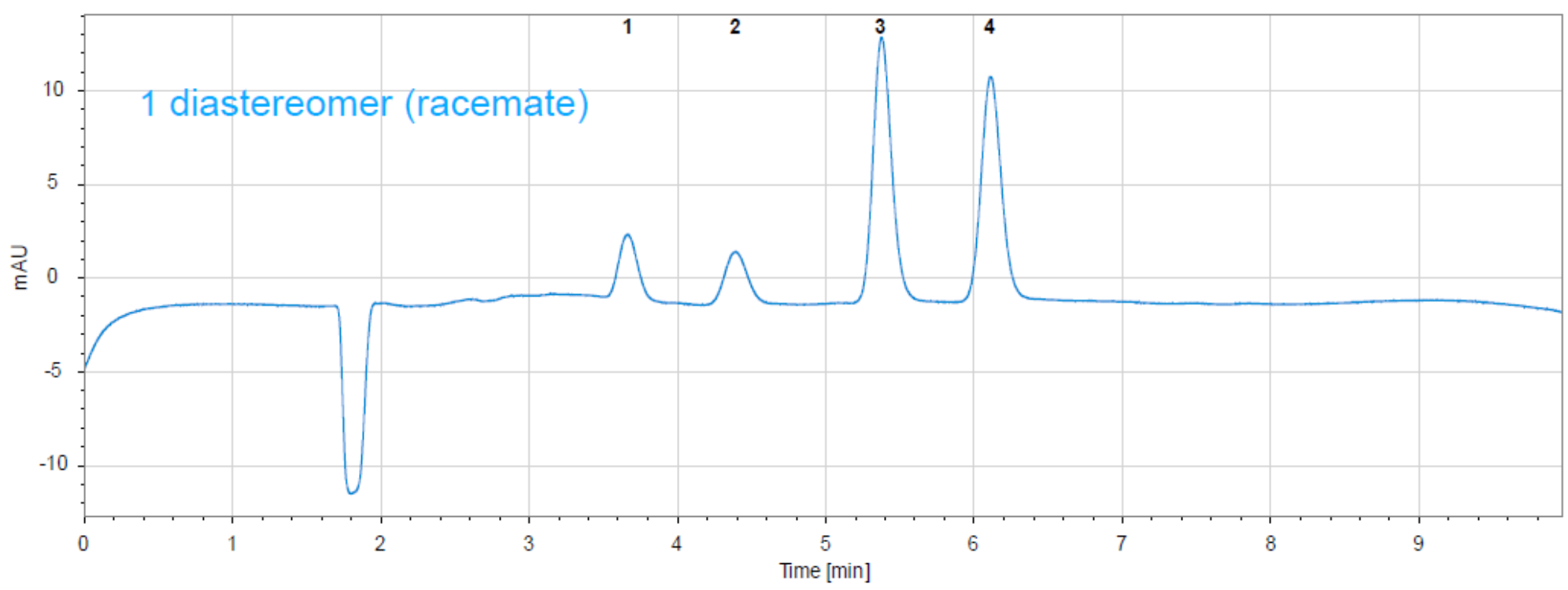

Compound	Cut	Ret.Time	Area	Width	Height	Symmetry
1	1	3.664	30.070<br>**30.070**	0.105	3.457	0.888
2	1	4.389	29.965<br>**29.965**	0.125	2.840	0.866
3	1	5.375	123.201<br>**123.201**	0.119	14.126	0.809
4	1	6.110	112.647<br>**112.647**	0.131	11.971	0.846

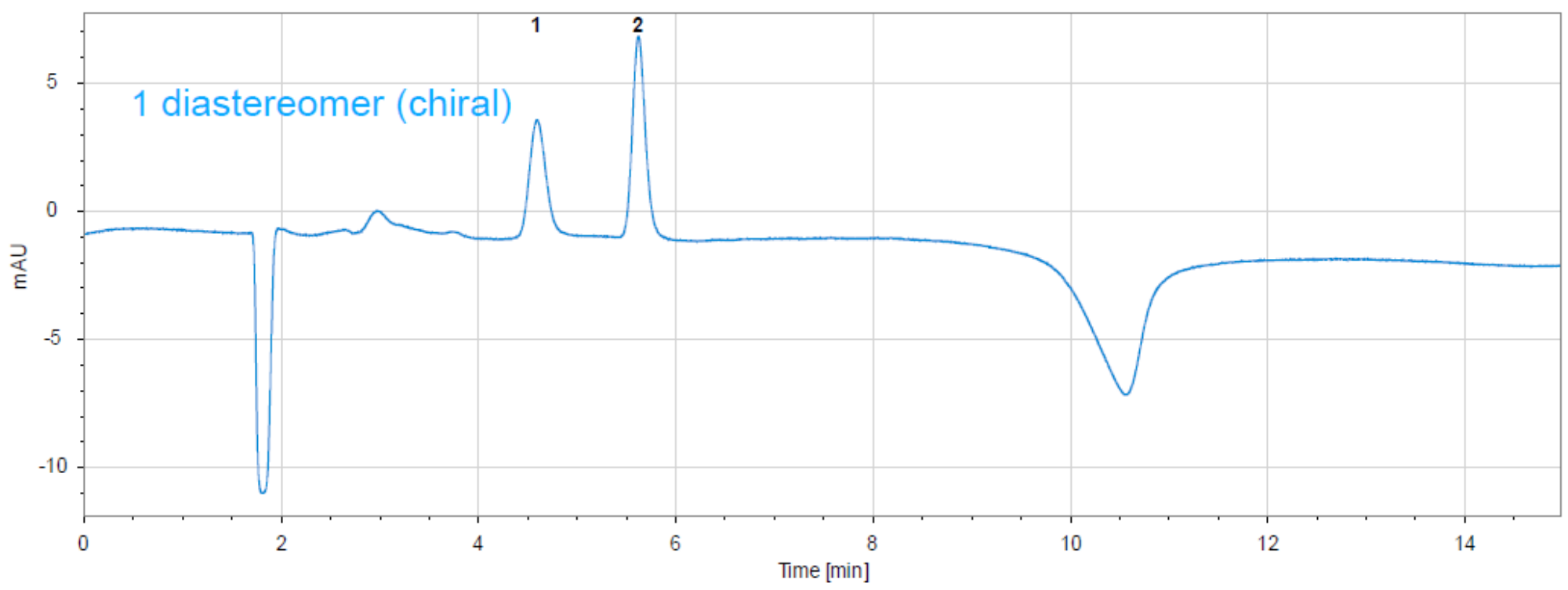

Compound	Cut	Ret.Time	Area	Width	Height	Symmetry
1	1	4.593	53.140<br>**53.140**	0.140	4.568	0.819
2	1	5.621	72.264<br>**72.264**	0.115	7.886	0.819

2D HPLC (1st dimension: PVA-SIL, 100% *n*-heptane, 308 K; 2nd dimension: Chiralcel OD-3, *n*-heptane/2-propanol = 99:1, 298 K; 210 nm)

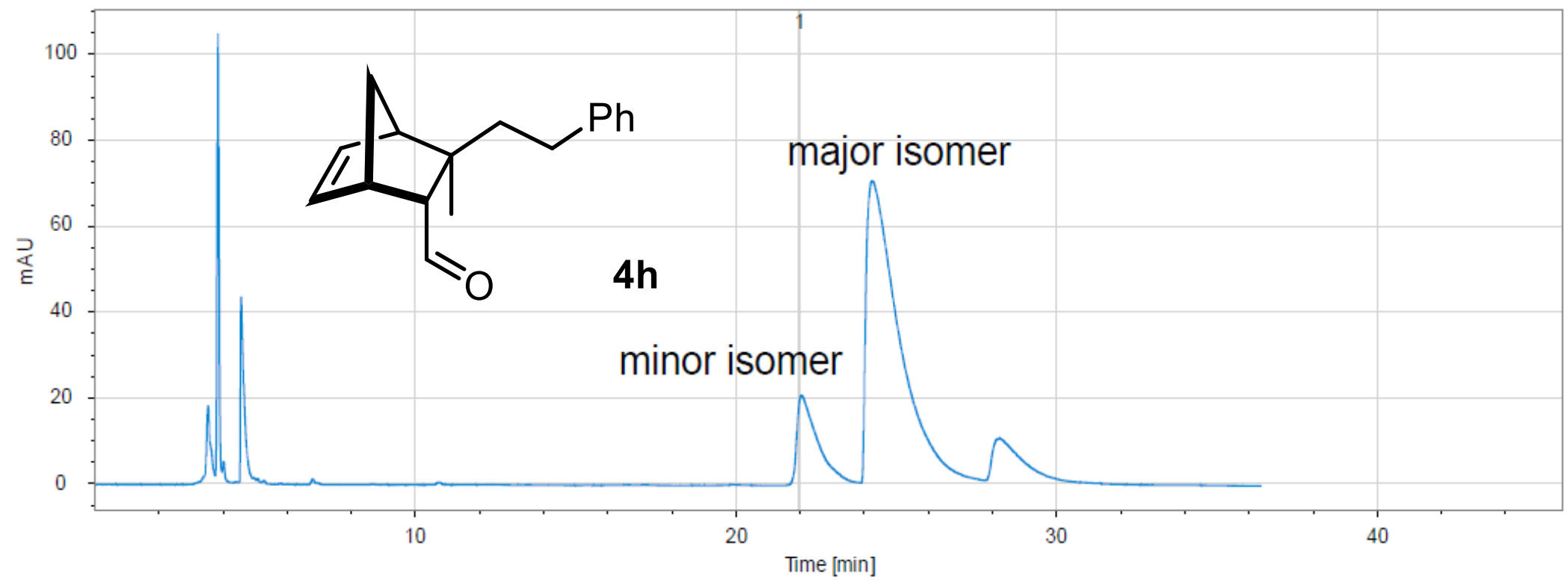

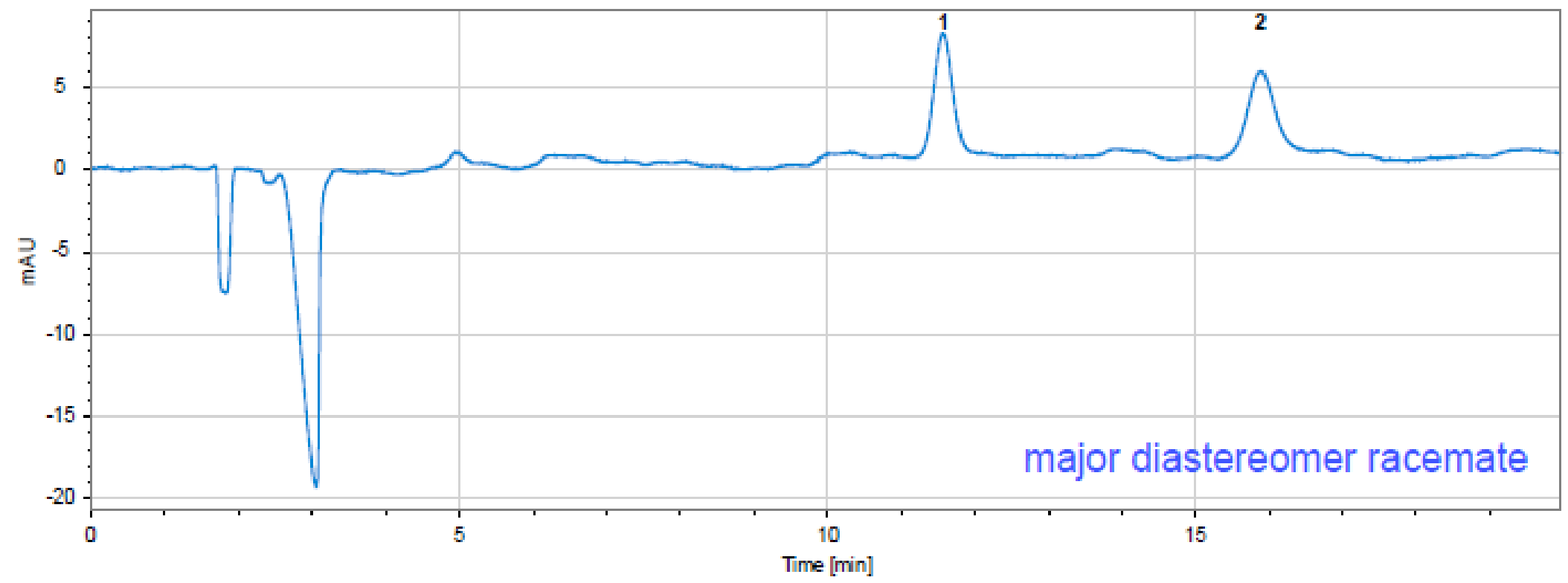

Compound	Cut	Ret.Time	Area	Width	Height	Symmetry
1	1	11.577	133.937<br>**133.937**	0.212	7.470	1.034
2	1	15.889	135.348<br>**135.348**	0.307	5.160	0.900

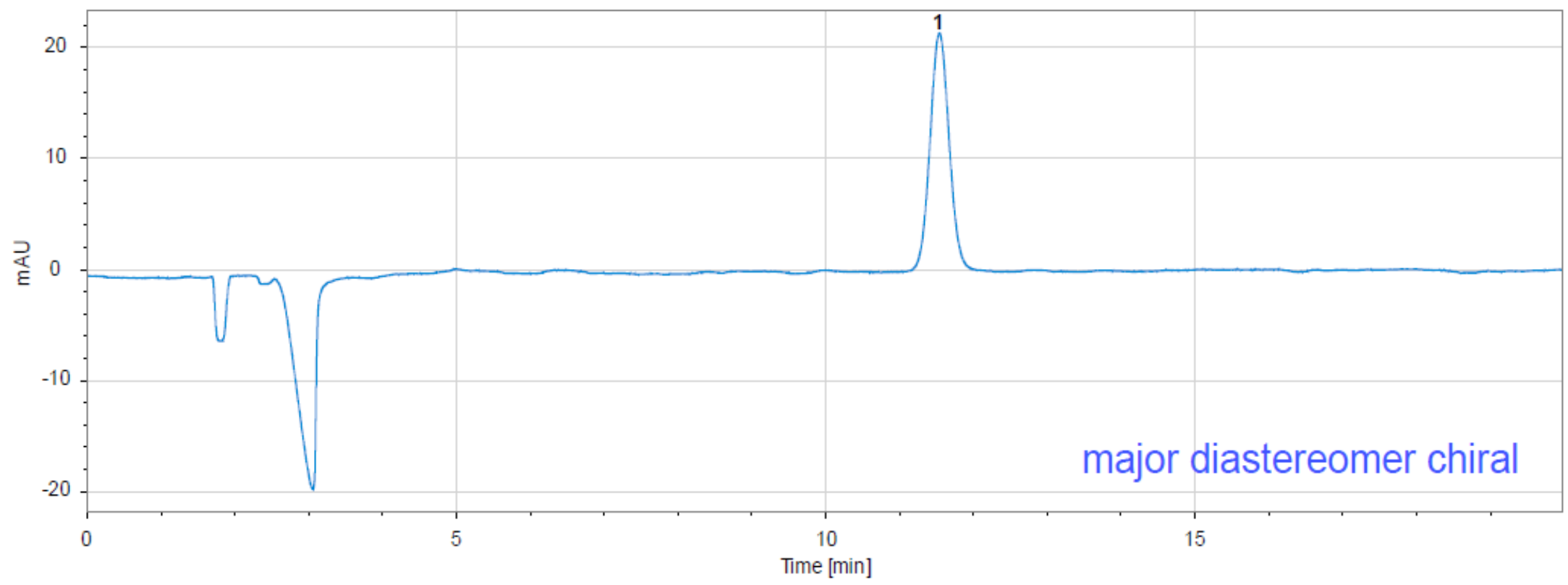

Compound	Cut	Ret.Time	Area	Width	Height	Symmetry
1	1	11.539	386.717<br>**386.717**	0.215	21.432	0.936

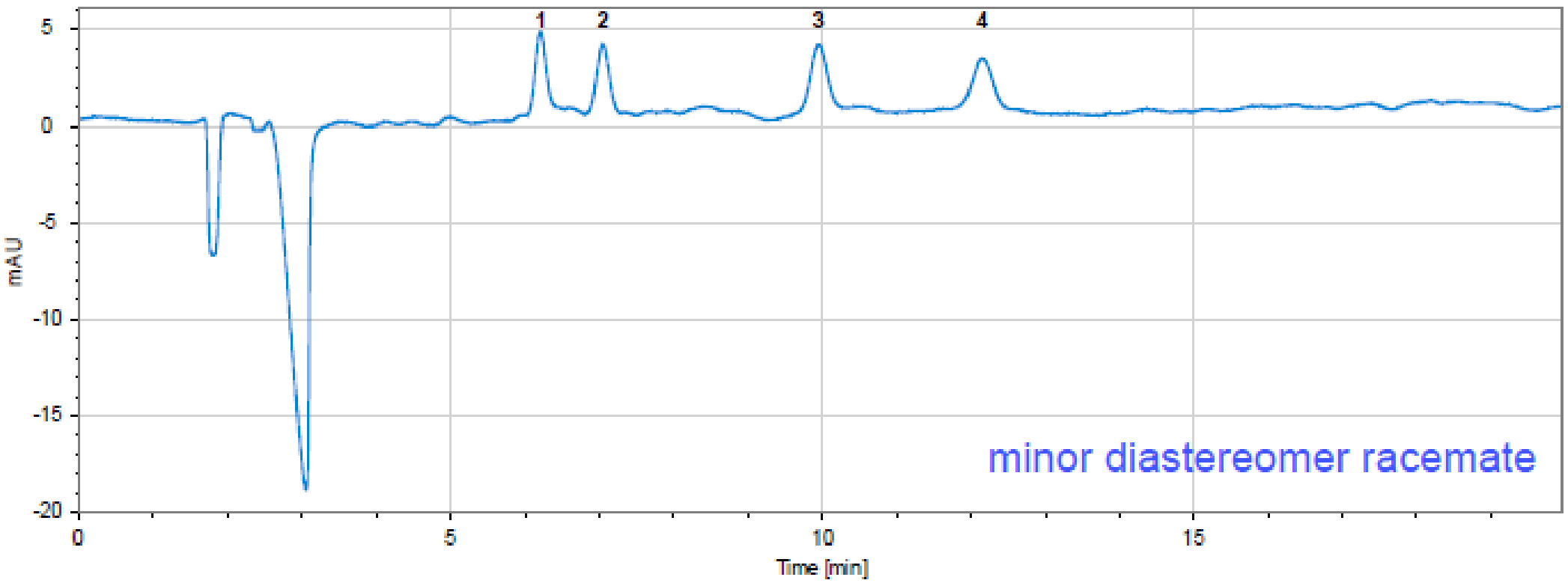

Compound	Cut	Ret.Time	Area	Width	Height	Symmetry
1	1	6.214	44.168<br>**44.168**	0.174	4.231	0.958
2	1	7.054	41.284<br>**41.284**	0.193	3.561	1.013
3	1	9.950	52.399<br>**52.399**	0.254	3.433	0.811
4	1	12.165	47.754<br>**47.754**	0.305	2.613	1.047

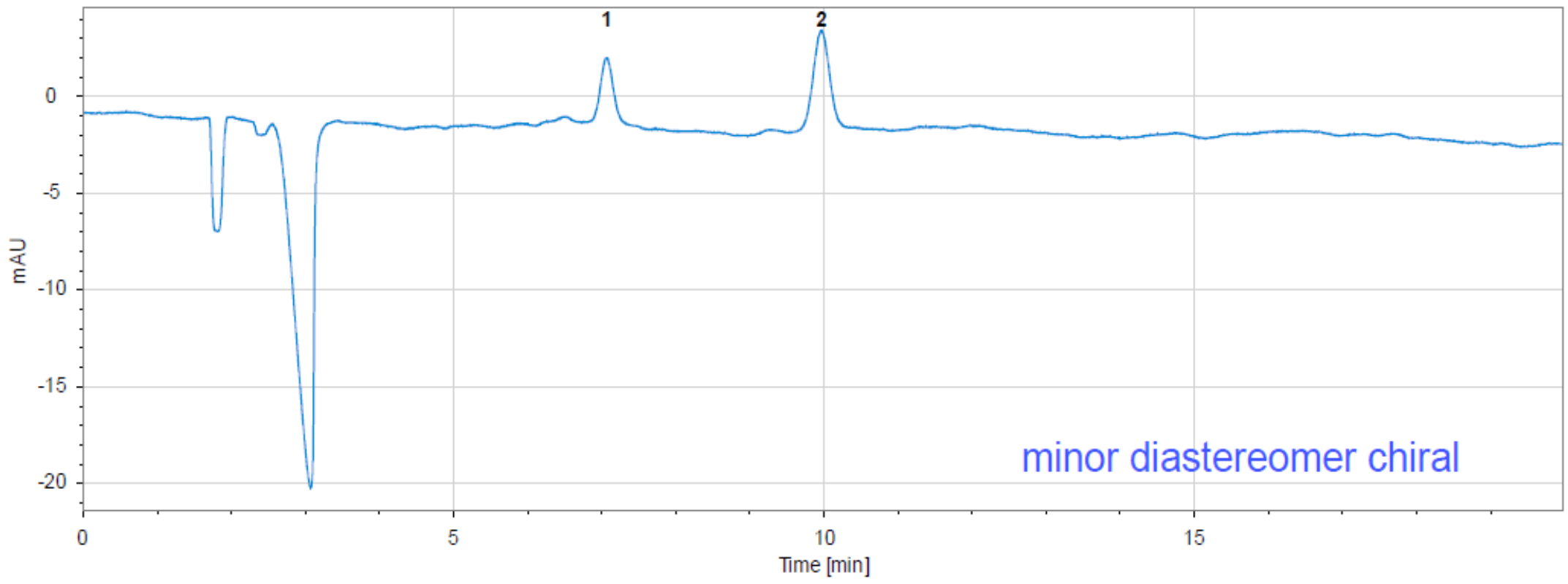

Compound	Cut	Ret.Time	Area	Width	Height	Symmetry
1	1	7.070	45.017<br>**45.017**	0.151	3.507	1.029
2	1	9.967	80.513<br>**80.513**	0.185	5.180	1.008

2D HPLC (1st dimension: YMC PVA-SIL *n*-heptane/2-propanol = 99.8:0.2, 308 K; 2nd dimension: Chiralpak IB-N3, *n*-heptane/2-propanol = 99:1, 298 K; 220 nm)

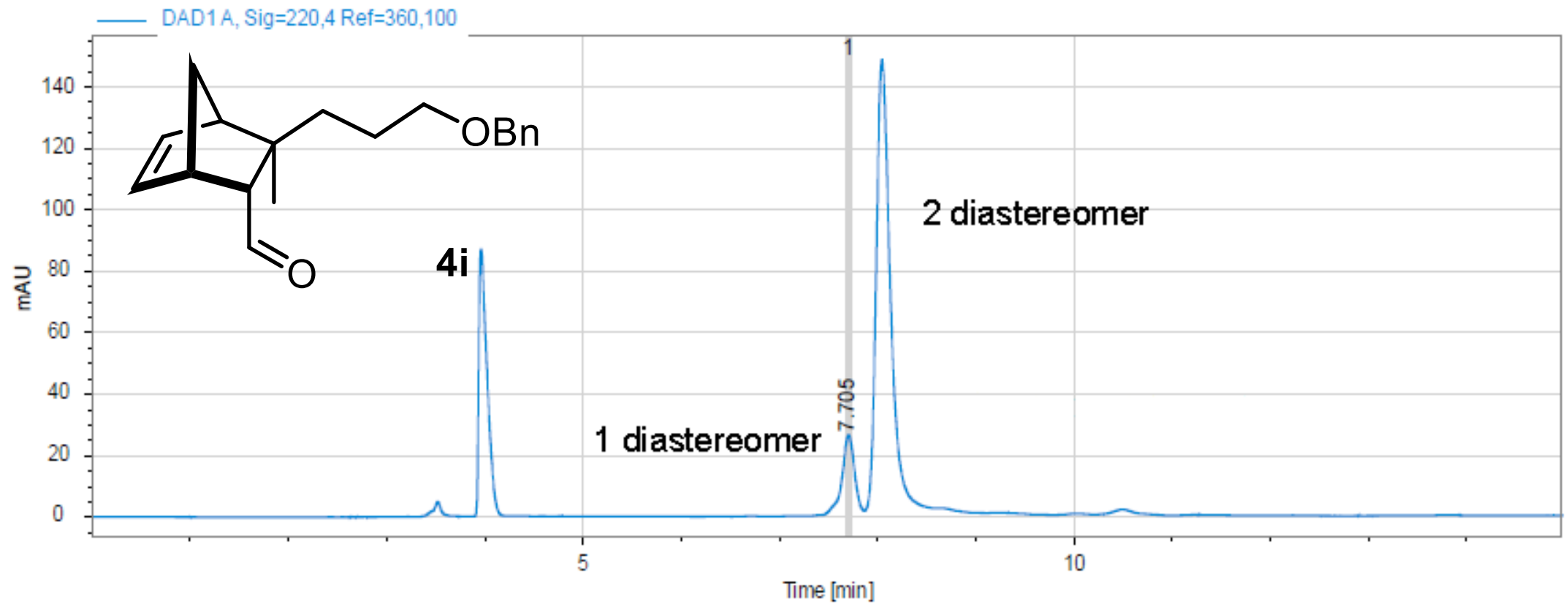

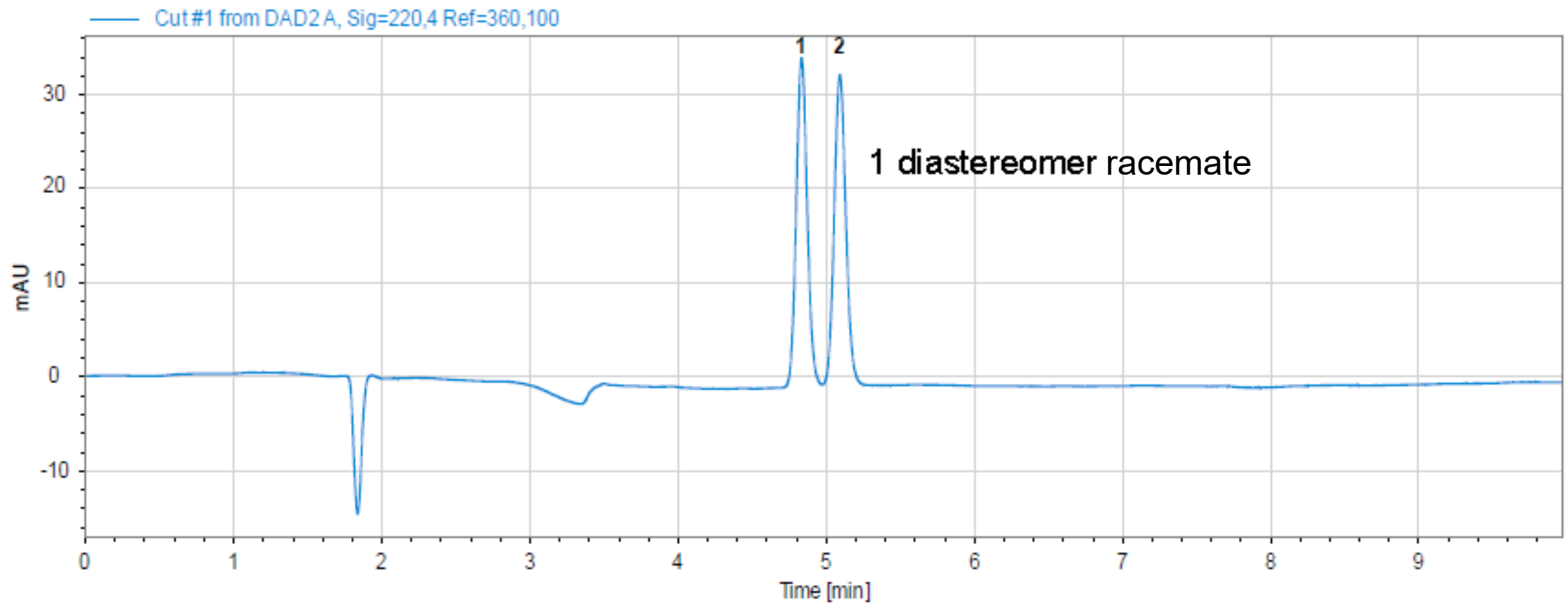

Component	¹D Sampling range [min]	Ret.Time ²D [min]	Area	Area%	
1	7.69 - 7.73	4.833	180.761	49.783	1st enantiomer
2	7.69 - 7.73	5.091	182.334	50.217	2nd enantiomer

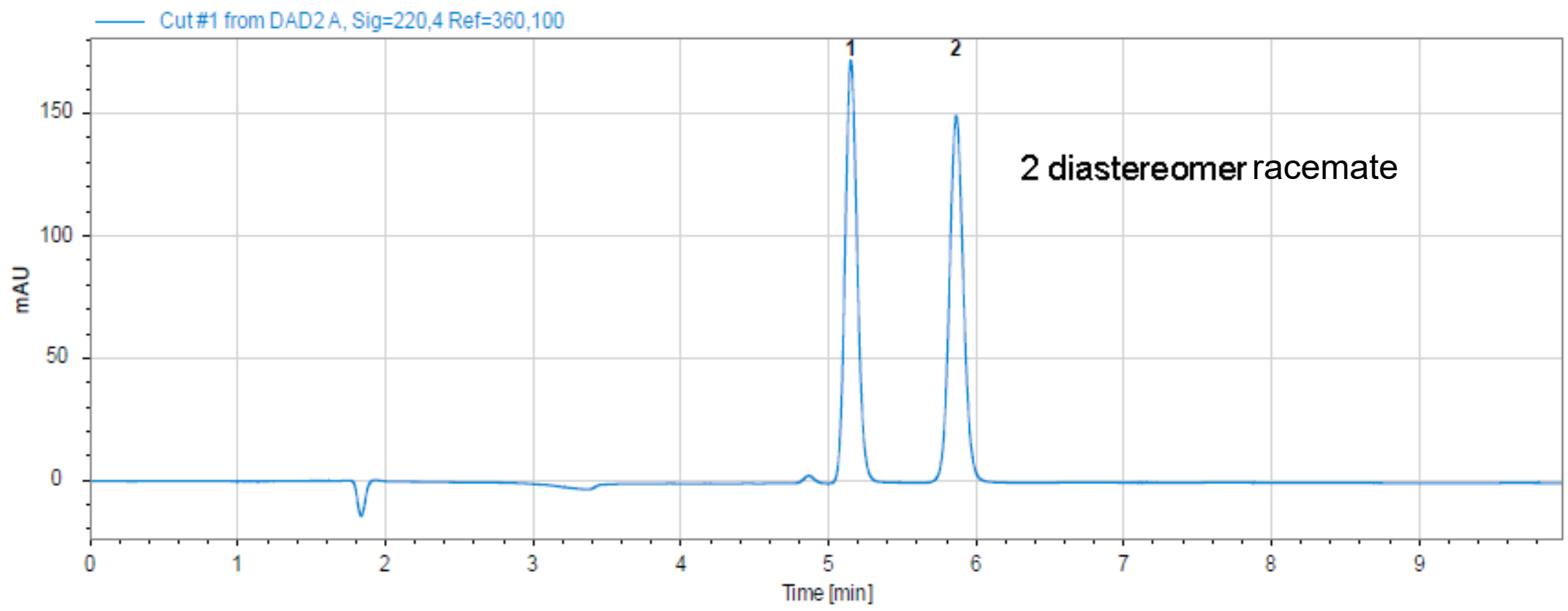

Component	¹D Sampling range [min]	Ret.Time ²D [min]	Area	Area%	
1	7.97 - 8.01	5.151	987.441	49.836	1st enantiomer
2	7.97 - 8.01	5.864	993.938	50.164	2nd enantiomer

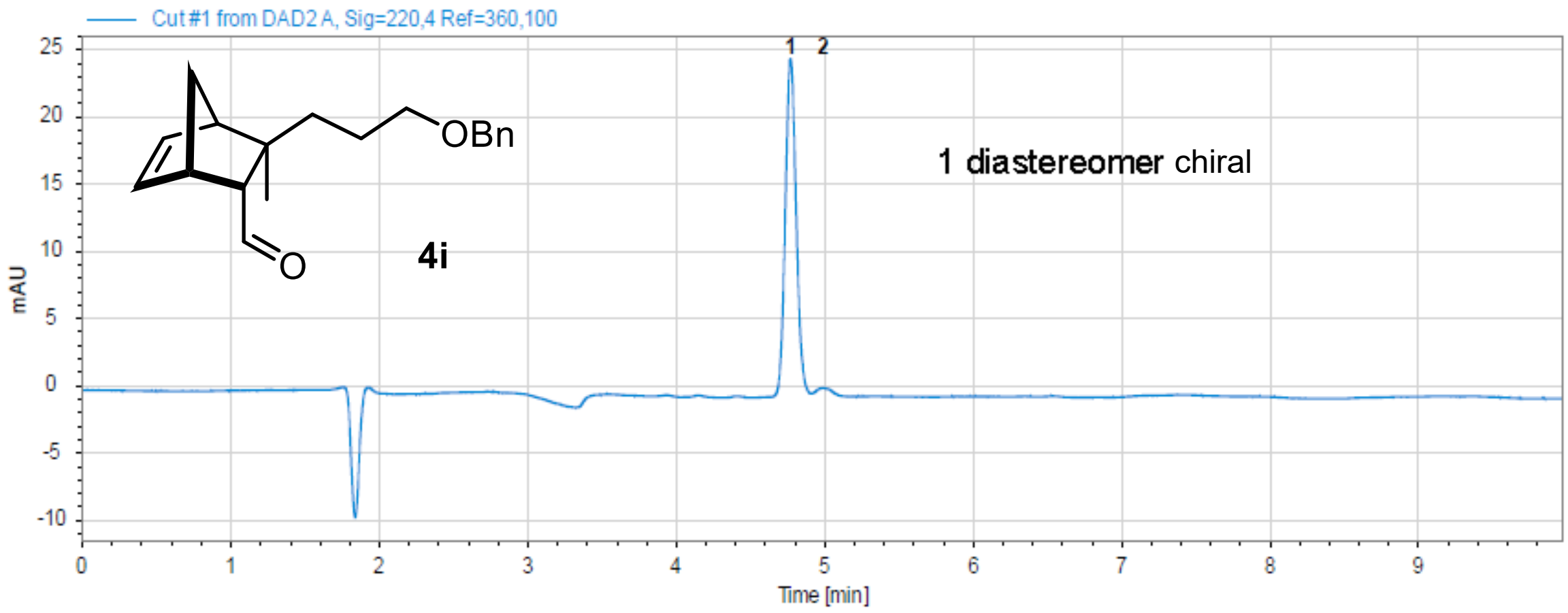

Component	¹D Sampling range [min]	Ret.Time ²D [min]	Area	Area%	
1	7.54 - 7.58	4.769	128.656	96.094	1st enantiomer
2	7.54 - 7.58	4.990	5.230	3.906	2nd enantiomer

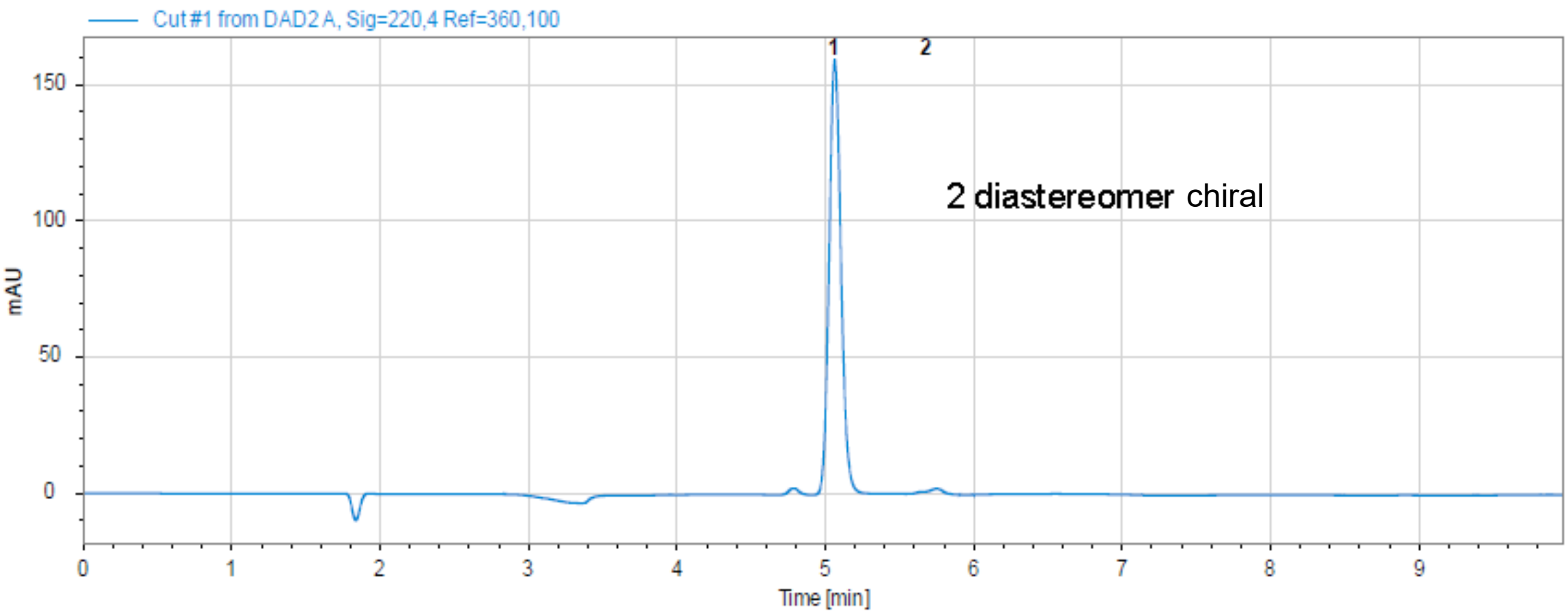

Component	¹D Sampling range [min]	Ret.Time ²D [min]	Area	Area%	
1	7.90 - 7.94	5.063	887.251	98.472	1st enantiomer
2	7.90 - 7.94	5.749	13.399	1.528	2nd enantiomer

GC (24.5 m Ivadex-1, injection temperature: 220 °C; 100 °C – iso 10 min, 1 °C/min, 180 °C – iso 10 min; 5 °C/min, 220 °C – iso 10 min; 0.5 bar H_2)

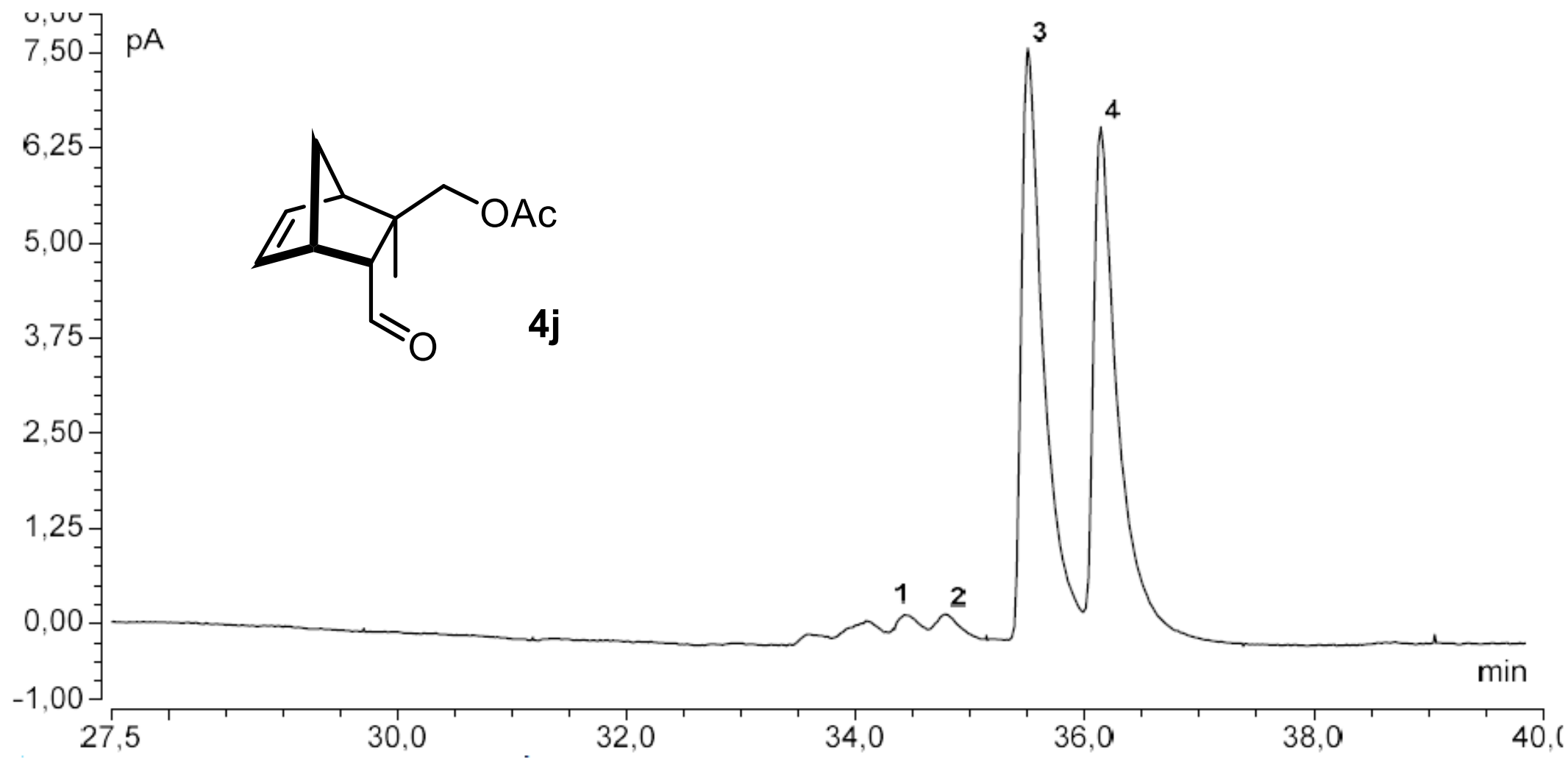

No.	Ret.Time min	Rel.Area %	Peak Name
1	34,09	3,73	Component 1
2	34,79	3,32	Component 2
3	35,50	46,83	Component 3
4	36,14	46,12	Component 4

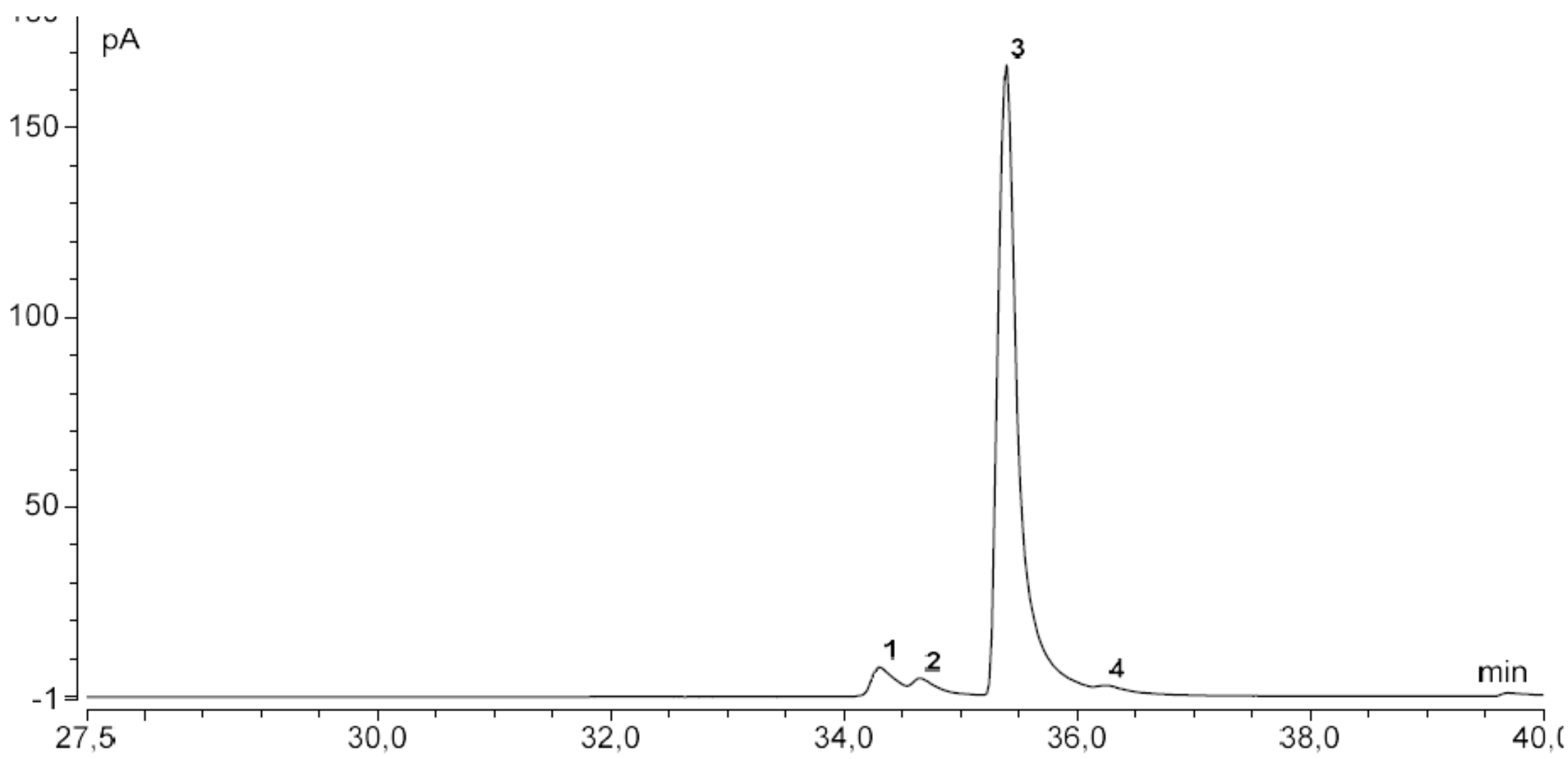

No.	Ret.Time min	Rel.Area %	Peak Name
1	34,30	4,86	Component 1
2	34,66	2,52	Component 2
3	35,39	89,85	Component 3
4	36,24	2,77	Component 4

GC (29.5 m BGB-176, injection temperature: 220 °C; 100 °C – iso 5 min, 1 °C/min, 180 °C; 5 °C/min, 220 °C iso 10 min, 0.5 bar H_2)

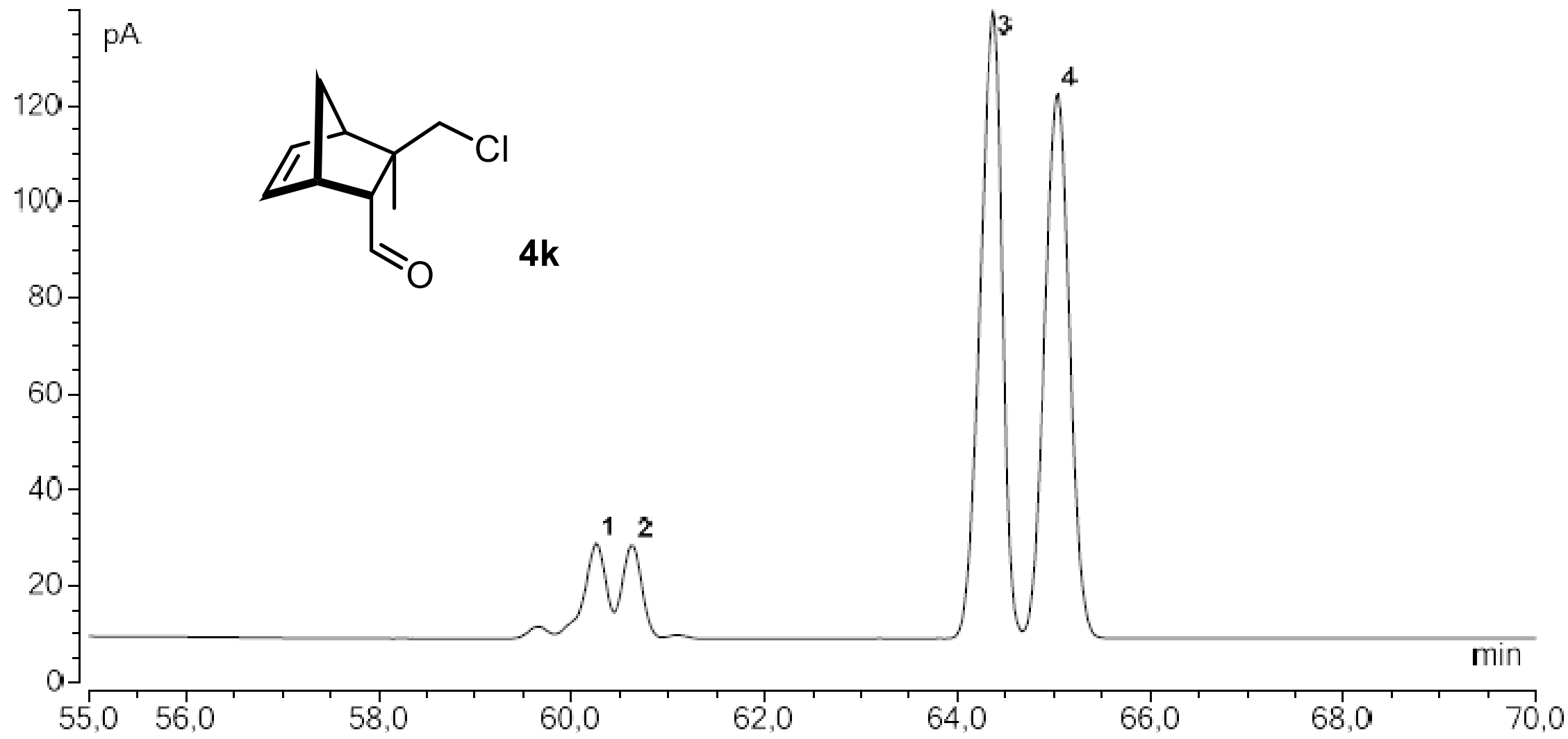

No.	Ret.Time min	area-% %	Peak Name
1	60,26	6,08	Component 1
2	60,63	5,78	Component 2
3	64,37	44,61	Component 3
4	65,04	43,54	Component 4

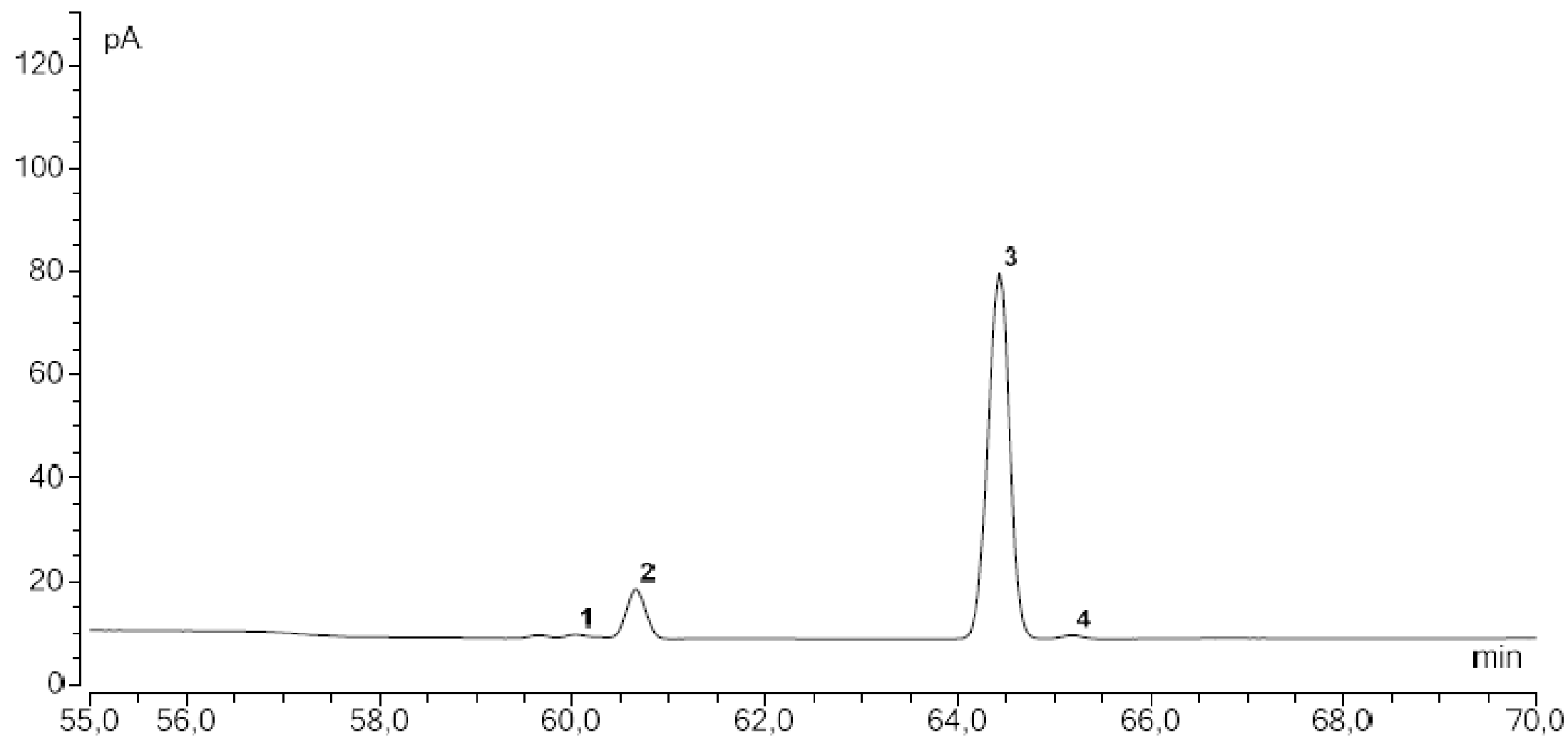

No.	Ret.Time min	area-% %	Peak Name
1	60,03	0,82	Component 1
2	60,66	10,71	Component 2
3	64,43	88,39	Component 3
4	65,19	0,08	Component 4

GC (29.5 m BGB-176, injection temperature: 220 °C; 80 °C – iso 5 min, 1 °C/min, 220 °C; iso 10 min, 0.5 bar He)

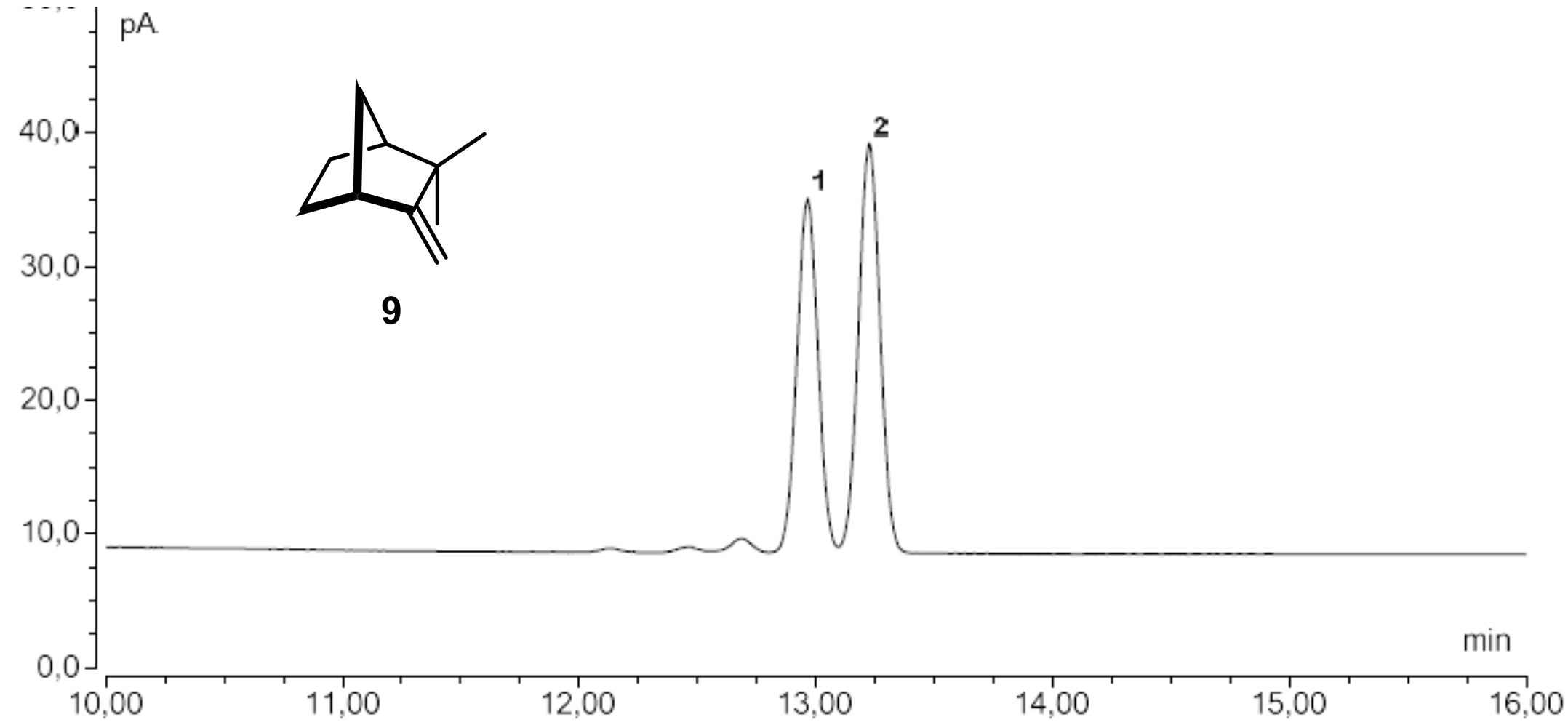

No.	Ret.Time min	area-% %	Peak Name
1	12,96	45,86	Component 1
2	13,22	54,14	Component 2

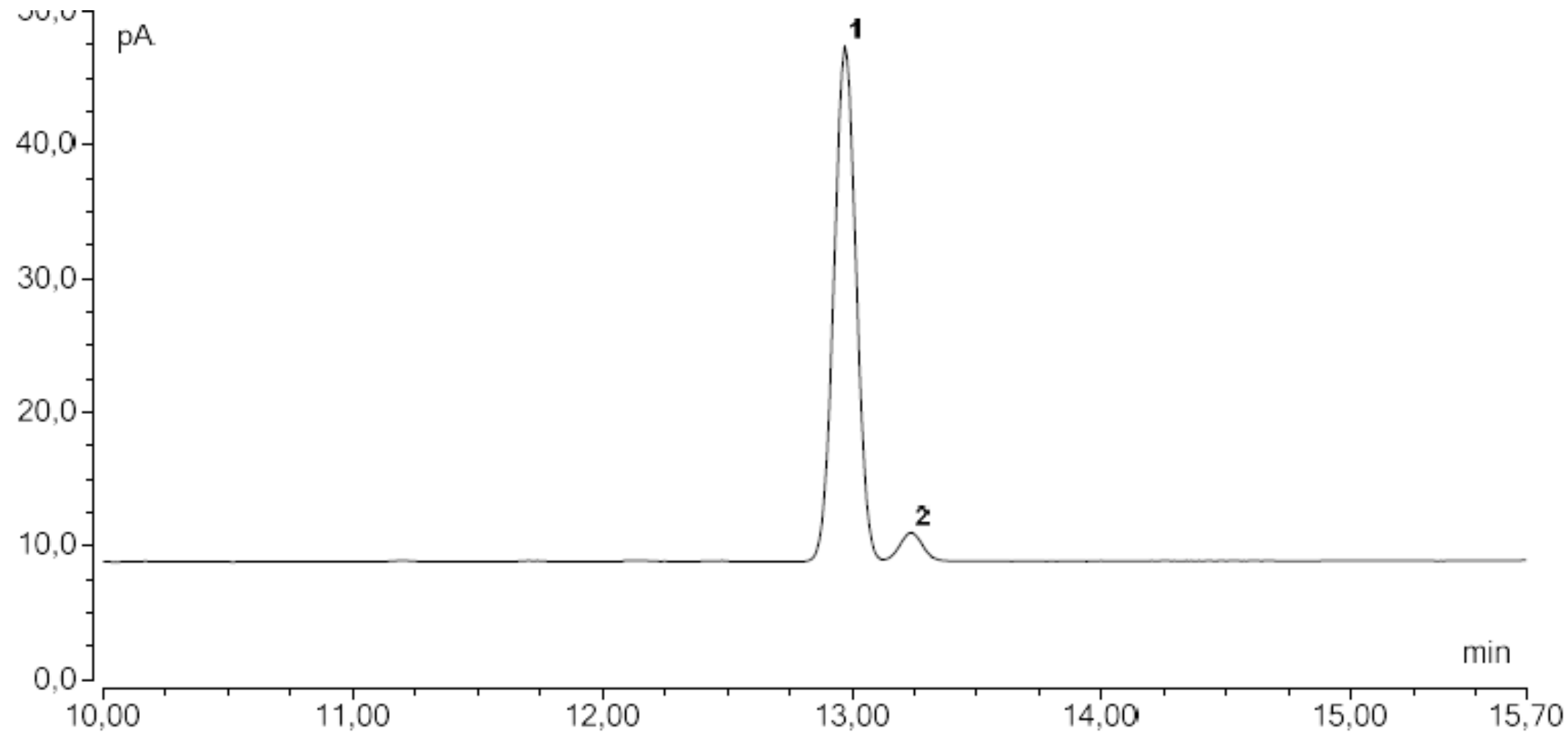

No.	Ret.Time min	area-% %	Peak Name
1	12,97	94,47	Component 1
2	13,24	5,53	Component 2

HPLC (Chiralpak IG-3, *n*-heptane/2-propanol = 99.5:0.5, 294 K, 210 nm)

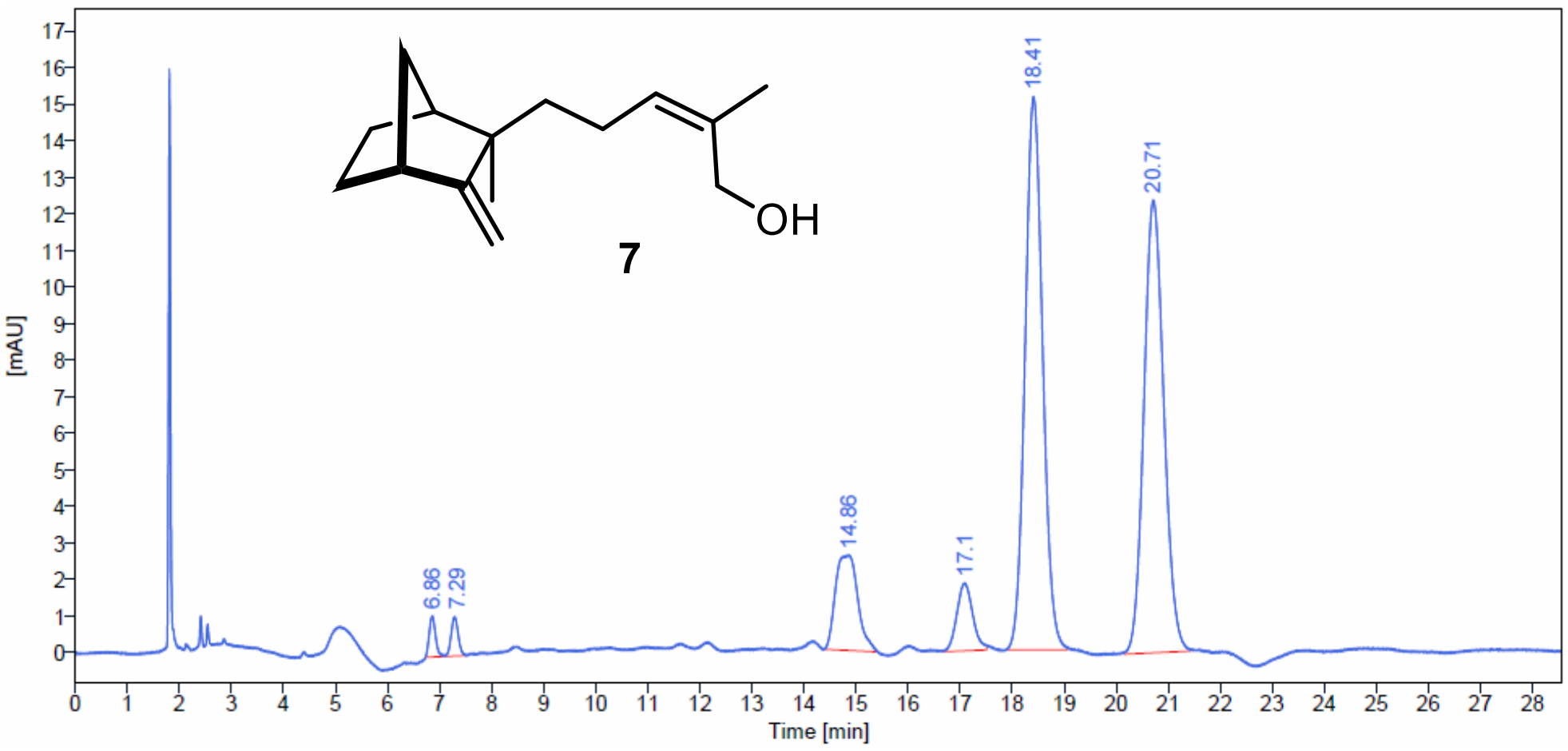

Peak	tR [min]	Peak Area [%]	Name
1	6.86	1.15	
2	7.29	1.23	
3	14.86	9.44	
4	17.10	4.77	
5	18.41	43.28	1 st enantiomer of major diastereomer
6	20.71	40.14	2nd enantiomer of major diastereomer

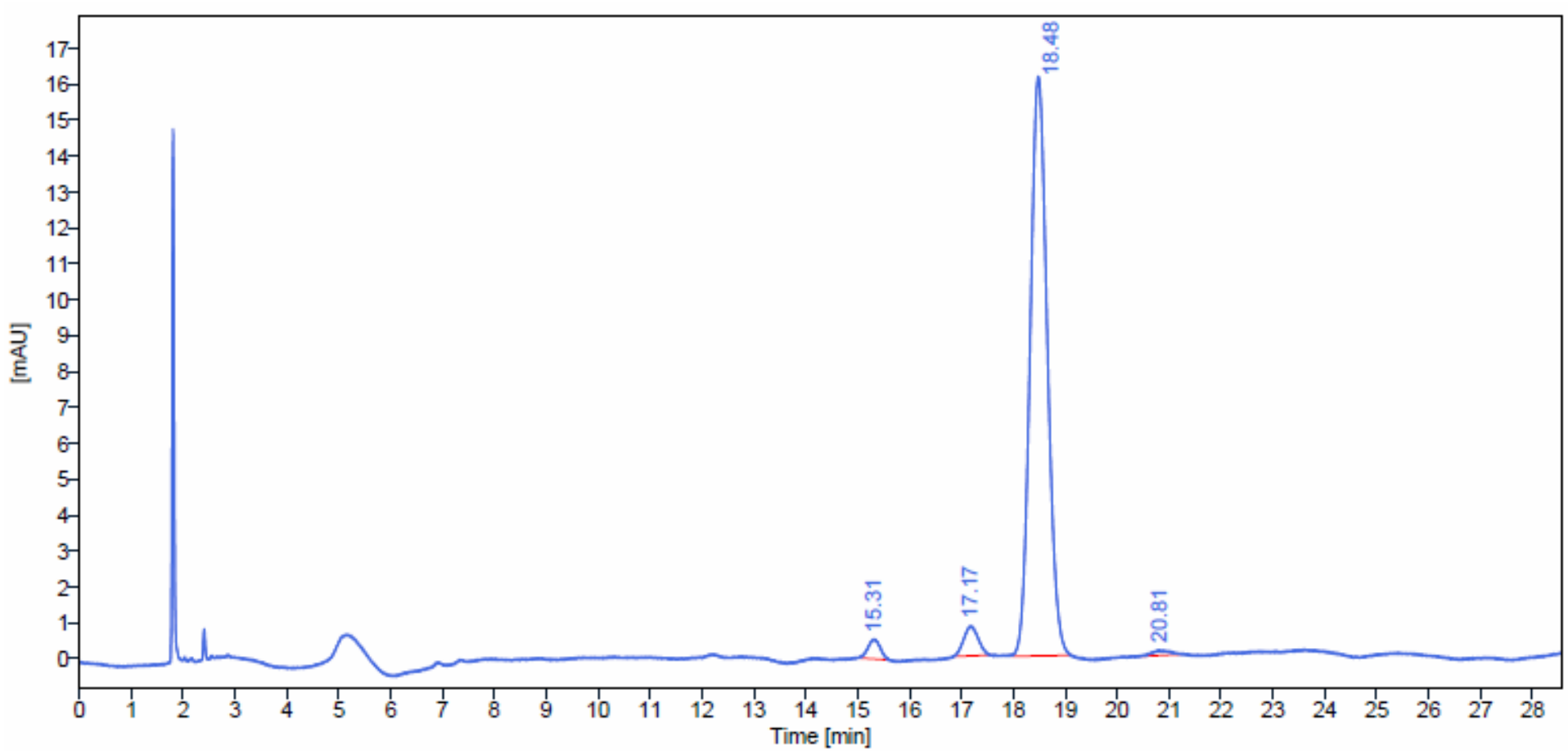

Peak	tR [min]	Peak Area [%]	Name
1	15.31	2.26	
2	17.17	4.03	
3	18.48	92.89	1 st enantiomer of major diastereomer
4	20.81	0.83	2 nd enantiomer of major diastereomer

11. References

(1) Lee, S.; Bae, H. Y.; List, B. Can a Ketone Be More Reactive than an Aldehyde? Catalytic Asymmetric Synthesis of Substituted Tetrahydrofurans. *Angew. Chem. Int. Ed.* **2018**, *57* (37), 12162–12166. https://doi.org/10.1002/anie.201806312.

(2) Kim, H.; Gerosa, G.; Aronow, J.; Kasaplar, P.; Ouyang, J.; Lingnau, J. B.; Guerry, P.; Farès, C.; List, B. A Multi-Substrate Screening Approach for the Identification of a Broadly Applicable Diels–Alder Catalyst. *Nat. Commun.* **2019**, *10* (1), 770. https://doi.org/10.1038/s41467-019-08374-z.

(3) Kaib, P. S. J.; Schreyer, L.; Lee, S.; Properzi, R.; List, B. Extremely Active Organocatalysts Enable a Highly Enantioselective Addition of Allyltrimethylsilane to Aldehydes. *Angew. Chem. Int. Ed.* **2016**, *55* (42), 13200–13203. https://doi.org/10.1002/anie.201607828.

(4) Lee, S.; Kaib, P. S. J.; List, B. Asymmetric Catalysis via Cyclic, Aliphatic Oxocarbenium Ions. *J. Am. Chem. Soc.* **2017**, *139* (6), 2156–2159. https://doi.org/10.1021/jacs.6b11993.

(5) Mahender Reddy, K.; Bhimireddy, E.; Thirupathi, B.; Breitler, S.; Yu, S.; Corey, E. J. Cationic Chiral Fluorinated Oxazaborolidines. More Potent, Second-Generation Catalysts for Highly Enantioselective Cycloaddition Reactions. *J. Am. Chem. Soc.* **2016**, *138* (7), 2443–2453. https://doi.org/10.1021/jacs.6b00100.

(6) Thirupathi, B.; Breitler, S.; Mahender Reddy, K.; Corey, E. J. Acceleration of Enantioselective Cycloadditions Catalyzed by Second-Generation Chiral Oxazaborolidinium Triflimidates by Biscoordinating Lewis Acids. *J. Am. Chem. Soc.* **2016**, *138* (34), 10842–10845. https://doi.org/10.1021/jacs.6b08018.

(7) Zhang, S.-L.; Wang, C.-Y. Highly Enantioselective Synthesis of Chiral Hydrocarbazole-4-Carbaldehyde Scaffolds and Constrained Analogue of MS-245 via Iminium Catalysis at Elevated Temperatures. *Org. Lett.* **2026**, *28* (16), 5121–5126. https://doi.org/10.1021/acs.orglett.6c00950.

(8) Hayashi, Y.; Bondzic, B. P.; Yamazaki, T.; Gupta, Y.; Ogasawara, S.; Taniguchi, T.; Monde, K. Asymmetric Diels–Alder Reaction of α-Substituted and β,β-Disubstituted α,β-Enals via Diarylprolinol Silyl Ether for the Construction of All-Carbon Quaternary Stereocenters. *Chem. Eur. J.* **2016**, *22* (44), 15874–15880. https://doi.org/10.1002/chem.201602345.

(9) Wu, Q.; Dong, Z.; Xu, J.; Yang, Z. Sulfur-Controlled and Rhodium-Catalyzed Formal (3 + 3) Transannulation of Thioacyl Carbenes with Alk-2-Enals and Mechanistic Insights. *Org. Biomol. Chem.* **2021**, *19* (14), 3173–3180. https://doi.org/10.1039/D1OB00116G.

(10) Leinung, W.; Mitschke, B.; Leutzsch, M.; Wakchaure, V. N.; Maji, R.; List, B. From Reagent to Catalyst: Dispersion-Driven Design of a General Asymmetric Transfer Hydrogenation Catalyst. *J. Am. Chem. Soc.* **2025**, *147* (20), 16722–16728. https://doi.org/10.1021/jacs.5c00641.

(11) Brenna, E.; Crotti, M.; De Pieri, M.; Gatti, F. G.; Manenti, G.; Monti, D. Chemo-Enzymatic Oxidative Rearrangement of Tertiary Allylic Alcohols: Synthetic Application and Integration into a Cascade Process. *Adv. Synth. Catal.* **2018**, *360* (19), 3677–3686. https://doi.org/10.1002/adsc.201800299.

(12) Egi, M.; Yamaguchi, Y.; Fujiwara, N.; Akai, S. Mo−Au Combo Catalysis for Rapid 1,3-Rearrangement of Propargyl Alcohols into α,β-Unsaturated Carbonyl Compounds. *Org. Lett.* **2008**, *10* (9), 1867–1870. https://doi.org/10.1021/ol800596c.

(13) Suzuki, J.; Miyano, N.; Yashiro, S.; Umezawa, T.; Matsuda, F. Total Synthesis of (−)-Kainic Acid and (+)-Allo-Kainic Acid through SmI_2 -Mediated Intramolecular Coupling between Allyl Chloride and an α,β-Unsaturated Ester. *Org. Biomol. Chem.* **2017**, *15* (31), 6557–6566. https://doi.org/10.1039/C7OB01427A.

(14) Heckenbichler, K.; Schweiger, A.; Brandner, L. A.; Binter, A.; Toplak, M.; Macheroux, P.; Gruber, K.; Breinbauer, R. Asymmetric Reductive Carbocyclization Using Engineered Ene Reductases. *Angew. Chem. Int. Ed.* **2018**, *57* (24), 7240–7244. https://doi.org/10.1002/anie.201802962.

(15) Furukawa, N.; Chen, X.; Asano, S.; Matsumoto, M.; Wu, Y.; Murata, K.; Takeuchi, A.; Tode, C.; Homma, T.; Koharazawa, R.; Usami, K.; Tie, J.-K.; Hirota, Y.; Suhara, Y. Synthesis of New Vitamin K Derivatives with a Ketone Group at the C-1′ Position of the Side Chain and Their Conversion to Menaquinone-4. *J. Mol. Struct.* **2023**, *1276*, 134614. https://doi.org/10.1016/j.molstruc.2022.134614.
(16) Polaino, S.; Herrador, M. M.; Cerdá-Olmedo, E.; Barrero, A. F. Splitting of β-Carotene in the Sexual Interaction of Phycomyces. *Org. Biomol. Chem.* **2010**, *8* (19), 4229. https://doi.org/10.1039/c0ob00321b.
(17) Söderberg, B. C.; Berry, A. K.; Jones, P. C. Reactions of Trimethylsilyl Dienol Ethers with Palladium (II) Salts: Formation of Formyl-Substituted η^3-Allylpalladium Complexes and 4-Acyloxy-2-Alkenals. *Organometallics* **1998**, *17* (6), 1069–1078. https://doi.org/10.1021/om971048x.
(18) Allgäuer, D. S.; Jangra, H.; Asahara, H.; Li, Z.; Chen, Q.; Zipse, H.; Ofial, A. R.; Mayr, H. Quantification and Theoretical Analysis of the Electrophilicities of Michael Acceptors. *J. Am. Chem. Soc.* **2017**, *139* (38), 13318–13329. https://doi.org/10.1021/jacs.7b05106.
(19) *Houben-Weyl Methods of Organic Chemistry Vol. V/1c, 4th Edition: Conjugated Dienes, Diels−Alder Reactions*, 4. 4th Edition.; Bahr, U., Müller, P., Müller-Dolezal, H., Stoltz, R., Söll, H., Eds.; Thieme: Stuttgart, 2014.
(20) Tsuji, N.; Kennemur, J. L.; Buyck, T.; Lee, S.; Prévost, S.; Kaib, P. S. J.; Bykov, D.; Farès, C.; List, B. Activation of Olefins via Asymmetric Brønsted Acid Catalysis. *Science* **2018**, *359* (6383), 1501–1505. https://doi.org/10.1126/science.aaq0445.
(21) Zhang, W.; Zhi, Y.; Huang, J.; Wu, X.; Zeng, S.; Xu, S.; Zheng, A.; Wei, Y.; Liu, Z. Methanol to Olefins Reaction Route Based on Methylcyclopentadienes as Critical Intermediates. *ACS Catal.* **2019**, *9* (8), 7373–7379. https://doi.org/10.1021/acscatal.9b02487.
(22) Mukaiyama, T.; Kitazawa, T.; Fujisawa, H. A Lewis Base-Catalyzed Hetero Diels–Alder Reaction between Aldehydes and the Danishefsky's Diene. *Chemistry Letters* **2006**, *35* (3), 328–329. https://doi.org/10.1246/cl.2006.328.
(23) Schreyer, L.; Kaib, P. S. J.; Wakchaure, V. N.; Obradors, C.; Properzi, R.; Lee, S.; List, B. Confined Acids Catalyze Asymmetric Single Aldolizations of Acetaldehyde Enolates. *Science* **2018**, *362* (6411), 216–219. https://doi.org/10.1126/science.aau0817.
(24) Breck, D. W. *Zeolite Molecular Sieves: Structure, Chemistry, and Use*; Wiley: New York, 1973.
(25) Roelofsen, D. P.; Wils, E. R. J.; Van Bekkum, H. Proton-Exchanged Type a Molecular Sieves. *J. Inorg. Nucl. Chem* **1972**, *34* (4), 1437–1448. https://doi.org/10.1016/0022-1902(72)80344-0.
(26) Lu, C.; Liu, T.; Shi, Q.; Li, Q.; Xin, Y.; Zheng, L.; Zhang, Z. Plausibility of Potassium Ion-Exchanged ZSM-5 as Soot Combustion Catalysts. *Sci. Rep.* **2017**, *7* (1), 3300. https://doi.org/10.1038/s41598-017-03504-3.
(27) Singleton, D. A.; Merrigan, S. R.; Beno, B. R.; Houk, K. N. Isotope Effects for Lewis Acid Catalyzed Diels−Alder Reactions. The Experimental Transition State. *Tetrahedron Lett.* **1999**, *40* (32), 5817–5821. https://doi.org/10.1016/S0040-4039(99)01148-X.
(28) Grimm, J. A. A.; Zhou, H.; Properzi, R.; Leutzsch, M.; Bistoni, G.; Nienhaus, J.; List, B. Catalytic Asymmetric Synthesis of Cannabinoids and Menthol from Neral. *Nature* **2023**, *615* (7953), 634–639. https://doi.org/10.1038/s41586-023-05747-9.
(29) Il'ina, I. V.; Volcho, K. P.; Korchagina, D. V.; Salakhutdinov, N. F. The Convenient Way for Obtaining Geranial by Acid-Catalyzed Kinetic Resolution of Citral. *Helv. Chim. Acta.* **2016**, *99* (5), 373–377. https://doi.org/10.1002/hlca.201500266.
(30) Baldovini, N.; Solladié, G. Application of the Asymmetric Diels–Alder Reaction of a 2-Substituted Chiral Maleate to the Formal Synthesis of (−)-β-Santalene. *Tetrahedron: Asymmetry* **2002**, *13* (8), 885–889. https://doi.org/10.1016/S0957-4166(02)00194-5.
(31) Oppolzer, W.; Chapuis, C. Asymmetric Diels−Alder Reaction of a Chiral Allenic Ester : Enantioselective Synthesis of (−)-β-Santalene. *Tetrahedron Lett.* **1983**, *24* (43), 4665–4668. https://doi.org/10.1016/S0040-4039(00)86221-8.

(32) Oppolzer, W.; Chapuis, C.; Dupuis, D.; Guo, M. Asymmetric *Diels - Alder* Reactions of Neopentyl-Ether-Shielded Acrylates and Allenic Esters: Syntheses of (−)-Norbornenone and (−)-β-Santalene. *Helv. Chim. Acta.* **1985**, *68* (8), 2100–2114. https://doi.org/10.1002/hlca.19850680803.
(33) Sonawane, H. R.; Bellur, N. S.; Ahuja, J. R.; Kulkarni, D. G. Site-Selective Rhodium(II) Acetate Mediated Intramolecular Metal-Carbene Insertions into Carbon-Hydrogen Bonds of Bicyclo[2.2.1]Heptanes: Effcient Syntheses of (+)-Albene and (−)-β-Santalene. *J. Org. Chem.* **1991**, *56* (4), 1434–1439. https://doi.org/10.1021/jo00004a019.
(34) Chapuis, C.; Skuy, D.; de Saint Laumer, J.; Brauchli, R. Endo/Exo Stereoselectivity in Diels−Alder Reactions of α,β-Dialkylated Conjugated Enals to Cyclic 1,3-Dienes: Intermediates in the Synthesis of (−)-β-Santalol and Its Analogs. *Chem. Biodivers.* **2014**, *11* (10), 1470–1516. https://doi.org/10.1002/cbdv.201400060.
(35) Abraham, R. J.; Canton, M.; Griffiths, L. Proton Chemical Shifts in NMR: Part 17. Chemical Shifts in Alkenes and Anisotropic and Steric Effects of the Double Bond. *Magn. Reson. Chem.* **2001**, *39* (8), 421–431. https://doi.org/10.1002/mrc.862.
(36) Clanton, N. A.; Wilson, N. A.; Ortiz, E.; Blumberg, S. T.; Frantz, D. E. Site-Selective Functionalization of Unactivated Allylic C–H Bonds via Direct Deprotonation with KTMP: Application to the Formal Total Synthesis of (+)-Artemisinin from Amorphadiene. *Org. Lett.* **2023**, *25* (1), 277–281. https://doi.org/10.1021/acs.orglett.2c04145.
(37) Christenson, P. A.; Willis, B. J. East Indian Sandalwood Oil. 1. Stereoselective Synthesis of (±)-β-Santalene and (±)-β-Santalol. *J. Org. Chem.* **1979**, *44* (12), 2012–2018. https://doi.org/10.1021/jo01326a026.
(38) Okugawa, H.; Ueda, R.; Matsumoto, K.; Kawanishi, K.; Kato, A. Effect of α-Santalol and β-Santalol from Sandalwood on the Central Nervous System in Mice. *Phytomedicine* **1995**, *2* (2), 119–126. https://doi.org/10.1016/S0944-7113(11)80056-5.
(39) Paudel, P.; Pandey, P.; Paris, J. J.; Ashpole, N. M.; Mahdi, F.; Tian, J.-M.; Lee, J.; Wang, M.; Xu, M.; Chittiboyina, A. G.; Khan, I. A.; Ross, S. A.; Li, X.-C. Cannabinoid Receptor Type II Ligands from Sandalwood Oil and Synthetic α-Santalol Derivatives. *J. Nat. Prod.* **2023**, *86* (7), 1786–1792. https://doi.org/10.1021/acs.jnatprod.3c00282.
(41) Krotz, A.; Helmchen, G. Total Syntheses, Optical Rotations and Fragrance Properties of Sandalwood Constituents: (−)-(*Z*)- and (−)-(*E*)-β-Santalol and Their Enantiomers, *Ent*-β-Santalene. *Liebigs Ann. Chem.* **1994**, *1994* (6), 601–609. https://doi.org/10.1002/jlac.199419940610.
(42) Maeda, S.; Harabuchi, Y.; Takagi, M.; Taketsugu, T.; Morokuma, K. Artificial Force Induced Reaction (AFIR) Method for Exploring Quantum Chemical Potential Energy Surfaces. *Chem. Rec.* **2016**, *16* (5), 2232–2248. https://doi.org/10.1002/tcr.201600043.
(43) Maeda, S.; Ohno, K.; Morokuma, K. Systematic Exploration of the Mechanism of Chemical Reactions: The Global Reaction Route Mapping (GRRM) Strategy Using the ADDF and AFIR Methods. *Phys. Chem. Chem. Phys.* **2013**, *15* (11), 3683. https://doi.org/10.1039/c3cp44063j.
(44) Anstine, D. M.; Zubatyuk, R.; Isayev, O. AIMNet2: A Neural Network Potential to Meet Your Neutral, Charged, Organic, and Elemental-Organic Needs. *Chem. Sci.* **2025**, *16* (23), 10228–10244. https://doi.org/10.1039/D4SC08572H.
(45) Neese, F. Software Update: The ORCA Program System—Version 6.0. *Wiley Interdiscip. Rev. Comput. Mol. Sci.* **2025**, *15* (2), e70019. https://doi.org/10.1002/wcms.70019.
(46) Froitzheim, T.; Müller, M.; Hansen, A.; Grimme, S. G-xTB: A General-Purpose Extended Tight-Binding Electronic Structure Method For the Elements H to Lr (Z=1–103). June 23, 2025. https://doi.org/10.26434/chemrxiv-2025-bjxvt.
(47) Grimme, S.; Hansen, A.; Ehlert, S.; Mewes, J.-M. r2SCAN-3c: A “Swiss Army Knife” Composite Electronic-Structure Method. *J. Chem. Phys.* **2021**, *154* (6), 064103. https://doi.org/10.1063/5.0040021.

(48) Weigend, F.; Ahlrichs, R. Balanced Basis Sets of Split Valence, Triple Zeta Valence and Quadruple Zeta Valence Quality for H to Rn: Design and Assessment of Accuracy. *Phys. Chem. Chem. Phys.* **2005**, *7* (18), 3297. https://doi.org/10.1039/b508541a.
(49) Mardirossian, N.; Head-Gordon, M. *ω* B97M-V: A Combinatorially Optimized, Range-Separated Hybrid, Meta-GGA Density Functional with VV10 Nonlocal Correlation. *J. Chem. Phys.* **2016**, *144* (21), 214110. https://doi.org/10.1063/1.4952647.
(50) Meng, E. C.; Goddard, T. D.; Pettersen, E. F.; Couch, G. S.; Pearson, Z. J.; Morris, J. H.; Ferrin, T. E. UCSF ChimeraX : Tools for Structure Building and Analysis. *Protein Sci.* **2023**, *32* (11), e4792. https://doi.org/10.1002/pro.4792.
(51) Blender Foundation. Blender, 2018. http://www.blender.org.
(52) Peng, Q.; Duarte, F.; Paton, R. S. Computing Organic Stereoselectivity – from Concepts to Quantitative Calculations and Predictions. *Chem. Soc. Rev.* **2016**, *45* (22), 6093–6107. https://doi.org/10.1039/C6CS00573J.
(53) Bickelhaupt, F. M.; Houk, K. N. Analyzing Reaction Rates with the Distortion/Interaction-Activation Strain Model. *Angew. Chem. Int. Ed.* **2017**, *56* (34), 10070–10086.
